# Temporal and functional profile of the transcriptional regulatory network in the early regenerative response to partial hepatectomy in the rat

**DOI:** 10.1186/1471-2164-9-527

**Published:** 2008-11-06

**Authors:** Egle Juskeviciute, Rajanikanth Vadigepalli, Jan B Hoek

**Affiliations:** 1 Department of Pathology, Anatomy and Cell Biology, Thomas Jefferson University, Philadelphia, PA 19107, USA

## Abstract

**Background:**

The goal of these studies was to characterize the transcriptional network regulating changes in gene expression in the remnant liver of the rat after 70% partial hepatectomy (PHx) during the early phase response including the transition of hepatocytes from the quiescent (G_0_) state and the onset of the G_1 _phase of the cell cycle.

**Results:**

The transcriptome of remnant livers was monitored at 1, 2, 4, and 6 hours after PHx using cDNA microarrays. Differentially regulated genes were grouped into six clusters according their temporal expression profiles. Promoter regions of genes in these clusters were examined for shared transcription factor binding sites (TFBS) by comparing enrichment of each TFBS relative to a reference set using the Promoter Analysis and Interaction Network Toolset (PAINT).

Analysis of the gene expression time series data using ANOVA resulted in a total of 309 genes significantly up- or down-regulated at *any *of the four time points at a 20% FDR threshold. Sham-operated animals showed no significant differential expression. A subset of the differentially expressed genes was validated using quantitative RT-PCR. Distinct sets of TFBS could be identified that were significantly enriched in each one of the different temporal gene expression clusters. These included binding sites for transcription factors that had previously been recognized as contributing to the onset of regeneration, including NF-κB, C/EBP, HNF-1, CREB, as well as factors, such as ATF, AP-2, LEF-1, GATA and PAX-6, that had not yet been recognized to be involved in this process. A subset of these candidate TFBS was validated by measuring activation of corresponding transcription factors (HNF-1, NK-κB, CREB, C/EBP-α and C/EBP-β, GATA-1, AP-2, PAX-6) in nuclear extracts from the remnant livers.

**Conclusion:**

This analysis revealed multiple candidate transcription factors activated in the remnant livers, some known to be involved in the early phase of liver regeneration, and several not previously identified. The study describes the predominant temporal and functional elements to which these factors contribute and demonstrates the potential of this novel approach to define the functional correlates of the transcriptional regulatory network driving the early response to partial hepatectomy.

## Background

The onset and progression of liver regeneration following acute injury reflects a complex program of responses involving growth factors, cytokines, hormones, matrix components and other factors. These extracellular mediators activate a carefully orchestrated sequence of intracellular signals resulting in a system-wide coordinated program of gene expression alterations and associated changes in the functional state of the liver cells [1-4]. Following the largely uncharacterized signals that mark the recognition of tissue damage after partial hepatectomy (PHx) and the onset of regeneration, which may include hemodynamic changes and stress signals mediated by adrenergic and purinergic agonists [5], hepatocytes emerge from the quiescent (G_0_) state to enter the pre-replicative phase of the cell cycle (G_1_) [1,2,6]. The exit from quiescence (sometimes referred to as "priming") is controlled by a wide range of signals from growth factors (HGF, TGF-α), cytokines, (tumor necrosis factor-α (TNF-α), interleukin-6) and structural components affected by proteases, such as urokinase plasminogen activator (uPA) and matrix metalloprotease-9 (MMP9) [1-4,7,8]. These and other signals result in the activation of a variety of transcription factors (TFs) important during the initial stages of liver regeneration before the onset of de novo protein synthesis and entry into the cell cycle [2]. Specific TFs, such as nuclear factor-κB (NF-κB), signal transducer and activator of transcription 3 (STAT3), CCAAT enhancer-binding protein β (C/EBP-β), and activator protein 1 (AP-1) are rapidly activated in the remnant liver within minutes to hours after PHx [9-12]. These events lead to the first phase of gene expression, referred to as the immediate early phase, which lasts for approximately 4 hours in the rat. The protooncogenes *c-fos, c-jun *and *c-myc *were among the first genes to be identified in this group [13,14]. Previous studies by Taub and colleagues identified a large set of genes participating in the immediate early response to PHx, which includes transcription factors, tyrosine phosphatases, as well as secreted and intracellular metabolic proteins [15,16].

Characterizing changes in gene expression using microarray technology has provided new insight into the regulation of liver regeneration [17-20]. Notably, a broad range of cellular processes appears to be represented among up- or down-regulated genes. Although the major emphasis in liver regeneration has been on signals that lead to cell proliferation, the response to PHx is much broader. Liver cells display a highly dynamic and coordinated response profile that affects almost every aspect of cell functioning [4]. However, our understanding of the temporal patterns of gene expression that occur during the course of liver regeneration and of the upstream regulatory signals responsible for these patterns is still limited.

In this study we used cDNA microarrays to monitor changes in gene expression at 1, 2, 4, 6 h after PHx in remnant livers in the rat. These time-points provide information on the course of events during the initiation of the regenerative response accompanying the emergence of hepatocytes from the quiescent state and the onset of the G_1 _phase [4,6]. We adopted a novel approach to analyze the microarray data that extends beyond the list of differentially expressed genes and focuses on the characterization of their transcriptional regulation, which is one of the key mechanisms by which protein expression changes are controlled. Candidate TFs responsible for differential expression profiles of the immediate early genes were characterized using the Promoter Analysis and Interaction Network Toolset (PAINT) software [21,22]. The concept driving the analysis in PAINT is that many co-expressed genes share regulatory elements, typically TF binding sites, in their promoters, leading to co-regulation. PAINT uses bioinformatics in combination with robust statistical approaches to identify the significantly enriched TREs in the promoters of the genes of interest (e.g., gene groups from cluster analysis of expression data). A key aspect of the analysis is the unbiased approach that considers all known TF binding sites as being equally probable for significance to winnow down the list of TFs from hundreds to a relatively small panel of TFs that could play a role under these experimental conditions. Based on these results, we characterize the transcriptional regulatory network interactions that drive functional responses during the early phase of regeneration after PHx.

## Results

### Liver regeneration function-relevant gene expression

**Figure 1:**
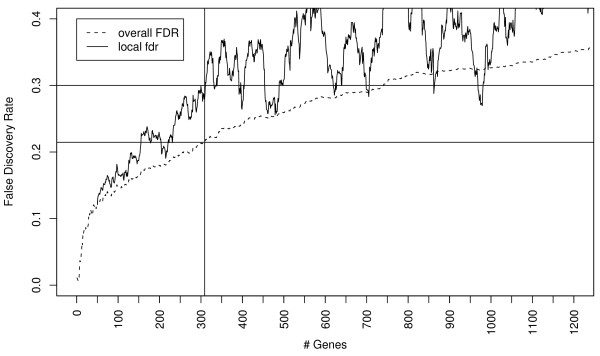
**Relationship between overall FDR, local fdr, and the number of predicted differentially expressed genes**. We chose a 30% local fdr as a threshold resulting in 309 differentially expressed genes (corresponding to a 21.4% overall FDR). Additional genes selected would be at a higher 'opportunity cost' as the local fdr is higher than 30% for the next 100 genes.

Differences in gene expression in rat liver were analyzed at 1, 2, 4 and 6 h after PHx corresponding to the transition from G_0 _and the early G_1 _phase in hepatocytes [4,6]. In a typical analysis of high-throughput gene expression data, the choice of false discovery rate (FDR) threshold is not objective, i.e., it represents an arbitrary balance between missing relevant genes due to a highly restrictive threshold, and a less restrictive threshold resulting in an increasing number of differentially-expressed genes with more false positives. In contrast, within a certain local fdr range, the number of differentially expressed genes is relatively insensitive to the choice of a particular fdr threshold [23]. Thus, the local fdr represents a robust metric of the opportunity cost (in specificity) of considering additional genes as differentially expressed (see Figure 1 for the relationship between false discovery rate estimates and the number of differentially expressed genes in our data set).

Analysis of the gene expression time series data using ANOVA resulted in a total of 309 genes significantly up- or down-regulated at *any *of the four time points at a 30% local false discovery rate threshold which corresponded to ~20% false discovery rate (FDR) threshold (Figure 1) (see Additional file 1 for detailed gene expression data and the Methods section for accession information to deposited raw data). Sham-operated animals showed only 16 genes that were significantly up-or down-regulated at 1 hr compared to control tissues (see Additional file 2). A similar number (15) of apparently differentially expressed genes was obtained when comparing the data from sets of 4 randomly selected control tissues from different animals (data not shown). Notably, the differences in gene expression found after sham surgery or between control samples did not overlap with the genes found to be responsive to PHx, suggesting that these represent random differences reflecting multiple testing error or biological variability.

**Figure 2:**
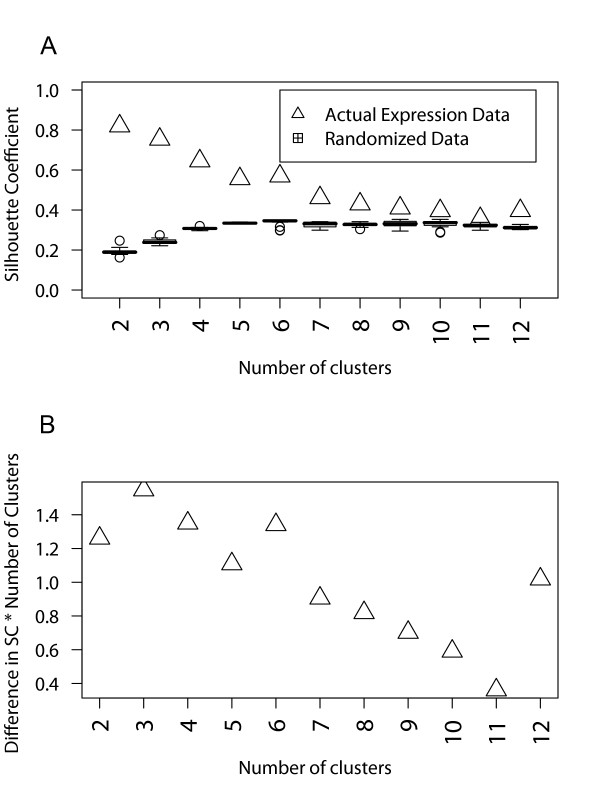
**Assessment of the gene expression clustering results using the Computational Negative Control (CNC) approach**. **(A) **For each specified number of clusters, the cluster quality metric, silhouette coefficient (SC), is evaluated and compared to that from the randomly permuted data. **(B) **Difference in SC from (A) multiplied by number of clusters shows a marked decrease at more than six clusters, indicating that SC is no longer distinct from the randomized data.

**Figure 3:**
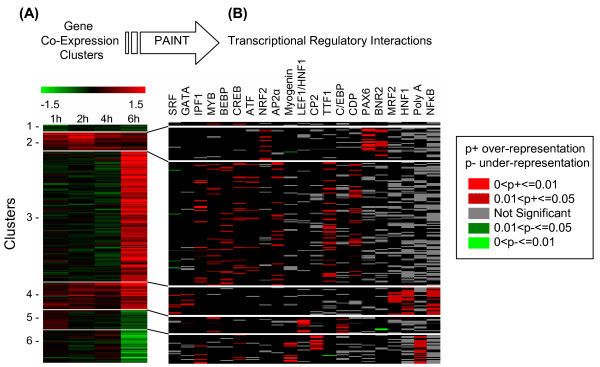
**Analysis of gene expression time series data during the onset of liver regeneration**. **(A) **Cluster analysis of the differential expression temporal profiles. The data was clustered using Partitioning Around Medoids using Pearson Correlation as the distance metric and with k = 6 (optimal number obtained from the results shown in Figure 1). Each row corresponds to a gene and each column corresponds to one of the four time points (1, 2, 4, 6 hours post partial hepatectomy). Lines demarcate the cluster boundaries. **(B) **The six clusters from (A) were analyzed for over-represented TF binding sites in the corresponding promoters using PAINT. The representative interaction matrix is shown. The rows represent the promoters and columns represent TFs. Each binding site for a TF on a promoter is marked red or grey, depending whether the *frequency of that binding site in that cluster *is statistically significantly overrepresented or not, respectively. Binding sites for several TFs known to be relevant in liver development and regeneration are enriched in distinct expression clusters. Lines indicate the mapping between the gene groups in the expression map and the corresponding promoter sets in the regulatory interaction matrix.

The 309 differentially regulated genes were clustered according their expression profiles following the Computational Negative Control (CNC) approach detailed in the Methods section. Six clusters provided maximum information on distinct temporal patterns and were well distinguishable from randomized data partitioning. Partitioning beyond six clusters reduces the confidence in the clustering result, as the performance is closer to that of randomized data (Figure 2). It should be noted that there might be additional informative temporal patterns in the data than are represented by the six clusters considered here. One limitation of the clustering algorithm employed here is that the number of clusters is user-specified, and hence, there could be genes that are considered as 'incorrectly clustered' for a given number of partitions. We have attempted to overcome this limitation by scanning a range of user-specified numbers of clusters and choosing the maximum number of patterns that are well distinguishable from clustering randomized data. The expression profiles corresponding to the six clusters are shown in Figure 3A. Approximately half of the differential regulation is comprised of up-regulation of a number of genes at the 6 hour time point (cluster 3). Several genes are also down-regulated by 6 hours (clusters 5 and 6). The early up-regulated genes are represented in clusters 2 and 4, and to some extent in cluster 5.

**Figure 4:**
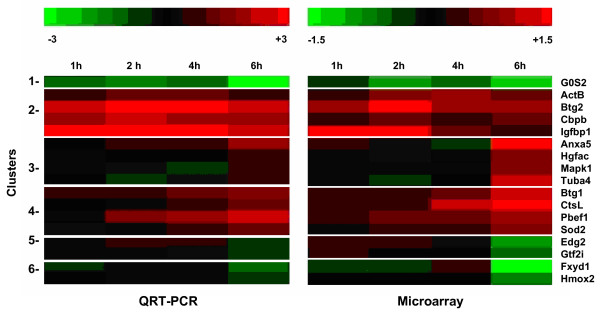
**Comparison of QRT-PCR and cDNA microarray data on 17 genes differentially expressed at 1, 2, 4 or 6 h after partial hepatectomy (PHx)**. Each row corresponds to a gene and each column corresponds to one of the four time points (1, 2, 4, 6 hours post PHx). The lines demarcate the expression cluster boundaries. The clusters correspond to the data in Figure 3.

In order to validate the differential expression of key genes across all the observed gene expression clusters QRT-PCR was performed on a total of 17 genes that represent the response profiles in the different clusters. The findings, shown in Figure 4, indicate a good concordance of the temporal profiles between the microarray and quantitative QRT-PCR results. The difference in scale between the two sets of results is typical, owing to multiple methodological factors [24,25].

**Figure 5:**
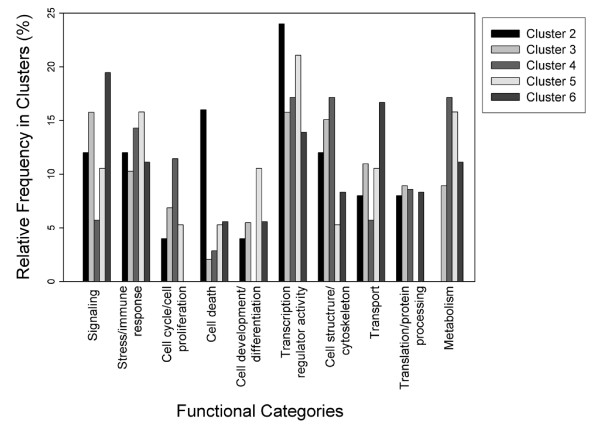
**Distribution of gene functional categories in differentially expressed clusters**. Bars correspond to the relative frequency of various gene functions in the differential expression clusters; the clusters are indicated with different fill patterns. Cluster 1 data were omitted in view of the small number of genes involved.

Functional categories were assigned to the 267 annotated differentially expressed genes based on Gene Ontology [26], following a manually curated assignment process as detailed in the supplemental data (see Additional file 3). Transcription related genes formed the most numerous category and were present in all clusters (Figure 5). We also observed rapid up-regulation of genes associated with stress response, signal transduction, and cell structure. A large number of cell proliferation-related genes were up-regulated at the 6 h time-point (clusters 3 and 4). Genes in the category Metabolism were absent from Cluster 2, which shows an early increase that is maintained or declines at later times. The range of functions we observed is expected at the initial stage of liver regeneration [18,19]. A more detailed discussion of the functional gene categories represented in the array studies is provided as supplemental text (see Additional file 4). It should be noted that we did not find GO-based functions that were over-represented (after multiple testing correction) among the differentially expressed genes. Hence, we only report the relative frequency of the functional categories (Figure 5).

### Transcriptional Regulatory Network Analysis

The Promoter Analysis and Interaction Network Tool (PAINT) is a software program designed to identify transcription factor binding sites in the promoter region of coordinately regulated genes [21]. PAINT analysis identified 22 TF binding sites enriched (FDR < 30%) in individual clusters with distinct temporal gene expression patterns (see Additional file 5). The transcriptional regulatory network obtained from the PAINT analysis is shown in Figure 3B. Binding sites for several TFs are significantly enriched (or, more infrequently, underrepresented) in each of the different gene expression clusters. Some of these TFs, e.g., NF-κB, HNF-1, CREB, ATF, GATA, and C/EBP are known to be involved in the early phase of liver regeneration from previous studies [27-30], whereas others (AP-2α, LEF-1, PAX-6) are known to contribute to the regulation of cellular processes related to proliferation and differentiation [31-33]. It should be noted that PAINT cannot differentiate between different C/EBP isoforms, which have highly conserved bZIP domains and interact with identical recognition sequences in the promoter of target genes (see below). The binding site for ATF was enriched in the group of genes that are up- or down regulated at the 6 h time-point (clusters 3 and 6). As for C/EBP isoforms, it is not possible to identify any specific ATF component candidate based on binding site information alone, since all members of the ATF family bind to the same consensus DNA sequence (TGACGTCA). ATF-3, also known as liver-regenerating factor-1 (LRF-1) is known to be highly expressed after PHx in mice [19]; however, this gene was not present on our array. Our microarray analysis identified two differentially expressed members of ATF family, namely ATF-4 and ATF-6. The expression profile of these two transcription factors suggests their potential role regulating expression of genes in clusters 3 and 6 (see supplemental text in Additional file 4 for details). The binding sites for paired box gene 6 (PAX-6) and BRN-2 were both highly enriched in cluster 2 genes. These factors are classically involved in neurogenesis and retinal development and recently PAX-6 expression was reported in hepatic oval cells under conditions where transdifferentiation into islet cells was promoted [34]. However, neither BRN-2, nor PAX-6 have previously been implicated in liver regeneration. The distribution of binding sites in our dataset suggests a role for these transcription factors as possible regulators contributing to the immediate early gene response. Binding sites for myogenin, a transcription factor involved in muscle cell differentiation, were enriched in cluster 6. The early response gene BTG-1 identified in our microarray analysis increases activity of myogenin [35].

#### Activation of Transcription Factors

**Figure 6:**
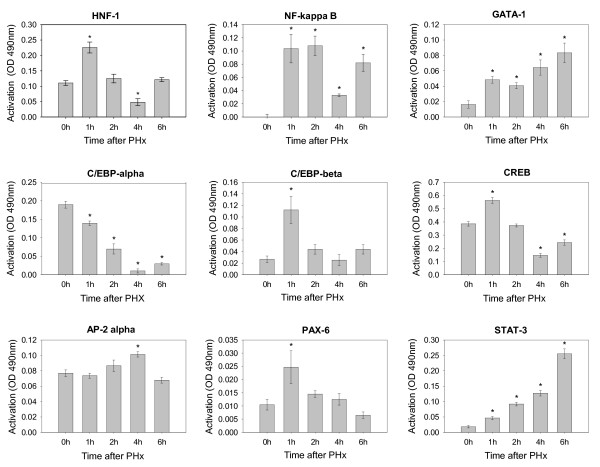
**Activation of select transcription factors after partial hepatectomy**. Transcription factor activation was monitored at different time points after PHx using TransAm NFκB p65, HNF-1, STAT-3, CREB, GATA family and C/EBP α/β kits (Active Motif, Carlsbad, CA) or TransFactor Universal Colorimetric Kit (Clontech, Mountain View, CA). In each case, 20–50 μg of nuclear extract was added per well with immobilized oligonucleotides based on the corresponding transcription factor binding site sequence. The primary antibodies were used to detect transcription factors bound to their target DNA. Addition of HRP-conjugated secondary antibody provided a colorimetric readout for quantification by spectrophotometry. At each time point, the data is normalized against a blank control sample. Error bars are based on replicate data from three animals.

In order to corroborate the PAINT analysis, we obtained time series data on the DNA binding activity detected in nuclear extracts from remnant livers for several of the transcription factors implicated by our PAINT analysis (Figure 6). We selected both transcription factors that had previously been reported to play a role in liver regeneration after partial hepatectomy (NF-κB, HNF-1, CREB, C/EBP-α and C/EBP-β) and others (AP2-α, PAX-6 and GATA) that were not known to be involved in this process. The regulatory dynamics we observe is generally consistent with the differential gene expression pattern between 1 h and 6 h post partial hepatectomy (Figure 3A). In particular, the temporal patterns of NF-κB and GATA-1 activity are consistent with the expression pattern of Cluster 4 in which the NF-κB and GATA binding sites were found to be enriched in our computational analysis. The early changes in PAX-6 activity also confirmed the potential role of this transcription factor in regulation of some of the immediate-early gene expression (Cluster 2 genes). In agreement with earlier reports, an early transient activation of NF-κB, HNF-1 and C/EBP-β was observed [11,36-38]. By contrast, C/EBP-α activity rapidly declined. Interestingly, C/EBP-α is suppressed by AP-2α [39], and the increase in the level of active AP-2α we detected at 4 h after partial hepatectomy may have contributed to that effect. The differential response of C/EBP-α and C/EBP-β after PHx has been documented before and is known to play a critical role in the onset of proliferation under the differentiated conditions of the adult liver [11,37,38,40,41]. Such a response is consistent with the temporal expression pattern of genes in Cluster 5, in which binding sites for C/EBP were enriched. The transient nature of the C/EBP-β response observed in our samples differs from some earlier reports [11,36,40,41]. However, multiple different isoforms of C/EBP-β contribute to its DNA binding activity that have differential temporal response patterns [41-43] and our analysis may be biased towards complexes exhibiting a more transient response.

It is notable that for many of the transcription factors analyzed that show an early transient increase, we observed a significant resurgence in the DNA-binding activity between 4 h and 6 h. Such a profile has been observed previously in time course studies of TF activation. For instance, Rudnick et al. [44] reported a temporal response of phospho-CREB after PHx that exactly matches the CREB-DNA binding profile shown in Fig. 6, with a further increase in phospho-CREB by 12 hrs. Similarly, an early, but transient activation of NF-κB followed by a later resurgence was reported by Diaz-Guerra et al. [45]. The resurgence phase coincides with the gene expression profile that suggests a broad range of functional changes occurring between 4 and 6 hrs after PHx. The initial 4 hr period after PHx has been attributed to the emergence of hepatocytes from G_0 _and the transition to the G_1 _phase of the cell cycle [6] and the 6 hr time point reflects the onset of the cell cycle progression.

In addition to the transcription factors identified by our PAINT analysis, we also monitored activation of STAT-3. The activation of this transcription factor after PHx was reported in the literature [9,28,46,47]. Although enrichment of the binding sites for this transcription factor was not identified by PAINT analysis, we observed a continuous increase in the level of active STAT-3 in the nuclear extract at 1–6 h after PHx. In addition, the microarray analysis showed a substantial increase in STAT-3 mRNA level at 4–6 h after PHx. Interestingly STAT-3 is one of the GATA target genes.

### Functional gene categories regulated by transcription factors

**Figure 7:**
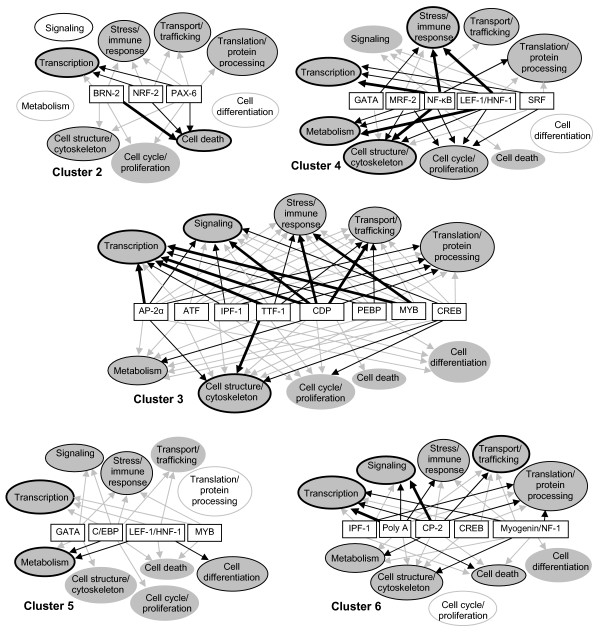
**Transcriptional regulatory networks of differentially expressed functional gene categories**. Candidate transcription factors are shown in rectangular boxes and functional categories of genes are in ovals. The strength of the connections between TFs and functional gene categories are illustrated as arrows of different shade/thickness, corresponding to the number of genes in each functional category that have binding sites for specific TFs (grey, 1(2) genes; thin black, 2(3) – 3(4) genes; heavy black, >3(4) genes, numbers in parentheses refer to cluster 3 only). The shade/thickness of borders on the ovals represents the number of genes in that category relative to the total number of genes in the cluster (thick, >15%; thin, 10–15%; grey, <10%). Open ovals are categories not associated with any significantly enriched transcription factor binding site in that cluster. Cluster 1 was omitted in view of the small number of genes involved.

Further insight into the functional consequences of the transcriptional regulatory network is obtained from Figure 7, which illustrates how the transcriptional regulators relate to functional annotations of differentially expressed gene categories in individual clusters (see also Additional file 5). The strength of the interactions (relative number of genes in different categories with promoters possessing binding sites for each transcription factor) is shown by thickness of the connecting arrows, and the predominance of individual categories in each cluster (relative number of genes in that category) is indicated by the thickness of the borders of each oval. It is apparent that the broad category of transcription related genes are distributed through all of the observed temporal expression clusters and are potential targets for the majority of identified TFs. However, other categories show more differential regulation. Interestingly, the two major early response clusters 2 and 4 show markedly distinct TF-functional category patterns. Significantly enriched TF binding sites in cluster 2 appear to regulate expression of several cell death-associated genes. Induction of both pro- and anti-apoptotic genes is an expected reaction to tissue injury. In cluster 4 enriched TF binding sites control a substantial number of genes related to cell proliferation and cell cycle control and also have dominant connections to the stress/immune response genes and the cell structure/cytoskeleton-related genes. In both late response clusters (3 and 6) TF binding sites are enriched that concentrate on process-oriented gene categories such as signaling, stress and immune response, transport and trafficking, or translation.

## Discussion

In this study, microarray gene expression data obtained during the initial 6-hour period after partial hepatectomy were used to characterize the transcriptional regulatory network that drives the onset and early progression phase of liver regeneration. Following clustering of the gene expression data, PAINT analysis was used to characterize significantly enriched TF binding sites in the different clusters to identify TFs that might have contributed to the temporal profile of gene expression obtained. TF activation could be directly confirmed by analysis of nuclear extracts. Insight into the functional role of the genes regulated by these TFs was obtained from the gene ontology analysis of TF-gene relationships. Not unexpectedly, the analysis suggests that multiple TFs coordinate to control a wide range of functions during the early phase of liver regeneration (Fig. 7). Importantly, functional categories identified by GO analysis often are broad and overlapping and should be interpreted with considerable caution. For that reason, we curated the individual assignments obtained from the GO analysis to optimize the functional associations presented in Figs. 5 and 7. A more detailed discussion of the functional categories identified in this analysis is provided as Supplemental text (see Additional file 4). In agreement with an earlier study on mice [19], sham-operated animals did not show significant changes in gene expression accompanying the early response to PHx that could not be accounted for by multiple comparison errors or animal-to-animal variability and there was no overlap with the differentially expressed genes detected after PHx.

Several previous studies reported microarray studies of gene expression changes in rodents after partial hepatectomy using a variety of platforms. The majority of these studies presented data on mice, including some that included early time points [17-20,48]. However, the onset and progression of liver regeneration after PHx is considerably slower in the mouse than in the rat [1]. Reported experimental results vary considerably between studies, both in the number and the nature of genes reported and in the number of replicates, making consistent evaluation of the statistical significance and validation of the resulting changes difficult. Therefore, these studies have not generally resulted in broader insights into the functional processes associated with these changes in gene expression. One previous study used the rat model [49], starting with the 6 hr time point. However, this study observed significant differences in only 16 (out of 4608) genes. Thus, our study is unique in presenting a robust analysis of the gene expression changes in the rat and, importantly, in using the temporal response profile to obtain information on the transcriptional regulation that drives these responses.

These results demonstrate that relevant functional information on the transcriptional control of the early response to partial hepatectomy can be obtained from the PAINT analysis of clustered microarray data. Each of the six temporally distinct gene expression clusters is characterized by a unique pattern of significantly over-represented binding sites for TFs. Activation of a selection of the candidate TFs was confirmed by oligonucleotide binding assays of nuclear extracts. Notably, there was relatively little overlap in the TFs driving the response in different temporal clusters. This is not to say that transcription factors involved in one cluster didn't play any role in the response in other clusters (e.g. note the broad presence of TF binding sites for NF-κB, HNF-1, or PolyA), but those associations didn't reach statistical significance in our analysis. It is possible that these factors contribute to the fine-regulation of the gene expression responses within clusters, but the number of differentially expressed genes in these studies was too low to identify such combinatorial control by multiple TFs with sufficient statistical weight.

As with any computational approach, it is important to note that our unbiased discovery approach using PAINT, while informative in predicting a role for novel TFs, is subject to false negatives, i.e. not all the currently known TFs in liver regeneration were present in the computational predictions. For example, STAT-3 is known to play role in liver regeneration [28,46,47] and analysis of our samples confirmed that STAT-3 activation occurred during the time frame of early responses that we investigated here. However, our PAINT analysis indicates that the differentially expressed genes were not enriched for STAT-3 target genes (based on the results from MATCH/TRANSFAC, only 2 of the 309 genes contained STAT-3 binding sites in their promoters). The STAT-3 binding site is characterized by a position weight matrix of 21 base pair length in the TRANSFAC database (Accession number M00225), with a position weight matrix similarity threshold of 0.934 for minimizing false positives in finding the binding site on genomic sequences. According to the TRANSFAC database, this similarity threshold corresponds to a false negative rate of ~20%. Hence, the unexpectedly low number of predicted STAT-3 binding sites may not be due to our choice of parameters in MATCH, but appear to arise from a combination of the pattern matching algorithm and the STAT-3 binding site description. As the databases and associated computational tools continue to improve, false negatives like these are likely to decrease, although they will probably never be completely eliminated. Nevertheless, our computational approach successfully predicted many known and novel TFs as playing a role in the onset of liver regeneration, several of which were experimentally validated (Figure 6).

## Conclusion

Our study highlights significant candidate mechanisms for transcriptional control of specific genes and gene clusters and classifies these by functional category, but does not identify individual genes as actively being controlled by these TFs. Further studies using chromatin immunoprecipitation (ChIP) or related methodologies will be required to validate the role of each TF in individual gene responses, which will further clarify the role of individual TFs in the functional changes occurring after PHx. Nevertheless, the study points not only to the complexity of the transcriptional control of the early response to PHx, but also suggests that there is a clear underlying organization to the temporal response of genes in different functional categories that is driven by transcriptional regulation. The data reported here should provide a basis for a more detailed analysis of the role of each of these transcription factors to the regulation of individual genes and gene categories. However, these findings also emphasize the fact that the study of any individual factor will not capture the systemic nature of the regulatory machinery that drives the regenerative response of the liver to partial hepatectomy.

This conclusion is also relevant for the recognition that multiple cell types contribute to regenerative responses in the remnant liver. The analysis of gene expression profiles in total tissue extracts from the remnant liver incorporates contributions from parenchymal cells, Kupffer cells, endothelial cells stellate cells and other non-parenchymal cells. Although parenchymal cells contribute approximately 70% of the total cell number and 90% of tissue mass in the liver, robust gene expression responses in non-parenchymal cells may occur that reach the threshold for detection in our microarray studies. Similarly, differences in zonal distribution across the liver acinus exist that are difficult to capture in such in vivo studies. However, the response to partial hepatectomy (and by extension the response to other forms of liver injury) is by nature a systemic response of the whole tissue, in which the contributions of different cell types are integrated to generate the coordinated temporal pattern of regeneration. Our analysis is an effort to capture this integrated response profile by focusing on the system-wide gene expression and regulation by transcription factors. A better understanding of this systemic response profile will ultimately be a critical step in mobilizing the regenerative potential of the liver for therapeutic purposes.

## Methods

### Animals and Tissues

Adult male Sprague-Dawley rats (275–350 g) were anesthetized and subjected to two-thirds PHx by ligation and resection of the median and left-lateral lobes, following standard procedures [50]. Liver sections removed by partial hepatectomy (PHx) were collected within 30 sec of starting the surgery and used both as controls (time = 0) and as individual reference material for each animal to reduce the error introduced by animal-to-animal variability, thereby improving the sensitivity and specificity in the statistical analysis. At 1, 2, 4, and 6 hours following PHx, rats were anaesthetized again and remnant liver samples were harvested. Sham-operated animals were treated similarly, except that livers were palpated for 30 sec without removing liver tissue. Liver samples intended for RNA isolation (4 animals/time-point) were frozen in liquid nitrogen immediately after harvest. Liver samples collected for nuclear extract preparation (3 animals/time-point) were processed immediately after the surgery without freezing. Total RNA was isolated using TRIzol reagent (Invitrogen, Carlsbad, CA) according to the manufacturer's instructions. Nuclear extract was prepared using Nuclear Extract Kit (Active Motif, Carlsbad, CA) according to the manufacturer's instructions.

### cDNA Microarrays

Frozen glycerol stocks of *Escherichia coli *containing individual sequence verified rat cDNA clones were purchased from Research Genetics (Huntsville, AL). cDNA clone inserts were amplified by PCR directly from the clones in culture with primers specific to the vector sequences flanking the insert cDNA. All cDNA clones spotted on the microarray were generated by PCR using GF200 primer pairs, therefore all clones contains 110 bps vector sequence on their GF200 forward primer side. Vector probes generated with a GF200 forward primer on an empty pT7T3Pac vector in RT reaction were used as a universal reference for all clones as a control for cDNA on the microarray [51]. 5 μl (150 – 350 ng) of amplified PCR product was re-suspended in an equal volume of DMSO. The array-ready cDNAs were printed on polylysin-coated glass microscope slides (Full Moon Biosystems, Sunnyvale, CA) using a *Micro*Grid II microarrayer (Biorobotics Inc., Woburn, MA). The array contains 9084 target clones that had annotation in the Unigene database linking them to known genes (for the complete list see Additional file 6) spotted in duplicate and 72 blank controls (no DNA spotted). The microarray is divided into 48 subarrays; each containing 380 spots (19 × 20). After printing, the slides were allowed to dry. Spotted DNA was bound to the surface of the slide by baking at 80°C for 2 hours. Slides were stored in an airtight box until hybridization was performed.

### Probe Preparation, Microarray Hybridization and Data Acquisition

Fluorescently labeled probes were prepared following an indirect cDNA labeling protocol. Rat liver RNA was labeled with Cy5, whereas Cy3-labeled vector probe was used as a reference for each sample [51]. Reference and experimental probes were combined and competitively hybridized to microarrays for 18 hours at 37°C. One microarray assay was conducted for each PHx sample and one for its control, for a total of 32 arrays. Additional microarrays were prepared for sham surgery samples. Slides were scanned using ScanArray 5000 fluorescent scanner (Perkin Elmer, Waltham, MA). The resulting images were quantified using ScanArray Express v2.2 software with the Adaptive Threshold segmentation (Perkin Elmer, Waltham, MA). Raw quantitated array data was normalized using the print-tip lowess and scale normalization algorithms [52].

MIAME compliant microarray data are deposited at , **accession # **GSE7415 (PHx) and GSE9137 (sham).

### ANOVA model

The normalized gene expression data was analyzed using a mixed-effects ANOVA response model for each gene using the statistical software package in R following Pavlidis and Scholtens [53,54]. We examined the effects of the following two relevant variables and their interactions on the gene expression levels in the regenerating liver: **(1) **partial hepatectomy (PHx or control), **(2) **time following PHx (1, 2, 4, and 6 hours). These variables were considered as fixed effects, whereas the biological variability (animal-to-animal effects) was considered as a random effect in the ANOVA response model. We estimated the parameters in the ANOVA response model to these fixed- and random-effect variables and sought genes with statistically significant parameters. For each gene, we evaluated the statistical significance of the observed expression against the null hypothesis that PHx has no effect on the gene expression at *any *of the four time points. Multiple testing was accounted for with the overall false discovery rate (FDR) controlling procedure of Benjamini and Hochberg [55] and a local false discovery rate (fdr) [23]. The local fdr estimates the false positive rate within a neighborhood of genes (chosen as 50 here). The local fdr estimate was used in conjunction with the overall FDR to limit the overall number of false positives in order to derive a more robust list of differentially expressed genes.

### Cluster Analysis

We employed the Partitioning Around Medoids (PAM) [56] clustering algorithm using Pearson Correlation as the distance metric to cluster the temporal expression profiles of the differentially regulated genes. This partitioning scheme relies on medoids and hence is robust to outliers, if any, in the data. The number of desired clusters is specified as an input parameter to the algorithm. We have investigated a range of clusters from 2 to 12 in number and evaluated the quality of the clustering results using a Computational Negative Control (CNC) approach [57,58]. Typically, a cluster quality metric called *silhouette coefficient *(SC) is utilized to assess the quality of the clustering results: the closer the SC is to 1, the better the quality. Our CNC approach takes this a step further and assesses the performance of the clustering results by comparing SC from clustering original data with that from the randomly permutated data (destroying any inherent structure): the larger the difference between the quality metric between the original data clustering vs. randomized data clustering, the higher the confidence in the resulting clusters. This information was utilized to explore different parameters in the clustering algorithm, i.e., number of clusters specified in PAM. We sought the largest number of *meaningful *clusters that are distinct from random.

### Transcriptional Regulatory Network Analysis

We employed PAINT [21] to analyze the gene groups derived from the cluster analysis of the gene expression time series data. Differentially expressed genes were mapped to unique promoters and the TF binding sites were analyzed in PAINT using TRANSFAC^® ^Profession 10.1 database and associated MATCH^® ^tool [59]. In each gene group, the over-representation ('enrichment') of TF binding site frequency on multiple promoters was assessed using Fisher's Exact Test and corrected for multiple testing using a False Discovery Rate estimate [55]. The over-representation p-value computed was based on the probability of occurrence of the observed TF binding site frequency in a random sample compared to a reference. For the analysis presented here, we employed the promoters corresponding to all the genes in the microarray as the reference in order to correctly account for the selection bias as our microarrays do not span the entire set of genes in the Ensembl database. The results on the binding sites were mapped to the corresponding TFs based on the data column 'Factor Name' in the results from MATCH^® ^tool.

### Quantitation of Gene Expression after Partial Hepatectomy using Real Time PCR

QRT-PCR analysis was performed to verify the microarray data. The cDNA templates were synthesized from total RNA using SuperScript II reverse transcriptase and oligo(dT) primer (Invitrogen, Carlsbad, CA). Quantitative analysis of gene expression was performed on ABI Prism 7000 (Applied Biosystems, Foster City, CA) using 2× SYBR Green master mix (Applied Biosystems, Foster City, CA). The primer pairs used in this analysis are shown in Table 1.

**Table 1:** Primers used in QRT-PCR

**Gene Name**	**Symbol**		**Primer Sequence**
Actin, beta	Actb	Forward Reverse	5'-TCGCTGACAGGATGCAGAAG-3' 5'-ACATCTGCTGGAAGGTGGACA-3'
Annexin A5	Anxa5	Forward Reverse	5'-CTGCCTACCTTGCAGAGACC-3' 5'-CGTGGCGAAGTTCTTCCTAA-3'
B-cell translocation gene 1, anti-proliferative	Btg1	Forward Reverse	5'-GAGGATGGCTCCATCTGTGT-3' 5'-TTTTGGAAGGGCTTGTTCTG-3'
B-cell translocation gene 2	Btg2	Forward Reverse	5'-GCTCTGTGGTTCTGCCATTTC-3' 5'-CAAAGCTGTGAATCGCTCCAG-3'
Cathepsin L	CtsL	Forward Reverse	5'-CAAAGACCGGAACAACCACT-3' 5'-CACTCAGAGACGGGTTTGGT-3'
CCAAT/enhancer binding protein (C/EBP), beta	Cebpb	Forward Reverse	5'-GGGTTGTTGCTGTTGATGTTT-3' 5'-CGAAACGGAAAAGGTTCTCA-3'
Endothelial differentiation G-protein-coupled receptor, 2	Edg2	Forward Reverse	5'-CCATGAACGAACAACAGTGC-3' 5'-AGCATGATGAACACGCAGAC-3'
FXYD domain-containing ion transport regulator 1	Fxyd1	Forward Reverse	5'-GCAGGAACCAGATCCATTCA-3' 5'-CCCAGTTCTCTGCTGTTGGT-3'
G0/G1 switch gene 2	G0s2	Forward Reverse	5'-CCCAGAGCTCAGATGGAAAG-3' 5'-ACTAGACCGAGCACCACACC-3'
General transcription factor 2i	Gtf2i	Forward Reverse	5'-GGGATGGCTAGCAAAATCAA-3' 5'-CGAACGGTAGAGGTCTGAGG-3'
Glyceraldehyde-3-phosphate dehydrogenase	Gapdh	Forward Reverse	5'-AGTTCAACGGCACAGTCAAG-3' 5'-GTGGTGAAGACGCCAGTAGA-3'
Heme oxygenase 2	Hmox2	Forward Reverse	5'-TCAGTTTTCCAGGCCTTTTG-3' 5'-TTAGAGTGCTGTGGCAGGTG-3'
Hepatocyte growth factor activator	Hgfac	Forward Reverse	5'-TGAGTCGACCTCAACTGCAC-3' 5'-AGCCGTTCCCAATGTAGATG-3'
Insulin-like growth factor binding protein 1	Igfbp1	Forward Reverse	5'-GCCAGGGAGCCTGTGTACTA-3' 5'-AGCAGCTGTTCCTCTGTCA-3'
Mitogen activated protein kinase 1	Mapk1	Forward Reverse	5'-CCTACGGCATGGTTTGTTCT-3' 5'-TCTCATGTCTGAAGCGCAGT-3'
Pre-B-cell colony enhancing factor 1	Pbef1	Forward Reverse	5'-CTGTTCCTGAGGGCTCTGTC-3' 5'-TTGTGGCCACTGTAATTGGA-3'
Superoxide dismutase 2, mitochondrial	Sod2	Forward Reverse	5'-TCTGTGGGAGTCCAAGGTTC-3' 5'-ACACATCAATCCCCAGCAGT-3'
Tubulin, alpha 4	Tuba4	Forward Reverse	5'-AGGAGATCATCGACCCAGTG-3' 5'-ACAGAAAGCCGTTCCATCAG-3'

### Transcription factor activation analysis

Activation of HNF-1, NFκB, STAT-3, CREB, GATA-1 CEBP/α and CEBP/β was identified as changes in DNA binding activity of the transcription factors in nuclear extracts using kits from Active Motif, Carlsbad, CA, as per the manufacturer's instructions. AP-2α and PAX-6 activity was assessed using the TransFactor Universal Colorimetric Kit (Clontech, Mountain View, CA) with biotinylated oligos containing binding sequence 5'-ACC GCC TGA GGC GTT A-3' (AP-2α) 5'-CTG ACC TGG AAC T-3' (PAX-6) and AP-2α and PAX-6 antibodies (Santa Cruz Biotechnology, Santa Cruz, CA).

## Authors' contributions

EJ carried out gene expression experiments and transcription factors activation assays, participated in drafting the manuscript and statistical analysis. RV performed statistical and PAINT analysis. JBH conceived of the study, and participated in its design and coordination, and drafting the manuscript. All authors read and approved the final manuscript.

## Supplementary Material

Additional file 1 **Table S1**. Liver regeneration associated changes in gene expression revealed by cDNA microarray analysis. Click here for file

Additional file 2 **Table S2**. Sham surgery associated changes in gene expression revealed by cDNA microarray analysis. Click here for file

Additional file 3 **Table S3**. TF binding sites enriched (FDR < 30%) in individual clusters. Click here for file

Additional file 4 **Supplemental text**. Analysis of functional gene categories changing after partial hepatectomy. Click here for file

Additional file 5 **Table S4**. Distribution of TF binding sites in the functional differentially expressed gene categories. Click here for file

Additional file 6 **Table S5**. Clones spotted on the array. Click here for file
