# Supplementary material for: Temporal and functional profile of the transcriptional regulatory network in the early regenerative response to partial hepatectomy in the rat
Source: BMC Genomics. 2008 Nov 6;9:527. doi: 10.1186/1471-2164-9-527 (PMC2613928; doi:10.1186/1471-2164-9-527)
Supplement: Additional file 1 — Table S1. Liver regeneration associated changes in gene expression revealed by cDNA microarray analysis. [file 1471-2164-9-527-S1.doc]

**Table S1**. Liver regeneration associated changes in gene expression revealed by cDNA microarray analysis. Significant up- (red) or down (green) regulation is highlighted.

| **GeneBank**  **Accession Number** | **Gene Name** | **Official Symbol** | **ANOVA *P* Value** | **Log_2_ Intensity Ratio** | | | | **Cluster** |
| --- | --- | --- | --- | --- | --- | --- | --- | --- |
|  |  |  |  | **1h** | **2h** | **4h** | **6h** |  |
| **Signal transduction** | | | | | | | | |
| CK842947 | Anti-Mullerian hormone type 2 receptor | Amhr2 | 0.00455 | -0.1 | 0.1 | -0.2 | **1.5** | 3 |
| BI296778 | CDC-like kinase 3 | Clk3 | 0.00050 | -0.1 | 0.2 | -0.3 | **1.6** | 3 |
| AA818983 | Diacylglycerol kinase, beta | Dgkb | 0.00514 | -0.1 | -0.1 | -0.3 | **0.7** | 3 |
| AA819366 | Dual specificity phosphatase 6 | Dusp6 | 0.00671 | 0.2 | 0.8 | 0.0 | **1.1** | 4 |
| BI285473 | Extracellular matrix protein 1 | Ecm1 | 0.00066 | 0.2 | 0.1 | 0.0 | **0.6** | 3 |
| BF545611 | Endothelial differentiation, lysophosphatidic acid G-protein-coupled receptor, 2 | Edg6 | 0.00141 | 0.3 | **0.5** | 0.2 | **-0.9** | 5 |
| AI071705 | GH3 domain containing | Ghdc | 0.00707 | -0.1 | 0.2 | **-0.4** | **0.1** | 3 |
| BG379318 | G protein-coupled receptor 83 | Gpr83 | 0.00372 | -0.1 | 0.0 | -0.3 | **1.6** | 3 |
| BE119649 | Hepatocyte growth factor activator | Hgfac | 0.00281 | 0.1 | -0.2 | 0.1 | **0.7** | 3 |
| AI454643 | Insulin-like growth factor binding protein 1 | Igfbp1 | 0.00139 | **1.5** | **1.6** | **0.6** | 0.2 | 2 |
| AA818337 | Inositol 1,4,5-triphosphate receptor 1 | Itpr1 | 0.00417 | -0.1 | -0.2 | -0.3 | **0.7** | 3 |
| CK842366 | Latent transforming growth factor beta binding protein 3 | Ltbp3 | 0.00605 | 0.0 | -0.1 | 0.0 | **1.0** | 3 |
| BG380475 | Mitogen activated protein kinase kinase 5 | Map2k5 | 0.00114 | 0.3 | 0.3 | 0.1 | **1.0** | 4 |
| BF555997 | Mitogen activated protein kinase 1 | Mapk1 | 0.00590 | -0.1 | -0.1 | -0.1 | **0.8** | 3 |
| BI290194 | Non-catalytic region of tyrosine kinase adaptor protein 1 | Nck1 | 0.00657 | -0.1 | 0.0 | -0.4 | **0.9** | 3 |
| AI044491 | Olfactomedin-like 2B | Olfml2b | 0.00417 | 0.0 | -0.2 | **0.6** | **-1.7** | 6 |
| AI501164 | Oncostatin M specific receptor | Osmr | 0.00161 | 0.3 | 0.1 | 0.0 | **-1.2** | 5 |
| BG371888 | Phosducin | Pdc | 0.00888 | -0.1 | -0.1 | **-0.7** | **1.7** | 3 |
| BI275649 | Phosducin-like 3 | Pdcl3 | 0.00202 | 0.1 | 0.0 | -0.2 | **1.3** | 3 |
| AI071374 | Platelet derived growth factor receptor, beta polypeptide | Pdgfrb | 0.00639 | -0.1 | 0.2 | -0.1 | **-0.5** | 6 |
| AA818100 | Protein phosphatase 2a, catalytic subunit, alpha isoform | Ppp2ca | 0.00395 | 0.2 | 0.0 | -0.1 | **0.8** | 3 |
| AI059606 | Protein phosphatase 4, regulatory subunit 1 | Ppp4r1 | 0.00070 | -0.1 | 0.0 | -0.2 | **1.0** | 3 |
| AI044238 | Protein kinase C, delta | Prkcd | 0.00823 | 0.2 | 0.1 | 0.3 | **-1.1** | 6 |
| BI274659 | Prolactin-like protein N | Prlpn | 0.00166 | 0.0 | -0.1 | -0.4 | **1.0** | 3 |
| AI071249 | Protein tyrosine phosphatase 4a1 | Ptp4a1 | 0.00000 | **0.7** | **1.4** | **0.7** | **0.6** | 2 |
| AI555816 | Rhomboid, veinlet-like 7 | Rrhbdl7 | 0.00624 | -0.2 | -0.1 | 0.2 | **-0.6** | 6 |
| CK839738 | V-ros UR2 sarcoma virus oncogene homolog 1 | Ros1 | 0.00424 | -0.3 | **-0.3** | -0.2 | **0.8** | 3 |
| BE101082 | Sclerostin | Sost | 0.00313 | -0.2 | **0.5** | **0.4** | -0.2 | 2 |
| AA956939 | Spleen tyrosine kinase | Syk | 0.00517 | 0.0 | -0.1 | **0.3** | **1.2** | 3 |
| **Neural signaling** | | | | | | | | |
| BE119983 | Gamma-aminobutyric acid A receptor, alpha 5 | Gabra5 | 0.00074 | 0.3 | **-0.5** | -0.3 | **1.8** | 3 |
| BE109632 | GRIP1 associated protein 1 | Gripap1 | 0.00296 | 0.0 | -0.2 | -0.2 | **1.2** | 3 |
| BF391593 | Homer homolog 1 | Homer1 | 0.00376 | 0.1 | **-0.5** | 0.2 | **-0.8** | 6 |
| BF549707 | Islet cell autoantigen 1 | Ica1 | 0.00505 | -0.1 | 0.0 | -0.3 | **0.6** | 3 |
| BI289803 | Polycystic kidney disease 1 homolog | Pkd1 | 0.00553 | 0.0 | 0.1 | -0.2 | **1.3** | 3 |
| CK840699 | Proopiomelanocortin, beta (endorphin, beta) | Pomc | 0.00504 | -0.1 | -0.1 | 0.0 | **-0.5** | 6 |
| BI298288 | Syntaxin 1A | Stx1a | 0.00562 | 0.0 | 0.0 | 0.0 | **-1.6** | 6 |

**Table S1** (continued).

| **GeneBank**  **Accession Number** | **Gene Name** | **Official Symbol** | **ANOVA *P* Value** | **Log_2_ Intensity Ratio** | | | | **Cluster** |
| --- | --- | --- | --- | --- | --- | --- | --- | --- |
|  |  |  |  | **1h** | **2h** | **4h** | **6h** |  |
| **Blood pressure regulation** | | | | | | | | |
| BI276266 | Angiotensin 1 converting enzyme | Ace | 0.00326 | -0.1 | 0.1 | -0.2 | **0.7** | 3 |
| **Stress/immune response** | | | | | | | | |
| AA955106 | Aldehyde dehydrogenase family 1, member A1 | Aldh1a1 | 0.00298 | 0.4 | 0.1 | -0.1 | **0.7** | 4 |
| BF546771 | Aldehyde dehydrogenase family 1, subfamily A2 | Aldh1a2 | 0.00090 | 0.2 | 0.0 | 0.0 | **-0.5** | 5 |
| AA957411 | Ankyrin repeat domain 24 | Ankrd17 | 0.00514 | 0.3 | 0.0 | 0.0 | **-0.5** | 5 |
| CK839422 | Arsenic (+3 oxidation state) methyltransferase | As3mt | 0.00289 | 0.3 | -0.1 | -0.2 | **1.2** | 3 |
| AA858513 | Complement component 1, q subcomponent binding protein | C1QBP | 0.00365 | 0.1 | 0.0 | 0.0 | **1.1** | 3 |
| AW522819 | Complement component 5, receptor 1 | C5r1 | 0.00815 | -0.2 | 0.0 | 0.0 | **0.4** | 3 |
| BE106615 | Cd200 antigen | Cd200 | 0.00068 | -0.2 | -0.1 | -0.3 | **2.0** | 3 |
| BF405160 | Cold inducible RNA binding protein | Cirbp | 0.00052 | -0.2 | 0.0 | -0.3 | **1.0** | 3 |
| BG373643 | Cytochrome P450, 4a12 | Cyp4a12 | 0.00615 | 0.0 | -0.2 | 0.1 | **1.2** | 3 |
| AA957658 | Cytochrome P450, 4b1 | Cyp4b1 | 0.00888 | **0.5** | -0.2 | 0.0 | -0.2 | 5 |
| CK841564 | Flavin containing monooxygenase 2 | Fmo2 | 0.00007 | 0.1 | 0.0 | -0.2 | **2.2** | 3 |
| BE113034 | HesB protein | HesB | 0.00323 | -0.4 | 0.0 | 0.2 | **0.7** | 3 |
| BF521858 | Heme oxygenase (decycling) 2 | Hmox2 | 0.00647 | 0.0 | 0.0 | 0.0 | **-0.7** | 6 |
| BF551538 | Histidine-rich glycoprotein | Hrg | 0.00188 | -0.3 | -0.4 | -0.3 | **-1.1** | 6 |
| BF549866 | Heat shock factor 2 | Hsf2 | 0.00483 | -0.2 | 0.2 | 0.1 | **1.6** | 3 |
| BG380317 | Heat shock protein 1 | Hspd1 | 0.00482 | 0.0 | 0.0 | 0.0 | **1.0** | 3 |
| AW535223 | K-kininogen | Kngk | 0.00606 | 0.1 | **0.6** | 0.2 | **2.1** | 4 |
| BF543953 | Mast cell antigen 32 | Mca32 | 0.00254 | -0.4 | -0.1 | -0.1 | **-0.4** | 1 |
| AA900218 | metallothionein 1A | Mt1A | 0.00004 | **1.1** | **1.5** | **1.0** | **1.6** | 2 |
| BF556086 | metallothionein 3 | Mt3 | 0.00121 | -0.3 | **0.9** | **1.2** | **1.8** | 4 |
| AA996887 | Nitrogen fixation cluster-like | NifU | 0.00494 | 0.2 | -0.1 | -0.2 | **0.3** | 3 |
| BF555573 | NAD(P)H dehydrogenase, quinone 1 | Nqo1 | 0.00742 | 0.1 | -0.2 | -0.2 | **1.4** | 3 |
| BF552706 | NAD(P)H dehydrogenase, quinone 2 | Nqo2 | 0.00203 | -0.2 | -0.2 | **-0.8** | **0.9** | 3 |
| AI705421 | RT1 class I, CE16 | RT1-CE16 | 0.00460 | 0.0 | -0.2 | -0.1 | **1.4** | 3 |
| AI535132 | RT1 class I, CE7 | RT1-CE7 | 0.00833 | 0.1 | 0.0 | -0.1 | **-0.5** | 6 |
| BF550957 | Superoxide dismutase 2, mitochondrial | Sod2 | 0.00719 | 0.1 | 0.2 | **0.3** | **0.8** | 4 |
| CK842049 | Thrombomodulin | Thbd | 0.00686 | 0.3 | **0.5** | 0.0 | **1.2** | 4 |
| CK840114 | Transformation related protein 53 binding protein 1 | Trp53bp1 | 0.00032 | **0.9** | **0.8** | 0.2 | **0.4** | 2 |
| BI290005 | Uncoupling protein 3 | Ucp3 | 0.00579 | -0.1 | -0.2 | **1.0** | **-1.8** | 6 |
| BE107282 | Ischemia related factor vof-16 | Vof16 | 0.00448 | 0.0 | -0.1 | -0.1 | **0.6** | 3 |
| AA899151 | Zinc finger protein 36 | Zfp36 | 0.00004 | **0.6** | **1.0** | **0.4** | **0.2** | 2 |
| **Cell cycle/cell proliferation** | | | | | | | | |
| BF565994 | A disintegrin-like and metalloprotease with thrombospondin type 1 motif, 1 | Adamts1 | 0.00321 | **0.5** | **0.6** | 0.0 | **0.7** | 4 |
| BI286150 | Breast cancer metastasis-suppressor 1 | Brms1 | 0.00093 | 0.0 | -0.2 | -0.1 | **1.5** | 3 |
| BG377456 | CTF18, chromosome transmission fidelity factor 18 homolog | Chtf18 | 0.00719 | -0.2 | -0.1 | -0.2 | **-0.2** | 1 |
| BI287277 | Cullin 7 | Cul7 | 0.00020 | 0.2 | 0.1 | -0.4 | **1.0** | 3 |
| AI029014 | Cysteine and glycine-rich protein 2 | Csrp2 | 0.00638 | 0.2 | 0.2 | 0.0 | **0.6** | 4 |

**Table S1** (continued).

| **GeneBank**  **Accession Number** | **Gene Name** | **Official Symbol** | **ANOVA *P* Value** | **Log_2_ Intensity Ratio** | | | | | **Cluster** | |
| --- | --- | --- | --- | --- | --- | --- | --- | --- | --- | --- |
|  |  |  |  | **1h** | **2h** | **4h** | **6h** | |  |  |
| BF522722 | DNA polymerase epsilon subunit 3 | Pole3 | 0.00545 | -0.2 | -0.2 | -0.1 | | **1.3** | | 3 |
| BF406636 | G0/G1 switch gene 2 | G0s2 | 0.00068 | -0.4 | **-1.0** | **-0.6** | | **-1.2** | | 1 |
| BF542233 | M-phase phosphoprotein | Mpp8 | 0.00426 | **0.3** | 0.2 | 0.1 | | **-1.3** | | 5 |
| BF556565 | Neuroblastoma, suppression of tumorigenicity 1 | Nbl1 | 0.00512 | 0.0 | 0.2 | **-0.7** | | **0.4** | | 3 |
| AW527882 | Neurofibromatosis 2 | Nf2 | 0.00052 | -0.1 | -0.1 | -0.3 | | **1.5** | | 3 |
| BI275165 | NHP2-like protein 1 (High mobility group-like nuclear protein 2 homolog 1) | Nhp2l1 | 0.00026 | 0.0 | 0.0 | -0.1 | | **0.3** | | 3 |
| AA926158 | Pre-B-cell colony enhancing factor 1 | Pbef1 | 0.00056 | 0.4 | **0.5** | **0.6** | | **1.0** | | 4 |
| AI113008 | Proteoglycan 4 | Prg4 | 0.00368 | 0.0 | 0.1 | -0.3 | | **1.0** | | 3 |
| BI292639 | Prothymosin alpha | Ptma | 0.00094 | -0.1 | -0.2 | -0.3 | | **1.0** | | 3 |
| AI454691 | Telomeric repeat binding factor 2 | Terf1 | 0.00000 | **-0.8** | -0.2 | 0.0 | | -0.1 | | 1 |
| AI602197 | Ubiquitin-conjugating enzyme E2S | Ube2i | 0.00847 | 0.1 | -0.3 | 0.1 | | **1.0** | | 3 |
| AA964763 | UTP20, small subunit processome component, homolog | Utp20 | 0.00000 | -0.1 | -0.2 | 0.4 | | **2.4** | | 3 |
| AA819392 | B-cell translocation gene 1, anti-proliferative | Btg1 | 0.00268 | 0.4 | **0.5** | **0.5** | | **1.3** | | 4 |
| AI146192 | B-cell translocation gene 2, anti-proliferative | Btg2 | 0.00011 | **1.0** | **2.6** | **1.0** | | **1.0** | | 2 |
| **Cell death** | | | | | | | | | | |
| BG372706 | Amyloid beta (A4) precursor protein-binding, family B, member 3 | Apbb3 | 0.00105 | 0.3 | 0.1 | 0.1 | | **1.5** | | 4 |
| AA818665 | Amyloid beta (A4) precursor-like protein 2 | Aplp2 | 0.00164 | 0.1 | 0.1 | 0.0 | | **0.9** | | 3 |
| BE110683 | Annexin A5 | Anxa5 | 0.00053 | 0.4 | -0.2 | -0.3 | | **1.5** | | 3 |
| AI385294 | BCL2/adenovirus E1B 19 kDa-interacting protein 3 | Bnip3 | 0.00637 | **0.5** | **0.5** | 0.2 | | **0.3** | | 2 |
| AI044631 | CD3 antigen, gamma polypeptide | Cd3g | 0.00205 | 0.3 | 0.2 | 0.1 | | **-0.5** | | 5 |
| BF552914 | Feminization 1 homolog b | Fem1b | 0.00324 | 0.1 | -0.1 | 0.2 | | **-0.6** | | 6 |
| AW534133 | Transformation related protein 53 inducible nuclear protein 1 | Trp53inp1 | 0.00391 | **0.4** | **0.6** | 0.2 | | 0.2 | | 2 |
| AI113283 | Transmembrane protein 23 | Tmem23 | 0.00429 | 0.3 | **0.3** | **0.3** | | 0.0 | | 2 |
| AA957270 | Tumor necrosis factor receptor superfamily, member 12a | Tnfrsf12a | 0.00569 | 0.1 | **0.5** | **0.4** | | **0.3** | | 2 |
| BE113431 | Tumor necrosis factor superfamily, member 5-induced protein 1 | Tnfsf5ip1 | 0.00141 | -0.1 | -0.2 | 0.0 | | **-0.7** | | 6 |
| BF558799 | Tumor necrosis factor, alpha-induced protein 8 | Tnfaip88 | 0.00343 | -0.3 | 0.0 | 0.1 | | **0.8** | | 3 |
| **Cell development/differentiation** | | | | | | | | | | |
| AA965204 | Complement component 1, s subcomponent | C1s | 0.00661 | 0.2 | -0.1 | 0.0 | | **0.9** | | 3 |
| BF554418 | COP9 (constitutive photomorphogenic) homolog, subunit 2 | Cops2 | 0.00535 | -0.2 | 0.0 | -0.1 | | **0.4** | | 3 |
| BF544850 | COP9 (constitutive photomorphogenic) homolog, subunit 7a | Cops7a | 0.00415 | 0.0 | -0.1 | -0.2 | | **0.5** | | 3 |
| AA955549 | Delta-like 3 | Dll3 | 0.00259 | 0.0 | **-0.5** | 0.3 | | **1.0** | | 3 |
| BE107225 | Disabled homolog 2 | Dab2 | 0.00715 | 0.0 | 0.1 | -0.4 | | **0.6** | | 3 |
| BF557660 | Microrchidia | Morc | 0.00792 | 0.0 | -0.2 | 0.0 | | **1.1** | | 3 |
| AI576682 | neuronal guanine nucleotide exchange factor | Ngef | 0.00865 | 0.0 | -0.1 | -0.1 | | **1.1** | | 3 |
| AI059154 | Notchless gene homolog | Nle1 | 0.00181 | 0.2 | 0.0 | -0.1 | | **-0.5** | | 5 |
| BF403834 | Numb gene homolog | Numb | 0.00170 | -0.3 | 0.0 | -0.1 | | **1.1** | | 3 |
| AI045342 | Pecanex-like 3 | Pcnxl3 | 0.00332 | 0.0 | -0.2 | 0.0 | | **-0.6** | | 6 |
| BF554026 | Vang, van gogh-like 1 | Vangl1 | 0.00149 | 0.0 | 0.0 | 0.3 | | **-0.8** | | 6 |

**Table S1** (continued).

| **GeneBank**  **Accession Number** | **Gene Name** | **Official Symbol** | **ANOVA *P* Value** | **Log_2_ Intensity Ratio** | | | | **Cluster** |
| --- | --- | --- | --- | --- | --- | --- | --- | --- |
|  |  |  |  | **1h** | **2h** | **4h** | **6h** |  |
| AI044919 | Wingless-type MMTV integration site family, member 2B | Wnt2b | 0.00491 | **0.4** | 0.1 | -0.2 | **-0.6** | 5 |
| CK838531 | Yip1 domain family, member 3 | Yipf3 | 0.00490 | 0.2 | **0.7** | **0.8** | **0.6** | 2 |
| **Chromosome organization** | | | | | | | | |
| AA819782 | H3 histone, family 3B | Hh3f3b | 0.00158 | 0.3 | **0.9** | **0.6** | **0.4** | 2 |
| BF525152 | Histone cluster 2, H2aa1 | Hist2h2aa1 | 0.00700 | 0.0 | -0.2 | 0.0 | **1.8** | 3 |
| AA964338 | SMC4 structural maintenance of chromosomes 4-like 1 | Smc4l1 | 0.00858 | -0.4 | 0.0 | -0.1 | **0.8** | 3 |
| BF544108 | SMC6 structural maintenance of chromosomes 6-like 1 | Smc6l1 | 0.00268 | -0.4 | 0.0 | 0.1 | **1.3** | 3 |
| **Transcription** | | | | | | | | |
| BI282081 | Activating transcription factor 4 | Atf4 | 0.00068 | 0.3 | **0.6** | **0.7** | **0.7** | 4 |
| AI059385 | Activating transcription factor 6 | Atf6 | 0.00564 | 0.4 | 0.0 | 0.0 | **-0.4** | 5 |
| AI072185 | Ankyrin repeat and SOCS box-containing protein 2 | Asb2 | 0.00201 | -0.1 | -0.1 | -0.1 | **-0.5** | 6 |
| AI030166 | Basic transcription element binding protein 1 (Kruppel-like factor 9) | Klf9 | 0.00711 | 0.1 | 0.4 | 0.0 | **1.0** | 4 |
| BI289386 | BCoR protein (BCL-6 corepressor) | Broc | 0.00000 | 0.0 | 0.0 | -0.3 | **2.1** | 3 |
| AW529356 | CCAAT/enhancer binding protein (C/EBP), beta | Cebpb | 0.00046 | **0.4** | **0.6** | 0.2 | **0.5** | 2 |
| CK841018 | Cofactor required for Sp1 transcriptional activation, subunit 3 | Crsp3 | 0.00458 | 0.0 | -0.2 | -0.4 | **1.5** | 3 |
| AA900368 | Core promoter element binding protein (Kruppel-like factor 6) | Klf6 | 0.00029 | 0.0 | **0.7** | **0.6** | **1.6** | 4 |
| AI059856 | Cryptochrome 2 (photolyase-like) | Cry2 | 0.00796 | **-0.5** | -0.4 | 0.3 | **-1.4** | 6 |
| AI112834 | D site albumin promoter binding protein | Dbp | 0.00790 | -0.4 | 0.0 | -0.1 | **0.6** | 3 |
| AI029203 | DNA-damage inducible transcript 3 | Ddit3 | 0.00001 | 0.1 | 0.1 | -0.2 | **1.1** | 3 |
| BF550577 | Elongation factor RNA polymerase II 2 | Ell2 | 0.00537 | 0.5 | **0.9** | **0.9** | **0.7** | 2 |
| BF546918 | FBJ osteosarcoma oncogene B | Fosl2 | 0.00840 | -0.3 | -0.1 | 0.0 | **1.2** | 3 |
| AI111628 | Forkhead box E1 (thyroid transcription factor 2) | Foxe1 | 0.00403 | 0.2 | -0.1 | -0.1 | **-0.6** | 5 |
| BE111515 | Forkhead box O1A | Foxo1a | 0.00783 | -0.1 | -0.1 | -0.1 | **0.6** | 3 |
| AI070190 | General transcription factor 2 I | Gtf2i | 0.00199 | 0.2 | 0.0 | 0.0 | **-0.6** | 5 |
| BE112211 | GLIS family zinc finger 2 | Glis1 | 0.00464 | 0.1 | -0.2 | 0.1 | **-0.2** | 6 |
| AA955827 | Homeo box A5 | Hoxa5 | 0.00239 | -0.1 | 0.0 | -0.2 | **0.6** | 3 |
| AI044131 | Jun-B oncogene | Junb | 0.00077 | 0.4 | **0.8** | **0.4** | 0.1 | 2 |
| BF546577 | Kruppel-like factor 3 | Klf3 | 0.00657 | 0.1 | -0.1 | 0.2 | **-0.6** | 6 |
| AI602501 | LIM and cysteine-rich domains 1 | Lmcd1 | 0.00472 | 0.0 | -0.1 | 0.0 | **0.9** | 3 |
| BF523563 | LPS-induced TNF factor | Litaf | 0.00454 | **0.4** | **0.4** | 0.1 | -0.2 | 2 |
| BE109596 | Mixed lineage leukemia gene homolog 2 protein | Mll2 | 0.00720 | **0.6** | -0.1 | -0.3 | **1.2** | 3 |
| BE101385 | Myelocytomatosis viral oncogene homolog | Myc | 0.00269 | **0.5** | **0.7** | **0.2** | **0.2** | 2 |
| BE110651 | Putative DNA/chromatin binding motif | Plu1 | 0.00636 | 0.1 | -0.1 | -0.3 | **1.6** | 3 |
| AA996525 | POU domain, class 3, transcription factor 4 | Pou3f4 | 0.00285 | -0.2 | -0.1 | -0.1 | **-0.6** | 1 |
| BI285687 | Pre-mRNA processing factor 8 | Prpf8 | 0.00441 | -0.1 | -0.1 | -0.4 | **0.9** | 3 |
| AI059832 | RD RNA-binding protein | Rdbp | 0.00483 | 0.2 | -0.2 | 0.1 | **-0.5** | 5 |
| BF559853 | Ring finger protein 10 | Rfp10 | 0.00107 | -0.1 | 0.1 | 0.0 | **0.9** | 3 |
| AI059580 | Ring finger protein 141 | rnf141 | 0.00791 | -0.2 | -0.2 | 0.2 | **1.0** | 3 |
| BF566561 | RIO kinase 2 | Riok2 | 0.00688 | -0.2 | 0.0 | -0.2 | **0.5** | 3 |
| BF556538 | RNA binding motif protein 14 | Rbm14 | 0.00399 | 0.0 | 0.1 | **-0.4** | **0.9** | 3 |

**Table S1** (continued).

| **GeneBank**  **Accession Number** | **Gene Name** | **Official Symbol** | **ANOVA *P* Value** | **Log_2_ Intensity Ratio** | | | | **Cluster** |
| --- | --- | --- | --- | --- | --- | --- | --- | --- |
|  |  |  |  | **1h** | **2h** | **4h** | **6h** |  |
| BF550542 | Scm-like with four mbt domains 1 | Sfmbt1 | 0.00100 | -0.1 | 0.0 | -0.2 | **1.3** | 3 |
| CK839299 | SERTA domain containing 1 | Sertad1 | 0.00873 | 0.0 | -0.1 | -0.2 | **-0.7** | 6 |
| AI045179 | Signal transducer and activator of transcription 3 | Stat3 | 0.00208 | -0.4 | 0.2 | **0.5** | **0.5** | 4 |
| BE110739 | Small optic lobes homolog | Solh | 0.00850 | 0.0 | -0.1 | -0.4 | **1.1** | 3 |
| BE104767 | Splicing factor, arginine/serine-rich 12 | Sfrs12 | 0.00081 | 0.3 | 0.4 | **0.7** | **1.5** | 4 |
| AA998705 | Topoisomerase I binding, arginine/serine-rich | Topors | 0.00011 | 0.2 | -0.1 | **1.4** | **2.0** | 4 |
| BI283445 | Tripartite motif-containing 27 | Trim27 | 0.00405 | -0.2 | 0.0 | 0.1 | **1.1** | 3 |
| CK842675 | Zinc finger protein 180 | Zfp180 | 0.00291 | 0.3 | 0.2 | **-0.7** | **1.5** | 3 |
| CK843606 | zinc finger protein 324 | Zfp324 | 0.00719 | -0.2 | -0.3 | 0.0 | **0.9** | 3 |
| **Cell structure/cytoskeleton** | | | | | | | | |
| AA900769 | Actin, alpha | Acta | 0.00553 | -0.1 | **0.8** | **0.9** | **0.6** | 2 |
| AI070848 | Actin, beta | Actb | 0.00738 | 0.3 | 0.8 | **1.0** | **0.6** | 2 |
| BI297592 | Rho GTPase activating protein 17 | Arhgap17 | 0.00235 | -0.3 | -0.2 | -0.2 | **0.9** | 3 |
| AI029319 | Calmodulin regulated spectrin-associated protein 1 | Camsap1 | 0.00001 | 0.2 | -0.1 | -0.3 | **-0.9** | 6 |
| AA963841 | Centaurin, delta 2 | Centd2 | 0.00216 | 0.0 | -0.2 | -0.1 | **1.4** | 3 |
| BF549935 | Cofilin 1 | Cfl1 | 0.00362 | 0.1 | 0.3 | 0.2 | **0.4** | 4 |
| AA819522 | Calponin 3, acidic | Cnn3 | 0.00220 | -0.2 | -0.2 | 0.3 | **0.8** | 3 |
| BF550885 | Cortactin binding protein 2 | Cttnbp2 | 0.00274 | 0.1 | -0.1 | 0.0 | **0.6** | 3 |
| BG378140 | Dystroglycan 1 | Dag1 | 0.00476 | -0.3 | -0.1 | -0.2 | **1.6** | 3 |
| CK840357 | Dynactin 3 | Dctn3 | 0.00040 | -0.1 | -0.1 | 0.0 | **0.8** | 3 |
| AA998140 | Dynactin 4 | Dctn4 | 0.00176 | 0.1 | 0.0 | -0.2 | **1.7** | 3 |
| AA957145 | Destrin | Dstn | 0.00026 | 0.1 | 0.2 | 0.1 | **0.5** | 4 |
| BF544124 | Dystrobrevin, beta | Dtnb | 0.00709 | 0.0 | 0.1 | 0.3 | **-0.7** | 6 |
| CK840640 | EBNA1 binding protein 2 | Ebna1bp2 | 0.00798 | 0.1 | 0.0 | -0.6 | **1.9** | 3 |
| BI292191 | Keratin complex 1, acidic, gene 19 | Krt1-19 | 0.00337 | 0.0 | 0.1 | -0.3 | **1.3** | 3 |
| CK845362 | Microtubule-associated protein 4 | Map4 | 0.00242 | 0.1 | 0.2 | -0.1 | **1.6** | 3 |
| AI703855 | PDZ domain containing 3 | Pdzk3 | 0.00247 | 0.1 | -0.1 | -0.1 | **1.0** | 3 |
| BE108510 | Polyamine modulated factor 1 binding protein 1 | Pmfbp1 | 0.00843 | 0.1 | -0.2 | -0.2 | **1.7** | 3 |
| CK842227 | Ras homolog gene family, member G | RhoG | 0.00734 | 0.1 | -0.2 | -0.2 | **1.0** | 3 |
| BI289636 | Ras homolog gene family, member J | RhoJ | 0.00031 | -0.2 | 0.2 | **0.8** | **-1.3** | 6 |
| BF559046 | Related RAS viral oncogene homolog 2 | RRAS2 | 0.00218 | **1.7** | **2.5** | **1.3** | **0.7** | 2 |
| BF414279 | Septin 2 | Sept2 | 0.00626 | -0.3 | 0.0 | -0.2 | **1.5** | 3 |
| AA964924 | Septin 4 | Sept4 | 0.00284 | 0.0 | -0.1 | **-0.5** | **0.4** | 3 |
| AA875313 | Septin 9 | Sept9 | 0.00311 | 0.4 | 0.1 | 0.0 | **0.9** | 4 |
| AA964882 | Sarcoglycan, | Sgcb | 0.00632 | -0.3 | 0.0 | -0.2 | **0.9** | 3 |
| AA818246 | Small membrane protein 1 | Smp1 | 0.00605 | 0.2 | **-0.3** | 0.0 | **0.9** | 3 |
| BG381575 | Stathmin-like 3 | Stmn3 | 0.00057 | 0.2 | -0.2 | 0.1 | **-0.2** | 5 |
| BF395575 | Tubulin, alpha 4 | Tuba4 | 0.00003 | -0.1 | -0.2 | 0.0 | **1.1** | 3 |
| CK840796 | Vezatin | Vezt | 0.00701 | 0.0 | -0.1 | -0.3 | **1.3** | 3 |
| BF547727 | Vimentin | Vim | 0.00103 | **0.4** | 0.2 | 0.3 | **0.4** | 4 |
| **Cell adhesion** | | | | | | | | |
| BF562140 | Adhesion regulating molecule 1 | Adrm1 | 0.00150 | 0.0 | -0.2 | -0.3 | **0.9** | 3 |
| BF549190 | Myelin protein zero-like 1 | Mpzl1 | 0.00405 | 0.0 | 0.4 | 0.1 | **1.1** | 4 |

**Table S1** (continued).

| **GeneBank**  **Accession Number** | **Gene Name** | **Official**  **Symbol** | **ANOVA *P* Value** | **Log_2_ Intensity Ratio** | | | | **Cluster** |
| --- | --- | --- | --- | --- | --- | --- | --- | --- |
|  |  |  |  | **1h** | **2h** | **4h** | **6h** |  |
| BE097213 | Sparc/osteonectin, cwcv and kazal-like domains proteoglycan 1 | Spock1 | 0.00204 | 0.0 | 0.0 | -0.2 | **1.1** | 3 |
| BF556916 | Spondin 2, extracellular matrix protein | Spon2 | 0.00122 | 0.1 | -0.1 | -0.3 | **0.9** | 3 |
| AA819385 | Syndecan 4 | Sdc4 | 0.00468 | **0.8** | **0.5** | 0.3 | **0.8** | 4 |
| **Transport** | | | | | | | | |
| CK839239 | Dynein, cytoplasmic, heavy chain 1 | Dync1h1 | 0.00034 | 0.2 | 0.0 | -0.3 | **1.2** | 3 |
| BF395685 | FXYD domain-containing ion transport regulator 1 | Fxyd1 | 0.00708 | **-0.5** | -0.3 | 0.3 | **-1.9** | 6 |
| AI113133 | Leucine rich repeat and sterile alpha motif containing 1 | Lrsam1 | 0.00497 | 0.0 | -0.1 | 0.1 | **-0.7** | 6 |
| BI288790 | Lysosomal-associated protein transmembrane 4A | Laptm4a | 0.00407 | 0.1 | -0.1 | -0.2 | **1.2** | 3 |
| AA900609 | Matrix metalloproteinase 23 | Mmp23 | 0.00549 | 0.0 | 0.1 | **-0.7** | **0.4** | 3 |
| BF525130 | Potassium voltage-gated channel, subfamily H, member 2 | Kcnh2 | 0.00170 | 0.2 | -0.1 | -0.2 | **0.9** | 3 |
| BG381311 | Solute carrier family 13 (sodium-dependent citrate transporter), member 5 | Slc13a5 | 0.00093 | 0.2 | **0.4** | 0.2 | **1.0** | 4 |
| AA859020 | Solute carrier family 2 (facilitated glucose transporter), member 4 | Slc2a4 | 0.00370 | 0.3 | -0.2 | -0.1 | **0.7** | 3 |
| AA957759 | solute carrier family 22 (organic cation transporter), member 18 | Slc22a18 | 0.00410 | -0.1 | -0.1 | -0.1 | **1.0** | 3 |
| BE103366 | Solute carrier family 25 (mitochondrial carrier, phosphate carrier), member 25 | Slc25a25 | 0.00005 | **0.8** | **1.1** | 0.3 | **0.4** | 2 |
| AA963102 | Solute carrier family 38, member 2 | Slc38a2 | 0.00040 | **0.5** | **0.9** | **0.3** | **0.5** | 2 |
| BF390320 | Solute carrier family 4, member 3 | Slc4a3 | 0.00441 | 0.0 | 0.0 | -0.1 | **1.2** | 3 |
| BF566991 | Solute carrier family 6 (neurotransmitter transporter, taurine), member 6 | Slc6a6 | 0.00705 | 0.3 | 0.1 | -0.1 | **1.1** | 3 |
| BF567888 | Transient receptor potential cation channel, subfamily M, member 3 | Trpm3 | 0.00723 | -0.1 | 0.1 | -0.3 | **1.3** | 3 |
| BF559886 | Translocase of outer mitochondrial membrane 20 homolog | Tomm20 | 0.00445 | 0.2 | **0.4** | 0.2 | **0.5** | 4 |
| BF420146 | Translocase of outer mitochondrial membrane 22 homolog | Tomm22 | 0.00008 | -0.2 | -0.2 | **-0.4** | **1.4** | 3 |
| BI297863 | Transthyretin | Ttr | 0.00444 | -0.1 | -0.3 | 0.1 | **-1.1** | 6 |
| AA858662 | Tyrosine 3-monooxygenase/tryptophan 5-mono-oxygenase activation protein, zeta polypeptide | Ywhaz | 0.00021 | -0.2 | -0.1 | 0.0 | **1.6** | 3 |
| AI070424 | Zinc finger, DHHC domain containing 3 | Zdhhc3 | 0.00668 | 0.0 | -0.1 | 0.1 | **-0.5** | 6 |
| **Vesicle-mediated transport** | | | | | | | | |
| BG378061 | ADP-ribosylation factor 3 | Arf3 | 0.00781 | 0.0 | -0.2 | -0.2 | **0.8** | 3 |
| BF558595 | ADP-ribosylation factor 6 | Arf6 | 0.00879 | 0.0 | -0.1 | 0.0 | **1.0** | 3 |
| CK839304 | ATPase, H+ transporting, V1 subunit G isoform 2 | Atp6v1g2 | 0.00438 | **0.4** | 0.0 | -0.2 | 0.1 | 5 |
| BF555071 | N-ethylmaleimide sensitive fusion protein attachment protein alpha | Napa | 0.00523 | 0.2 | 0.0 | 0.0 | **-0.6** | 5 |
| CK843306 | RAB2, member RAS oncogene family-like | Rab2l | 0.00754 | 0.0 | 0.1 | -0.1 | **1.5** | 3 |
| AA997773 | Ras and Rab interactor 3 | Rin3 | 0.00713 | -0.3 | 0.0 | -0.1 | **1.1** | 3 |
| AA957824 | RER1 homolog | Rer1 | 0.00106 | 0.1 | -0.1 | **0.4** | **-0.5** | 6 |
| BE113797 | Synaptogyrin 1 | Syngr1 | 0.00091 | 0.1 | -0.1 | -0.2 | **-0.7** | 6 |
| BE106583 | Synaptotagmin 2 | Syt2 | 0.00865 | **-0.5** | **0.3** | 0.2 | **-0.3** | 1 |

**Table S1** (continued).

| **GeneBank**  **Accession Number** | **Gene Name** | **Official**  **Symbol** | **ANOVA *P* Value** | **Log_2_ Intensity Ratio** | | | | **Cluster** |
| --- | --- | --- | --- | --- | --- | --- | --- | --- |
|  |  |  |  | **1h** | **2h** | **4h** | **6h** |  |
| BI304063 | Vacuolar protein sorting 4b | Vps4b | 0.00635 | -0.4 | -0.1 | -0.2 | **0.7** | 3 |
| **Translation/protein processing** | | | | | | | | |
| AA818636 | Beta galactoside alpha 2,6 sialyltransferase 1 | St6gal1 | 0.00858 | 0.3 | 0.2 | 0.2 | **1.5** | 4 |
| AA819336 | Cathepsin H | Ctsh | 0.00215 | 0.0 | -0.1 | -0.2 | **1.1** | 3 |
| AA859498 | Cathepsin L | Ctsl | 0.00146 | 0.3 | 0.4 | **1.1** | **1.4** | 4 |
| BF561675 | Eukaryotic translation elongation factor 1 epsilon 1 | Eef1e1 | 0.00120 | 0.1 | 0.2 | -0.1 | **1.1** | 3 |
| AA955540 | Eukaryotic translation initiation factor 1A | Eif1a | 0.00413 | 0.3 | **0.6** | 0.0 | **0.5** | 2 |
| BF556406 | Heterogeneous nuclear ribonucleoproteins methyltransferase-like 2 | Hrmt1l2 | 0.00825 | 0.2 | 0.0 | -0.2 | **1.1** | 3 |
| BF556843 | IBR domain containing 1 | Ibrdc1 | 0.00022 | -0.2 | 0.1 | -0.3 | **1.3** | 3 |
| BG380542 | Mahogunin, ring finger 1 | Mgrn1 | 0.00196 | -0.1 | -0.1 | 0.1 | **1.3** | 3 |
| BF415014 | Mitochondrial ribosomal protein L38 | mrpl38 | 0.00680 | -0.2 | -0.2 | 0.2 | **-1.3** | 6 |
| BF558495 | N6-DNA-methyltransferase isoform 1 | N6amt1 | 0.00582 | **0.7** | **1.4** | **0.5** | 0.1 | 2 |
| AA956005 | Poly(rC) binding protein 3 | Pcbp3 | 0.00647 | -0.1 | -0.1 | -0.1 | **1.1** | 3 |
| AI454578 | Polyadenylate-binding protein-interacting protein 2 | Paip2 | 0.00074 | 0.1 | -0.3 | -0.2 | **0.5** | 3 |
| BI300697 | Proteasome subunit, beta type 2 | Psmb2 | 0.00044 | -0.2 | 0.0 | -0.2 | **0.7** | 3 |
| BI284687 | Protein Tyr phosphatase | Ptp | 0.00305 | -0.1 | -0.3 | -0.1 | **0.9** | 3 |
| BI296229 | Proteosome subunit, beta type 9 | Psmb9 | 0.00608 | 0.1 | 0.1 | 0.3 | **-1.3** | 6 |
| AA874997 | Ribosomal protein S8 | Rps8 | 0.00449 | 0.5 | 0.2 | 0.1 | **0.7** | 4 |
| AW433845 | Ribosomal protein, large P2 | Rplp2 | 0.00834 | 0.2 | 0.0 | -0.1 | **0.4** | 3 |
| AA957307 | Seryl-aminoacyl-tRNA synthetase 1 | Sars1 | 0.00576 | 0.1 | 0.0 | 0.1 | **-0.6** | 6 |
| AA957708 | SUMO/sentrin specific protease 2 | Senp2 | 0.00519 | -0.1 | 0.0 | 0.0 | **0.7** | 3 |
| BE109655 | translation factor sui1 homolog | Sui1 | 0.00146 | 0.1 | 0.0 | 0.1 | **1.1** | 3 |
| BF567853 | Ubiquitin specific protease 33 | Usp33 | 0.00185 | 0.1 | -0.3 | -0.1 | **1.4** | 3 |
| **Amino acid metabolism** | | | | | | | | |
| AA818673 | Argininosuccinate lyase | Asl | 0.00386 | 0.6 | **0.7** | **0.8** | **1.2** | 4 |
| BI295687 | Gamma-glutamyltransferase-like 3 | Ggtl3 | 0.00283 | -0.1 | -0.1 | 0.0 | **1.1** | 3 |
| BF555631 | Glutamate oxaloacetate transaminase 1 | Got1 | 0.00196 | **0.7** | **1.3** | 0.5 | **1.8** | 4 |
| BF549503 | Leucine carboxyl methyltransferase 2 | Lcmt2 | 0.00126 | 0.2 | 0.2 | -0.1 | **-0.3** | 5 |
| BF555192 | Methionine adenosyltransferase I, alpha | Mat1a | 0.00376 | 0.7 | 0.2 | 0.0 | **1.3** | 4 |
| AA818680 | Ornithine aminotransferase | Oat | 0.00142 | 0.1 | 0.0 | -0.3 | **0.8** | 3 |
| BF418848 | Phosphoserine phosphatase | Psph | 0.00023 | -0.1 | 0.0 | -0.1 | **1.4** | 3 |
| CK845109 | Procollagen-lysine, 2-oxoglutarate 5-dioxygenase 3 | Plod3 | 0.00880 | -0.2 | -0.2 | 0.2 | **1.1** | 3 |
| BF556588 | Protoporphyrinogen oxidase | Ppox | 0.00166 | 0.0 | 0.1 | 0.1 | **-0.4** | 6 |
| BE121281 | Spermidine/spermine N1-acetyl transferase | Sat | 0.00018 | 0.0 | **1.0** | **1.6** | **1.6** | 4 |
| BE115543 | Sulfatase 1 | Sulf1 | 0.00508 | 0.0 | -0.1 | -0.4 | **1.6** | 3 |
| AA818350 | Tryptophan 2,3-dioxygenase | Tdo2 | 0.00016 | 0.3 | **0.6** | **0.3** | **0.6** | 4 |
| BF524147 | Tyrosinase (albino coat color) | Tyr | 0.00334 | -0.3 | 0.0 | -0.1 | **0.1** | 3 |
| **Nucleic acid metabolism** | | | | | | | | |
| AA964867 | Guanine deaminase | Gda | 0.00134 | **-0.5** | -0.2 | -0.3 | **1.3** | 3 |
| **Lipid metabolism** | | | | | | | | |
| BI277600 | Adiponutrin | Adpn | 0.00126 | 0.0 | 0.0 | -0.1 | **1.7** | 3 |

**Table S1** (continued).

| **GeneBank**  **Accession Number** | **Gene Name** | | **Official**  **Symbol** | **ANOVA *P* Value** | **Log_2_ Intensity Ratio** | | | | **Cluster** |
| --- | --- | --- | --- | --- | --- | --- | --- | --- | --- |
|  |  |  |  |  | **1h** | **2h** | **4h** | **6h** |  |
| BE108576 | Lipid phosphate phosphatase-related protein type 2 | | Lppr2 | 0.00393 | 0.0 | -0.1 | **-0.5** | **0.9** | 3 |
| BF554387 | Myotubularin related protein 9 | | Mtmr9 | 0.00393 | 0.1 | 0.0 | 0.1 | **-0.5** | 6 |
| BF553121 | Phosphatidylserine synthase 2 | | Ptdss2 | 0.00098 | 0.2 | -0.1 | -0.1 | **-0.5** | 5 |
| **Carbohydrate metabolism** | | | | | | | | | |
| BF559000 | Alpha glucosidase 2, alpha neutral subunit | | Ganab | 0.00303 | 0.7 | 0.0 | 0.2 | **1.0** | 4 |
| BF553404 | Phosphogluconate dehydrogenase | | Pgd | 0.00380 | 0.0 | -0.1 | 0.1 | **-0.4** | 6 |
| AA874837 | Phosphoglycerate mutase | | Pgam | 0.00480 | 0.1 | 0.2 | 0.1 | **1.3** | 3 |
| **Mitochondria metabolism** | | | | | | | | | |
| AI045119 | Dihydrolipoamide dehydrogenase | | Dld | 0.00221 | **0.4** | -0.2 | 0.1 | **-0.4** | 5 |
| AA875221 | Isocitrate dehydrogenase 3, gamma | | Idh3g | 0.00151 | 0.0 | 0.0 | -0.3 | **1.2** | 3 |
| BE108941 | Mitochondrial intermediate peptidase | | Mipep | 0.00519 | 0.0 | -0.1 | -0.2 | **1.4** | 3 |
| AI576526 | Thiamine triphosphatase | | Thtpa | 0.00800 | -0.1 | 0.0 | -0.6 | **0.7** | 3 |
| **Cofactor metabolism** | | | | | | | | | |
| BI276995 | | Phosphohistidine phosphatase 1 | Phpt1 | 0.00243 | 0.0 | 0.0 | 0.0 | **-0.6** | 6 |
| **Unknown function** | | | | | | | | | |
| BF555938 | 2810036L13Rik | | — | 0.00280 | -0.1 | -0.1 | 0.0 | **0.8** | 3 |
| BF566412 | B430201G11Rik | | — | 0.00236 | -0.1 | -0.1 | -0.3 | **1.2** | 3 |
| AI763611 | BC019806 | | — | 0.00349 | 0.0 | -0.1 | -0.2 | **0.7** | 3 |
| BF556937 | CG15118-PB | | — | 0.00063 | 0.0 | -0.1 | -0.3 | **1.2** | 3 |
| BI284785 | CG9646-PA | | — | 0.00530 | -0.1 | -0.1 | -0.1 | **1.2** | 3 |
| BF524632 | D930015E06 | | — | 0.00296 | 0.4 | -0.2 | -0.1 | **1.0** | 3 |
| CK845633 | LOC290635 | | — | 0.00052 | -0.2 | 0.1 | -0.5 | **1.4** | 3 |
| AA926035 | LOC305525 | | — | 0.00147 | 0.2 | 0.0 | 0.1 | **1.7** | 3 |
| AW522894 | LOC308556 | | — | 0.00242 | 0.1 | 0.0 | -0.3 | **1.6** | 3 |
| AI137525 | LOC308911 | | — | 0.00088 | -0.1 | 0.1 | **0.6** | **-1.3** | 6 |
| AI044607 | LOC360987 | | — | 0.00133 | 0.2 | 0.0 | 0.2 | **-0.7** | 6 |
| BF552836 | LOC362065 | | — | 0.00107 | -0.1 | 0.3 | 0.1 | **-1.1** | 6 |
| AI137544 | LOC362592 | | — | 0.00209 | 0.1 | -0.1 | 0.0 | **-0.8** | 6 |
| BF420607 | LOC364514 | | — | 0.00118 | 0.1 | 0.0 | -0.1 | **-0.4** | 5 |
| BF547761 | LOC364805 | | — | 0.00015 | -0.1 | 0.0 | -0.5 | **1.8** | 3 |
| BI290610 | LOC382585 | | — | 0.00318 | 0.0 | 0.0 | -0.4 | **1.0** | 3 |
| AA818617 | LOC497835 | | — | 0.00444 | 0.2 | 0.0 | -0.4 | **0.9** | 3 |
| AA955382 | LOC71177 | | — | 0.00800 | 0.1 | -0.2 | -0.1 | **0.6** | 3 |
| BE118161 | RGD1304622 | | — | 0.00310 | 0.0 | -0.1 | -0.3 | **1.9** | 3 |
| BF547600 | RGD1305440 | | — | 0.00464 | -0.1 | -0.2 | -0.2 | **0.9** | 3 |
| AA874936 | RGD1305809 | | — | 0.00862 | -0.1 | 0.1 | -0.1 | **-0.4** | 6 |
| CK843842 | RGD1306500 | | — | 0.00249 | -0.3 | 0.0 | -0.1 | **-0.5** | 1 |
| BI282882 | RGD1306660 | | — | 0.00132 | 0.1 | -0.1 | -0.1 | **0.6** | 3 |
| BF406560 | RGD1309651 | | — | 0.00753 | -0.3 | 0.1 | -0.1 | **0.6** | 3 |
| AA955989 | RGD1309735 | | — | 0.00299 | -0.1 | 0.0 | 0.0 | **1.0** | 3 |
| BF557643 | RGD1310320 | | — | 0.00573 | **0.4** | -0.1 | 0.1 | **-0.6** | 5 |
| AI555302 | RGD1310480 | | — | 0.00685 | **0.5** | 0.0 | -0.2 | **-0.6** | 5 |

**Table S1** (continued).

| **GeneBank**  **Accession Number** | **Gene Name** | **Official**  **Symbol** | **ANOVA *P* Value** | **Log_2_ Intensity Ratio** | | | | **Cluster** |
| --- | --- | --- | --- | --- | --- | --- | --- | --- |
|  |  |  |  | **1h** | **2h** | **4h** | **6h** |  |
| BI294925 | RGD1310609 | — | 0.00223 | -0.2 | 0.0 | -0.3 | **2.2** | 3 |
| BF556143 | RGD1310953 | — | 0.00366 | 0.1 | -0.2 | -0.3 | **0.6** | 3 |
| CK840913 | RGD1311634 | — | 0.00567 | 0.2 | -0.2 | -0.3 | **1.6** | 3 |
| AA997115 | RGD1311859 | — | 0.00756 | 0.3 | -0.2 | **-0.4** | **-0.3** | 5 |
| BG373502 | RGD1311899 | — | 0.00730 | -0.2 | -0.1 | **-0.3** | **-0.1** | 1 |
| AI045518 | RGD1561029 | — | 0.00810 | 0.3 | 0.0 | 0.1 | **-0.7** | 5 |
| CK840551 | RGD1561311 | — | 0.00598 | -0.1 | -0.1 | 0.2 | **0.5** | 3 |
| BI277158 | RGD1562200 | — | 0.00733 | -0.1 | -0.3 | 0.3 | **-0.8** | 6 |
| BG374630 | RGD1562884 | — | 0.00215 | 0.0 | -0.3 | 0.0 | **0.7** | 3 |
| AA963656 | RGD1563633 | — | 0.00144 | 0.3 | **1.0** | **0.6** | **0.9** | 2 |
| BI289288 | RGD1564778 | — | 0.00067 | -0.2 | 0.1 | -0.1 | **0.6** | 3 |
| AI059837 | RGD735112 | — | 0.00650 | 0.2 | 0.0 | 0.0 | **-0.3** | 5 |
| AI575918 | Transcribed locus | — | 0.00197 | 0.0 | **-0.3** | -0.3 | **0.6** | 3 |
| AI029979 | Transcribed locus | — | 0.00229 | 0.3 | 0.1 | -0.2 | **1.5** | 3 |
| CK838606 | Transcribed locus | — | 0.00173 | 0.5 | 0.2 | **0.7** | **1.1** | 4 |
