# Supplementary material for: Temporal and functional profile of the transcriptional regulatory network in the early regenerative response to partial hepatectomy in the rat
Source: BMC Genomics. 2008 Nov 6;9:527. doi: 10.1186/1471-2164-9-527 (PMC2613928; doi:10.1186/1471-2164-9-527)
Supplement: Additional file 2 — Table S2. Sham surgery associated changes in gene expression revealed by cDNA microarray analysis. [file 1471-2164-9-527-S2.doc]

**Table S2**. Changes in gene expression in sham-operated animals (1h) revealed by cDNA microarray analysis.

| **GeneBank**  **Accession Number** | **Gene Name** | **Official Symbol** | **ANOVA *P* Value** | **Log_2_ Intensity Ratio** |
| --- | --- | --- | --- | --- |
| BF394132 | CG16812-PA | RGD1307554 | 0.006194 | -0.76914 |
| BI288673 | Leucine zipper, putative tumor suppressor 2 | Lzts2 | 0.004896 | -0.68418 |
| BI299340 | COMM domain containing 10 | Commd10 | 0.002155 | -0.54571 |
| BG377481 | Pregnancy specific beta-1-glycoprotein 4 | Psg4 | 0.009079 | -0.54256 |
| BF555139 | Granulin | Grn | 0.002943 | -0.49841 |
| BG373037 | Glutamic pyruvic transaminase 1, soluble | Gpt1 | 0.001097 | -0.49783 |
| AI454106 | GIY-YIG domain containing 2 | Giyd2 | 0.007017 | -0.44814 |
| BI276707 | GTP binding protein 6 | Gtpbp6 | 0.005481 | -0.43146 |
| BI290169 | Serine (or cysteine) peptidase inhibitor, clade A (alpha-1 antiproteinase, antitrypsin), member 10 | Serpina10 | 0.002431 | -0.39805 |
| BI302711 | Vacuolar protein sorting 35 | Vps35 | 0.008198 | -0.38866 |
| BF542263 | N-myristoyltransferase 1 | Nmt1 | 0.00963 | 0.381072 |
| AI136569 | Phosphatase and tensin homolog | Pten | 0.00356 | 0.390728 |
| BF555222 | Poliovirus receptor-related 2 (herpesvirus entry mediator B) | Pvrl2 | 0.006252 | 0.42052 |
| BF557229 | Calcium channel, voltage-dependent, beta 2 subunit | Cacnb2 | 0.002618 | 0.453968 |
| CK845092 | PHD finger protein 5A | Phf5a | 0.004942 | 0.711643 |
| AI712625 | RIKEN cDNA 0610011N22 gene | RGD735106 | 0.004306 | 0.819867 |
