# Supplementary material for: Temporal and functional profile of the transcriptional regulatory network in the early regenerative response to partial hepatectomy in the rat
Source: BMC Genomics. 2008 Nov 6;9:527. doi: 10.1186/1471-2164-9-527 (PMC2613928; doi:10.1186/1471-2164-9-527)
Supplement: Additional file 4 — Supplemental text. Analysis of functional gene categories changing after partial hepatectomy. [file 1471-2164-9-527-S4.doc]

# Analysis of functional gene categories changing after partial hepatectomy

Several major functional categories could be assigned to the 267 annotated differentially expressed genes using Gene Ontology [1] ([http://www.geneontology.org](http://www.geneontology.org/)). Below is a detailed discussion of the individual genes identified in these categories.

***Transcription Factors.*** We identified 41 transcription-related genes that are differentially regulated during the priming phase [see Additional file 1]. These genes include transcription factors, co-activators and co-repressors of transcription, many of which overlap with previously reported immediate-early genes [2-7]. We observed rapid up-regulation of such widely reported immediate-early transcription factors as JunB, Myc, C/EBPβ, and increased expression level of STAT3 at later time-points. We also observed changes in expression level of several Kruppel-like factors. Core promoter element binding protein (Klf6) was up-regulated at 2-6 h after PHx. Klf6 is a zinc-finger protein broadly involved in differentiation and development, growth-related signal transduction, cell proliferation, apoptosis, and angiogenesis [8]. Although the specific function of Klf6 in liver regeneration is unknown, this factor is involved in regulation of expression levels of several genes previously implicated in hepatocyte priming, such as p21 and IGF-I receptor (IGF1R) [9, 10]. The up-regulation of p21 was previously reported 4 h after PHx [6], although we did not detect a significant increase in our microarray samples. The expression profile of IGF1R after PHx is not known, but liver regeneration is impaired in mice with liver-specific IGF1R knockout [11]. Basic transcription element binding protein 1 (Kfl9) was up-regulated at the 6 h time-point. This factor is known to interact with members of the JNK family to mediate the expression of growth-associated genes, such as p21 and IGFBP-2 [12]. Basic Kruppel-like factor (Klf3) acts to repress transcription [13].

Activating transcription factor 4 (ATF4) was induced starting at 2 h after PHx. ATF4 reportedly directly interacts with Jun proteins to mediate transcription regulatory specificity [14]. ATF4 was reported to be involved in up-regulation of insulin-like growth factor binding protein-1 (IGFBP-1) in hepatic endoplasmic reticulum (ER) stress conditions [15]. In our samples, the expression of IGFBP-1, which is known to be up-regulated during the initial phase of liver regeneration, peaked at 2 h and returned to basal level by 6 h after PHx. Hence, the transcription profile we observed suggests that increased expression of ATF4 is not required for upregulation of IGFBP-1 in the initial phase of liver regeneration. Expression of activating transcription factor 6 (ATF6) decreased by the 6 h time-point. ATF6 also is an ER stress-regulated transcription factor that activates the transcription of ER stress proteins. Specific ER stress signaling transmitted by ATF6 promotes apoptosis in developmental processes [16]. ATF6 also plays a part in maintaining homeostasis of lipids and glucose [17].

***Signal Transduction.*** We observed differential regulation of multiple genes related to signal transduction. Notably, expression of several genes of the mitogen-activated protein kinase (MAPK) cascades and related pathways occurs at 6 hours after PHx. MAPKs act as an integration point for multiple biochemical signals, and are involved in proliferation, differentiation, transcription regulation and development [18]. The expression level of MAPK1, also known as extracellular signal-regulated kinase 2 (ERK2) goes up at the 6 h time-point. MAPK kinase 5 (Map2k5) is a dual specificity protein kinase that belongs to the MAPK kinase (MKK) family. This kinase specifically interacts with and activates MAPK7 and thereby affects cell proliferation [19]. The signal cascade mediated by this kinase is involved in growth factor stimulated cell proliferation and cell differentiation. Map2k5 may be one of the Stat3-regulated genes [20].

Dups6, also known as MAP kinase phosphatase 3 (MKP3) is up-regulated at 6h after PHx. The protein encoded by this gene is a member of the dual specificity protein phosphatase subfamily that dephosphorylates both the phosphoserine/threonine and phosphotyrosine residues on their target MAPK proteins. Different members of the family of dual specificity phosphatases show distinct substrate specificities for various MAP kinases. DUPS6 protein dephosphorylates the active form of MAPK1, generating a feedback loop to control MAPK1 activity [21].

We observed up-regulation of Ppp2ca at the 6 h time-point. This gene encodes the protein phosphatase 2A (PP2A) catalytic subunit. PP2A consists of a common heteromeric core enzyme, which is composed of a catalytic subunit and a variety of regulatory subunits. PP2A is one of the four major classes of Ser/Thr phosphatases, and is implicated in the negative control of a wide variety of signaling pathways with function in metabolic regulation, cell growth and division [22]. PP2A may also contribute to the regulation of MAPK signaling pathway by inactivating MAP kinases [23].

***Cell Proliferation and Cell Cycle Related Genes.*** Consistent with the expression of both pro- and antiproliferative transcription factors, we observe the differential regulation of genes that stimulate and inhibit cell-cycle entry starting at 1 h after PHx. Overall, we detected the differential regulation of 19 cell proliferation related genes during the time course of hepatocyte priming. Several of these genes are checkpoint genes at major cell-cycle transitions that can act to inhibit the cell cycle.

Pre-B cell colony-enhancing factor (Pbef1) is a secreted cytokine-like protein associated with cell cycle regulation [24] and is regulated via IL-6 trans-signaling [25]. The same protein has also been identified as an insulin-mimetic hormone visfatin, which binds to and activates the insulin receptor and affects glucose tolerance [26]. More recently, intracellular functions of PBEF1 were identified. The enzyme mediates the rate-limiting step in NAD biosynthesis from nicotinamide, is required for vascular smooth muscle cell maturation, and is associated with activation of SIRT1 and regulation of longevity [26, 27].

B-cell translocation gene 1 (Btg1) and B-cell translocation gene 2 (Btg2), members of the anti-proliferative gene family that regulates cell growth and differentiation, were rapidly induced after PHx. The levels of Btg2 mRNA, a p53-dependent growth arrest gene that inhibits the G_1_/S transition [28], reached maximum levels by 2 hours after PHx. The induction of this gene is reported as early as 10 minutes after PHx [6]. Btg2 promotes cell differentiation and is required for survival of terminally differentiated cells [29]. Induction of Btg1 was somewhat slower; its expression was up-regulated starting at 2 h and increasing until 6 h after PHx. A negative correlation between Btg1 mRNA expression and cell proliferation is observed both in vitro and in vivo [30, 31]. Btg1 is also involved in cell differentiation and organogenesis [32, 33]. Btg1 is strongly expressed in the G_0_/G_1_ phases of the cell cycle, and then down-regulated during the later phases [31]. Both Btg1 and Btg2 may participate in transcription regulation [34]. They act as coactivators for nuclear receptors and myogenic factors [35].

*Stress and inflammatory response.* Inflammatory responses have been implicated in the priming of liver and other types of tissue repair [36, 37]. We do not see induction of such liver regeneration associated cytokines as IL-6, IL-2, IL-3, IL-4, IL-5, TNFα, TGFβ in our samples, indicating that if induction of these genes occurred, it was below our detection limit. For instance, activation of cytokine synthesis in non-parenchymal cells in the liver probably would not be detected owing to the fact that parenchymal cells are the predominant cell type represented in liver tissue (approximately 70% of liver cell number and 90% of liver cell mass). However, expression of several pro- and anti-inflammatory proteins was increased in our experiments. In accordance with recent studies [38], we observed induction of several complement-related genes at 6 h after PHx, such as complement component 1, q subcomponent binding protein (C1QBP) and complement component 5, receptor 1 (C5r1). Kininogen (KNG) was up-regulated at 2-6 hours after PHx. KNG is pro-inflammatory protein that induces production of IL-6 and IL-8 [39]. Zinc finger protein 36 (Zfp36), also known as tristetraprolin (TTP), was rapidly induced after PHx. Initially discovered as a gene that could be induced rapidly by the stimulation of fibroblasts with growth factors and mitogens, it is now known that TTP can bind to AU-rich elements in mRNA, leading to the removal of the poly(A) tail from that mRNA and increased rates of mRNA turnover [40]. By binding to and destabilizing mRNAs, TTP down-regulates expression of such pro-inflammatory genes as IL-2 and TNFα [41]. Expression levels of TTP increase in response to tissue damage. In damaged muscle TTP is induced 30 minutes after injury [42]. Production of pro-inflammatory cytokines is up-regulated after PHx and TTP may play a part in controlling their expression levels.

Induction of several oxidative stress response genes was observed in our experiments. For example, superoxide dismutase 2 (SOD2) was rapidly up-regulated after PHx. The expression level of SOD2 is regulated by NFκB [43]. The expression level of flavin containing monooxygenase 2 (Fmo2) also increased after PHx. In addition, we observed rapid up-regulation of metallothionein isoform Mt1a after partial hepatectomy followed by a slower response of Mt3. Several studies demonstrated defective liver regeneration after injury in Mt-knockout mice [44, 45].

# References

1 Ashburner M, Ball CA, Blake JA, Botstein D, Butler H, Cherry JM, Davis AP, Dolinski K, Dwight SS, Eppig JT, Harris MA, Hill DP, Issel-Tarver L, Kasarskis A, Lewis S, Matese JC, Richardson JE, Ringwald M, Rubin GM, Sherlock G: **Gene ontology: tool for the unification of biology. The Gene Ontology Consortium.** *Nat Genet* 2000, **25**:25-29.

2 Cressman DE, Greenbaum LE, DeAngelis RA, Ciliberto G, Furth EE, Poli V, Taub R: **Liver failure and defective hepatocyte regeneration in interleukin-6-deficient mice.** *Science* 1996, **274**:1379-1383.

3 Li W, Liang X, Kellendonk C, Poli V, Taub R: **STAT3 contributes to the mitogenic response of hepatocytes during liver regeneration.** *J Biol Chem* 2002, **277**:28411-28417.

4 Leu JI, Crissey MA, Leu JP, Ciliberto G, Taub R: **Interleukin-6-induced STAT3 and AP-1 amplify hepatocyte nuclear factor 1-mediated transactivation of hepatic genes, an adaptive response to liver injury.** *Mol Cell Biol* 2001, **21**:414-424.

5 Otu HH, Naxerova K, Ho K, Can H, Nesbitt N, Libermann TA, Karp SJ: **Restoration of liver mass after injury requires proliferative and not embryonic transcriptional patterns.** *J Biol Chem* 2007, **282**:11197-11204.

6 Su AI, Guidotti LG, Pezacki JP, Chisari FV, Schultz PG: **Gene expression during the priming phase of liver regeneration after partial hepatectomy in mice.** *Proc Natl Acad Sci USA* 2002, **99**:11181-11186.

7 Taub R, Greenbaum LE, Peng Y: **Transcriptional regulatory signals define cytokine-dependent and -independent pathways in liver regeneration.** *Semin Liver Dis* 1999, **19**:117-127.

8 Cho YG, Kim CJ, Park CH, Yang YM, Kim SY, Nam SW, Lee SH, Yoo NJ, Lee JY, Park WS: **Genetic alterations of the KLF6 gene in gastric cancer.** *Oncogene* 2005, **24**:4588-4590.

9 Narla G, Kremer-Tal S, Matsumoto N, Zhao X, Yao S, Kelley K, Tarocchi M, Friedman SL: **In vivo regulation of p21 by the Kruppel-like factor 6 tumor-suppressor gene in mouse liver and human hepatocellular carcinoma.** *Oncogene* 2007, **26**:4428-4434.

10 Rubinstein M, Idelman G, Plymate SR, Narla G, Friedman SL, Werner H: **Transcriptional activation of the insulin-like growth factor I receptor gene by the Kruppel-like factor 6 (KLF6) tumor suppressor protein: potential interactions between KLF6 and p53.** *Endocrinology* 2004, **145**:3769-3777.

11 Desbois-Mouthon C, Wendum D, Cadoret A, Rey C, Leneuve P, Blaise A, Housset C, Tronche F, Le Bouc Y, Holzenberger M: **Hepatocyte proliferation during liver regeneration is impaired in mice with liver-specific IGF-1R knockout.** *FASEB J* 2006, **20**:773-775.

12 Simmen RC, Zhang XL, Michel FJ, Min SH, Zhao G, Simmen FA: **Molecular markers of endometrial epithelial cell mitogenesis mediated by the Sp/Kruppel-like factor BTEB1.** *DNA Cell Biol* 2002, **21**:115-128.

13 Turner J, Crossley M: **Basic Kruppel-like factor functions within a network of interacting haematopoietic transcription factors.** *Int J Biochem Cell Biol* 1999, **31**:1169-1174.

14 Horisawa K, Tateyama S, Ishizaka M, Matsumura N, Takashima H, Miyamoto-Sato E, Doi N, Yanagawa H: **In vitro selection of Jun-associated proteins using mRNA display.** *Nucleic Acids Res* 2004, **32**:e169.

15 Marchand A, Tomkiewicz C, Magne L, Barouki R, Garlatti M: **Endoplasmic reticulum stress induction of insulin-like growth factor-binding protein-1 involves ATF4.** *J Biol Chem* 2006, **281**:19124-19133.

16 Nakanishi K, Sudo T, Morishima N: **Endoplasmic reticulum stress signaling transmitted by ATF6 mediates apoptosis during muscle development.** *J Cell Biol* 2005, **169**:555-560.

17 Zeng L, Lu M, Mori K, Luo S, Lee AS, Zhu Y, Shyy JY: **ATF6 modulates SREBP2-mediated lipogenesis.** *EMBO J* 2004, **23**:950-958.

18 Gerits N, Kostenko S, Moens U: **In vivo functions of mitogen-activated protein kinases: conclusions from knock-in and knock-out mice.** *Transgenic Res* 2007, **16**:281-314.

19 Cameron SJ, Abe J, Malik S, Che W, Yang J: **Differential role of MEK5alpha and MEK5beta in BMK1/ERK5 activation.** *J Biol Chem* 2004, **279**:1506-1512.

20 Song H, Jin X, Lin J: **Stat3 upregulates MEK5 expression in human breast cancer cells.** *Oncogene* 2004, **23**:8301-8309.

21 Karlsson M, Mathers J, Dickinson RJ, Mandl M, Keyse SM: **Both nuclear-cytoplasmic shuttling of the dual specificity phosphatase MKP-3 and its ability to anchor MAP kinase in the cytoplasm are mediated by a conserved nuclear export signal.** *J Biol Chem* 2004, **279**:41882-41891.

22 Ofek P, Ben-Meir D, Kariv-Inbal Z, Oren M, Lavi S: **Cell cycle regulation and p53 activation by protein phosphatase 2C alpha.** *J Biol Chem* 2003, **278**:14299-14305.

23 Avdi NJ, Malcolm KC, Nick JA, Worthen GS: **A role for protein phosphatase-2A in p38 mitogen-activated protein kinase-mediated regulation of the c-Jun NH(2)-terminal kinase pathway in human neutrophils.** *J Biol Chem* 2002, **277**:40687-40696.

24 Kitani T, Okuno S, Fujisawa H: **Growth phase-dependent changes in the subcellular localization of pre-B-cell colony-enhancing factor.** *FEBS Lett* 2003, **544**:74-78.

25 Nowell MA, Richards PJ, Fielding CA, Ognjanovic S, Topley N, Williams AS, Bryant-Greenwood G, Jones SA: **Regulation of pre-B cell colony-enhancing factor by STAT-3-dependent interleukin-6 trans-signaling: implications in the pathogenesis of rheumatoid arthritis.** *Arthritis Rheum* 2006, **54**:2084-2095.

26 Revollo JR, Grimm AA, Imai S: **The regulation of nicotinamide adenine dinucleotide biosynthesis by Nampt/PBEF/visfatin in mammals.** *Curr Opin Gastroenterol* 2007, **23**:164-170.

27 Pilz S, Mangge H, Obermayer-Pietsch B, Marz W: **Visfatin/pre-B-cell colony-enhancing factor: a protein with various suggested functions.** *J Endocrinol Invest* 2007, **30**:138-144.

28 Guardavaccaro D, Corrente G, Covone F, Micheli L, D'Agnano I, Starace G, Caruso M, Tirone F: **Arrest of G(1)-S progression by the p53-inducible gene PC3 is Rb dependent and relies on the inhibition of cyclin D1 transcription.** *Mol Cell Biol* 2000, **20**:1797-1815.

29 Morel AP, Sentis S, Bianchin C, Le Romancer M, Jonard L, Rostan MC, Rimokh R, Corbo L: **BTG2 antiproliferative protein interacts with the human CCR4 complex existing in vivo in three cell-cycle-regulated forms.** *J Cell Sci* 2003, **116**:2929-2936.

30 Corjay MH, Kearney MA, Munzer DA, Diamond SM, Stoltenborg JK: **Antiproliferative gene BTG1 is highly expressed in apoptotic cells in macrophage-rich areas of advanced lesions in Watanabe heritable hyperlipidemic rabbit and human.** *Lab Invest* 1998, **78**:847-858.

31 Rouault JP, Rimokh R, Tessa C, Paranhos G, Ffrench M, Duret L, Garoccio M, Germain D, Samarut J, Magaud JP: **BTG1, a member of a new family of antiproliferative genes.** *EMBO J* 1992, **11**:1663-1670.

32 Iwai K, Hirata K, Ishida T, Takeuchi S, Hirase T, Rikitake Y, Kojima Y, Inoue N, Kawashima S, Yokoyama M: **An anti-proliferative gene BTG1 regulates angiogenesis in vitro.** *Biochem Biophys Res Commun* 2004, **316**:628-635.

33 Sakaguchi T, Kuroiwa A, Takeda H: **Expression of zebrafish btg-b, an anti-proliferative cofactor, during early embryogenesis.** *Mech Dev* 2001, **104**:113-115.

34 Prevot D, Morel AP, Voeltzel T, Rostan MC, Rimokh R, Magaud JP, Corbo L: **Relationships of the antiproliferative proteins BTG1 and BTG2 with CAF1, the human homolog of a component of the yeast CCR4 transcriptional complex: involvement in estrogen receptor alpha signaling pathway.** *J Biol Chem* 2001, **276**:9640-9648.

35 Busson M, Carazo A, Seyer P, Grandemange S, Casas F, Pessemesse L, Rouault JP, Wrutniak-Cabello C, Cabello G: **Coactivation of nuclear receptors and myogenic factors induces the major BTG1 influence on muscle differentiation.** *Oncogene* 2005, **24**:1698-1710.

36 Li W, Liang X, Leu JI, Kovalovich K, Ciliberto G, Taub R: **Global changes in interleukin-6-dependent gene expression patterns in mouse livers after partial hepatectomy.** *Hepatology* 2001, **33**:1377-1386.

37 Piehl F, Lidman O: **Neuroinflammation in the rat--CNS cells and their role in the regulation of immune reactions.** *Immunol Rev* 2001, **184**:212-225.

38 Strey CW, Markiewski M, Mastellos D, Tudoran R, Spruce LA, Greenbaum LE, Lambris JD: **The proinflammatory mediators C3a and C5a are essential for liver regeneration.** *J Exp Med* 2003, **198**:913-923.

39 Zhu YM, Bradbury DA, Pang L, Knox AJ: **Transcriptional regulation of interleukin (IL)-8 by bradykinin in human airway smooth muscle cells involves prostanoid-dependent activation of AP-1 and nuclear factor (NF)-IL-6 and prostanoid-independent activation of NF-kappaB.** *J Biol Chem* 2003, **278**:29366-29375.

40 Carrick DM, Lai WS, Blackshear PJ: **The tandem CCCH zinc finger protein tristetraprolin and its relevance to cytokine mRNA turnover and arthritis.** *Arthritis Res Ther* 2004, **6**:248-264.

41 Ogilvie RL, Abelson M, Hau HH, Vlasova I, Blackshear PJ, Bohjanen PR: **Tristetraprolin down-regulates IL-2 gene expression through AU-rich element-mediated mRNA decay.** *J Immunol* 2005, **174**:953-961.

42 Cao H, Kelly MA, Kari F, Dawson HD, Urban JF,Jr, Coves S, Roussel AM, Anderson RA: **Green tea increases anti-inflammatory tristetraprolin and decreases pro-inflammatory tumor necrosis factor mRNA levels in rats.** *J Inflamm* 2007, **4**:1.

43 Murley JS, Kataoka Y, Weydert CJ, Oberley LW, Grdina DJ: **Delayed radioprotection by nuclear transcription factor kappaB -mediated induction of manganese superoxide dismutase in human microvascular endothelial cells after exposure to the free radical scavenger WR1065.** *Free Radic Biol Med* 2006, **40**:1004-1016.

44 Oliver JR, Jiang S, Cherian MG: **Augmented hepatic injury followed by impaired regeneration in metallothionein-I/II knockout mice after treatment with thioacetamide.** *Toxicol Appl Pharmacol* 2006, **210**:190-199.

45 Oliver JR, Mara TW, Cherian MG: **Impaired hepatic regeneration in metallothionein-I/II knockout mice after partial hepatectomy.** *Exp Biol Med* 2005, **230**:61-67.
