# Supplementary material for: Temporal and functional profile of the transcriptional regulatory network in the early regenerative response to partial hepatectomy in the rat
Source: BMC Genomics. 2008 Nov 6;9:527. doi: 10.1186/1471-2164-9-527 (PMC2613928; doi:10.1186/1471-2164-9-527)
Supplement: Additional file 5 — Table S4. Distribution of TF binding sites in the functional differentially expressed gene categories. [file 1471-2164-9-527-S5.doc]

**Table S4**. Distribution of TF binding sites in the functional differentially expressed gene categories. Number of genes in each functional category that have one or more binding sites for TFs enriched in specific gene expression clusters is shown.

| **Transcription factor** | **Metabolism** | **Cell structure/**  **cytoskeleton** | **Cell cycle/**  **proliferation** | **Cell death** | **Cell differentiation** | **Transcription** | **Signaling** | **Stress/immune response** | **Transport/**  **trafficking** | **Translation/**  **protein processing** |
| --- | --- | --- | --- | --- | --- | --- | --- | --- | --- | --- |
| **Cluster 1** | | | | | | | | | | |
| MyoD | 0 | 0 | 2 | 0 | 0 | 0 | 0 | 0 | 0 | 0 |
| CREB | 0 | 0 | 2 | 0 | 0 | 0 | 0 | 0 | 0 | 0 |
| MYB | 0 | 0 | 2 | 0 | 0 | 0 | 0 | 0 | 0 | 0 |
| **Cluster 2** | | | | | | | | | | |
| BRN-2 | 0 | 1 | 1 | 4 | 0 | 1 | 0 | 1 | 0 | 0 |
| NRF-2 | 0 | 0 | 0 | 2 | 0 | 2 | 0 | 1 | 1 | 0 |
| PAX6 | 0 | 1 | 1 | 2 | 0 | 2 | 0 | 1 | 1 | 1 |
| **Cluster3** | | | | | | | | | | |
| AP-2α | 2 | 3 | 0 | 0 | 1 | 5 | 3 | 2 | 3 | 1 |
| ATF | 1 | 1 | 1 | 0 | 0 | 2 | 2 | 2 | 1 | 2 |
| CDP | 1 | 3 | 4 | 1 | 0 | 5 | 5 | 6 | 5 | 2 |
| CREB | 1 | 3 | 3 | 0 | 0 | 0 | 3 | 2 | 1 | 2 |
| IPF-1 | 0 | 1 | 1 | 0 | 2 | 3 | 3 | 2 | 1 | 3 |
| PEBP | 1 | 1 | 0 | 0 | 1 | 4 | 1 | 2 | 4 | 2 |
| TTF1 | 2 | 5 | 2 | 0 | 2 | 11 | 2 | 4 | 3 | 3 |
| MYB | 1 | 1 | 0 | 0 | 0 | 5 | 2 | 3 | 1 | 0 |
| **Cluster 4** | | | | | | | | | | |
| GATA | 0 | 1 | 0 | 0 | 0 | 3 | 0 | 2 | 0 | 0 |
| HNF-1 | 4 | 3 | 3 | 0 | 0 | 2 | 1 | 5 | 0 | 1 |
| MRF-2 | 3 | 3 | 3 | 0 | 0 | 3 | 1 | 1 | 1 | 0 |
| NFκB | 3 | 5 | 3 | 1 | 0 | 4 | 1 | 5 | 1 | 2 |
| SRF | 0 | 1 | 2 | 0 | 0 | 2 | 1 | 0 | 0 | 1 |
| **Cluster 5** | | | | | | | | | | |
| C/EBP | 2 | 0 | 1 | 1 | 0 | 1 | 1 | 1 | 1 | 0 |
| GATA | 1 | 1 | 0 | 0 | 0 | 0 | 1 | 1 | 0 | 0 |
| LEF-1/HNF-1 | 2 | 0 | 0 | 0 | 2 | 1 | 1 | 0 | 1 | 0 |
| MYB | 0 | 0 | 0 | 1 | 0 | 1 | 0 | 1 | 0 | 0 |
| **Cluster 6** | | | | | | | | | | |
| CP-2 | 1 | 1 | 0 | 1 | 0 | 3 | 4 | 1 | 1 | 1 |
| CREB | 0 | 1 | 0 | 0 | 0 | 1 | 0 | 0 | 1 | 1 |
| IPF-1 | 0 | 0 | 0 | 1 | 0 | 0 | 1 | 0 | 0 | 2 |
| Poly A | 2 | 1 | 0 | 2 | 0 | 5 | 2 | 2 | 3 | 2 |
| Myogenin / NF-1 | 1 | 2 | 0 | 1 | 1 | 2 | 1 | 1 | 2 | 0 |
