# Supplementary material for: Temporal and functional profile of the transcriptional regulatory network in the early regenerative response to partial hepatectomy in the rat
Source: BMC Genomics. 2008 Nov 6;9:527. doi: 10.1186/1471-2164-9-527 (PMC2613928; doi:10.1186/1471-2164-9-527)
Supplement: Additional file 6 — Table S5. Clones spotted on the array. [file 1471-2164-9-527-S6.doc]

**Table S5.** Clones spotted on the array.

| **#** | **UniGene Clone ID** | **Gene Name** | **GeneBank Accession Number** |
| --- | --- | --- | --- |
| 1 | UI-R-A1-do-e-09-0-UI | Matrilin 1, cartilage matrix protein | BF558617 |
| 2 | UI-R-AF0-yf-e-06-0-UI | Spindle pole body component 25 homolog | CK840570 |
| 3 | UI-R-BJ0p-aio-f-02-0-UI | RIKEN cDNA 2810421I24 | AW525166 |
| 4 | UI-R-CA0-axa-c-11-0-UI | Hypothetical protein LOC689486 | BE108092 |
| 5 | UI-R-E0-bo-b-12-0-UI | Transcribed locus | AA858492 |
| 6 | UI-R-E0-cm-f-05-0-UI | UbiA prenyltransferase domain containing 1 | BF554993 |
| 7 | UI-R-A1-ds-b-12-0-UI | Prostaglandin E synthase 2 | BF558739 |
| 8 | UI-R-A1-el-g-04-0-UI | Retinoblastoma-binding protein 2 (RBBP-2) | AA926315 |
| 9 | UI-R-Y0-lz-g-07-0-UI | Down syndrome cell adhesion molecule-like 1 | AI111488 |
| 10 | UI-R-C2p-nw-c-07-0-UI | spinocerebellar ataxia 7 homolog | BF553592 |
| 11 | UI-R-C0-hb-c-12-0-UI | XP_001106579.1 muscleblind-like 1 | AA964972 |
| 12 | UI-R-C0-hg-e-03-0-UI | SAP30 binding protein | AA996837 |
| 13 | UI-R-C0-jd-e-01-0-UI | microfilament and actin filament cross-linker protein isoform b | AI030741 |
| 14 | UI-R-C0-jm-h-09-0-UI | Angio-associated migratory protein | AI043785 |
| 15 | UI-R-C0-jr-g-08-0-UI | transmembrane protein TM9SF3 | BF545305 |
| 16 | UI-R-C1-jv-d-12-0-UI | Phosphomannomutase 1 | AI043619 |
| 17 | UI-R-A0-aw-f-01-0-UI | Splicing factor proline/glutamine rich (polypyrimidine tract binding protein associated) | BF549693 |
| 18 | UI-R-C0-hc-f-02-0-UI | Complement component 4a | BF560017 |
| 19 | UI-R-A0-ae-a-05-0-UI | Transmembrane protein 109 | AA817788 |
| 20 | UI-R-A0-ax-g-01-0-UI | RIKEN cDNA 2700038C09 | BF549713 |
| 21 | UI-R-E1-fo-e-12-0-UI | Mitofusin 2 | AA956740 |
| 22 | UI-R-Y0-lv-f-09-0-UI | Upstream of NRAS | AI070521 |
| 23 | UI-R-A1-es-c-08-0-UI | TAF9 RNA polymerase II, TATA box binding protein (TBP)-associated factor | AA926009 |
| 24 | UI-R-E1-fy-a-12-0-UI | Budding uninhibited by benzimidazoles 1 homolog, beta | BF557145 |
| 25 | UI-R-C2-mx-h-09-0-UI | Electron-transferring-flavoprotein dehydrogenase | AI070723 |
| 26 | UI-R-BT0-qh-b-06-0-UI | Ndr3 protein | AI146115 |
| 27 | UI-R-A0-ax-a-04-0-UI | Transmembrane protein 50A | AA818246 |
| 28 | UI-R-A0-ba-d-11-0-UI | Protein tyrosine phosphatase, non-receptor type 22 (lymphoid) | BF555199 |
| 29 | UI-R-C0-gs-b-08-0-UI | Signal peptide, CUB domain, EGF-like 1 | AA963813 |
| 30 | UI-R-C0-gv-g-10-0-UI | AarF domain containing kinase 4 | BF557780 |
| 31 | UI-R-E0-cc-e-05-0-UI | Villin 2 | AA859812 |
| 32 | UI-R-E0-ci-c-07-0-UI | Protein kinase inhibitor beta, cAMP dependent, catalytic | AA874865 |
| 33 | UI-R-E1-fd-a-07-0-UI | XP_001087239.1 neurolysin isoform 3 | AA955894 |
| 34 | UI-R-E1-fo-g-10-0-UI | Adaptor-related protein complex 2, sigma 1 subunit | AA956749 |
| 35 | UI-R-E1-fz-g-09-0-UI | Secreted acidic cysteine rich glycoprotein | AA957962 |
| 36 | UI-R-C0-hz-g-12-0-UI | Neurochondrin | AA998302 |
| 37 | UI-R-A1-et-a-02-0-UI | Hydroxyprostaglandin dehydrogenase 15 (NAD) | BF558398 |
| 38 | UI-R-E1-gj-a-04-0-UI | XP_001082958.1 damage-specific DNA binding protein 1 | AA963202 |
| 39 | UI-R-C0-hh-a-06-0-UI | Transcribed locus | AA996862 |
| 40 | UI-R-C0-jn-d-11-0-UI | Chloride intracellular channel 4 | AI030141 |
| 41 | UI-R-Y0-lv-h-03-0-UI | Heat shock transcription factor 1 | AI070360 |
| 42 | UI-R-C2-mw-h-01-0-UI | Guanine nucleotide binding protein, alpha 11 | AI385333 |
| 43 | UI-R-C2-nb-c-03-0-UI | Myotilin | AI071239 |
| 44 | UI-R-C2-nd-d-08-0-UI | Retinoic acid receptor responder (tazarotene induced) 2 | AI072016 |
| 45 | UI-R-C2-nn-f-08-0-UI | Casitas B-lineage lymphoma b | BF542300 |
| 46 | UI-R-C2p-ns-d-11-0-UI | Gene model 1960, (NCBI) | BF553317 |
| 47 | UI-R-C2p-nw-e-01-0-UI | RGD1564391 | AI136561 |
| 48 | UI-R-C2p-nz-c-11-0-UI | RNA pseudouridylate synthase domain containing 4 | AI136961 |
| 49 | UI-R-E0-bo-e-12-0-UI | Glutamate dehydrogenase 1 | AA859423 |
| 50 | UI-R-E0-bv-f-02-0-UI | Aryl hydrocarbon receptor | AA859478 |
| 51 | UI-R-C0-if-d-12-0-UI | THO complex subunit 4 (Tho4) (RNA and export factor binding protein 1) (REF1-I) (Ally of AML-1 and LEF-1) (Aly/REF) | BF561643 |
| 52 | UI-R-C0-ij-h-03-0-UI | Cat eye syndrome chromosome region, candidate 5 homolog | BF551092 |
| 53 | UI-R-C0-jp-c-12-0-UI | tubulin-specific chaperone d | AI031037 |
| 54 | UI-R-C1-jx-b-10-0-UI | Retinoblastoma-binding protein 2 (RBBP-2) | AI044642 |
| 55 | UI-R-C1-jy-e-12-0-UI | Septin 1 | AI045730 |
| 56 | UI-R-C1-kb-d-11-0-UI | Rho, GDP dissociation inhibitor (GDI) beta | BF550928 |
| 57 | UI-R-C1-kq-c-02-0-UI | Transcribed locus | AI058863 |
| 58 | UI-R-C1-kr-h-04-0-UI | Ubiquitin specific protease 32 | BF554444 |
| 59 | UI-R-C1-kw-c-08-0-UI | Rtf1, Paf1/RNA polymerase II complex component, homolog | BF547092 |
| 60 | UI-R-C1-ky-e-03-0-UI | SWI/SNF related, matrix associated, actin dependent regulator of chromatin, subfamily d, member 3 | BF559142 |
| 61 | UI-R-A0-aj-g-03-0-UI | 106 kDa O-GlcNAc transferase-interacting protein | AA866395 |
| 62 | UI-R-A0-am-e-06-0-UI | Myelin basic protein expression factor 2, repressor | AA818095 |
| 63 | UI-R-A0-ar-a-02-0-UI | Heterogeneous nuclear ribonucleoprotein D-like | AA818783 |
| 64 | UI-R-A0-av-e-05-0-UI | Guanylate nucleotide binding protein 2 | BF548349 |
| 65 | UI-R-E1-fn-f-07-0-UI | hypothetical protein FLJ25359 | BF556836 |
| 66 | UI-R-E1-ft-e-12-0-UI | Serine hydroxymethyltransferase 2 (mitochondrial) | AA957199 |
| 67 | UI-R-E1-fv-a-11-0-UI | Hypothetical protein LOC689959 | AA957899 |
| 68 | UI-R-C0-gr-b-02-0-UI | Reelin | BF557106 |
| 69 | UI-R-C0-hz-f-01-0-UI | Xenotropic and polytropic retrovirus receptor 1 | BF561471 |
| 70 | UI-R-C0-ic-b-11-0-UI | Hypothetical LOC305452 | AA998522 |
| 71 | UI-R-C0-gw-c-04-0-UI | Glutathione peroxidase 4 | AA964072 |
| 72 | UI-R-C0-hb-d-07-0-UI | Phosphofructokinase, muscle | AA964978 |
| 73 | UI-R-C0-jh-d-09-0-UI | Myxovirus (influenza virus) resistance 2 | AI030615 |
| 74 | UI-R-C0-jo-e-09-0-UI | RAB26, member RAS oncogene family | AI030702 |
| 75 | UI-R-C1-jv-f-04-0-UI | Toll-like receptor 4 | BF550815 |
| 76 | UI-R-C1-kc-a-10-0-UI | Potassium intermediate/small conductance calcium-activated channel, subfamily N, member 3 | BF550943 |
| 77 | UI-R-C1-ll-g-02-0-UI | Eukaryotic translation initiation factor 4E binding protein 1 | AI060153 |
| 78 | UI-R-C2-mr-e-03-0-UI | Carboxylesterase 2 (intestine, liver) | AI070587 |
| 79 | UI-R-C2-na-g-10-0-UI | Nclone10 mRNA | AI071000 |
| 80 | UI-R-C2-nh-h-06-0-UI | Pleiotrophin | BF549668 |
| 81 | UI-R-DK0-cfx-b-01-0-UI | Tetraspanin 9 | BI290594 |
| 82 | UI-R-DK0-cgb-h-10-0-UI | BM259 protein | BI290977 |
| 83 | UI-R-DK0-cgk-c-10-0-UI | Eukaryotic translation initiation factor 4E | BI297020 |
| 84 | UI-R-CV2-cgw-f-08-0-UI | Nucleolar protein 3 (apoptosis repressor with CARD domain) | BI297290 |
| 85 | UI-R-DQ0-cja-p-20-0-UI | Antxr2 protein | BI303662 |
| 86 | UI-R-E0-dl-e-08-0-UI | Protein-L-isoaspartate (D-aspartate) O-methyltransferase 1 | BF558340 |
| 87 | UI-R-A1-dr-d-05-0-UI | Vesicle docking protein | AA924366 |
| 88 | UI-R-A1-du-f-09-0-UI | Adrenergic receptor, alpha 1d | BF558823 |
| 89 | UI-R-E1-gi-b-09-0-UI | Latent transforming growth factor beta binding protein 1 | AA963308 |
| 90 | UI-R-E1-gp-f-05-0-UI | Proteasome (prosome, macropain) subunit, alpha type 3 | AA964414 |
| 91 | UI-R-DB0-bza-g-11-0-UI | S100 calcium-binding protein, ventral prostate | BI278703 |
| 92 | UI-R-DD0-bzv-a-05-0-UI | squamous cell carcinoma antigen 2 | BI285631 |
| 93 | UI-R-CW0s-cce-e-02-0-UI | Tenascin XA | BI282727 |
| 94 | UI-R-CX0s-ccp-b-01-0-UI | Legumain | BI285894 |
| 95 | UI-R-CX0s-cct-e-09-0-UI | Exostoses (multiple) 2 | BI284896 |
| 96 | UI-R-DK0-cdc-c-06-0-UI | ARP6 actin-related protein 6 homolog | BI288127 |
| 97 | UI-R-DK0-ceo-e-09-0-UI | Ring finger and CHY zinc finger domain containing 1 | BI294479 |
| 98 | UI-R-DK0-cez-d-02-0-UI | Metaxin 2 | BI296351 |
| 99 | UI-R-DK0-cfe-f-12-0-UI | Transmembrane protein 30A | BI289118 |
| 100 | UI-R-CM0-bjn-b-09-0-UI | XP_001108238.1 ribosomal protein L27 | BF395474 |
| 101 | UI-R-CN0-bmf-f-07-0-UI | SAM pointed domain containing ets transcription factor | BF410922 |
| 102 | UI-R-BT1-bmy-d-03-0-UI | Cut-like 1 | BF412079 |
| 103 | UI-R-BT1-bnt-c-09-0-UI | SRY-box containing gene 21 | BF412711 |
| 104 | UI-R-BJ2-bol-b-11-0-UI | Arrestin, beta 2 | BF413993 |
| 105 | UI-R-BJ2-bqj-a-10-0-UI | Transcription factor myocardin | BF418793 |
| 106 | UI-R-BJ2-bqp-c-07-0-UI | UDP-GlcNAc:betaGal beta-1,3-N-acetylglucosaminyltransferase 7 | BF419239 |
| 107 | UI-R-CW0-bvx-b-10-0-UI | Leucine-rich alpha-2-glycoprotein 1 | BG371585 |
| 108 | UI-R-CW0-bwg-g-11-0-UI | Proteasome (prosome, macropain) 28 subunit, alpha | BI274258 |
| 109 | UI-R-CZ0-byg-b-05-0-UI | Morf4 family associated protein 1 | BI278402 |
| 110 | UI-R-DA0-byo-a-03-0-UI | Eukaryotic translation initiation factor 2 alpha kinase 3 | BI279476 |
| 111 | UI-R-CA0-bfx-g-05-0-UI | Transcribed locus | BF394607 |
| 112 | UI-R-CA0-bgl-d-06-0-UI | Rabphilin 3A homolog | BF401508 |
| 113 | UI-R-CA1-biz-c-19-0-UI | Opioid receptor, kappa 1 | BF403560 |
| 114 | UI-R-CA1-bjd-g-21-0-UI | Phosphatidylinositol 4-kinase type 2 alpha | BF404296 |
| 115 | UI-R-CA1-bjf-m-05-0-UI | Ras-like without CAAX 2 | BF406174 |
| 116 | UI-R-CV1-brx-e-12-0-UI | Crystallin, gamma E | BG373338 |
| 117 | UI-R-CT0-bue-e-05-0-UI | SET translocation | BG381708 |
| 118 | UI-R-CT0-buo-h-09-0-UI | Protein tyrosine phosphatase, non-receptor type 11 | BG376388 |
| 119 | UI-R-CU0-buw-g-07-0-UI | Pleckstrin homology domain containing, family H (with MyTH4 domain) member 3 | BG377161 |
| 120 | UI-R-CU0-bvh-d-05-0-UI | Hexamthylene bis-acetamide inducible 2 | BG377974 |
| 121 | UI-R-CA0-axd-g-08-0-UI | Serine/threonine kinase 25 (STE20 homolog, yeast) | BE112873 |
| 122 | UI-R-CA0-axl-e-11-0-UI | ATPase, Na+/K+ transporting, alpha 3 polypeptide | BE110316 |
| 123 | UI-R-BS1-axy-d-08-0-UI | RIKEN cDNA 2010106G01 | BE116006 |
| 124 | UI-R-BS1-ayq-c-02-0-UI | Steroid receptor RNA activator 1 | BE107133 |
| 125 | UI-R-CA0-bar-a-04-0-UI | Solute carrier family 4, sodium bicarbonate transporter-like, member 10 | BE121003 |
| 126 | UI-R-CA1-bba-c-07-0-UI | XP_578736.1 ribosomal protein L21 | BF386125 |
| 127 | UI-R-CA1-bbr-a-01-0-UI | AT rich interactive domain 1B (Swi1 like) | BF387308 |
| 128 | UI-R-CA1-bcd-c-03-0-UI | Testis/prostate/placenta-expressed protein | BF391103 |
| 129 | UI-R-BS2-bem-b-05-0-UI | Protein phosphatase 1, regulatory (inhibitor) subunit 12A | BF398081 |
| 130 | UI-R-BS2-bfa-g-09-0-UI | Adipocyte-specific adhesion molecule | BF397632 |
| 131 | UI-R-BU0-ang-f-06-0-UI | RIKEN cDNA 1110025F24 | BF562508 |
| 132 | UI-R-BU0-apa-e-05-0-UI | Rap guanine nucleotide exchange factor (GEF) 1 | BE095513 |
| 133 | UI-R-BX0-arm-h-08-0-UI | Kinesin family member 5C | BE104278 |
| 134 | UI-R-BO1-asj-c-09-0-UI | Bladder cancer associated protein homolog | BE105881 |
| 135 | UI-R-BO1-asr-a-12-0-UI | C1GALT1-specific chaperone 1 | BE106692 |
| 136 | UI-R-BJ1-atb-a-10-0-UI | XP_001088137.1 ribosomal protein S21 | BE098629 |
| 137 | UI-R-BJ1-aup-g-04-0-UI | Calcium channel, voltage-dependent, gamma subunit 6 | CK839737 |
| 138 | UI-R-BJ1-avb-e-11-0-UI | Solute carrier family 25, member 1 | BE111503 |
| 139 | UI-R-BJ1-avo-d-03-0-UI | UDP-N-acetyl-alpha-D-galactosamine:polypeptide N-acetylgalactosaminyltransferase 7 | CK842612 |
| 140 | UI-R-BJ1-avu-b-05-0-UI | RIKEN cDNA 1810021J13 | BE110624 |
| 141 | UI-R-BO0-ahy-b-12-0-UI | CG1841-PA, isoform A | BF568021 |
| 142 | UI-R-BO0-aie-f-06-0-UI | Leucine rich repeat containing 4B | AW523930 |
| 143 | UI-R-BJ0p-ail-b-10-0-UI | ADP-ribosylarginine hydrolase | CK843960 |
| 144 | UI-R-BJ0p-ais-b-11-0-UI | Sperm associated antigen 5 | BF563328 |
| 145 | UI-R-BO1-ajv-f-12-0-UI | Expressed sequence AW060207 | BF565393 |
| 146 | UI-R-BT1-akd-d-10-0-UI | SEC14-like 2 | BF566339 |
| 147 | UI-R-BT1-akk-a-12-0-UI | XP_001096131.1 taxilin | AW529422 |
| 148 | UI-R-BT1-akq-g-11-0-UI | Potassium voltage-gated channel, subfamily H (eag-related), member 1 | AW530733 |
| 149 | UI-R-BU0-amt-a-08-0-UI | Triadin | AW529765 |
| 150 | UI-R-BU0-ana-e-09-0-UI | Myosin IC | BF564912 |
| 151 | UI-R-AB1-ys-a-10-0-UI | HSPC043 protein | CK840822 |
| 152 | UI-R-AB1-yy-h-08-0-UI | RIKEN cDNA 3200002M19 | CK840913 |
| 153 | UI-R-AF1-aav-c-09-0-UI | RIKEN cDNA B230312A22 | CK838463 |
| 154 | UI-R-Y0-abc-h-07-0-UI | Coagulation factor III | BF543279 |
| 155 | UI-R-Y0-abp-g-08-0-UI | Thyroid hormone receptor alpha | AI715302 |
| 156 | UI-R-Y0-acb-f-11-0-UI | GM2 ganglioside activator protein | AI717483 |
| 157 | UI-R-BJ0p-aez-a-08-0-UI | Transcribed locus |  |
| 158 | UI-R-BJ0p-afg-g-12-0-UI | 2010003J03Rik protein | CK839318 |
| 159 | UI-R-BJ0p-afn-d-11-0-UI | Tumor necrosis factor receptor superfamily, member 5 | AW433947 |
| 160 | UI-R-BJ0p-afx-b-08-0-UI | RIKEN cDNA E430013E20 gene | CK841273 |
| 161 | UI-R-C2p-rj-d-11-0-UI | Brain and kidney protein | BF546314 |
| 162 | UI-R-C2p-rs-g-03-0-UI | ST8 alpha-N-acetyl-neuraminide alpha-2,8-sialyltransferase 1 | AI501037 |
| 163 | UI-R-C2p-sb-f-07-0-UI | F-box and WD-40 domain protein 9 | AI501481 |
| 164 | UI-R-C3-sj-f-12-0-UI | NP_064525.1 protein; amyloid-beta precursor protein intracellular domain associated protein 1; cajalin 2 | BF542518 |
| 165 | UI-R-C3-ua-a-06-0-UI | PDZ domain containing 8 | AI549380 |
| 166 | UI-R-G0-uk-b-08-0-UI | G kinase anchoring protein 1 | AI577679 |
| 167 | UI-R-Y0-uw-d-09-0-UI | Solute carrier family 7 (cationic amino acid transporter, y+ system), member 3 | BF522753 |
| 168 | UI-R-Y0-vd-f-07-0-UI | Protein tyrosine phosphatase, receptor type, D | BF523782 |
| 169 | UI-R-AC1-xs-h-05-0-UI | 3222401M22Rik protein | CK839033 |
| 170 | UI-R-AC0-yi-a-07-0-UI | POU domain, class 2, transcription factor 1 | CK838820 |
| 171 | UI-R-E1-fx-h-08-0-UI | Plexin B2 | AA962980 |
| 172 | UI-R-E1-gg-b-05-0-UI | DEAH (Asp-Glu-Ala-His) box polypeptide 8 | AA963697 |
| 173 | UI-R-C0-jd-g-09-0-UI | RIKEN cDNA D430028G21 | AI502240 |
| 174 | UI-R-C1-jy-d-08-0-UI | Transcribed locus |  |
| 175 | UI-R-C1-kt-f-12-0-UI | Nitric oxide synthase trafficker | BF552610 |
| 176 | UI-R-C1-lj-b-05-0-UI | Phosphatidylinositol-4-phosphate 5-kinase, type 1, beta | BF546729 |
| 177 | UI-R-C2p-oh-e-08-0-UI | Potassium voltage-gated channel, subfamily G, member 3 | AI137172 |
| 178 | UI-R-BT0-pm-b-03-0-UI | Protein tyrosine phosphatase, non-receptor type 12 | BF553701 |
| 179 | UI-R-BT0-ps-d-12-0-UI | Sulfatase 2 | BF553871 |
| 180 | UI-R-BT0-qa-b-09-0-UI | Transcribed locus | AI454016 |
| 181 | UI-R-A0-ai-d-12-0-UI | NP_663449.2 P450-like | AA818057 |
| 182 | UI-R-A0-ay-g-05-0-UI | ankyrin repeat domain protein 17 isoform b | AI454370 |
| 183 | UI-R-A0-bm-e-10-0-UI | Prohibitin | AA866323 |
| 184 | UI-R-E0-br-h-04-0-UI | Lipocalin 7 | AA866478 |
| 185 | UI-R-E0-cw-f-07-0-UI | Nucleolar and spindle associated protein 1 | BF550184 |
| 186 | UI-R-E0-dd-g-07-0-UI | MOB1, Mps One Binder kinase activator-like 1A | AA900230 |
| 187 | UI-R-E0-dk-f-08-0-UI | DNA (cytosine-5-)-methyltransferase 1 | AA900818 |
| 188 | UI-R-A1-dq-h-05-0-UI | Transcribed locus | AA924003 |
| 189 | UI-R-A1-eq-c-05-0-UI | Nidogen 2 | BF558902 |
| 190 | UI-R-E1-fc-c-02-0-UI | Ras homolog enriched in brain | AA955709 |
| 191 | UI-R-CS0-bth-c-11-0-UI | PAS domain containing serine/threonine kinase | BG379473 |
| 192 | UI-R-CX0-bxi-g-09-0-UI | PRO1853 homolog | BI275982 |
| 193 | UI-R-DO0-cix-m-07-0-UI | Nudix (nucleoside diphosphate linked moiety X)-type motif 19 | BI302922 |
| 194 | UI-R-E1-gd-b-11-0-UI | Transcribed locus |  |
| 195 | UI-R-C0-gr-f-03-0-UI | hypothetical protein MGC9712 | AA964996 |
| 196 | UI-R-C0-ig-f-04-0-UI | Death associated transcription factor 1 | AA998669 |
| 197 | UI-R-C1-jw-h-09-0-UI | RIKEN cDNA G431001E03 gene | AI044504 |
| 198 | UI-R-C2p-qw-f-07-0-UI | Membrane-spanning 4-domains, subfamily A, member 10 | AI555523 |
| 199 | UI-R-C2p-rn-b-10-0-UI | Ubiquitin specific protease 25 | AI500911 |
| 200 | UI-R-C0-iq-d-01-0-UI | Ribonuclease H1 | AI029282 |
| 201 | UI-R-C0-iw-h-06-0-UI | Coatomer protein complex, subunit gamma | BF551615 |
| 202 | UI-R-C1-kl-f-12-0-UI | Proline dehydrogenase (oxidase) 2 | AI058310 |
| 203 | UI-R-C1-ko-e-02-0-UI | Ectodermal-neural cortex 1 | AI071307 |
| 204 | UI-R-C1-lh-e-08-0-UI | Retinoblastoma binding protein 6 | BF546654 |
| 205 | UI-R-C1-lm-b-10-0-UI | Metastasis associated 3 | AI060288 |
| 206 | UI-R-C0-gr-e-06-0-UI | Zinc finger protein 655 | AA964988 |
| 207 | UI-R-C0-ii-b-10-0-UI | Claudin 5 | AA998729 |
| 208 | UI-R-C1-kb-h-08-0-UI | Serine/threonine kinase 19 | BF550939 |
| 209 | UI-R-C1-lr-d-04-0-UI | Spastic paraplegia 21 homolog | BF559435 |
| 210 | UI-R-C0-hj-h-06-0-UI | Phosphatase and actin regulator 1 | AA996943 |
| 211 | UI-R-C0-ir-h-11-0-UI | Transcribed locus | BF554530 |
| 212 | UI-R-C1-js-f-09-0-UI | Proline-rich polypeptide 3 | AI043989 |
| 213 | UI-R-C1-lj-b-08-0-UI | RUN and TBC1 domain containing 3 | BF549569 |
| 214 | UI-R-A1-eu-h-05-0-UI | Polycomb group ring finger 1 | AA926371 |
| 215 | UI-R-E1-fc-h-05-0-UI | Sin3-associated polypeptide, 18kDa | AA955677 |
| 216 | UI-R-E1-fv-f-03-0-UI | ATP-binding cassette, sub-family F (GCN20), member 3 | BF556575 |
| 217 | UI-R-E1-fz-a-03-0-UI | Myeloid leukemia factor 1 | AA957805 |
| 218 | UI-R-C0-ig-b-12-0-UI | Odd-skipped related 2 | BF553961 |
| 219 | UI-R-C0-il-h-08-0-UI | Metal response element binding transcription factor 1 | BF551324 |
| 220 | UI-R-A1-eq-a-09-0-UI | Heterogeneous nuclear ribonucleoproteins methyltransferase-like 2 | BF556406 |
| 221 | UI-R-A1-ex-a-11-0-UI | ATPase, Ca++ transporting, plasma membrane 2 | BF560112 |
| 222 | UI-R-C0-io-a-08-0-UI | Prostaglandin F2 receptor negative regulator | BF551367 |
| 223 | UI-R-C0-iw-c-08-0-UI | Replication factor C 1 | BF552681 |
| 224 | UI-R-C0-jm-f-01-0-UI | Small nuclear ribonucleoprotein polypeptides B and B1 | AI043856 |
| 225 | UI-R-BT0-qe-e-06-0-UI | Syntaxin binding protein 1 | AI145393 |
| 226 | UI-R-C1-lh-b-02-0-UI | Transcribed locus | AI059762 |
| 227 | UI-R-C2-ms-f-04-0-UI | Oxysterol binding protein-like 1A | BF542294 |
| 228 | UI-R-E0-bs-d-02-0-UI | Ectonucleotide pyrophosphatase/phosphodiesterase 3 | AA859670 |
| 229 | UI-R-E0-dc-a-06-0-UI | Transcribed locus | AA900892 |
| 230 | UI-R-C2-nf-c-07-0-UI | SNF1-like kinase | AI072078 |
| 231 | UI-R-C2-nh-a-10-0-UI | Seizure related 6 homolog | BF521811 |
| 232 | UI-R-C2-nj-h-02-0-UI | RT1 class II, locus Bb | BF553199 |
| 233 | UI-R-C2-nl-h-04-0-UI | CCR4-NOT transcription complex, subunit 7 | AI071980 |
| 234 | UI-R-C1-kp-d-01-0-UI | Nucleoporin 153 | BF554373 |
| 235 | UI-R-A0-as-f-04-0-UI | Albumin | AA818960 |
| 236 | UI-R-A0-az-h-03-0-UI | Amyloid beta (A4) precursor-like protein 2 | AA818665 |
| 237 | UI-R-A0-bh-h-10-0-UI | Hydroxysteroid 11-beta dehydrogenase 1 | BF555224 |
| 238 | UI-R-E0-cq-c-10-0-UI | XP_001102188.1 peroxiredoxin 1 isoform 2 | AA875245 |
| 239 | UI-R-E0-cz-g-09-0-UI | Alkaline phosphatase, tissue-nonspecific | BF555607 |
| 240 | UI-R-C0-jg-b-05-0-UI | Glycine decarboxylase | AI029729 |
| 241 | UI-R-C0-jj-b-09-0-UI | Transcribed locus | AI043720 |
| 242 | UI-R-C1-kd-d-09-0-UI | RIKEN cDNA 2310042P20 | BF551516 |
| 243 | UI-R-C1-kf-f-10-0-UI | Oxidation resistance 1 | AI045087 |
| 244 | UI-R-C1-ki-h-11-0-UI | Phosphoribosylformylglycinamidine synthase (FGAR amidotransferase) | AI044757 |
| 245 | UI-R-C1-kk-e-12-0-UI | Adaptor-related protein complex 1, sigma 2 subunit | BF551686 |
| 246 | UI-R-C1-lb-e-02-0-UI | Biogenesis of lysosome-related organelles complex-1, subunit 2 | AI059281 |
| 247 | UI-R-C1-lf-a-05-0-UI | WAP four-disulfide core domain 10A precursor | BF549536 |
| 248 | UI-R-C1-lo-d-07-0-UI | Oxysterol binding protein | AI069929 |
| 249 | UI-R-C1-lq-a-04-0-UI | TRM5 tRNA methyltransferase 5 homolog | AI058972 |
| 250 | UI-R-A1-ey-e-11-0-UI | ankyrin repeat and SOCs box-containing protein 5 | AA955046 |
| 251 | UI-R-E1-ff-c-02-0-UI | Mitochondrial ribosomal protein L15 | AA955952 |
| 252 | UI-R-E1-fg-h-03-0-UI | ATPase, Na+/K+ transporting, beta 1 polypeptide | AA956184 |
| 253 | UI-R-E1-fl-c-04-0-UI | RAC/CDC42 exchange factor | AI113076 |
| 254 | UI-R-C0-gx-f-07-0-UI | Phytanoyl-CoA 2-hydroxylase 2 | BF560860 |
| 255 | UI-R-C0-he-b-04-0-UI | component of oligomeric golgi complex 2 | BF557930 |
| 256 | UI-R-C0-hi-e-07-0-UI | Steroid 5 alpha-reductase 1 | BF561273 |
| 257 | UI-R-C0-hv-a-09-0-UI | COP9 (constitutive photomorphogenic) homolog, subunit 5 | AA997760 |
| 258 | UI-R-C0-it-h-12-0-UI | CG12753-PA | BF551574 |
| 259 | UI-R-C0-iy-h-06-0-UI | Stimulated by retinoic acid gene 6 homolog | AI029719 |
| 260 | UI-R-C0-hu-d-11-0-UI | Thymosin beta-like protein 1 | AA997865 |
| 261 | UI-R-C0-ja-c-12-0-UI | Homeo box, msh-like 1 | AI029934 |
| 262 | UI-R-C1-ki-d-07-0-UI | Fumarylacetoacetate hydrolase | AI044732 |
| 263 | UI-R-C1-kp-a-10-0-UI | Growth hormone releasing hormone | AI058642 |
| 264 | UI-R-C1-kx-c-12-0-UI | Arg/Abl-interacting protein ArgBP2 | BF546366 |
| 265 | UI-R-C1-lf-g-12-0-UI | Syntaxin 12 | AI059347 |
| 266 | UI-R-C2p-nw-b-11-0-UI | Membrane metallo endopeptidase | AI136717 |
| 267 | UI-R-C2p-oi-a-03-0-UI | Solute carrier family 12, member 1 | AI137982 |
| 268 | UI-R-BT0-po-f-03-0-UI | Prostaglandin D2 synthase | AI144744 |
| 269 | UI-R-A0-af-a-12-0-UI | Block of proliferation 1 | BF549115 |
| 270 | UI-R-CV2-chu-a-02-0-UI | COMM domain containing 10 | BI299340 |
| 271 | UI-R-DM0-ciq-d-21-0-UI | Proline-rich protein | BI302321 |
| 272 | UI-R-DN0-ciu-l-20-0-UI | Major vault protein | BI292135 |
| 273 | UI-R-DO0-ciw-o-12-0-UI | Keratin complex 2, basic, gene 8 | BI292603 |
| 274 | UI-R-A1-ea-b-01-0-UI | Maternal G10 transcript | AA924681 |
| 275 | UI-R-A1-ec-h-02-0-UI | Isocitrate dehydrogenase 1 (NADP+), soluble | BF550286 |
| 276 | UI-R-A1-eh-b-10-0-UI | NP_066390.1 histone family, member A | AA924988 |
| 277 | UI-R-A1-el-b-05-0-UI | ATP synthase, H+ transporting, mitochondrial F1 complex, delta subunit | AA926040 |
| 278 | UI-R-C0-hi-g-04-0-UI | Aurora kinase B | AA996466 |
| 279 | UI-R-C0-hp-d-12-0-UI | Isovaleryl coenzyme A dehydrogenase | AA998915 |
| 280 | UI-R-CU0s-cbs-d-10-0-UI | XP_001087784.1 NADH dehydrogenase (ubiquinone) 1 alpha subcomplex, 10, 42kDa precursor isoform 1 | BI281779 |
| 281 | UI-R-CU0s-cby-h-03-0-UI | G protein pathway suppressor 1 | BI282292 |
| 282 | UI-R-DK0-cdk-d-12-0-UI | Liver regeneration-related protein | BI293160 |
| 283 | UI-R-DK0-cdu-d-09-0-UI | Talin 1 | BI293678 |
| 284 | UI-R-DK0-cec-f-12-0-UI | Chemokine (C-C motif) ligand 4 | BI294833 |
| 285 | UI-R-DK0-cef-e-03-0-UI | Fc receptor, IgG, low affinity III | BI295071 |
| 286 | UI-R-CA0-bmb-a-06-0-UI | Protein phosphatase 1G (formerly 2C), magnesium-dependent, gamma isoform | BF410676 |
| 287 | UI-R-BJ2-bra-f-03-0-UI | Signal sequence receptor, alpha | BF408176 |
| 288 | UI-R-BJ2-brf-f-02-0-UI | Abelson helper integration site 1 | BF408595 |
| 289 | UI-R-DK0-cfo-f-09-0-UI | Zinc finger protein 161 | BI289823 |
| 290 | UI-R-BJ2-bos-f-02-0-UI | CD79B antigen | CK844561 |
| 291 | UI-R-BJ2-bpi-e-04-0-UI | Symplekin | CK844587 |
| 292 | UI-R-BJ2-bpp-c-09-0-UI | Cytochrome P450, family 27, subfamily a, polypeptide 1 | CK843131 |
| 293 | UI-R-BJ2-bpy-a-07-0-UI | XP_001082677.1 hypothetical protein LOC54537 isoform 2 | BF420607 |
| 294 | UI-R-CX0-bwq-g-04-0-UI | hypothetical protein FLJ22555 | BI276472 |
| 295 | UI-R-CX0-bxd-g-06-0-UI | Collectin sub-family member 12 | BI274764 |
| 296 | UI-R-CX0-bxk-h-06-0-UI | neighbor of Brca1 gene 1 | BI277903 |
| 297 | UI-R-CY0-bxs-g-07-0-UI | Fatty acid binding protein 4, adipocyte | BI277568 |
| 298 | UI-R-DE0-cag-g-05-0-UI | RT1 class Ib, locus Aw2 | BI280335 |
| 299 | UI-R-DC0-cay-e-05-0-UI | Proteasome (prosome, macropain) 28 subunit, beta | BI279893 |
| 300 | UI-R-CA1-bib-e-02-0-UI | Matrix metalloproteinase 16 | BF404689 |
| 301 | UI-R-CA1-biq-e-07-0-UI | Cold inducible RNA binding protein | BF405160 |
| 302 | UI-R-CV1-bsf-d-05-0-UI | Transcribed locus | BG373089 |
| 303 | UI-R-CV1-bsr-g-09-0-UI | Amplified in osteosarcoma | BG374595 |
| 304 | UI-R-CS0-btl-h-11-0-UI | DEAD (Asp-Glu-Ala-Asp) box polypeptide 17 | BG380090 |
| 305 | UI-R-CT0-btw-g-09-0-UI | Neurexin 3 | BG380402 |
| 306 | UI-R-CV1-bvq-e-12-0-UI | ATP synthase, H+ transporting, mitochondrial F0 complex, subunit d | BG378308 |
| 307 | UI-R-CA0-bkk-a-09-0-UI | Melanocortin 4 receptor | BF416476 |
| 308 | UI-R-CN0-bky-g-04-0-UI | Transcribed locus |  |
| 309 | UI-R-CA1-blh-e-01-0-UI | Neuropeptide B | BG376816 |
| 310 | UI-R-BS1-ayx-e-08-0-UI | R3H domain containing 1 | BE108288 |
| 311 | UI-R-BS1-azf-e-04-0-UI | Postsynaptic protein Cript | BE117746 |
| 312 | UI-R-BS1-azr-h-10-0-UI | Jumonji domain containing 3 | BE118720 |
| 313 | UI-R-CA0-bak-f-10-0-UI | Solute carrier family 1 (glial high affinity glutamate transporter), member 3 | BE120108 |
| 314 | UI-R-CA1-bcq-b-12-0-UI | RIKEN cDNA 1110063F24 | BF391175 |
| 315 | UI-R-BS2-bde-f-09-0-UI | Leucine rich repeat containing 8 family, member D | BF388799 |
| 316 | UI-R-BS2-bdq-b-03-0-UI | XP_001096972.1 RAB3 GTPase-activating protein | BF389908 |
| 317 | UI-R-BS2-bea-a-02-0-UI | Apolipoprotein B | BF396597 |
| 318 | UI-R-CA0-bgw-f-02-0-UI | FCH and double SH3 domains 2 | BF393917 |
| 319 | UI-R-CA0-bhi-d-08-0-UI | Angiopoietin 1 | BF402869 |
| 320 | UI-R-BT1-aqi-c-12-0-UI | Gastric inhibitory polypeptide receptor | BE101942 |
| 321 | UI-R-BT1-aqx-a-05-0-UI | Homogentisate 1, 2-dioxygenase | BE103138 |
| 322 | UI-R-BJ1-atl-f-01-0-UI | XP_001092331.1 trafficking protein particle complex 2 | CK844939 |
| 323 | UI-R-BJ1-atr-b-05-0-UI | RIKEN cDNA 4930538D17 | CK843003 |
| 324 | UI-R-BJ1-atw-h-10-0-UI | 1-acylglycerol-3-phosphate O-acyltransferase 5 (lysophosphatidic acid acyltransferase, epsilon) | BE099485 |
| 325 | UI-R-BJ1-auf-g-08-0-UI | Zinc finger, DHHC domain containing 4 | CK842121 |
| 326 | UI-R-BJ1-avy-h-08-0-UI | Solute carrier family 39 (metal ion transporter), member 6 | CK844258 |
| 327 | UI-R-BJ1-awd-a-11-0-UI | Programmed cell death protein 11 | BE113231 |
| 328 | UI-R-BJ1-awk-e-08-0-UI | putative phosphatase subunit | CK839970 |
| 329 | UI-R-CA0-awv-f-09-0-UI | Pancreas specific transcription factor, 1a | BE107609 |
| 330 | UI-R-BO1-aiy-g-01-0-UI | Ubiquitin specific protease 38 | AW526053 |
| 331 | UI-R-BO1-ajg-b-12-0-UI | Toll-like receptor 2 | BF563187 |
| 332 | UI-R-BO1-ajj-f-05-0-UI | Glutamic acid decarboxylase 1 | BF565262 |
| 333 | UI-R-BO1-ajq-a-12-0-UI | NP_035880.1 finger protein 162 | AW529858 |
| 334 | UI-R-C4-akz-h-07-0-UI | DEAD (Asp-Glu-Ala-Asp) box polypeptide 27 | BF565792 |
| 335 | UI-R-C4-alh-g-04-0-UI | Protein kinase, AMP-activated, alpha 2 catalytic subunit | BF564138 |
| 336 | UI-R-C4-alp-e-05-0-UI | EGL nine homolog 3 | AW534130 |
| 337 | UI-R-C4-aly-a-08-0-UI | Hook homolog 3 | AW535564 |
| 338 | UI-R-BU0-aph-d-04-0-UI | Heterogeneous nuclear ribonucleoprotein F | BE096542 |
| 339 | UI-R-BO1-apv-h-02-0-UI | Zic family member 2 (odd-paired homolog, Drosophila) | BE097311 |
| 340 | UI-R-AA1-zz-c-12-0-UI | Aldehyde dehydrogenase family 5, subfamily A1 | AI710075 |
| 341 | UI-R-AG1-aam-c-11-0-UI | Phosphoribosyl pyrophosphate synthetase-associated protein 1 | CK843342 |
| 342 | UI-R-Y0-aci-d-07-0-UI | Phosphatidylinositol transfer protein, membrane-associated 1 | BF524014 |
| 343 | UI-R-Y0-acv-a-05-0-UI | LOC361111 | AI763946 |
| 344 | UI-R-BJ0-ade-c-01-0-UI | Protein inhibitor of activated STAT 3 | CK841091 |
| 345 | UI-R-BJ0-aed-a-06-0-UI | WD repeat and SOCS box-containing 2 | AW253927 |
| 346 | UI-R-BJ0p-age-f-06-0-UI | Succinate dehydrogenase complex, subunit A, flavoprotein (Fp) | AW520890 |
| 347 | UI-R-BO0-agm-c-02-0-UI | Beta-1,3-glucuronyltransferase 2 (glucuronosyltransferase S) | BF566519 |
| 348 | UI-R-BO0-agy-e-02-0-UI | chromosome 11 open reading frame2 | BF567338 |
| 349 | UI-R-BO0-aho-e-07-0-UI | Transient receptor potential cation channel, subfamily M, member 3 | BF567888 |
| 350 | UI-R-C3-sr-h-12-0-UI | DEAH (Asp-Glu-Ala-His) box polypeptide 37 | BF542805 |
| 351 | UI-R-C3-sz-f-10-0-UI | Transcribed locus | AI547647 |
| 352 | UI-R-C3-tk-d-04-0-UI | Ica69-related protein | AI578083 |
| 353 | UI-R-C3-ts-d-11-0-UI | Ghrelin precursor | AI549172 |
| 354 | UI-R-AB0-vv-d-04-0-UI | Creatine kinase, brain | CK840391 |
| 355 | UI-R-AD0-wg-g-09-0-UI | Phosphoribosylglycinamide formyltransferase | CK842345 |
| 356 | UI-R-AG0-wu-c-09-0-UI | Bcl2-interacting killer | AI579311 |
| 357 | UI-R-AE0-xd-a-08-0-UI | Peptidylprolyl isomerase B | AI602605 |
| 358 | UI-R-AE1-zj-h-10-0-UI | XP_001089751.1 phosphorylase kinase gamma subunit 1 | CK839133 |
| 359 | UI-R-AD1-zp-f-04-0-UI | Kelch domain containing 3 | CK842383 |
| 360 | UI-R-C0-hr-g-06-0-UI | Acireductone dioxygenase 1 | AA997430 |
| 361 | UI-R-C0-ie-c-08-0-UI | Serine/threonine/tyrosine interacting-like 1 | AA998484 |
| 362 | UI-R-Y0-mb-g-11-0-UI | small nuclear ribonucleoprotein E | AI073278 |
| 363 | UI-R-Y0-mo-h-09-0-UI | EGF-containing fibulin-like extracellular matrix protein 2 | AI111990 |
| 364 | UI-R-C2-nf-d-02-0-UI | Wnt inhibitory factor 1 | BF552992 |
| 365 | UI-R-C2p-nx-a-06-0-UI | MYG1 protein | AI136010 |
| 366 | UI-R-BT0-qh-h-05-0-UI | Splicing factor 3a, subunit 1 | BF544607 |
| 367 | UI-R-C2p-qn-g-09-0-UI | LUC7-like | BF522315 |
| 368 | UI-R-C2p-qt-h-05-0-UI | EH-domain containing 2 | BF545109 |
| 369 | UI-R-C2p-rd-e-05-0-UI | Zeta-chain (TCR) associated protein kinase 70 | BF545972 |
| 370 | UI-R-E0-bv-g-04-0-UI | Insulin-like growth factor binding protein-like 1 | BF549939 |
| 371 | UI-R-E0-ca-h-11-0-UI | Cyclin M4 | AA859983 |
| 372 | UI-R-E0-ci-b-05-0-UI | Transmembrane protein 93 | AA874930 |
| 373 | UI-R-E0-co-c-05-0-UI | Dendritic cell protein GA17 | BF549876 |
| 374 | UI-R-A1-dw-a-03-0-UI | Adiponectin receptor 2 | AA901328 |
| 375 | UI-R-A1-dz-d-03-0-UI | Synaptotagmin I | AA924659 |
| 376 | UI-R-A1-ef-b-02-0-UI | F-box and WD-40 domain protein 5 | BF556268 |
| 377 | UI-R-A1-ej-g-03-0-UI | Solute carrier family 25, member 26 | BF544110 |
| 378 | UI-R-C0-gr-h-07-0-UI | RIKEN cDNA A530089I17 | BF557398 |
| 379 | UI-R-C0-hm-b-04-0-UI | Phytanoyl-CoA hydroxylase interacting protein-like | BF556996 |
| 380 | UI-R-BT0-qf-f-06-0-UI | Hypothetical LOC292874 | AI145258 |
| 381 | UI-R-Y0-acr-a-07-0-UI | Thioredoxin domain containing 8 | AI764117 |
| 382 | UI-R-C4-alr-f-09-0-UI | RIKEN cDNA 2310011J03 | AW533994 |
| 383 | UI-R-BS2-beo-a-03-0-UI | Nucleolar GTP-binding protein 1 (Chronic renal failure gene protein) (GTP-binding protein NGB) | BF398367 |
| 384 | UI-R-E0-cc-d-03-0-UI | Related RAS viral (r-ras) oncogene homolog 2 | BF559046 |
| 385 | UI-R-E0-dl-d-05-0-UI | Retinoblastoma binding protein 6 | AA900525 |
| 386 | UI-R-A1-dz-a-08-0-UI | Glyoxylate reductase/hydroxypyruvate reductase | AA924630 |
| 387 | UI-R-E1-fe-b-03-0-UI | Chromobox homolog 1 (Drosophila HP1 beta) | BF561085 |
| 388 | UI-R-C2-mw-g-12-0-UI | DEAD (Asp-Glu-Ala-Asp) box polypeptide 59 | BF521750 |
| 389 | UI-R-C2p-ok-b-02-0-UI | IGFBP-2-Binding Protein, IIp45 | BF552116 |
| 390 | UI-R-C0-hc-e-11-0-UI | Dehydrogenase/reductase (SDR family) member 3 | AA965122 |
| 391 | UI-R-C0-hk-a-06-0-UI | DKFZP434H132 protein | BF558075 |
| 392 | UI-R-C0-jf-b-12-0-UI | F-box only protein 22 | AI030899 |
| 393 | UI-R-C0-jo-c-08-0-UI | Ubiquitin specific peptidase 9, X chromosome | BF554676 |
| 394 | UI-R-C1-jt-e-05-0-UI | G protein-coupled receptor, family C, group 5, member C | BF551155 |
| 395 | UI-R-C1-jz-g-10-0-UI | tRNA-splicing endonuclease subunit Sen54 (tRNA-intron endonuclease Sen54) | AI045468 |
| 396 | UI-R-E0-cl-c-12-0-UI | Solute carrier family 29 (nucleoside transporters), member 2 | AA859360 |
| 397 | UI-R-E1-fb-b-02-0-UI | Solute carrier family 3 (activators of dibasic and neutral amino acid transport), member 2 | BF557361 |
| 398 | UI-R-A0-ar-d-12-0-UI | Myelin protein zero-like 1 | BF549190 |
| 399 | UI-R-E1-fb-g-08-0-UI | 2010321M09Rik protein | AA955997 |
| 400 | UI-R-C0-ig-d-01-0-UI | Serine/threonine kinase 10 | AA998657 |
| 401 | UI-R-A0-aw-h-06-0-UI | Nudix (nucleoside diphosphate linked moiety X)-type motif 22 | BF547640 |
| 402 | UI-R-E1-fc-c-01-0-UI | Transcribed locus | AA955708 |
| 403 | UI-R-C0-gv-a-01-0-UI | Oligodendrocyte-myelin glycoprotein | BF557291 |
| 404 | UI-R-E0-bp-h-02-0-UI | Tubulin, beta 2b | AA899219 |
| 405 | UI-R-A0-bh-h-05-0-UI | NP_001037058.1 receptor-related 2 (herpesvirus entry mediator B) | BF555222 |
| 406 | UI-R-A0-ay-e-07-0-UI | RGD1559720 | AA818721 |
| 407 | UI-R-A1-es-h-08-0-UI | GTP binding protein 5 | AA925931 |
| 408 | UI-R-C0-gt-g-08-0-UI | testes development-related NYD-SP22 isoform 1 | AA964117 |
| 409 | UI-R-C0-gz-e-04-0-UI | Protein O-fucosyltransferase 2 | BF556708 |
| 410 | UI-R-E0-cf-c-01-0-UI | WAP four-disulfide core domain 1 | AA874977 |
| 411 | UI-R-E0-cl-c-05-0-UI | NP_066298.1 histone family, member I | BF559485 |
| 412 | UI-R-E1-fh-a-10-0-UI | Prenylated SNARE protein | AA956066 |
| 413 | UI-R-E1-fu-e-02-0-UI | Pleckstrin homology, Sec7 and coiled-coil domains 2 | AA957298 |
| 414 | UI-R-E1-ge-d-08-0-UI | Tubulin, alpha 1 | AA957078 |
| 415 | UI-R-C0-ih-c-10-0-UI | Hemopoietic cell kinase | BF554004 |
| 416 | UI-R-E1-fr-g-10-0-UI | Phosphatidylinositol glycan, class M | BF562101 |
| 417 | UI-R-C0-gt-f-09-0-UI | Transcribed locus | AA964107 |
| 418 | UI-R-C0-iw-f-09-0-UI | Phosphoribosyl pyrophosphate amidotransferase | AI029409 |
| 419 | UI-R-C1-jv-h-05-0-UI | Jun-B oncogene | AI044131 |
| 420 | UI-R-C2-mw-a-04-0-UI | Hypothetical LOC310540 | BF549607 |
| 421 | UI-R-C2-na-c-01-0-UI | Bromodomain containing 2 | AI070951 |
| 422 | UI-R-C2-nc-a-02-0-UI | HGFL protein | AI071477 |
| 423 | UI-R-C2-ne-b-12-0-UI | Protein kinase, membrane associated tyrosine/threonine 1 | AI072387 |
| 424 | UI-R-C2p-nq-a-12-0-UI | Glucosidase 1 | BF521832 |
| 425 | UI-R-C2p-nv-a-08-0-UI | Sh3kbp1 binding protein 1 | AI136469 |
| 426 | UI-R-C2p-nx-g-12-0-UI | Elastase 3B, pancreatic | AI136077 |
| 427 | UI-R-C2p-ob-c-01-0-UI | Phosphatase and actin regulator 3 | BF553801 |
| 428 | UI-R-E0-bt-h-08-0-UI | ATPase, Ca++ transporting, plasma membrane 1 | AA858553 |
| 429 | UI-R-E0-bz-e-09-0-UI | Microtubule-associated protein 1 light chain 3 beta | AA860032 |
| 430 | UI-R-C0-ii-h-03-0-UI | Hypothetical protein MGC11690 | BF551076 |
| 431 | UI-R-C0-ik-h-12-0-UI | Methionyl aminopeptidase 1 | BF551309 |
| 432 | UI-R-C0-jp-h-09-0-UI | Interleukin 17 receptor E | BF550982 |
| 433 | UI-R-C1-jx-f-08-0-UI | Bardet-Biedl syndrome 2 homolog | BF550866 |
| 434 | UI-R-C1-jz-a-05-0-UI | Myeloid cell surface antigen CD33 precursor (gp67) (Siglec-3) | BF550882 |
| 435 | UI-R-C1-kc-d-10-0-UI | XP_872487.2 KAT protein isoform 2 [Bos taurus] | AI044529 |
| 436 | UI-R-C1-kq-h-12-0-UI | hypothetical protein MGC18873 | AI059188 |
| 437 | UI-R-C1-ks-g-05-0-UI | hypothetical protein PRO0971 | BF550625 |
| 438 | UI-R-C1-kx-d-12-0-UI | Fucose-1-phosphate guanylyltransferase | BF546372 |
| 439 | UI-R-C1-kz-e-12-0-UI | SECIS binding protein 2 | AI060054 |
| 440 | UI-R-A0-al-f-09-0-UI | Ras homolog enriched in brain like 1 | AA819212 |
| 441 | UI-R-A0-aq-a-12-0-UI | ATP synthase, H+ transporting, mitochondrial F1 complex, O subunit | AA819876 |
| 442 | UI-R-A0-as-f-12-0-UI | Cadherin EGF LAG seven-pass G-type receptor 2 | BF548849 |
| 443 | UI-R-A0-be-c-07-0-UI | Transcribed locus | AA858731 |
| 444 | UI-R-E1-fo-h-11-0-UI | Hypothetical LOC298018 | AI112804 |
| 445 | UI-R-E1-fu-a-05-0-UI | hypothetical protein | BF556148 |
| 446 | UI-R-E1-gq-b-07-0-UI | Golgi SNAP receptor complex member 2 | AA964687 |
| 447 | UI-R-C0-gw-d-12-0-UI | channel-interacting PDZ domain protein isoform 1 | AA964092 |
| 448 | UI-R-C0-ib-b-03-0-UI | IQ motif and WD repeats 1 | BF561661 |
| 449 | UI-R-C0-ie-a-11-0-UI | Transcribed locus | AA998478 |
| 450 | UI-R-C0-gy-d-01-0-UI | Solute carrier family 37 (glycerol-6-phosphate transporter), member 4 | AA964286 |
| 451 | UI-R-C0-he-f-02-0-UI | Urate oxidase | AA996409 |
| 452 | UI-R-C0-jl-h-03-0-UI | Ribonuclease/angiogenin inhibitor 1 | AI043708 |
| 453 | UI-R-C1-js-h-11-0-UI | ATPase, Ca++ transporting, cardiac muscle, slow twitch 2 | AI044003 |
| 454 | UI-R-C1-jx-g-10-0-UI | Ninjurin 1 | AI044670 |
| 455 | UI-R-C1-ke-c-03-0-UI | CD1d1 antigen | AI044941 |
| 456 | UI-R-C1-lp-f-10-0-UI | Low density lipoprotein receptor-related protein 3 | AI070184 |
| 457 | UI-R-C2-mv-g-04-0-UI | Nucleoporin 107 | AI070849 |
| 458 | UI-R-C2-nf-a-09-0-UI | ATPase, Na+/K+ transporting, alpha 1 polypeptide | AI072060 |
| 459 | UI-R-C1-kh-e-09-0-UI | NIMA (never in mitosis gene a)- related kinase 9 | AI045249 |
| 460 | UI-R-DK0-cfz-c-02-0-UI | Sirtuin 3 (silent mating type information regulation 2, homolog) 3 | BI296779 |
| 461 | UI-R-DK0-cgf-d-03-0-UI | Phosphoinositide-3-kinase, regulatory subunit 4, p150 | BI291091 |
| 462 | UI-R-CV2-cgq-d-07-0-UI | Replication factor C (activator 1) 4 | BI291451 |
| 463 | UI-R-CV2-chc-f-12-0-UI | Acetyl-Coenzyme A acyltransferase 1 | BI297474 |
| 464 | UI-R-DR0-cjc-m-24-0-UI | Transcribed locus | BI304063 |
| 465 | UI-R-A1-do-e-08-0-UI | Dynein, cytoplasmic, light chain 2A | AA933176 |
| 466 | UI-R-A1-dt-f-01-0-UI | Cell division cycle 25 homolog B | BF558790 |
| 467 | UI-R-A1-dw-g-06-0-UI | Protein phosphatase 1, catalytic subunit, beta isoform | AA901261 |
| 468 | UI-R-E1-gk-a-06-0-UI | Endothelin receptor type B | AA963856 |
| 469 | UI-R-C0-gu-g-02-0-UI | Phospholipase A2, group VI | BF557280 |
| 470 | UI-R-DC0-bzm-f-02-0-UI | Histocompatibility 13 | BI280621 |
| 471 | UI-R-DD0-caa-e-10-0-UI | Extracellular matrix protein 1 | BI285473 |
| 472 | UI-R-CW0s-cck-b-08-0-UI | Prostate tumor over expressed gene 1 | BI287647 |
| 473 | UI-R-CX0s-ccr-b-03-0-UI | Solute carrier family 7 (cationic amino acid transporter, y+ system), member 8 | BI284439 |
| 474 | UI-R-CX0s-ccx-g-03-0-UI | Prolactin family 8, subfamily a, member 9 | BI283877 |
| 475 | UI-R-DK0-cde-h-02-0-UI | Uncharacterized protein family UPF0227 member RGD1359682 | BI288782 |
| 476 | UI-R-DK0-cev-g-01-0-UI | Solute carrier family 30 (zinc transporter), member 3 | BI295552 |
| 477 | UI-R-DK0-cfc-f-12-0-UI | O-acyltransferase (membrane bound) domain containing 5 | BI295955 |
| 478 | UI-R-DK0-cfg-c-10-0-UI | Thymidine kinase 1 | BI289385 |
| 479 | UI-R-CA1-bjt-e-09-0-UI | Checkpoint suppressor 1 | BF410102 |
| 480 | UI-R-BT1-bmq-a-05-0-UI | SRY-box containing gene 10 | BF411714 |
| 481 | UI-R-BT1-bnk-d-01-0-UI | Luteinizing hormone beta | BG379148 |
| 482 | UI-R-CA0-bod-d-06-0-UI | Zinc finger protein 644 | BF413075 |
| 483 | UI-R-BJ2-boo-d-11-0-UI | Cysteine conjugate-beta lyase 1 | BF414271 |
| 484 | UI-R-BJ2-bqm-f-01-0-UI | Guanine nucleotide binding protein-like 1 | CK843742 |
| 485 | UI-R-BJ2-bqq-e-11-0-UI | RDCR-0918-3 protein | BF419351 |
| 486 | UI-R-CW0-bvz-g-04-0-UI | Thiosulfate sulfurtransferase | BI273854 |
| 487 | UI-R-CW0-bwl-a-12-0-UI | H1 histone family, member 0 | BI274545 |
| 488 | UI-R-DA0-byj-g-03-0-UI | Amylase 1, salivary | BI279142 |
| 489 | UI-R-DB0-byv-b-09-0-UI | NADH dehydrogenase (ubiquinone) 1 beta subcomplex 8 | BI285010 |
| 490 | UI-R-CA0-bge-g-02-0-UI | axonemal dynein light chain 1 | BF394767 |
| 491 | UI-R-CA0-bgr-h-05-0-UI | Dual specificity phosphatase-like 15 | BF394353 |
| 492 | UI-R-CA1-bjb-m-08-0-UI | Carnitine acetyltransferase | BF399378 |
| 493 | UI-R-CA1-bje-p-06-0-UI | Acyltransferase like 2 | BF406111 |
| 494 | UI-R-CV0-brm-a-04-0-UI | Aldehyde dehydrogenase family 3, member A1 | BG372072 |
| 495 | UI-R-CV1-bsa-c-10-0-UI | Growth differentiation factor 11 | BG372830 |
| 496 | UI-R-CT0-buk-f-02-0-UI | Lix1 homolog like | BG381551 |
| 497 | UI-R-CU0-bus-f-07-0-UI | Armadillo repeat containing 5 | BG376279 |
| 498 | UI-R-CU0-bvd-h-04-0-UI | Acyl-Coenzyme A binding domain containing 6 | BG377530 |
| 499 | UI-R-CT0-bvn-a-05-0-UI | neuropathy target esterase homolog | BG378029 |
| 500 | UI-R-CA0-axg-a-09-0-UI | Nischarin | BE108587 |
| 501 | UI-R-CA0-axs-d-12-0-UI | Hyaluronic acid binding protein 4 | BE115750 |
| 502 | UI-R-BS1-ayi-g-11-0-UI | Phosphatidylinositol 3-kinase, catalytic, alpha polypeptide | BE117439 |
| 503 | UI-R-BS1-ayt-f-07-0-UI | Protein inhibitor of activated STAT 1 | CK845413 |
| 504 | UI-R-CA0-bax-b-04-0-UI | Transformation/transcription domain-associated protein | BE121370 |
| 505 | UI-R-CA1-bbi-b-09-0-UI | KIAA0528 protein | BF386322 |
| 506 | UI-R-CA1-bbz-c-04-0-UI | DD1 | BF388266 |
| 507 | UI-R-CA1-bci-d-12-0-UI | Hbs1-like | BF390872 |
| 508 | UI-R-BS2-bes-c-10-0-UI | Kelch-like 5 | BF398775 |
| 509 | UI-R-CA0-bfl-g-06-0-UI | Nuclear receptor binding SET domain protein 1 | BF392695 |
| 510 | UI-R-BS0-aoe-f-10-0-UI | Solute carrier family 37 (glycerol-3-phosphate transporter), member 3 | AW535395 |
| 511 | UI-R-BU0-ape-d-03-0-UI | Fras1 related extracellular matrix protein 1 | BE096295 |
| 512 | UI-R-BX0-asb-a-08-0-UI | Transcribed locus | BE105229 |
| 513 | UI-R-BO1-asm-c-10-0-UI | Leupaxin | BE106297 |
| 514 | UI-R-BJ1-asx-b-02-0-UI | Protein phosphatase 2, regulatory subunit B (B56), delta isoform | CK844834 |
| 515 | UI-R-BJ1-atg-h-01-0-UI | Hypothetical LOC302495 | CK842860 |
| 516 | UI-R-BJ1-aut-g-04-0-UI | Seven in absentia 1A | CK839809 |
| 517 | UI-R-BJ1-avg-c-09-0-UI | Transmembrane protein 53 | CK844129 |
| 518 | UI-R-BJ1-avp-g-04-0-UI | Thyroid hormone receptor beta | BE109577 |
| 519 | UI-R-BJ1-avw-b-05-0-UI | Sulfatase 1 | BE115543 |
| 520 | UI-R-BO0-aib-b-12-0-UI | zinc finger protein 709 | BF567496 |
| 521 | UI-R-BO0-aii-a-01-0-UI | Splicing factor 3a, subunit 3 | AW524140 |
| 522 | UI-R-BJ0p-aiq-a-12-0-UI | XP_001075705.1 NFkB interacting protein 1 | BF562839 |
| 523 | UI-R-BJ0p-aiv-f-07-0-UI | Cappuccino homolog | CK842060 |
| 524 | UI-R-BT1-ajy-h-08-0-UI | Excision repair cross-complementing rodent repair deficiency, complementation group 3 | AW527800 |
| 525 | UI-R-BT1-akg-e-12-0-UI | spermatogenesis associated 11 | AW528002 |
| 526 | UI-R-BT1-akm-f-08-0-UI | Down syndrome cell adhesion molecule | BF565509 |
| 527 | UI-R-BT1-akv-d-12-0-UI | Adenylate cyclase 9 | BF563915 |
| 528 | UI-R-BU0-amx-b-10-0-UI | Four jointed box 1 | BF564728 |
| 529 | UI-R-BU0-and-g-12-0-UI | Transcribed locus | AW533599 |
| 530 | UI-R-AB1-yv-c-08-0-UI | Fucosidase, alpha-L- 1, tissue | CK845101 |
| 531 | UI-R-AE1-zc-d-03-0-UI | XP_001083368.1 proteasome beta 7 subunit | AI711201 |
| 532 | UI-R-Y0-aaz-a-02-0-UI | Methyltransferase like 7A | AI713156 |
| 533 | UI-R-Y0-abj-h-06-0-UI | Tumor-associated calcium signal transducer 2 | AI764478 |
| 534 | UI-R-Y0-abv-g-08-0-UI | Vasoactive intestinal peptide receptor 2 | AI764593 |
| 535 | UI-R-Y0-ace-b-11-0-UI | Homeodomain interacting protein kinase 1 | AI764711 |
| 536 | UI-R-BJ0p-afe-b-04-0-UI | Calcium binding protein p22 | BF566710 |
| 537 | UI-R-BJ0p-afl-e-08-0-UI | Zinc finger protein 532 | BF566783 |
| 538 | UI-R-BJ0p-aft-b-12-0-UI | Polymerase (DNA directed), alpha 2 | CK841631 |
| 539 | UI-R-BJ0p-agb-g-06-0-UI | Tripartite motif protein 11 | BF566999 |
| 540 | UI-R-C2p-rp-b-12-0-UI | CD2 antigen | AI556893 |
| 541 | UI-R-C2p-rw-g-05-0-UI | Mkrn1 protein | BF545165 |
| 542 | UI-R-C2p-se-e-05-0-UI | Monoglyceride lipase | BF542323 |
| 543 | UI-R-C3-so-a-12-0-UI | DnaJ (Hsp40) related, subfamily B, member 13 | BF542711 |
| 544 | UI-R-G0-ue-e-11-0-UI | XP_001085183.1 Exocyst complex component 1 (Exocyst complex component Sec3) | BF523322 |
| 545 | UI-R-G0-uq-d-01-0-UI | Thiamine triphosphatase | AI576526 |
| 546 | UI-R-Y0-uz-d-09-0-UI | Transcribed locus | AI575941 |
| 547 | UI-R-Y0-vm-c-04-0-UI | Leucine carboxyl methyltransferase 1 | AI576392 |
| 548 | UI-R-AF0-yc-d-09-0-UI | Regulator of G-protein signaling 19 | BF525145 |
| 549 | UI-R-AC0-ym-e-01-0-UI | Glutamate receptor, ionotropic, N-methyl D-aspartate-like 1A | CK838877 |
| 550 | UI-R-E1-gc-a-03-0-UI | Protein tyrosine phosphatase-like (proline instead of catalytic arginine), member b | AA957716 |
| 551 | UI-R-E1-gl-b-09-0-UI | Tektin 3 | BF559884 |
| 552 | UI-R-C0-jo-f-09-0-UI | XP_001118656.1 CTCL tumor antigen L14-2, partial | AI030707 |
| 553 | UI-R-C1-ki-b-02-0-UI | Ext1 | AI044805 |
| 554 | UI-R-C1-lb-g-10-0-UI | Cystatin F (leukocystatin) | BF523613 |
| 555 | UI-R-C1-lr-d-11-0-UI | Kinesin family member 7 | BF545517 |
| 556 | UI-R-C2p-oj-h-07-0-UI | Yip1 domain family, member 4 | AI137232 |
| 557 | UI-R-BT0-pq-e-10-0-UI | Thymus cell antigen 1, theta | AI145313 |
| 558 | UI-R-BT0-pv-e-03-0-UI | Cc1-9 | AI145205 |
| 559 | UI-R-BT0-qf-f-05-0-UI | Zinc finger homeobox 2 | BF545911 |
| 560 | UI-R-A0-at-c-09-0-UI | Camello-like 2 | BF549681 |
| 561 | UI-R-A0-bd-c-09-0-UI | Thioredoxin domain containing 4 (endoplasmic reticulum) | BF555382 |
| 562 | UI-R-E0-bo-h-11-0-UI | Suppressor of variegation 3-9 homolog 1 | AA858468 |
| 563 | UI-R-E0-bu-a-04-0-UI | Prolactin family 3, subfamily c, member 1 | AA875114 |
| 564 | UI-R-E0-cz-h-10-0-UI | CCR4-NOT transcription complex, subunit 8 | AA899265 |
| 565 | UI-R-E0-dg-e-02-0-UI | Glutaredoxin 1 (thioltransferase) | AA899621 |
| 566 | UI-R-A1-do-e-11-0-UI | Membrane interacting protein of RGS16 | AA901006 |
| 567 | UI-R-A1-ds-h-08-0-UI | CKLF-like MARVEL transmembrane domain containing 8 | BF555897 |
| 568 | UI-R-A1-ew-e-02-0-UI | Replication protein A3 | AA955320 |
| 569 | UI-R-E1-fi-d-09-0-UI | Transcribed locus | AA956228 |
| 570 | UI-R-CA0-bkv-f-11-0-UI | C-type (calcium dependent, carbohydrate recognition domain) lectin, superfamily member 6 | BF416842 |
| 571 | UI-R-DK0-cdq-a-04-0-UI | RIB43A domain with coiled-coils 1 | BI293404 |
| 572 | UI-R-E1-gh-h-07-0-UI | Keratin 10 | AA963283 |
| 573 | UI-R-C0-hr-g-12-0-UI | Alcohol dehydrogenase PAN2 | AA997435 |
| 574 | UI-R-C0-jf-a-02-0-UI | Mitochondrial ribosomal protein L46 | BF523542 |
| 575 | UI-R-C1-kx-g-05-0-UI | Sialic acid binding Ig-like lectin 10 | BF546380 |
| 576 | UI-R-C2p-rg-b-11-0-UI | hypothetical protein FLJ32825 | BF544928 |
| 577 | UI-R-C2p-rv-e-03-0-UI | Transcribed locus | BF522944 |
| 578 | UI-R-C0-ir-h-06-0-UI | Myotubularin related protein 1 | AI029466 |
| 579 | UI-R-C0-jb-f-01-0-UI | Transcribed locus | AI030300 |
| 580 | UI-R-C1-km-f-05-0-UI | Cytidine monophospho-N-acetylneuraminic acid synthetase | AI045944 |
| 581 | UI-R-C1-kv-a-02-0-UI | protein phosphatase 1, regulatory (inhibitory) subunit 1C; thymocyte ARPP; DNA segment, Chr 9, Brigham & Womens Genetics 1012 expressed | AI058489 |
| 582 | UI-R-C1-lk-e-05-0-UI | Retinoblastoma binding protein 5 | BF559895 |
| 583 | UI-R-C1-lq-d-12-0-UI | Fanconi anemia, complementation group A | AI059004 |
| 584 | UI-R-C0-hh-c-02-0-UI | Serine/threonine kinase 6 | AA996882 |
| 585 | UI-R-C0-je-c-08-0-UI | Neural stem cell-derived dendrite regulator | AI030989 |
| 586 | UI-R-C1-kw-b-03-0-UI | Proprotein convertase subtilisin/kexin type 9 | AI059511 |
| 587 | UI-R-C2-mx-d-02-0-UI | Pam, highwire, rpm 1 | AI070678 |
| 588 | UI-R-C0-ii-a-12-0-UI | Nasal embryonic LHRH factor | BF551055 |
| 589 | UI-R-C0-je-f-10-0-UI | Fanconi anemia D2 protein | AI031007 |
| 590 | UI-R-C1-km-a-08-0-UI | Kinesin family member C2 | AI045836 |
| 591 | UI-R-Y0-ls-g-03-0-UI | Ectonucleoside triphosphate diphosphohydrolase 3 | AI070096 |
| 592 | UI-R-E1-fa-g-04-0-UI | Eukaryotic translation initiation factor 1A | AA955540 |
| 593 | UI-R-E1-fr-h-06-0-UI | Ubiquitin specific peptidase 3 | AA956794 |
| 594 | UI-R-E1-fx-b-12-0-UI | Diablo homolog | AA963090 |
| 595 | UI-R-E1-gk-c-07-0-UI | proliferation associated nuclear element 1 isoform 1 | AA963877 |
| 596 | UI-R-C0-ii-a-04-0-UI | Transcribed locus | AA998806 |
| 597 | UI-R-C0-in-d-09-0-UI | Tensin 4 | AA999047 |
| 598 | UI-R-A1-es-h-12-0-UI | Prolactin family 8, subfamily a, member 2 | BF558397 |
| 599 | UI-R-A1-ey-h-04-0-UI | Cytochrome b-245, alpha polypeptide | AA955072 |
| 600 | UI-R-C0-ip-c-07-0-UI | Insulin 2 | BF551436 |
| 601 | UI-R-Y0-lv-c-11-0-UI | Fibromodulin | AI070507 |
| 602 | UI-R-BT0-pt-e-04-0-UI | Eukaryotic translation initiation factor 2 alpha kinase 1 | AI145068 |
| 603 | UI-R-A0-az-b-01-0-UI | UDP-Gal:betaGlcNAc beta 1,4- galactosyltransferase, polypeptide 1 | BF549771 |
| 604 | UI-R-C1-ln-h-11-0-UI | KIAA1128 protein | AI070061 |
| 605 | UI-R-A0-ay-b-02-0-UI | Lymphocyte antigen 6 complex, locus B | AA818756 |
| 606 | UI-R-E0-ch-d-08-0-UI | Transcribed locus | AA900546 |
| 607 | UI-R-A1-ew-f-06-0-UI | Protein kinase C-binding protein Beta15 | AA955328 |
| 608 | UI-R-C2-ng-b-09-0-UI | XP_001103658.1 zinc finger and BTB domain containing 8 opposite strand isoform 2 | AI072313 |
| 609 | UI-R-C2-ni-e-08-0-UI | Ribosomal protein L11 | AI072543 |
| 610 | UI-R-C2-nk-f-01-0-UI | Solute carrier family 35 (CMP-sialic acid transporter), member 1 | AI072449 |
| 611 | UI-R-C2-nm-g-12-0-UI | Pleckstrin homology domain containing, family G (with RhoGef domain) member 2 | BF552845 |
| 612 | UI-R-A0-ak-h-08-0-UI | Guanine nucleotide binding protein (G protein), beta polypeptide 2 like 1 | AA859083 |
| 613 | UI-R-A0-ax-a-05-0-UI | Polypyrimidine tract binding protein 1 | AA818247 |
| 614 | UI-R-A0-be-b-10-0-UI | Acyl-CoA synthetase long-chain family member 4 | AA858723 |
| 615 | UI-R-A0-bl-g-08-0-UI | Protein tyrosine phosphatase 4a2 | BF547880 |
| 616 | UI-R-E0-cu-f-09-0-UI | RAB14, member RAS oncogene family | AA875211 |
| 617 | UI-R-E0-de-a-07-0-UI | Procollagen, type II, alpha 1 | AA899463 |
| 618 | UI-R-C0-ji-b-08-0-UI | Coiled-coil domain containing 92 | BF554604 |
| 619 | UI-R-C0-jl-e-09-0-UI | hypothetical protein FLJ21827 | AI043648 |
| 620 | UI-R-C1-ke-d-03-0-UI | Oxidative stress responsive gene | AI044947 |
| 621 | UI-R-C1-kh-d-07-0-UI | Dynactin 2 | BF554335 |
| 622 | UI-R-C1-kj-g-04-0-UI | UDP-N-acetyl-alpha-D-galactosamine:polypeptide N-acetylgalactosaminyltransferase 1 | BF547008 |
| 623 | UI-R-C1-kp-c-03-0-UI | chromosome 10 open reading frame 18 | AI058731 |
| 624 | UI-R-C1-ld-f-06-0-UI | Zinc finger protein 313 | AI059254 |
| 625 | UI-R-C1-lg-a-05-0-UI | Protein kinase C, eta | CK840727 |
| 626 | UI-R-C1-lp-c-06-0-UI | Roundabout homolog 2 | AI069991 |
| 627 | UI-R-Y0-lv-d-11-0-UI | Valyl-tRNA synthetase 2 | AI070511 |
| 628 | UI-R-A1-ez-f-11-0-UI | P7 protein | AA955094 |
| 629 | UI-R-E1-ff-g-05-0-UI | e(y)2 protein | BF560152 |
| 630 | UI-R-E1-fk-d-05-0-UI | Chromosome 6 open reading frame 4 | BF556487 |
| 631 | UI-R-E1-fm-h-02-0-UI | WD repeat and FYVE domain containing 1 | AA956708 |
| 632 | UI-R-C0-hd-b-05-0-UI | Polymerase (RNA) I associated factor 1 | AA965258 |
| 633 | UI-R-C0-hh-e-06-0-UI | RAB34, member of RAS oncogene family | AA996775 |
| 634 | UI-R-C0-ht-h-01-0-UI | DNA segment, Chr 16, ERATO Doi 472, expressed | AI137629 |
| 635 | UI-R-C0-hv-g-04-0-UI | Dehydrogenase/reductase (SDR family) member 4 | AA997825 |
| 636 | UI-R-C0-iu-h-01-0-UI | Ubiquitin D | AI030354 |
| 637 | UI-R-C0-ja-e-04-0-UI | NOL1/NOP2/Sun domain family, member 5 | AI030029 |
| 638 | UI-R-C0-hw-e-08-0-UI | Casein alpha s1 | BF561522 |
| 639 | UI-R-C0-jd-b-05-0-UI | Forkhead box Q1 | AI030728 |
| 640 | UI-R-C1-kl-f-10-0-UI | Low density lipoprotein receptor-related protein 2 | AI058308 |
| 641 | UI-R-C1-kv-b-10-0-UI | Mevalonate (diphospho) decarboxylase | BF559092 |
| 642 | UI-R-C1-la-d-01-0-UI | Vacuolar protein sorting 33B | AI059963 |
| 643 | UI-R-C1-li-f-11-0-UI | Hepsin | BF549566 |
| 644 | UI-R-C2p-ob-c-08-0-UI | Signal sequence receptor 4 | AI136606 |
| 645 | UI-R-C2p-om-g-09-0-UI | Islet amyloid polypeptide | AI138126 |
| 646 | UI-R-A0-ac-g-02-0-UI | Lectin, galactose binding, soluble 1 | BF548668 |
| 647 | UI-R-A0-ah-h-09-0-UI | RIKEN cDNA 2310016C16 | AA818239 |
| 648 | UI-R-CV2-cif-b-12-0-UI | mKIAA0195 protein | BI300457 |
| 649 | UI-R-DN0-cit-l-18-0-UI | Coxsackie virus and adenovirus receptor-like 1 | BI291832 |
| 650 | UI-R-DN0-civ-o-05-0-UI | Cd86 antigen | BI292219 |
| 651 | UI-R-DQ0-ciz-g-10-0-UI | Expressed in non-metastatic cells 6, protein (nucleoside diphosphate kinase) | BI303181 |
| 652 | UI-R-A1-eb-a-07-0-UI | DnaJ (Hsp40) homolog, subfamily A, member 2 | BF559183 |
| 653 | UI-R-A1-ee-d-05-0-UI | Spondin 1 | AA925370 |
| 654 | UI-R-A1-ei-b-03-0-UI | Platelet derived growth factor receptor, alpha polypeptide | AA925099 |
| 655 | UI-R-A1-eo-h-06-0-UI | Phosphate cytidylyltransferase 1, choline, alpha isoform | AA925887 |
| 656 | UI-R-C0-hl-g-08-0-UI | Nudix (nucleoside diphosphate linked moiety X)-type motif 6 | AA997476 |
| 657 | UI-R-C0-hs-f-05-0-UI | Casein kinase 2, beta subunit | BF550675 |
| 658 | UI-R-CU0s-cbx-b-03-0-UI | Transcribed locus | BI282144 |
| 659 | UI-R-CW0s-ccb-h-04-0-UI | HtrA serine peptidase 1 | BI285585 |
| 660 | UI-R-DK0-cdq-h-03-0-UI | Ribosomal protein L3-like | BI293470 |
| 661 | UI-R-DK0-ceb-a-04-0-UI | Component of oligomeric golgi complex 1 | BI294695 |
| 662 | UI-R-DK0-cee-a-06-0-UI | Ectonucleoside triphosphate diphosphohydrolase 4 | BI294951 |
| 663 | UI-R-DK0-ceg-f-11-0-UI | NEL-like 1 (chicken) | BI295178 |
| 664 | UI-R-BJ2-bqw-e-06-0-UI | EGL nine homolog 1 | BF407173 |
| 665 | UI-R-BJ2-brd-b-01-0-UI | Limbic system-associated membrane protein | CK843444 |
| 666 | UI-R-DK0-cfi-c-11-0-UI | Ras homolog gene family, member J | BI289636 |
| 667 | UI-R-DK0-cfs-b-07-0-UI | Calcyclin binding protein | BI290117 |
| 668 | UI-R-BJ2-boy-f-08-0-UI | Perlecan | BF415013 |
| 669 | UI-R-BJ2-bpl-b-04-0-UI | DNA fragmentation factor, alpha subunit | CK844626 |
| 670 | UI-R-BJ2-bpt-c-12-0-UI | Rap2 interacting protein | CK843182 |
| 671 | UI-R-BJ2-bqh-c-03-0-UI | Amyotrophic lateral sclerosis 2 (juvenile) chromosome region, candidate 4 | BF418549 |
| 672 | UI-R-CX0-bwx-f-04-0-UI | Solute carrier family 6 (neurotransmitter transporter, betaine/GABA), member 12 | BI278848 |
| 673 | UI-R-CX0-bxi-e-08-0-UI | NP_085911.2 (Asp-Glu-Ala-Asp/His) box polypeptide 11 isoform 1 | BI275964 |
| 674 | UI-R-CY0-bxp-b-10-0-UI | Sorting and assembly machinery component 50 homolog | BI277013 |
| 675 | UI-R-CZ0-byc-a-11-0-UI | Pleiotropic regulator 1, PRL1 homolog | BI278003 |
| 676 | UI-R-CT0s-cas-c-09-0-UI | DnaJ (Hsp40) homolog, subfamily C, member 7 | BI285682 |
| 677 | UI-R-CU0s-cbp-g-10-0-UI | XP_001116473.1 40S ribosomal protein S26 | BI281341 |
| 678 | UI-R-CA1-bih-h-07-0-UI | Formin binding protein 4 | BF404640 |
| 679 | UI-R-CA1-bix-g-24-0-UI | Von Ebners gland protein 2 | BF405590 |
| 680 | UI-R-CV1-bsl-c-01-0-UI | Deoxythymidylate kinase | BG373993 |
| 681 | UI-R-CV1-bta-b-02-0-UI | Plasma membrane proteolipid | BG374621 |
| 682 | UI-R-CS0-btt-d-08-0-UI | SAR1 gene homolog A | BG381368 |
| 683 | UI-R-CT0-btz-h-01-0-UI | Protein tyrosine phosphatase, receptor type, H | BG380654 |
| 684 | UI-R-CA1-bjr-h-12-0-UI | RAB36, member RAS oncogene family | BF409886 |
| 685 | UI-R-CA0-bkq-f-07-0-UI | Suppressor of cytokine signaling 4 | BF416136 |
| 686 | UI-R-CN0-bld-d-12-0-UI | Translocator of inner mitochondrial membrane 17a | BF417375 |
| 687 | UI-R-CN0-blt-h-08-0-UI | Hermansky-Pudlak syndrome 5 | BF418212 |
| 688 | UI-R-BS1-azb-c-02-0-UI | Topoisomerase (DNA) 2 alpha | BE109123 |
| 689 | UI-R-BJ1-azo-c-07-0-UI | NADH dehydrogenase (ubiquinone) 1 alpha subcomplex, 12 | BE118192 |
| 690 | UI-R-CA0-bab-d-12-0-UI | General transcription factor II E, polypeptide 1 (alpha subunit) | BE118764 |
| 691 | UI-R-CA0-bao-g-06-0-UI | Sparc/osteonectin, cwcv and kazal-like domains proteoglycan 2 | BE120030 |
| 692 | UI-R-CA1-bcx-h-08-0-UI | DEAD/H (Asp-Glu-Ala-Asp/His) box polypeptide 3, X-linked | BF391513 |
| 693 | UI-R-BS2-bdk-f-12-0-UI | Adaptor-related protein complex 3, mu 2 subunit | BF389005 |
| 694 | UI-R-BS2-bdw-f-04-0-UI | Wolf-Hirschhorn syndrome candidate 1-like 1 | BF396304 |
| 695 | UI-R-BS2-bed-e-02-0-UI | Aryl hydrocarbon receptor nuclear translocator | BF397687 |
| 696 | UI-R-CA0-bhd-a-11-0-UI | Mitochondrial ribosomal protein L17 | BF400581 |
| 697 | UI-R-CA0-bhr-d-12-0-UI | Coiled-coil-helix-coiled-coil-helix domain containing 3 | BF402528 |
| 698 | UI-R-BT1-aqo-c-06-0-UI | Transformation related protein 53 binding protein 2 | BE102556 |
| 699 | UI-R-BX0-arf-d-10-0-UI | Aurora kinase C | BE103696 |
| 700 | UI-R-BJ1-ato-g-02-0-UI | Inhibin alpha | BE099377 |
| 701 | UI-R-BJ1-att-b-01-0-UI | Prolactin regulatory element binding | BE100313 |
| 702 | UI-R-BJ1-aua-h-12-0-UI | Transcribed locus | CK839441 |
| 703 | UI-R-BJ1-aui-e-08-0-UI | Transcribed locus | BE101310 |
| 704 | UI-R-BJ1-awa-h-01-0-UI | Solute carrier family 16 (monocarboxylic acid transporters), member 3 | BE112961 |
| 705 | UI-R-BJ1-awg-d-08-0-UI | Odd Oz/ten-m homolog 3 | BE113619 |
| 706 | UI-R-BJ1-awq-b-03-0-UI | Fibroblast growth factor 1 | CK840006 |
| 707 | UI-R-CA0-awy-h-11-0-UI | Solute carrier family 8 (sodium/calcium exchanger), member 1 | BE107974 |
| 708 | UI-R-BO1-ajc-b-10-0-UI | Synuclein, beta | AW526495 |
| 709 | UI-R-BO1-aji-e-05-0-UI | Synaptotagmin XI | BF563293 |
| 710 | UI-R-BO1-ajm-e-02-0-UI | Zinc finger protein 330 | AW527185 |
| 711 | UI-R-BO1-ajs-g-11-0-UI | Transcribed locus | AW527309 |
| 712 | UI-R-C4-ald-f-03-0-UI | Mitogen-activated protein kinase 8 interacting protein 2 | AW530104 |
| 713 | UI-R-C4-all-g-08-0-UI | Sno, strawberry notch homolog 1 | AW532300 |
| 714 | UI-R-C4-als-g-04-0-UI | Myosin VC | AW535147 |
| 715 | UI-R-BT1-amg-c-07-0-UI | SUMO/sentrin specific protease family member 8 | BE095577 |
| 716 | UI-R-BO1-apo-f-06-0-UI | Cytochrome c oxidase, subunit VIIa 2 | BE095655 |
| 717 | UI-R-BO1-aqc-f-05-0-UI | Cyclin-dependent kinase 5, regulatory subunit 1 (p35) | BE097854 |
| 718 | UI-R-AA1-aae-c-01-0-UI | Polymerase (DNA-directed), delta interacting protein 2 | BF543017 |
| 719 | UI-R-AF1-aas-d-04-0-UI | small unique nuclear receptor co-repressor | AI712600 |
| 720 | UI-R-Y0-aco-d-04-0-UI | Calcium channel, voltage-dependent, alpha 1F subunit | AI717377 |
| 721 | UI-R-BJ0-acz-b-01-0-UI | Signal recognition particle 19 | AW253762 |
| 722 | UI-R-BJ0-adt-e-07-0-UI | Centaurin, alpha 1 | AW252246 |
| 723 | UI-R-BJ0-aen-b-06-0-UI | High mobility group nucleosomal binding domain 3 | CK841128 |
| 724 | UI-R-BO0-agk-a-06-0-UI | DnaJ (Hsp40) homolog, subfamily B, member 1 | AW521162 |
| 725 | UI-R-BO0-agr-e-06-0-UI | Diacylglycerol kinase, gamma | AW521765 |
| 726 | UI-R-BO0-ahl-e-07-0-UI | mKIAA0934 protein | BF567787 |
| 727 | UI-R-BO0-ahu-d-06-0-UI | phosphatidylinositol-specific phospholipase C, X domain containing 2 | AW523551 |
| 728 | UI-R-C3-sw-b-09-0-UI | tubulin tyrosine ligase-like family, member 4 | BF543618 |
| 729 | UI-R-C3-ti-d-08-0-UI | Transcribed locus | AI548256 |
| 730 | UI-R-C3-tn-e-04-0-UI | lipin 3 | AI709541 |
| 731 | UI-R-C3-tw-c-01-0-UI | UDP-N-acetyl-alpha-D-galactosamine:polypeptide N-acetylgalactosaminyltransferase 2 | AI548701 |
| 732 | UI-R-AD0-wc-d-01-0-UI | RIKEN cDNA 0610007P22 | BF525061 |
| 733 | UI-R-AA0-wp-c-09-0-UI | Calmodulin 2 | CK840427 |
| 734 | UI-R-AG0-wy-c-07-0-UI | Brain expressed X-linked 1 | AI579422 |
| 735 | UI-R-AE0-xm-g-04-0-UI | Leprecan 1 | BF524639 |
| 736 | UI-R-AD1-zo-b-02-0-UI | Alanyl-tRNA synthetase | CK839707 |
| 737 | UI-R-AD1-zu-a-11-0-UI | scaffolding protein SLIPR | CK841057 |
| 738 | UI-R-C0-hx-b-12-0-UI | Sulfotransferase family, cytosolic, 2B, member 1 | BF561552 |
| 739 | UI-R-C0-ir-f-10-0-UI | Solute carrier family 2 (facilitated glucose transporter), member 3 | AI029371 |
| 740 | UI-R-Y0-mk-e-08-0-UI | Protein phosphatase 3, catalytic subunit, gamma isoform | AI111664 |
| 741 | UI-R-C2-mu-g-09-0-UI | Protein (peptidyl-prolyl cis/trans isomerase) NIMA-interacting 1 | AI385373 |
| 742 | UI-R-C2p-nr-f-10-0-UI | Sestrin 3 | AI113325 |
| 743 | UI-R-C2p-od-f-05-0-UI | Acyl-Coenzyme A binding domain containing 4 | AI136400 |
| 744 | UI-R-BT0-qk-g-07-0-UI | Tubulin tyrosine ligase | BF544712 |
| 745 | UI-R-C2p-qq-e-03-0-UI | RIKEN cDNA 4921531G14 | AI555299 |
| 746 | UI-R-C2p-qw-a-06-0-UI | Heat shock 70kDa protein 4-like | AI555324 |
| 747 | UI-R-C2p-rg-h-05-0-UI | Centaurin, gamma 1 | BF544947 |
| 748 | UI-R-E0-by-a-07-0-UI | Geminin | BF549976 |
| 749 | UI-R-E0-ce-b-11-0-UI | Paired-like homeobox 2a | AA875274 |
| 750 | UI-R-E0-ck-h-02-0-UI | Lymphocyte antigen 86 | AA874924 |
| 751 | UI-R-E0-ct-d-12-0-UI | Hypothetical LOC293114 | AA875518 |
| 752 | UI-R-A1-dx-f-09-0-UI | Ubiquitin-conjugating enzyme E2A, RAD6 homolog | BF559553 |
| 753 | UI-R-A1-ec-d-10-0-UI | Thioredoxin-like 1 | AA925446 |
| 754 | UI-R-A1-eh-e-04-0-UI | Dolichol-phosphate (beta-D) mannosyltransferase 1 | AA925213 |
| 755 | UI-R-A1-el-h-06-0-UI | Suppression of tumorigenicity 7 | AA926326 |
| 756 | UI-R-C0-ha-f-12-0-UI | Pleckstrin homology-like domain, family A, member 1 | AA965045 |
| 757 | UI-R-C0-hq-a-02-0-UI | Atpase, class I, type 8B, member 2 | AA997117 |
| 758 | UI-R-A0-ao-f-08-0-UI | Glycine-N-acyltransferase | AA818998 |
| 759 | UI-R-AD0-vz-b-05-0-UI | Methyltransferase like 6 | BF525046 |
| 760 | UI-R-BJ0p-aik-e-07-0-UI | Serum response factor binding protein 1 | CK843952 |
| 761 | UI-R-BJ1-awu-d-12-0-UI | Glycosyltransferase 8 domain containing 1 | BE115033 |
| 762 | UI-R-A0-bl-g-09-0-UI | Carboxypeptidase A6 | AA819425 |
| 763 | UI-R-E0-ck-f-10-0-UI | Phosphatidylinositol glycan, class C | AA874910 |
| 764 | UI-R-A1-dr-g-02-0-UI | Procollagen, type XV | BF558723 |
| 765 | UI-R-A1-el-e-10-0-UI | Serine dehydratase-like | BF521655 |
| 766 | UI-R-Y0-lx-g-06-0-UI | c-myc promoter binding protein | AI073077 |
| 767 | UI-R-C2p-nv-f-04-0-UI | Carbonic anhydrase 14 | CK840259 |
| 768 | UI-R-C0-ha-h-06-0-UI | SWI/SNF related, matrix associated, actin dependent regulator of chromatin, subfamily c, member 1 | AA965063 |
| 769 | UI-R-C0-hg-d-08-0-UI | Protein tyrosine phosphatase, receptor type, B | BF561204 |
| 770 | UI-R-C0-jd-d-03-0-UI | Heparan sulfate 2-O-sulfotransferase 1 | AI030737 |
| 771 | UI-R-C0-jm-g-02-0-UI | Chemokine (C-X-C motif) ligand 14 | AI043862 |
| 772 | UI-R-C0-jr-g-05-0-UI | Gelsolin | AI030084 |
| 773 | UI-R-C1-jv-a-11-0-UI | F-box protein FBL2 | BF551173 |
| 774 | UI-R-A0-av-g-07-0-UI | Cytochrome P450, family 2, subfamily c, polypeptide 23 | BF544884 |
| 775 | UI-R-C0-hb-a-10-0-UI | 3-alpha-hydroxysteroid dehydrogenase | AA964948 |
| 776 | UI-R-A0-ac-h-02-0-UI | Dynein, axonemal, light chain 4 | AA866309 |
| 777 | UI-R-A0-ax-f-07-0-UI | NAD(P) dependent steroid dehydrogenase-like | BF549711 |
| 778 | UI-R-E1-fk-e-02-0-UI | Myeloid/lymphoid or mixed-lineage leukemia 3 | AA956478 |
| 779 | UI-R-Y0-lv-d-12-0-UI | Neurexin 2 | BF521761 |
| 780 | UI-R-A1-es-a-04-0-UI | Transcribed locus |  |
| 781 | UI-R-E1-fx-h-07-0-UI | Transmembrane protein 34 | AA962979 |
| 782 | UI-R-C2-mx-e-01-0-UI | Synaptobrevin-like 1 | BF549620 |
| 783 | UI-R-A1-ez-d-07-0-UI | Immunoglobulin heavy chain (alpha polypeptide) | AA955154 |
| 784 | UI-R-A0-ax-a-03-0-UI | Rnf37-pending protein | BF547600 |
| 785 | UI-R-A0-ba-b-08-0-UI | Glutathione S-transferase, theta 3 | AA819129 |
| 786 | UI-R-E1-gl-b-03-0-UI | Bifunctional methylenetetrahydrofolate dehydrogenase/cyclohydrolase, mitochondrial precursor | AA964227 |
| 787 | UI-R-C0-gv-f-03-0-UI | Cyclin A1 | BF557313 |
| 788 | UI-R-E0-cc-a-12-0-UI | Podoplanin | BF554829 |
| 789 | UI-R-E0-ch-f-07-0-UI | N-ethylmaleimide sensitive fusion protein attachment protein alpha | BF555071 |
| 790 | UI-R-E1-fc-g-07-0-UI | Glutathione S-transferase, pi 2 | AA955668 |
| 791 | UI-R-E1-fo-b-03-0-UI | Aldehyde dehydrogenase family 3, subfamily A2 | AA956846 |
| 792 | UI-R-E1-fy-g-03-0-UI | Cartilage homeo protein 1 | AA957438 |
| 793 | UI-R-C0-hz-e-04-0-UI | Dual specificity phosphatase 5 | AA998372 |
| 794 | UI-R-A1-es-e-01-0-UI | Ephrin A1 | AA925894 |
| 795 | UI-R-E1-fy-h-08-0-UI | Polo-like kinase 3 | AA957449 |
| 796 | UI-R-C0-hg-g-09-0-UI | Doublecortin-like kinase 1 | BF561219 |
| 797 | UI-R-C0-jm-h-08-0-UI | Alpha 1 microglobulin/bikunin | AI043784 |
| 798 | UI-R-Y0-lv-h-11-0-UI | Brain and acute leukemia, cytoplasmic | AI070533 |
| 799 | UI-R-C2-mw-f-12-0-UI | PC4 and SFRS1 interacting protein 1 | AI071640 |
| 800 | UI-R-C2-nb-b-10-0-UI | Lymphoid enhancer binding factor 1 | AI071400 |
| 801 | UI-R-C2-nd-c-12-0-UI | XP_001095010.1 THAP domain containing 11 | AI072010 |
| 802 | UI-R-C2-nn-f-07-0-UI | Exportin 4 | AI072630 |
| 803 | UI-R-C2p-ns-d-08-0-UI | Coxsackie virus and adenovirus receptor | BF553315 |
| 804 | UI-R-C2p-nw-d-10-0-UI | Pentatricopeptide repeat domain 1 | BF553598 |
| 805 | UI-R-C2p-ny-g-05-0-UI | Retinoic acid induced 17 | AI136155 |
| 806 | UI-R-E0-bo-d-11-0-UI | Prosaposin | AA858514 |
| 807 | UI-R-E0-bv-f-12-0-UI | Calreticulin | AA859488 |
| 808 | UI-R-C0-if-c-11-0-UI | Receptor (TNFRSF)-interacting serine-threonine kinase 1 | AA998569 |
| 809 | UI-R-C0-ij-h-11-0-UI | Ly1 antibody reactive clone | BF524359 |
| 810 | UI-R-C0-jp-c-10-0-UI | Hephaestin | AI031036 |
| 811 | UI-R-C1-jw-h-02-0-UI | Transcribed locus | AI044591 |
| 812 | UI-R-C1-jy-d-12-0-UI | Transcribed locus | AI045725 |
| 813 | UI-R-C1-kb-d-10-0-UI | Cullin 4B | AI044229 |
| 814 | UI-R-C1-kq-c-10-0-UI | Heparanase | AI059162 |
| 815 | UI-R-C1-kr-f-08-0-UI | Janus kinase 2 | BF554439 |
| 816 | UI-R-C1-kw-c-05-0-UI | Cysteine-rich with EGF-like domains 2 | AI059519 |
| 817 | UI-R-C1-ky-e-12-0-UI | Hypothetical protein MGC59076 | AI071566 |
| 818 | UI-R-A0-aj-e-11-0-UI | APG3 autophagy 3-like | AA866384 |
| 819 | UI-R-A0-am-e-04-0-UI | MHC class II region expressed gene KE2 | BF549298 |
| 820 | UI-R-A0-aq-h-09-0-UI | Basic leucine zipper transcription factor, ATF-like | AA819819 |
| 821 | UI-R-A0-av-a-07-0-UI | NADH dehydrogenase (ubiquinone) 1 beta subcomplex 3 | AA818866 |
| 822 | UI-R-E1-fn-f-06-0-UI | WD repeat domain 12 | AA956631 |
| 823 | UI-R-E1-ft-d-10-0-UI | XP_001091524.1 component of oligomeric golgi complex 5 isoform 1 isoform 1 | BF562163 |
| 824 | UI-R-E1-fv-a-01-0-UI | NP_001035755.1 transmembrane epithelial antigen of the prostate 2 isoform a | BF560333 |
| 825 | UI-R-C0-gr-b-10-0-UI | RGD1565210 | AA964870 |
| 826 | UI-R-C0-hz-e-10-0-UI | Biphenyl hydrolase-like (serine hydrolase, breast epithelial mucin-associated antigen) | AA998291 |
| 827 | UI-R-C0-ic-b-10-0-UI | Stathmin-like 2 | BF561712 |
| 828 | UI-R-C0-gw-c-10-0-UI | ADP-ribosylation factor 5 | AA964078 |
| 829 | UI-R-C0-hb-d-10-0-UI | Cytochrome P450, family 4, subfamily F, polypeptide 2 | AA964981 |
| 830 | UI-R-C0-jh-d-07-0-UI | Jumping translocation breakpoint | AI030613 |
| 831 | UI-R-C0-jo-c-06-0-UI | ADP-ribosyltransferase (NAD+; poly (ADP-ribose) polymerase)-like 1 | AI030775 |
| 832 | UI-R-C1-jv-d-06-0-UI | Thymopoietin | AI044110 |
| 833 | UI-R-C1-kb-h-10-0-UI | P21 (CDKN1A)-activated kinase 2 | AI044252 |
| 834 | UI-R-C1-ll-g-01-0-UI | Proteasome (prosome, macropain) 26S subunit, ATPase, 4 | AI060152 |
| 835 | UI-R-C2-mr-d-02-0-UI | SH3-domain GRB2-like 1 | AI070581 |
| 836 | UI-R-C2-na-e-06-0-UI | Glycosylation dependent cell adhesion molecule 1 | AI070974 |
| 837 | UI-R-C2-nh-g-08-0-UI | Chloride channel 2 | AI071865 |
| 838 | UI-R-DK0-cfx-a-10-0-UI | Pericentrin 1 | BI290593 |
| 839 | UI-R-DK0-cgb-b-05-0-UI | Splicing factor 3a, subunit 2, 66kDa | BI290920 |
| 840 | UI-R-DK0-cgk-b-11-0-UI | Glypican 1 | BI297011 |
| 841 | UI-R-CV2-cgt-e-05-0-UI | KH-type splicing regulatory protein | BI296632 |
| 842 | UI-R-DQ0-cja-j-18-0-UI | Programmed cell death 8 | BI303637 |
| 843 | UI-R-E0-dl-d-07-0-UI | Ribosomal protein S26 | AA900527 |
| 844 | UI-R-A1-dr-d-10-0-UI | Cathepsin E | AA923919 |
| 845 | UI-R-A1-du-e-08-0-UI | Mitogen activated protein kinase kinase 1 | AA924063 |
| 846 | UI-R-E1-gi-a-12-0-UI | Adenosine A2a receptor | BF557584 |
| 847 | UI-R-E1-gp-b-07-0-UI | Secreted phosphoprotein 1 | AA964431 |
| 848 | UI-R-DB0-byz-g-05-0-UI | Prostatic steroid-binding protein C2 | BI285411 |
| 849 | UI-R-DD0-bzu-d-08-0-UI | Actin, gamma 2 | BI281471 |
| 850 | UI-R-CW0s-cce-d-06-0-UI | PDZ-domain protein scribble | BI282719 |
| 851 | UI-R-CX0s-cco-f-01-0-UI | Cathepsin M | BI284391 |
| 852 | UI-R-CX0s-cct-d-07-0-UI | Cytoglobin | BI284885 |
| 853 | UI-R-DK0-cdc-c-02-0-UI | Phytanoyl-CoA 2-hydroxylase 2 | BI288123 |
| 854 | UI-R-DK0-ceo-b-05-0-UI | Ras-GTPase-activating protein SH3-domain binding protein | BI294449 |
| 855 | UI-R-DK0-cez-c-07-0-UI | Desmuslin | BI296344 |
| 856 | UI-R-DK0-cfe-f-07-0-UI | Placenta-specific 8 | BI289114 |
| 857 | UI-R-CM0-bjm-g-11-0-UI | RAB3B, member RAS oncogene family | BF395302 |
| 858 | UI-R-CN0-bmf-b-08-0-UI | Seminal vesicle secretion 1 | BF410882 |
| 859 | UI-R-BT1-bmw-h-09-0-UI | Syndecan 3 | BF411843 |
| 860 | UI-R-CA1-bnr-a-05-0-UI | RAVER1 homolog | BF419384 |
| 861 | UI-R-BJ2-bol-b-06-0-UI | Palmdelphin | BF413989 |
| 862 | UI-R-BJ2-bqi-g-06-0-UI | Actin related protein 2/3 complex, subunit 2 | CK845399 |
| 863 | UI-R-BJ2-bqp-b-01-0-UI | Hydroxysteroid dehydrogenase like 1 | BF419219 |
| 864 | UI-R-CW0-bvw-b-06-0-UI | Tumor necrosis factor receptor superfamily, member 1a | CK845494 |
| 865 | UI-R-CW0-bwg-g-07-0-UI | phospholysine phosphohistidine inorganic pyrophosphate phosphata (5M590) | BI274254 |
| 866 | UI-R-CZ0-byg-a-09-0-UI | Latent transforming growth factor beta binding protein 2 | BI278397 |
| 867 | UI-R-DA0-byn-e-10-0-UI | ATP-binding cassette, sub-family A (ABC1), member 7 | BI279367 |
| 868 | UI-R-CA0-bfx-f-07-0-UI | Transcribed locus | BF394597 |
| 869 | UI-R-CA0-bgl-d-03-0-UI | Adaptor-related protein complex 3, delta 1 subunit | BF401505 |
| 870 | UI-R-CA1-biy-m-08-0-UI | RIKEN cDNA 4931400A14 | BF403404 |
| 871 | UI-R-CA1-bjd-f-08-0-UI | ALEX3 protein | BF403581 |
| 872 | UI-R-CA1-bjf-l-17-0-UI | Nucleolar complex associated 4 homolog | BF406332 |
| 873 | UI-R-CV1-brw-d-11-0-UI | Cathepsin F | BG373242 |
| 874 | UI-R-CT0-bud-h-06-0-UI | CDNA sequence BC024561 | BG381659 |
| 875 | UI-R-CT0-buo-h-08-0-UI | Adenylate cyclase activating polypeptide 1 | BG376387 |
| 876 | UI-R-CU0-buw-g-06-0-UI | Zinc finger protein 580 | BG377160 |
| 877 | UI-R-CU0-bvh-c-06-0-UI | Phosphatidylinositol glycan, class L | BG377965 |
| 878 | UI-R-CA0-axd-e-01-0-UI | Dopamine receptor 2 | BE112846 |
| 879 | UI-R-CA0-axk-b-06-0-UI | Rous sarcoma oncogene | BE110190 |
| 880 | UI-R-BS1-axx-g-10-0-UI | YLP motif containing protein 1 (Nuclear protein ZAP3) (ZAP113) | BE115952 |
| 881 | UI-R-BS1-ayq-a-01-0-UI | Eph receptor B1 | BE107109 |
| 882 | UI-R-CA0-baq-f-12-0-UI | Protein kinase, cGMP-dependent, type II | BE120376 |
| 883 | UI-R-CA0-baz-h-05-0-UI | RGD1566180 | BE119697 |
| 884 | UI-R-CA1-bbq-f-09-0-UI | Tachykinin receptor 1 | BF387196 |
| 885 | UI-R-CA1-bcc-d-09-0-UI | WD repeat domain 17 | BF390554 |
| 886 | UI-R-BS2-bel-f-02-0-UI | Calcium/calmodulin-dependent serine protein kinase (MAGUK family) | BF397490 |
| 887 | UI-R-BS2-bez-f-08-0-UI | LIM domain binding 2 | BF397537 |
| 888 | UI-R-BU0-ang-f-02-0-UI | Amyloid beta (A4) precursor protein-binding, family A, member 1 | AW532912 |
| 889 | UI-R-BU0-apa-e-04-0-UI | Tumor necrosis factor receptor superfamily, member 4 | BE095512 |
| 890 | UI-R-BX0-arm-g-06-0-UI | Kinetochore associated 1 | BE104266 |
| 891 | UI-R-BO1-asj-b-11-0-UI | Mannosidase 2, alpha 2 | BE105872 |
| 892 | UI-R-BO1-asr-a-08-0-UI | Neurotrophic tyrosine kinase, receptor, type 3 | BE106688 |
| 893 | UI-R-BJ1-ata-h-09-0-UI | Zinc finger protein 180 | CK842675 |
| 894 | UI-R-BJ1-aup-a-04-0-UI | Crystallin, mu | BE109688 |
| 895 | UI-R-BJ1-avb-e-09-0-UI | Lysyl oxidase-like 1 | CK844035 |
| 896 | UI-R-BJ1-avn-d-12-0-UI | Protective protein for beta-galactosidase | BE112592 |
| 897 | UI-R-BJ1-avt-g-11-0-UI | Obscurin, cytoskeletal calmodulin and titin-interacting RhoGEF | BE110595 |
| 898 | UI-R-BO0-ahy-b-04-0-UI | Zinc finger proliferation 1 | BF568019 |
| 899 | UI-R-BO0-aie-e-06-0-UI | Solute carrier organic anion transporter family, member 1c1 | AW523920 |
| 900 | UI-R-BJ0p-ail-b-01-0-UI | Rad54 like 2 | BF562775 |
| 901 | UI-R-BJ0p-ais-b-08-0-UI | Asparagine-linked glycosylation 3 homolog (yeast, alpha-1,3-mannosyltransferase) | BF563327 |
| 902 | UI-R-BO1-ajv-d-10-0-UI | Transmembrane protein 115 | BF565382 |
| 903 | UI-R-BT1-akc-e-11-0-UI | Membrane protein, palmitoylated 2 (MAGUK p55 subfamily member 2) | BF566303 |
| 904 | UI-R-BT1-akj-g-03-0-UI | YTH domain family 3 | AW528886 |
| 905 | UI-R-BT1-akq-f-04-0-UI | Tumor necrosis factor (ligand) superfamily, member 13 | BF563730 |
| 906 | UI-R-BU0-ams-h-04-0-UI | Neuronal regeneration related protein | AW532846 |
| 907 | UI-R-BU0-ana-b-12-0-UI | Cysteine and histidine-rich domain (CHORD)-containing, zinc-binding protein 1 | BF564901 |
| 908 | UI-R-AC0-yq-e-11-0-UI | Heat shock 27kD protein family, member 7 | BF525257 |
| 909 | UI-R-AB1-yy-h-03-0-UI | DNA topoisomerase I, mitochondrial | CK838963 |
| 910 | UI-R-AF1-aav-b-07-0-UI | NP_067412.1 protein LOC58248 | CK838459 |
| 911 | UI-R-Y0-abc-b-02-0-UI | Fas apoptotic inhibitory molecule 2 | AI713005 |
| 912 | UI-R-Y0-abp-g-03-0-UI | Hect domain and RLD 3 | AI715299 |
| 913 | UI-R-Y0-acb-e-05-0-UI | Arginase 1 | BF521981 |
| 914 | UI-R-BJ0p-aex-h-11-0-UI | RIKEN cDNA 1110012L19 | CK841142 |
| 915 | UI-R-BJ0p-afg-g-09-0-UI | Defensin beta 29 | CK839317 |
| 916 | UI-R-BJ0p-afn-c-10-0-UI | Glutaredoxin 5 homolog | AW433936 |
| 917 | UI-R-BJ0p-afx-a-06-0-UI | Protein phosphatase 2 (formerly 2A), regulatory subunit A (PR 65), beta isoform | AW520483 |
| 918 | UI-R-C2p-rj-d-03-0-UI | Minichromosome maintenance deficient 6 | BF546309 |
| 919 | UI-R-C2p-rq-h-07-0-UI | Janus kinase 3 | BF546077 |
| 920 | UI-R-C2p-sb-e-10-0-UI | endoplasmic oxidoreductase 1 beta | AI501472 |
| 921 | UI-R-C3-sj-b-09-0-UI | Claudin 3 | BF542491 |
| 922 | UI-R-C3-tz-f-07-0-UI | Zinc finger, DHHC domain containing 23 | AI548865 |
| 923 | UI-R-G0-uj-g-09-0-UI | TBC1 domain family, member 8; BUB2-like protein 1; vascular Rab-GAP/TBC-containing | AI577924 |
| 924 | UI-R-Y0-uv-a-05-0-UI | Immediate early response 5 | BF522725 |
| 925 | UI-R-Y0-vd-a-09-0-UI | Fatty acid binding protein 7, brain | BF523766 |
| 926 | UI-R-AC1-xs-e-10-0-UI | RT1 class Ib, locus Aw2 | AI705421 |
| 927 | UI-R-AC0-yi-a-01-0-UI | TATA box binding protein | CK838819 |
| 928 | UI-R-E1-fu-e-06-0-UI | Tryptophan rich basic protein | BF560317 |
| 929 | UI-R-E1-gf-a-07-0-UI | 2410001H17Rik protein | AA957634 |
| 930 | UI-R-C0-jd-f-07-0-UI | Secretory leukocyte peptidase inhibitor | AI502239 |
| 931 | UI-R-C1-jw-b-05-0-UI | Rearranged L-myc fusion sequence | AI044562 |
| 932 | UI-R-C1-kr-c-08-0-UI | Prostaglandin F receptor | CK840712 |
| 933 | UI-R-C1-lj-a-07-0-UI | Putative GTP-binding protein | BF546725 |
| 934 | UI-R-C2p-oh-e-04-0-UI | Elongation of very long chain fatty acids (FEN1/Elo2, SUR4/Elo3, yeast)-like 2 | AI172603 |
| 935 | UI-R-BT0-pm-b-02-0-UI | Tnf receptor-associated factor 7 | BF553700 |
| 936 | UI-R-BT0-ps-c-09-0-UI | Proteasome (prosome, macropain) 26S subunit, non-ATPase, 6 | AI145614 |
| 937 | UI-R-BT0-qa-b-01-0-UI | Fas apoptotic inhibitory molecule | BF545715 |
| 938 | UI-R-A0-ai-c-08-0-UI | Myeloid differentiation primary response gene 88 | AI453865 |
| 939 | UI-R-A0-ay-c-09-0-UI | Scrapie responsive gene 1 | BF549743 |
| 940 | UI-R-A0-bl-h-09-0-UI | Solute carrier family 34 (sodium phosphate), member 1 | AA819433 |
| 941 | UI-R-E0-br-g-12-0-UI | High mobility group AT-hook 1 | AA866475 |
| 942 | UI-R-E0-cw-e-09-0-UI | Transcribed locus | AA923930 |
| 943 | UI-R-E0-dd-g-01-0-UI | Chromodomain helicase DNA binding protein 7 | AA900224 |
| 944 | UI-R-E0-dk-f-02-0-UI | Testis specific protein kinase 1 | BF558316 |
| 945 | UI-R-A1-dq-c-09-0-UI | Transcribed locus | AA901404 |
| 946 | UI-R-A1-eq-c-02-0-UI | Pyrroline-5-carboxylate synthetase (glutamate gamma-semialdehyde synthetase) | AA926225 |
| 947 | UI-R-E1-fc-b-04-0-UI | POU domain, class 3, transcription factor 1 | BF559366 |
| 948 | UI-R-CA1-bjb-n-11-0-UI | N-acetylneuraminic acid phosphatase | BF399464 |
| 949 | UI-R-CX0-bxi-e-10-0-UI | Signal-induced proliferation-associated gene 1 | BI275966 |
| 950 | UI-R-DN0-ciu-d-08-0-UI | Tubulointerstitial nephritis antigen | BI292092 |
| 951 | UI-R-E1-gd-a-03-0-UI | Oxysterol binding protein 2 | AA962986 |
| 952 | UI-R-E1-gq-a-08-0-UI | chromosome 16 open reading frame 5 | BF561161 |
| 953 | UI-R-C0-ib-g-11-0-UI | hypothetical protein MGC32132 | BF561700 |
| 954 | UI-R-C1-jw-e-09-0-UI | Transcribed locus | AI044487 |
| 955 | UI-R-C2p-qw-e-04-0-UI | E2f3 protein | AI555342 |
| 956 | UI-R-C2p-rm-e-09-0-UI | XP_001102385.1 hypothetical protein | CK840295 |
| 957 | UI-R-C0-iq-b-01-0-UI | RAN binding protein 5 | BF551452 |
| 958 | UI-R-C0-iw-h-04-0-UI | Ankyrin repeat domain 11 | AI029504 |
| 959 | UI-R-C1-kl-e-03-0-UI | Brca1 associated protein 1 | AI045769 |
| 960 | UI-R-C1-ko-b-04-0-UI | Regulator of telomere elongation helicase 1 | AI071280 |
| 961 | UI-R-C1-lh-c-09-0-UI | CG14980-PB | BF549512 |
| 962 | UI-R-C1-lm-a-08-0-UI | novel protein | BF559907 |
| 963 | UI-R-E1-fy-c-10-0-UI | RT1 class I, locus Ke4 | AA999124 |
| 964 | UI-R-C0-ih-h-07-0-UI | NP61201 | AA999024 |
| 965 | UI-R-C1-ka-a-10-0-UI | FLN29 gene product | CK840696 |
| 966 | UI-R-C1-lq-f-01-0-UI | Zinc finger protein 292 | AI059013 |
| 967 | UI-R-C0-hh-c-11-0-UI | Basic leucine zipper and W2 domains 2 | BF561232 |
| 968 | UI-R-C0-ir-e-02-0-UI | Fibroblast growth factor (acidic) intracellular binding protein | AI029444 |
| 969 | UI-R-C1-js-d-03-0-UI | Rhesus blood group-associated C glycoprotein | AI044046 |
| 970 | UI-R-C1-li-h-06-0-UI | Actin-binding Rho activating protein | BF549525 |
| 971 | UI-R-A1-eu-g-03-0-UI | Endonuclease G | AA926357 |
| 972 | UI-R-E1-fc-h-03-0-UI | Nuclear fragile X mental retardation protein interacting protein 1 | AA955675 |
| 973 | UI-R-E1-fv-e-08-0-UI | Transcribed locus | BF556573 |
| 974 | UI-R-E1-fz-a-06-0-UI | SET binding protein 1 | AI137781 |
| 975 | UI-R-C0-ig-a-07-0-UI | F-box only protein 3 isoform 1 | BF553955 |
| 976 | UI-R-C0-il-h-05-0-UI | Nudix (nucleoside diphosphate linked moiety X)-type motif 7 | AI028929 |
| 977 | UI-R-E0-dj-a-11-0-UI | Tyrosine 3-monooxygenase/tryptophan 5-monooxygenase activation protein, epsilon polypeptide | AA900933 |
| 978 | UI-R-A1-ew-g-08-0-UI | Ceruloplasmin | AA955455 |
| 979 | UI-R-C0-in-h-12-0-UI | Chondroitin sulfate proteoglycan 6 | BF545221 |
| 980 | UI-R-C0-iw-a-06-0-UI | PDZ and LIM domain 1 (elfin) | BF551604 |
| 981 | UI-R-C0-is-g-03-0-UI | Solute carrier family 25 (mitochondrial carrier; adenine nucleotide translocator), member 3 | AI044410 |
| 982 | UI-R-BT0-qb-g-06-0-UI | ATP synthase, H+ transporting, mitochondrial F1 complex, beta polypeptide | AI146173 |
| 983 | UI-R-C1-kw-b-11-0-UI | Wild-type p53-induced gene 1 | AI059419 |
| 984 | UI-R-C2-ms-c-06-0-UI | Hyaluronan and proteoglycan link protein 2 | AI070870 |
| 985 | UI-R-E0-bs-c-10-0-UI | Solute carrier family 25 (mitochondrial carrier; dicarboxylate transporter), member 10 | BF550114 |
| 986 | UI-R-E0-cx-g-11-0-UI | Polymerase (RNA) II (DNA directed) polypeptide G | AA899179 |
| 987 | UI-R-C2-nf-b-12-0-UI | RAB3 GTPase activating protein subunit 2 | AI072072 |
| 988 | UI-R-C2-ng-h-02-0-UI | Deoxyguanosine kinase | BF553025 |
| 989 | UI-R-C2-nj-g-08-0-UI | Solute carrier family 16 (monocarboxylic acid transporters), member 10 | BF553195 |
| 990 | UI-R-C2-nl-g-08-0-UI | S-phase kinase-associated protein 1A | AI072207 |
| 991 | UI-R-A0-ah-b-07-0-UI | Ribosomal protein L24 | AA817997 |
| 992 | UI-R-A0-ar-h-08-0-UI | NP_066953.1 isomerase A isoform 1 | BF522138 |
| 993 | UI-R-A0-az-h-11-0-UI | Argininosuccinate lyase | AA818673 |
| 994 | UI-R-A0-bh-g-04-0-UI | Ribosomal protein S23 | AA900188 |
| 995 | UI-R-E0-cp-c-06-0-UI | Fc receptor, IgG, alpha chain transporter | AA875464 |
| 996 | UI-R-E0-cz-c-02-0-UI | Inhibitor of DNA binding 1 | AA899322 |
| 997 | UI-R-C0-jg-b-04-0-UI | Mitochondrial ribosomal protein L47 | AI045672 |
| 998 | UI-R-C0-jj-b-08-0-UI | Aspartoacylase | BF550548 |
| 999 | UI-R-C1-kd-d-08-0-UI | Pleckstrin homology domain containing, family M (with RUN domain) member 1 | BF544790 |
| 1000 | UI-R-C1-kf-f-01-0-UI | Ribonuclease T2 | AI045043 |
| 1001 | UI-R-C1-ki-f-05-0-UI | Tumor protein p53 inducible protein 13 | AI044832 |
| 1002 | UI-R-C1-kk-d-07-0-UI | hypothetical protein FLJ11712 | AI045160 |
| 1003 | UI-R-C1-lb-d-07-0-UI | Copine VIII | AI059204 |
| 1004 | UI-R-C1-le-h-06-0-UI | Cleavage and polyadenylation specific factor 2 | BF549533 |
| 1005 | UI-R-C1-lo-c-08-0-UI | GH regulated TBC protein 1 | BF552250 |
| 1006 | UI-R-C1-lq-a-02-0-UI | Ribonuclease, RNase A family, 6 | BF552301 |
| 1007 | UI-R-A0-bf-f-06-0-UI | Poly(rC) binding protein 2 | AA819535 |
| 1008 | UI-R-E1-fa-f-12-0-UI | Hypothetical protein LOC680815 | AA955536 |
| 1009 | UI-R-E1-fg-h-11-0-UI | XP_001118829.1 TBC1 domain family, member 14 | AA955771 |
| 1010 | UI-R-E1-fl-b-09-0-UI | Bromodomain containing 1 | AA956934 |
| 1011 | UI-R-C0-gx-e-03-0-UI | Stromal cell-derived factor 2-like 1 | BF557838 |
| 1012 | UI-R-C0-he-b-02-0-UI | Complement component 1, q subcomponent, alpha polypeptide | AA996499 |
| 1013 | UI-R-C0-hi-d-09-0-UI | RAB5B, member RAS oncogene family | AA996576 |
| 1014 | UI-R-C0-hu-g-11-0-UI | Biogenesis of lysosome-related organelles complex-1, subunit 1 | AA997882 |
| 1015 | UI-R-C0-it-h-11-0-UI | Iroquois related homeobox 3 | AI030203 |
| 1016 | UI-R-C0-iy-h-05-0-UI | hypothetical protein FLJ21148 | AI029718 |
| 1017 | UI-R-C0-hu-c-08-0-UI | Dimethylglycine dehydrogenase precursor | BF550714 |
| 1018 | UI-R-C0-iz-g-03-0-UI | Scaffold attachment factor B | AI029791 |
| 1019 | UI-R-C1-kh-h-07-0-UI | Heat shock 70kD protein 1-like | BF546948 |
| 1020 | UI-R-C1-ko-e-10-0-UI | Casein kinase 1, gamma 3 | AI071315 |
| 1021 | UI-R-C1-kx-c-11-0-UI | NP_001041567.1 proliferative activated receptor, delta | AI058392 |
| 1022 | UI-R-C1-lf-e-04-0-UI | XP_001105924.1 eukaryotic translation initiation factor 2, subunit 1 alpha, 35kDa isoform 2 | AI059334 |
| 1023 | UI-R-C2p-nv-f-12-0-UI | Bone morphogenetic protein 4 | BF553423 |
| 1024 | UI-R-C2p-oh-g-03-0-UI | Tenascin XA | AI172612 |
| 1025 | UI-R-BT0-pn-h-12-0-UI | Huntingtin-associated protein 1 | AI144717 |
| 1026 | UI-R-A0-ae-h-08-0-UI | FXYD domain-containing ion transport regulator 6 | BF548182 |
| 1027 | UI-R-CV2-cht-h-06-0-UI | Solute carrier family 8 (sodium/calcium exchanger), member 3 | BI299332 |
| 1028 | UI-R-DM0-ciq-d-19-0-UI | Cytidine monophospho-N-acetylneuraminic acid hydroxylase | BI302320 |
| 1029 | UI-R-DN0-ciu-l-14-0-UI | Transmembrane protease, serine 2 | BI292133 |
| 1030 | UI-R-DO0-ciw-m-13-0-UI | SWI/SNF related, matrix associated, actin dependent regulator of chromatin, subfamily a, member 5 | BI292524 |
| 1031 | UI-R-A1-dz-f-04-0-UI | Interferon regulatory factor 1 | AA924546 |
| 1032 | UI-R-A1-ec-h-01-0-UI | Surfactant, pulmonary-associated protein A1 | BF556197 |
| 1033 | UI-R-A1-eg-e-09-0-UI | Solute carrier family 16 (monocarboxylic acid transporters), member 7 | BF550412 |
| 1034 | UI-R-A1-el-a-08-0-UI | Enoyl coenzyme A hydratase 1, peroxisomal | AA926032 |
| 1035 | UI-R-C0-hi-e-06-0-UI | Achaete-scute complex homolog-like 1 | AA996446 |
| 1036 | UI-R-C0-hp-b-07-0-UI | Annexin A3 | BF554231 |
| 1037 | UI-R-CU0s-cbs-d-03-0-UI | Phosphoenolpyruvate carboxykinase 1 | BI281773 |
| 1038 | UI-R-CU0s-cby-g-02-0-UI | Heat shock 70kDa protein 9A | BI282281 |
| 1039 | UI-R-DK0-cdj-c-02-0-UI | Cyclin M2 | BI293019 |
| 1040 | UI-R-DK0-cdu-a-11-0-UI | Potassium inwardly-rectifying channel, subfamily J, member 16 | BI293649 |
| 1041 | UI-R-DK0-cec-e-10-0-UI | V-ral simian leukemia viral oncogene homolog A (ras related) | BI294822 |
| 1042 | UI-R-DK0-cef-d-09-0-UI | splicing factor, arginine/serine-rich 2 | BI295066 |
| 1043 | UI-R-CA0-blz-d-01-0-UI | Ryanodine receptor 2, cardiac | BF418091 |
| 1044 | UI-R-BJ2-bra-e-09-0-UI | Zinc finger protein 278 | BF408170 |
| 1045 | UI-R-BJ2-brf-b-05-0-UI | Katanin p80 (WD40-containing) subunit B 1 | BF408552 |
| 1046 | UI-R-DK0-cfo-d-07-0-UI | Polycystic kidney disease 1 homolog | BI289803 |
| 1047 | UI-R-BJ2-bos-c-01-0-UI | mKIAA1737 protein | BF414801 |
| 1048 | UI-R-BJ2-bph-f-04-0-UI | 2310050N11Rik protein | BF419910 |
| 1049 | UI-R-BJ2-bpp-c-04-0-UI | NP_001020471.2 member RAS oncogene family | BF406798 |
| 1050 | UI-R-BJ2-bpx-g-06-0-UI | XP_001088569.1 PDZ domain containing 1 isoform 1 | CK843605 |
| 1051 | UI-R-CX0-bwq-b-03-0-UI | ATPase, H+ transporting, V0 subunit D isoform 1 | BI276424 |
| 1052 | UI-R-CX0-bxd-f-03-0-UI | 5-hydroxytryptamine (serotonin) receptor 1D | BI274751 |
| 1053 | UI-R-CX0-bxk-g-01-0-UI | Eukaryotic translation initiation factor 3, subunit 8, 110kDa | BI277887 |
| 1054 | UI-R-CY0-bxs-f-02-0-UI | hypothetical protein MGC7537 | BI277555 |
| 1055 | UI-R-DE0-cag-e-03-0-UI | Glycoprotein (transmembrane) nmb | BI280315 |
| 1056 | UI-R-CT0s-cav-h-06-0-UI | XP_001108087.1 proteasome beta 9 subunit isoform 2 proprotein | BI281736 |
| 1057 | UI-R-CA1-bhz-g-11-0-UI | NIMA (never in mitosis gene a)-related expressed kinase 1 | BF399864 |
| 1058 | UI-R-CA1-biq-e-02-0-UI | Solute carrier organic anion transporter family, member 3a1 | BF405156 |
| 1059 | UI-R-CV1-bse-f-01-0-UI | Crystallin, alpha A | BG373590 |
| 1060 | UI-R-CV1-bsr-a-10-0-UI | cytochrome P450, family 2, subfamily J, polypeptide 4 | BG374493 |
| 1061 | UI-R-CS0-btl-f-11-0-UI | Fatty acid binding protein 5, epidermal | BG380069 |
| 1062 | UI-R-CT0-btw-f-10-0-UI | XP_001106978.1 BCL2/adenovirus E1B 19kD interacting protein like isoform 1 | BG380394 |
| 1063 | UI-R-CV1-bvq-e-02-0-UI | 2310043K02Rik protein | BG378299 |
| 1064 | UI-R-CA0-bkj-f-12-0-UI | Zinc finger protein 294 | BF416362 |
| 1065 | UI-R-CN0-bky-e-12-0-UI | Calmodulin 3 | BF417308 |
| 1066 | UI-R-CN0-blg-h-08-0-UI | Nuclear factor of kappa light chain gene enhancer in B-cells inhibitor, alpha | BF417507 |
| 1067 | UI-R-BS1-ayx-c-10-0-UI | Cbp/p300-interacting transactivator, with Glu/Asp-rich carboxy-terminal domain, 4 | BE108268 |
| 1068 | UI-R-BS1-aze-f-03-0-UI | Polymerase (DNA directed), delta 1, catalytic subunit | BE117676 |
| 1069 | UI-R-BS1-azr-d-10-0-UI | THO complex 2 | BE118674 |
| 1070 | UI-R-CA0-baj-a-04-0-UI | Solute carrier family 24 (sodium/potassium/calcium exchanger), member 2 | BE119874 |
| 1071 | UI-R-CA1-bcp-f-05-0-UI | 1-acylglycerol-3-phosphate O-acyltransferase 1 | BF390804 |
| 1072 | UI-R-BS2-bdd-g-08-0-UI | Origin recognition complex, subunit 3-like | BF388634 |
| 1073 | UI-R-BS2-bdq-a-02-0-UI | Zinc finger, BED domain containing 4 | BF389898 |
| 1074 | UI-R-BS2-bdz-h-07-0-UI | Myeloid/lymphoid or mixed-lineage leukemia | BF396591 |
| 1075 | UI-R-CA0-bgw-e-06-0-UI | Heterogeneous nuclear ribonucleoprotein U-like 1 | BF393911 |
| 1076 | UI-R-CA0-bhg-h-06-0-UI | Transcribed locus | BF400876 |
| 1077 | UI-R-BT1-aqh-h-09-0-UI | RIKEN cDNA 2810002D13 gene | BE101835 |
| 1078 | UI-R-BT1-aqu-f-01-0-UI | RIKEN cDNA 9330177P20 | BE102964 |
| 1079 | UI-R-BJ1-atl-e-03-0-UI | mKIAA1797 protein | BE099902 |
| 1080 | UI-R-BJ1-atq-h-07-0-UI | Ras and a-factor-converting enzyme 1 homolog | CK842994 |
| 1081 | UI-R-BJ1-atw-f-04-0-UI | Type II keratin Kb4 | CK839343 |
| 1082 | UI-R-BJ1-auf-d-08-0-UI | Presenilin 2 | BE100792 |
| 1083 | UI-R-BJ1-avy-g-11-0-UI | Seryl-tRNA synthetase 2 | CK844256 |
| 1084 | UI-R-BJ1-awc-g-02-0-UI | Glutamate oxaloacetate transaminase 2, mitochondrial | BE113202 |
| 1085 | UI-R-BJ1-awk-d-01-0-UI | Grb10 protein | BE114278 |
| 1086 | UI-R-CA0-awv-e-10-0-UI | Sorting nexin 14 | BE107598 |
| 1087 | UI-R-BO1-aiy-e-05-0-UI | Transmembrane protein 106B | AW526034 |
| 1088 | UI-R-BO1-ajg-b-11-0-UI | Protein tyrosine phosphatase, receptor type, K, extracellular region | BF563186 |
| 1089 | UI-R-BO1-ajj-b-09-0-UI | Golgi apparatus protein 1 | BF565240 |
| 1090 | UI-R-BO1-ajp-g-11-0-UI | Thromboxane A2 receptor | BF565745 |
| 1091 | UI-R-C4-akz-g-02-0-UI | CG3740-PA | AW531833 |
| 1092 | UI-R-C4-alh-b-05-0-UI | Hypothetical LOC308869 | AW531937 |
| 1093 | UI-R-C4-alp-d-07-0-UI | Mediator of RNA polymerase II transcription, subunit 8 homolog | AW534120 |
| 1094 | UI-R-C4-alx-h-11-0-UI | Zinc finger protein 307 | AW535557 |
| 1095 | UI-R-BU0-apg-f-11-0-UI | Esterase D/formylglutathione hydrolase | BE096486 |
| 1096 | UI-R-BO1-apv-e-06-0-UI | Transcribed locus | BE097283 |
| 1097 | UI-R-AA1-zy-g-02-0-UI | Microtubule-associated protein 1 A | CK839191 |
| 1098 | UI-R-AG1-aam-b-03-0-UI | Serine (or cysteine) peptidase inhibitor, clade F, member 1 | CK838424 |
| 1099 | UI-R-Y0-aci-b-09-0-UI | Chromogranin A | AI716872 |
| 1100 | UI-R-Y0-acu-g-06-0-UI | Solute carrier family 25 (mitochondrial carrier; adenine nucleotide translocator), member 4 | AI717084 |
| 1101 | UI-R-BJ0-add-g-12-0-UI | Protein phosphatase 2, regulatory subunit B (B56), alpha isoform | AW252634 |
| 1102 | UI-R-BJ0-aed-a-01-0-UI | heart alpha-kinase | AW253922 |
| 1103 | UI-R-BJ0p-age-f-02-0-UI | Suppressor of Ty 6 homolog | CK841353 |
| 1104 | UI-R-BO0-agl-f-10-0-UI | WD repeat domain 41 | AW521367 |
| 1105 | UI-R-BO0-agy-d-12-0-UI | BMP/retinoic acid-inducible neural-specific protein 3 | BF567336 |
| 1106 | UI-R-BO0-aho-c-09-0-UI | Neurofilament, light polypeptide | AW523447 |
| 1107 | UI-R-C3-sr-h-07-0-UI | Aminoacylase 1-like 2 | BF523195 |
| 1108 | UI-R-C3-sz-e-06-0-UI | Glioma tumor suppressor candidate region gene 1 | BF543487 |
| 1109 | UI-R-C3-tk-d-03-0-UI | hypothetical protein FLJ34389 | BF524401 |
| 1110 | UI-R-C3-ts-d-06-0-UI | Stromal antigen 2 | AI549167 |
| 1111 | UI-R-AB0-vt-h-07-0-UI | Interferon-related developmental regulator 2 | AI576921 |
| 1112 | UI-R-AD0-wg-g-05-0-UI | Tubulin, alpha 1 | AI579831 |
| 1113 | UI-R-AG0-wu-b-06-0-UI | Acetyl-Coenzyme A acetyltransferase 2 | BF524229 |
| 1114 | UI-R-AG0-xc-g-08-0-UI | RNA-binding protein Musashi2-S | CK844823 |
| 1115 | UI-R-AE1-zj-d-06-0-UI | Ets variant gene 1 | BF524947 |
| 1116 | UI-R-AD1-zp-e-01-0-UI | Protein kinase N3 | CK839725 |
| 1117 | UI-R-C0-hr-f-08-0-UI | 2310047B19Rik protein | CK845233 |
| 1118 | UI-R-C0-ie-b-06-0-UI | NADH-ubiquinone oxidoreductase B9 subunit (Complex I-B9) (CI-B9) | AA998235 |
| 1119 | UI-R-Y0-ma-f-09-0-UI | Fyn-related kinase | AI111536 |
| 1120 | UI-R-Y0-mo-h-05-0-UI | Potassium voltage gated channel, Shab-related subfamily, member 1 | AI112120 |
| 1121 | UI-R-C2-nf-c-02-0-UI | Fatty acid binding protein 1, liver | BF552985 |
| 1122 | UI-R-C2p-nw-g-08-0-UI | UDP-N-acetyl-alpha-D-galactosamine:polypeptide N-acetylgalactosaminyltransferase 10 | BF522221 |
| 1123 | UI-R-BT0-qh-h-02-0-UI | F-box and leucine-rich repeat protein 2 | BF544605 |
| 1124 | UI-R-C2p-qn-e-12-0-UI | Transcribed locus | AI555098 |
| 1125 | UI-R-C2p-qt-f-12-0-UI | Sema domain, immunoglobulin domain (Ig), transmembrane domain (TM) and short cytoplasmic domain, (semaphorin) 4G | AI555178 |
| 1126 | UI-R-C2p-rd-a-02-0-UI | Chordin | BF547335 |
| 1127 | UI-R-E0-bv-e-04-0-UI | Shb protein | AA859468 |
| 1128 | UI-R-E0-ca-f-03-0-UI | Cathepsin Z | AA859956 |
| 1129 | UI-R-E0-ci-b-04-0-UI | Transcribed locus | AI454329 |
| 1130 | UI-R-E0-co-c-04-0-UI | 3'UTR of CDK6 (unknown) protein | BF544067 |
| 1131 | UI-R-A1-dv-e-12-0-UI | Solute carrier family 22 (organic anion/cation transporter), member 12 | AA901051 |
| 1132 | UI-R-A1-dz-c-10-0-UI | CDC like kinase 4 | AA924654 |
| 1133 | UI-R-A1-ef-a-07-0-UI | Signal transducer and activator of transcription 1 | BF550244 |
| 1134 | UI-R-A1-ej-b-10-0-UI | Glycine C-acetyltransferase (2-amino-3-ketobutyrate-coenzyme A ligase) | BF544099 |
| 1135 | UI-R-C0-gr-f-07-0-UI | SplA/ryanodine receptor domain and SOCS box containing 2 | AI113003 |
| 1136 | UI-R-C0-hl-g-03-0-UI | Hypothetical protein LOC691452 | AA997471 |
| 1137 | UI-R-BT0-pp-d-01-0-UI | Aldehyde dehydrogenase 3 family, member B1 | AI144910 |
| 1138 | UI-R-Y0-abm-c-09-0-UI | RNA polymerase 1-1 | AI715416 |
| 1139 | UI-R-C4-alj-b-11-0-UI | Growth arrest and DNA-damage-inducible, gamma interacting protein 1 | AW532171 |
| 1140 | UI-R-CA1-bcp-f-10-0-UI | Purinergic receptor P2Y, G-protein coupled, 13 | BF390809 |
| 1141 | UI-R-E0-cb-d-07-0-UI | Methionine aminopeptidase-like 1 | AA875011 |
| 1142 | UI-R-E0-dk-h-01-0-UI | S100 calcium binding protein A15 | BF556629 |
| 1143 | UI-R-A1-dy-d-02-0-UI | Vaccinia related kinase 1 | BF555986 |
| 1144 | UI-R-E1-fd-a-09-0-UI | Transcription factor E2F3 (E2F-3) | AA955896 |
| 1145 | UI-R-C2-mu-d-12-0-UI | JAZF zinc finger 1 | BF553072 |
| 1146 | UI-R-C2p-oh-b-10-0-UI | tumor necrosis factor receptor superfamily, member 25 | AI137157 |
| 1147 | UI-R-C0-hc-e-08-0-UI | Mitochondrial ribosomal protein S27 | AI137642 |
| 1148 | UI-R-C0-hk-a-04-0-UI | Hypothetical protein LOC691995 | BF554062 |
| 1149 | UI-R-C0-jf-a-05-0-UI | Abhydrolase domain containing 1 | AI030936 |
| 1150 | UI-R-C0-jo-c-05-0-UI | PERP, TP53 apoptosis effector | BF550780 |
| 1151 | UI-R-C1-jt-e-12-0-UI | Nicotinamide N-methyltransferase | BF551157 |
| 1152 | UI-R-C1-jz-f-09-0-UI | Microtubule-associated serine/threonine-protein kinase 3 | AI045461 |
| 1153 | UI-R-E0-cj-b-10-0-UI | Tropomyosin 3, gamma | AA859305 |
| 1154 | UI-R-E0-dk-d-08-0-UI | V-raf-1 murine leukemia viral oncogene homolog 1 | AA900883 |
| 1155 | UI-R-A0-aq-e-01-0-UI | H3 histone, family 3B | AA819782 |
| 1156 | UI-R-E1-fa-g-05-0-UI | MAD2L1 binding protein | BF557355 |
| 1157 | UI-R-C0-ig-c-09-0-UI | Abl-interactor 2 | AA998956 |
| 1158 | UI-R-A0-bh-g-02-0-UI | Potassium channel tetramerisation domain containing 13 | AA900186 |
| 1159 | UI-R-E1-fb-g-07-0-UI | Interferon, alpha-inducible protein 27-like | AA955996 |
| 1160 | UI-R-C0-gu-f-04-0-UI | Splicing factor proline/glutamine rich (polypyrimidine tract binding protein associated) | BF557275 |
| 1161 | UI-R-A0-bk-f-03-0-UI | Immunoglobulin heavy chain 1a (serum IgG2a) | AA819363 |
| 1162 | UI-R-A0-bh-h-02-0-UI | Mitogen-activated protein kinase kinase 1 interacting protein 1 | AA900195 |
| 1163 | UI-R-A0-ay-b-03-0-UI | Proline-rich Gla (G-carboxyglutamic acid) polypeptide 2 | BF549733 |
| 1164 | UI-R-A1-es-g-12-0-UI | Myoneurin | BF558392 |
| 1165 | UI-R-C0-gt-g-03-0-UI | XP_001091601.1 cathepsin D isoform 3 | AA964112 |
| 1166 | UI-R-C0-gz-d-11-0-UI | EH-domain containing 1 | AA965111 |
| 1167 | UI-R-E0-cf-b-09-0-UI | Hydroxysteroid (17-beta) dehydrogenase 4 | AA874974 |
| 1168 | UI-R-E0-ck-g-09-0-UI | MutS homolog 2 | AA874919 |
| 1169 | UI-R-E1-fh-a-01-0-UI | Solute carrier family 16 (monocarboxylic acid transporters), member 1 | AA956058 |
| 1170 | UI-R-E1-ft-g-10-0-UI | Protein phosphatase 5, catalytic subunit | AA957215 |
| 1171 | UI-R-E1-ge-c-09-0-UI | Transcribed locus |  |
| 1172 | UI-R-C0-ih-b-01-0-UI | Secretory granule neuroendocrine protein 1 | BF553996 |
| 1173 | UI-R-E1-fr-b-10-0-UI | Putative small membrane protein NID67 | AA956903 |
| 1174 | UI-R-C0-gt-b-08-0-UI | RAB13, member RAS oncogene family | BF559668 |
| 1175 | UI-R-C0-iw-e-03-0-UI | Granzyme A | AI029488 |
| 1176 | UI-R-C1-jv-g-11-0-UI | Actinin alpha 4 | AI044058 |
| 1177 | UI-R-C2-mw-a-10-0-UI | Mitochondrial ribosomal protein S18C | AI071605 |
| 1178 | UI-R-C2-na-b-08-0-UI | CG31653-PA | BF552937 |
| 1179 | UI-R-C2-nb-h-05-0-UI | PYD and CARD domain containing | AI071267 |
| 1180 | UI-R-C2-ne-a-05-0-UI | Zinc finger protein ZFPM1 (Zinc finger protein multitype 1) (Friend of GATA protein 1) (Friend of GATA-1) (FOG-1) | AI072257 |
| 1181 | UI-R-C2p-nq-a-10-0-UI | eukaryotic translation initiation factor 3, subunit 3 gamma, 40kDa | BF521831 |
| 1182 | UI-R-C2p-nu-g-08-0-UI | Hypothetical protein LOC688422 | BF553378 |
| 1183 | UI-R-C2p-nx-g-06-0-UI | Protein KIAA1543 | BF553661 |
| 1184 | UI-R-C2p-ob-b-06-0-UI | Kringle containing transmembrane protein 1 | BF553798 |
| 1185 | UI-R-E0-bt-f-01-0-UI | NSFL1 (p97) cofactor (p47) | BF547737 |
| 1186 | UI-R-E0-bz-b-03-0-UI | ATPase, H transporting, lysosomal V1 subunit F | AA899050 |
| 1187 | UI-R-C0-ii-g-01-0-UI | FK506 binding protein 8 | AA998836 |
| 1188 | UI-R-C0-ik-g-09-0-UI | RIKEN cDNA 6720467C03 | AI028889 |
| 1189 | UI-R-C0-jp-h-10-0-UI | Vasoactive intestinal polypeptide | BF550793 |
| 1190 | UI-R-C1-jx-f-05-0-UI | Cadherin 2 | AI044325 |
| 1191 | UI-R-C1-jz-a-03-0-UI | DiGeorge syndrome critical region gene 8 | AI045520 |
| 1192 | UI-R-C1-kc-b-05-0-UI | Kinesin-Like Protein family member (klp-6) | BF550946 |
| 1193 | UI-R-C1-kq-g-05-0-UI | RIKEN cDNA 2010305A19 | AI058886 |
| 1194 | UI-R-C1-ks-g-11-0-UI | XP_001111566.1 leucine zipper protein 1 | AI059074 |
| 1195 | UI-R-C1-kx-c-08-0-UI | CDNA clone IMAGE:7455180, containing frame-shift errors | AI058390 |
| 1196 | UI-R-C1-kz-e-10-0-UI | RIKEN cDNA B230118H07 | BF558459 |
| 1197 | UI-R-A0-al-f-12-0-UI | Pyruvate dehydrogenase (lipoamide) beta | AA819215 |
| 1198 | UI-R-A0-aq-a-01-0-UI | C1orf25 | AA819867 |
| 1199 | UI-R-A0-as-e-03-0-UI | Programmed cell death 6 | AA818950 |
| 1200 | UI-R-A0-be-c-11-0-UI | Hypoxia up-regulated 1 | AA858735 |
| 1201 | UI-R-E1-fo-g-09-0-UI | IBR domain containing 1 | BF556843 |
| 1202 | UI-R-E1-fu-a-12-0-UI | XP_001085723.1 thioredoxin domain containing 5 isoform 2 isoform 1 | AA957385 |
| 1203 | UI-R-E1-gq-a-11-0-UI | XP_001111479.1 interferon regulatory factor 2 binding protein 1 | AA964680 |
| 1204 | UI-R-C0-gw-c-06-0-UI | Transcribed locus | AA964074 |
| 1205 | UI-R-C0-ib-b-10-0-UI | XP_001110226.1 elongation protein 3 homolog isoform 2 | AA998202 |
| 1206 | UI-R-C0-id-f-06-0-UI | Nitrilase 1 | AA998079 |
| 1207 | UI-R-C0-gy-a-04-0-UI | Apolipoprotein B editing complex 1 | AA964261 |
| 1208 | UI-R-C0-he-e-06-0-UI | High mobility group protein 2 (HMG-2) | BF553901 |
| 1209 | UI-R-C0-jl-g-07-0-UI | Secreted phosphoprotein 2 | AI043655 |
| 1210 | UI-R-C1-js-c-11-0-UI | D123 gene product | AI043975 |
| 1211 | UI-R-C1-jx-f-06-0-UI | Potassium inwardly rectifying channel, subfamily J, member 11 | AI044326 |
| 1212 | UI-R-C1-ke-c-10-0-UI | Gap junction membrane channel protein beta 5 | AI044985 |
| 1213 | UI-R-C1-lp-b-03-0-UI | CDC42 binding protein kinase alpha | BF552275 |
| 1214 | UI-R-C2-mv-c-09-0-UI | RAB2, member RAS oncogene family | AI070618 |
| 1215 | UI-R-C2-ne-e-02-0-UI | Ras-related associated with diabetes | BF549637 |
| 1216 | UI-R-C0-ix-h-04-0-UI | Proprotein convertase subtilisin/kexin type 1 inhibitor | BF552723 |
| 1217 | UI-R-DK0-cfz-c-01-0-UI | CDC-like kinase 3 | BI296778 |
| 1218 | UI-R-DK0-cgf-c-09-0-UI | MTERF domain containing 2 | BI291085 |
| 1219 | UI-R-CV2-cgq-a-08-0-UI | RIKEN cDNA 1110004E09 | BI291418 |
| 1220 | UI-R-CV2-chc-f-09-0-UI | Integrin, beta 7 | BI297471 |
| 1221 | UI-R-DR0-cjc-j-19-0-UI | Lumican | BI304121 |
| 1222 | UI-R-A1-do-e-06-0-UI | Calpain 1 | BF555492 |
| 1223 | UI-R-A1-dt-e-12-0-UI | 4-hydroxyphenylpyruvic acid dioxygenase | AA924020 |
| 1224 | UI-R-A1-dw-g-01-0-UI | Fos-like antigen 1 | AA901256 |
| 1225 | UI-R-E1-gk-a-03-0-UI | LEYDIG CELL TUMOR 10 KD PROTEIN | AA963853 |
| 1226 | UI-R-C0-gu-g-12-0-UI | Coatomer protein complex, subunit beta 2 (beta prime) | AA964331 |
| 1227 | UI-R-DC0-bzm-c-06-0-UI | Seminal vesicle protein 4 | BI280591 |
| 1228 | UI-R-DD0-caa-d-04-0-UI | Ribosomal protein S20 | BI285458 |
| 1229 | UI-R-CW0s-cci-b-04-0-UI | Peroxisomal delta3, delta2-enoyl-Coenzyme A isomerase | BI287501 |
| 1230 | UI-R-CX0s-ccr-a-12-0-UI | Eukaryotic translation initiation factor 4A1 | BI284436 |
| 1231 | UI-R-CX0s-ccx-c-05-0-UI | HIV-1 tat interactive protein, homolog | BI283835 |
| 1232 | UI-R-DK0-cde-f-09-0-UI | Aldo-keto reductase family 1, member B10 (aldose reductase) | BI288765 |
| 1233 | UI-R-DK0-cev-f-10-0-UI | Zinc finger protein 688 | BI295549 |
| 1234 | UI-R-DK0-cfc-c-04-0-UI | Malignant T cell amplified sequence 1 | BI295915 |
| 1235 | UI-R-DK0-cfg-c-07-0-UI | Utrophin | BI289382 |
| 1236 | UI-R-CA1-bjt-b-02-0-UI | Transducin-like enhancer of split 4, E(spl) homolog | BF410063 |
| 1237 | UI-R-BT1-bmq-a-01-0-UI | Adenylate cyclase 8 | BF411712 |
| 1238 | UI-R-BT1-bnk-b-01-0-UI | 2410024A21Rik protein | BG379136 |
| 1239 | UI-R-CA0-bod-b-09-0-UI | Opsin 4 (melanopsin) | BF413019 |
| 1240 | UI-R-BJ2-boo-b-12-0-UI | RIKEN cDNA 1110061L23 | BF414252 |
| 1241 | UI-R-BJ2-bqm-e-09-0-UI | J domain protein 1 | BF419015 |
| 1242 | UI-R-BJ2-bqq-d-03-0-UI | Phytn_dehydro and Pyr_redox domain containing protein RGD1303232 | CK843797 |
| 1243 | UI-R-CW0-bvz-f-05-0-UI | Fibulin 2 | BI273844 |
| 1244 | UI-R-CW0-bwj-h-12-0-UI | Sorting nexin 7 | BI274534 |
| 1245 | UI-R-DA0-byj-e-11-0-UI | Adaptor-related protein complex 3, mu 1 subunit | BI279126 |
| 1246 | UI-R-DB0-byv-b-05-0-UI | Splicing factor 3b, subunit 3 | BI285007 |
| 1247 | UI-R-CA0-bge-g-01-0-UI | Neuro-oncological ventral antigen 1 | BF394766 |
| 1248 | UI-R-CA0-bgr-h-02-0-UI | Synovial sarcoma, X breakpoint 2 interacting protein | BF394350 |
| 1249 | UI-R-CA1-bjb-k-20-0-UI | Btg3 associated nuclear protein | BF399372 |
| 1250 | UI-R-CA1-bje-m-14-0-UI | Expressed sequence BB220380 | BF405819 |
| 1251 | UI-R-CV0-brl-g-10-0-UI | Crystallin, beta B2 | BG372058 |
| 1252 | UI-R-CV1-brz-e-11-0-UI | Amyloid beta (A4) precursor protein-binding, family B, member 3 | BG372706 |
| 1253 | UI-R-CT0-buk-e-03-0-UI | Acetylcholinesterase | BG381540 |
| 1254 | UI-R-CU0-bus-e-04-0-UI | Chloride channel Kb | BG376265 |
| 1255 | UI-R-CU0-bvd-c-04-0-UI | Pregnancy specific beta-1-glycoprotein 4 | BG377481 |
| 1256 | UI-R-CS0-bvm-f-09-0-UI | ADP-ribosylation factor-like 6 interacting protein 5 | BG377040 |
| 1257 | UI-R-CA0-axf-h-04-0-UI | UDP galactosyltransferase 8 | BE108577 |
| 1258 | UI-R-CA0-axs-a-11-0-UI | Dynein, cytoplasmic, light intermediate polypeptide 2 | BE115714 |
| 1259 | UI-R-BS1-ayg-a-09-0-UI | Kinesin family member 20A | BE116418 |
| 1260 | UI-R-BS1-ayt-f-06-0-UI | protein tyrosine phosphatase, receptor type, D | BE107433 |
| 1261 | UI-R-CA0-baw-g-03-0-UI | RWD domain containing 3 | BE121336 |
| 1262 | UI-R-CA1-bbi-b-03-0-UI | Abelson murine leukemia viral (v-abl) oncogene homolog 2 | BF386316 |
| 1263 | UI-R-CA1-bby-e-06-0-UI | Follistatin-like 1 | BF388124 |
| 1264 | UI-R-CA1-bcg-f-03-0-UI | Transcribed locus | BF390404 |
| 1265 | UI-R-BS2-bes-b-01-0-UI | GLI-Kruppel family member GLI3 | BF398755 |
| 1266 | UI-R-CA0-bfl-f-12-0-UI | hypothetical protein | BF392654 |
| 1267 | UI-R-BS0-aoe-f-09-0-UI | U2af1-rs2 | AW535394 |
| 1268 | UI-R-BU0-ape-b-04-0-UI | Radixin | BE096275 |
| 1269 | UI-R-BX0-arz-f-04-0-UI | Neuronal growth regulator 1 | BE105194 |
| 1270 | UI-R-BO1-asl-f-11-0-UI | Calsyntenin 1 | BE106247 |
| 1271 | UI-R-BJ1-asw-g-10-0-UI | Nth (endonuclease III)-like 1 (E.coli) | CK844828 |
| 1272 | UI-R-BJ1-atg-g-12-0-UI | Insulin induced gene 1 | BE098965 |
| 1273 | UI-R-BJ1-aut-f-03-0-UI | Protein tyrosine phosphatase, receptor type, A | BE109437 |
| 1274 | UI-R-BJ1-avg-a-03-0-UI | Retinoblastoma binding protein 7 | CK844125 |
| 1275 | UI-R-BJ1-avp-e-10-0-UI | Guanine nucleotide binding protein (G protein), gamma 11 | BE109559 |
| 1276 | UI-R-BJ1-avv-d-01-0-UI | Cytoskeleton associated protein 5 | CK845547 |
| 1277 | UI-R-BO0-aia-e-11-0-UI | Transcribed locus | AW524626 |
| 1278 | UI-R-BO0-aih-f-03-0-UI | Endothelin converting enzyme-like 1 | BF562978 |
| 1279 | UI-R-BJ0p-aip-h-03-0-UI | Copper chaperone for superoxide dismutase | BF562835 |
| 1280 | UI-R-BJ0p-aiv-b-06-0-UI | Thrombomodulin | CK842049 |
| 1281 | UI-R-BT1-ajy-d-02-0-UI | Acyl-CoA synthetase bubblegum family member 1 | AW527713 |
| 1282 | UI-R-BT1-akg-e-06-0-UI | Sodium channel, voltage-gated, type III, alpha polypeptide | AW527997 |
| 1283 | UI-R-BT1-akm-c-09-0-UI | ST8 alpha-N-acetyl-neuraminide alpha-2,8-sialyltransferase 5 | AW529294 |
| 1284 | UI-R-BT1-akv-d-05-0-UI | Mixed lineage kinase 4 | BF563910 |
| 1285 | UI-R-BU0-amv-d-07-0-UI | Amiloride-sensitive cation channel 4, pituitary | AW530335 |
| 1286 | UI-R-BU0-and-g-05-0-UI | Leucine zipper-EF-hand containing transmembrane protein 2 | BF562377 |
| 1287 | UI-R-AB1-yv-b-11-0-UI | Hypothetical protein LOC501706 | CK840863 |
| 1288 | UI-R-AE1-zc-c-12-0-UI | Proteasome (prosome, macropain) 26S subunit, ATPase 3, interacting protein | CK840952 |
| 1289 | UI-R-AF1-aay-g-12-0-UI | ERO1-like | CK845690 |
| 1290 | UI-R-Y0-abi-a-07-0-UI | XP_001086209.1 hypothetical protein | AI716517 |
| 1291 | UI-R-Y0-abv-f-06-0-UI | Leukotriene B4 receptor | AI764582 |
| 1292 | UI-R-Y0-ace-a-10-0-UI | Hypothetical protein RDA279 | AI764701 |
| 1293 | UI-R-BJ0p-afe-a-01-0-UI | Hyaluronan and proteoglycan link protein 1 | CK839256 |
| 1294 | UI-R-BJ0p-afl-e-01-0-UI | Vacuolar protein sorting 52 | AW434378 |
| 1295 | UI-R-BJ0p-afq-h-11-0-UI | Leucine-rich repeats and immunoglobulin-like domains 1 | AW433783 |
| 1296 | UI-R-BJ0p-aga-g-09-0-UI | Solute carrier family 6 (neurotransmitter transporter, taurine), member 6 | BF566991 |
| 1297 | UI-R-C2p-rn-d-08-0-UI | CCAAT/enhancer binding protein (C/EBP), alpha | BF545000 |
| 1298 | UI-R-C2p-rw-g-04-0-UI | CCAAT/enhancer binding protein (C/EBP), gamma | AI501108 |
| 1299 | UI-R-C2p-se-b-01-0-UI | Mss4 protein | BF542644 |
| 1300 | UI-R-C3-sn-e-06-0-UI | Transforming growth factor, beta receptor III | BF542698 |
| 1301 | UI-R-G0-ud-c-07-0-UI | phosphatidylinositol transfer protein, cytoplasmic 1 isoform a | BF523312 |
| 1302 | UI-R-G0-uq-b-04-0-UI | Dihydrofolate reductase | BF525301 |
| 1303 | UI-R-Y0-uz-d-08-0-UI | expressed sequence AV340375 | AI575940 |
| 1304 | UI-R-Y0-vm-b-01-0-UI | Cytochrome P450, family 2, subfamily b, polypeptide 13 | AI576382 |
| 1305 | UI-R-AF0-yc-b-01-0-UI | Mucosal vascular addressin cell adhesion molecule 1 | BF525141 |
| 1306 | UI-R-AC0-ym-d-05-0-UI | Y box protein 1 | CK838876 |
| 1307 | UI-R-E1-gb-h-05-0-UI | DOT1-like, histone H3 methyltransferase | AA957531 |
| 1308 | UI-R-E1-gk-h-05-0-UI | RIKEN cDNA 4931406C07 | AA963801 |
| 1309 | UI-R-C0-jm-a-06-0-UI | Pleckstrin homology domain containing, family A member 5 | AI043834 |
| 1310 | UI-R-C1-kh-h-04-0-UI | Hydroxyacid oxidase 2 (long chain) | AI045346 |
| 1311 | UI-R-C1-lb-e-01-0-UI | XP_001113557.1 ataxin-1 ubiquitin-like interacting protein | AI502674 |
| 1312 | UI-R-C1-lr-b-07-0-UI | Dimethylarginine dimethylaminohydrolase 1 | AI058941 |
| 1313 | UI-R-C2p-oj-f-10-0-UI | E74-like factor 2 | AI137223 |
| 1314 | UI-R-BT0-pq-b-11-0-UI | Phosphatidylinositol (4,5) bisphosphate 5-phosphatase, A | BF544285 |
| 1315 | UI-R-BT0-pv-e-02-0-UI | Vacuolar protein sorting 16 | AI145204 |
| 1316 | UI-R-BT0-qf-c-08-0-UI | PRP4 pre-mRNA processing factor 4 homolog B | BF545898 |
| 1317 | UI-R-A0-ar-e-04-0-UI | Solute carrier family 13 (sodium/sulfate symporters), member 1 | BF549840 |
| 1318 | UI-R-A0-bc-d-03-0-UI | Alpha glucosidase 2 alpha neutral subunit | BF559000 |
| 1319 | UI-R-E0-bo-h-04-0-UI | Nucleoside phosphorylase | AA858461 |
| 1320 | UI-R-E0-bt-h-06-0-UI | Ubiquitin-conjugating enzyme E2E 3, UBC4/5 homolog | BF547744 |
| 1321 | UI-R-E0-cz-g-04-0-UI | Phosphate regulating gene with homologies to endopeptidases on the X chromosome (hypophosphatemia, vitamin D resistant rickets) | AA899251 |
| 1322 | UI-R-E0-dg-c-07-0-UI | Mitochondrial acyl-CoA thioesterase 1 | AA899721 |
| 1323 | UI-R-A1-do-e-05-0-UI | Tetratricopeptide repeat domain 7B | CK845718 |
| 1324 | UI-R-A1-ds-f-04-0-UI | XP_001109807.1 craniofacial development protein 1 isoform 1 | AA924257 |
| 1325 | UI-R-A1-ew-b-11-0-UI | Sperm specific antigen 2 | BF560076 |
| 1326 | UI-R-E1-fi-d-02-0-UI | Lipase, hepatic | AA956221 |
| 1327 | UI-R-CV1-bvt-e-04-0-UI | Sarcoglycan, alpha (dystrophin-associated glycoprotein) | BG378841 |
| 1328 | UI-R-DK0-cdf-e-05-0-UI | Mitochondrial ATP synthase regulatory component factor B | BI288841 |
| 1329 | UI-R-E1-gh-g-10-0-UI | WD repeat domain 35 | AA963275 |
| 1330 | UI-R-C0-hr-d-12-0-UI | hypothetical protein DKFZp434H0115 | AA997115 |
| 1331 | UI-R-C0-jb-h-08-0-UI | Ets variant gene 6 (TEL oncogene) | BF546884 |
| 1332 | UI-R-C1-kx-f-04-0-UI | hypothetical protein | BF523604 |
| 1333 | UI-R-C2p-re-b-02-0-UI | Oxoglutarate dehydrogenase-like | AI555824 |
| 1334 | UI-R-C2p-rt-e-12-0-UI | Desmoglein 1 gamma | AI501187 |
| 1335 | UI-R-C0-ir-h-01-0-UI | Glycyl-tRNA synthetase | BF554526 |
| 1336 | UI-R-C0-jb-d-05-0-UI | 2810027O19Rik protein | BF551773 |
| 1337 | UI-R-C1-km-d-12-0-UI | Hypothetical protein LOC685233 | AI045854 |
| 1338 | UI-R-C1-ku-h-05-0-UI | Friend leukemia integration 1 | BF547037 |
| 1339 | UI-R-C1-lk-b-03-0-UI | Enthoprotin | BF546748 |
| 1340 | UI-R-C1-ln-h-12-0-UI | Nudix (nucleoside diphosphate linked moiety X)-type motif 14 | AI070062 |
| 1341 | UI-R-C0-he-e-08-0-UI | V-myc myelocytomatosis viral related oncogene, neuroblastoma derived | BF557860 |
| 1342 | UI-R-C0-je-b-02-0-UI | G-protein signalling modulator 1 (AGS3-like, C. elegans) | AI030808 |
| 1343 | UI-R-C1-kv-f-11-0-UI | 0610007L01Rik protein | BF547068 |
| 1344 | UI-R-C2-mr-e-11-0-UI | hypothetical protein FLJ23518 | BF542261 |
| 1345 | UI-R-C0-ih-d-07-0-UI | Mitochondrial tumor suppressor 1 | AA999004 |
| 1346 | UI-R-C0-jd-g-05-0-UI | Solute carrier family 9 (sodium/hydrogen exchanger), isoform 3 regulator 1 | AI030754 |
| 1347 | UI-R-C1-kl-e-01-0-UI | Transcribed locus | AI045767 |
| 1348 | UI-R-Y0-ls-d-04-0-UI | Hydroxysteroid (17-beta) dehydrogenase 12 | AI070081 |
| 1349 | UI-R-E1-fa-f-09-0-UI | Cleft lip and palate associated transmembrane protein 1 | AA955534 |
| 1350 | UI-R-E1-fr-h-03-0-UI | CAMP responsive element binding protein 3-like 2 | AA956791 |
| 1351 | UI-R-E1-fx-a-05-0-UI | cofactor required for Sp1 transcriptional activation subunit 8 | AA963072 |
| 1352 | UI-R-E1-gk-c-06-0-UI | Serine hydroxymethyltransferase 1 (soluble) | AA963876 |
| 1353 | UI-R-C0-ih-g-02-0-UI | Hypothetical protein LOC691300 | AA998712 |
| 1354 | UI-R-C0-in-b-08-0-UI | Polymerase (DNA-directed), delta 4 | BF551348 |
| 1355 | UI-R-A1-es-h-10-0-UI | Cathepsin S | AA925933 |
| 1356 | UI-R-A1-ey-g-03-0-UI | Phospholipase A2, group IB | AA955059 |
| 1357 | UI-R-C0-ip-c-11-0-UI | RAB geranylgeranyl transferase, b subunit | AI029123 |
| 1358 | UI-R-Y0-lu-g-04-0-UI | Crystallin, beta B3 | AI070226 |
| 1359 | UI-R-BT0-ps-g-10-0-UI | Protein kinase, cAMP dependent regulatory, type I, alpha | AI145654 |
| 1360 | UI-R-A0-ay-b-08-0-UI | LanC (bacterial lantibiotic synthetase component C)-like 1 | BF549735 |
| 1361 | UI-R-C1-ln-e-07-0-UI | Transcribed locus | AI070040 |
| 1362 | UI-R-A0-ax-h-11-0-UI | Kidney-specific protein (KS) | BF549727 |
| 1363 | UI-R-E0-cf-h-10-0-UI | Pleiomorphic adenoma gene-like 1 | BF550365 |
| 1364 | UI-R-A1-ew-d-08-0-UI | Serine (or cysteine) peptidase inhibitor, clade I, member 2 | AI146193 |
| 1365 | UI-R-C2-ng-b-06-0-UI | H2A histone family, member V isoform 1 | AI072310 |
| 1366 | UI-R-C2-ni-e-02-0-UI | XP_001101105.1 PRP3 pre-mRNA processing factor 3 homolog isoform 3 | AI072539 |
| 1367 | UI-R-C2-nk-d-08-0-UI | Dystonia 1 | AI072675 |
| 1368 | UI-R-C2-nm-g-10-0-UI | Alpha 1,4-galactosyltransferase | BF552844 |
| 1369 | UI-R-A0-ak-h-04-0-UI | Mitogen-activated protein kinase 6 | AA859081 |
| 1370 | UI-R-A0-aw-e-04-0-UI | Vascular endothelial growth factor A | BF547545 |
| 1371 | UI-R-A0-bd-f-02-0-UI | Sulfotransferase family 1A, phenol-preferring, member 1 | AA866493 |
| 1372 | UI-R-A0-bl-f-12-0-UI | Ras homolog gene family, member B | AA819420 |
| 1373 | UI-R-E0-ct-h-07-0-UI | Internexin, alpha | AA875659 |
| 1374 | UI-R-E0-dd-h-04-0-UI | S100 calcium binding protein A10 (calpactin) | AA900235 |
| 1375 | UI-R-C0-jh-g-11-0-UI | TNFRSF1A-associated via death domain | AI030632 |
| 1376 | UI-R-C0-jl-e-06-0-UI | Putatative 28 kDa protein | AI043694 |
| 1377 | UI-R-C1-ke-c-06-0-UI | BCL2/adenovirus E1B 19 kDa-interacting protein 3-like | BF546474 |
| 1378 | UI-R-C1-kh-c-01-0-UI | MAP/microtubule affinity-regulating kinase 4 | AI045317 |
| 1379 | UI-R-C1-kj-g-02-0-UI | RIKEN cDNA 0610016J10 gene | AI045595 |
| 1380 | UI-R-C1-kp-c-01-0-UI | XP_001113525.1 myosin, light polypeptide kinase isoform 7 | AI058729 |
| 1381 | UI-R-C1-ld-d-06-0-UI | Transcribed locus | AI059244 |
| 1382 | UI-R-C1-lf-g-05-0-UI | PHD finger protein 3 | AI059342 |
| 1383 | UI-R-C1-lp-c-04-0-UI | expressed sequence AU021034 | AI069989 |
| 1384 | UI-R-Y0-lv-b-04-0-UI | Dispatched homolog 2 | AI070328 |
| 1385 | UI-R-A1-ez-f-10-0-UI | Phosphatidylinositol glycan, class Q | AI146091 |
| 1386 | UI-R-E1-ff-g-12-0-UI | Peroxisome proliferative activated receptor, gamma, coactivator-related 1 | AA955877 |
| 1387 | UI-R-E1-fk-d-10-0-UI | RIKEN cDNA 4922503N01 | AA956597 |
| 1388 | UI-R-E1-fm-h-11-0-UI | Non-catalytic region of tyrosine kinase adaptor protein 2 | BF556830 |
| 1389 | UI-R-C0-hd-b-12-0-UI | Heterogeneous nuclear ribonucleoprotein K | AA965265 |
| 1390 | UI-R-C0-hh-e-04-0-UI | RIKEN cDNA 1500041N16 | BF561240 |
| 1391 | UI-R-C0-ht-g-05-0-UI | Calmodulin 1 | AI137692 |
| 1392 | UI-R-C0-hv-g-01-0-UI | Leucyl-tRNA synthetase | CK840159 |
| 1393 | UI-R-C0-iu-g-09-0-UI | Serine/arginine-rich protein specific kinase 2 | BF551587 |
| 1394 | UI-R-C0-ja-d-04-0-UI | XP_001104117.1 spindle pole body component 24 homolog | BF551759 |
| 1395 | UI-R-C0-hw-d-02-0-UI | CD63 antigen | AA997566 |
| 1396 | UI-R-C0-jd-b-02-0-UI | PDZ and LIM domain 3 | AI030725 |
| 1397 | UI-R-C1-kl-e-04-0-UI | Calcium channel, voltage-dependent, P/Q type, alpha 1A subunit | AI045770 |
| 1398 | UI-R-C1-ku-b-02-0-UI | Multiple inositol polyphosphate histidine phosphatase 1 | AI071438 |
| 1399 | UI-R-C1-la-b-09-0-UI | Aryl hydrocarbon receptor nuclear translocator-like | AI059871 |
| 1400 | UI-R-C1-li-c-08-0-UI | Thyroid stimulating hormone receptor | BF546708 |
| 1401 | UI-R-C2p-nz-g-09-0-UI | Microtubule-associated protein 6 | BF553730 |
| 1402 | UI-R-C2p-om-g-05-0-UI | Guanosine monophosphate reductase | AI138122 |
| 1403 | UI-R-A0-ac-g-10-0-UI | RIKEN cDNA 0610037P05 | AA866307 |
| 1404 | UI-R-A0-ah-h-08-0-UI | Transcribed locus | AA818238 |
| 1405 | UI-R-CV2-cif-a-09-0-UI | hypothetical protein D630010C10 | BI300442 |
| 1406 | UI-R-DN0-cit-j-18-0-UI | Fucosidase, alpha-L- 2, plasma | BI291823 |
| 1407 | UI-R-DN0-civ-n-10-0-UI | Cadherin 17 | BI292457 |
| 1408 | UI-R-DQ0-ciz-d-15-0-UI | Translocated promoter region | BI303240 |
| 1409 | UI-R-A1-ea-h-06-0-UI | XP_001101685.1 kininogen 1 isoform 1 | BF559178 |
| 1410 | UI-R-A1-ee-c-07-0-UI | FK506 binding protein 1a | AA925361 |
| 1411 | UI-R-A1-ei-b-12-0-UI | General transcription factor IIB | BF555384 |
| 1412 | UI-R-A1-eo-h-12-0-UI | Ribosomal protein L39 | AA925893 |
| 1413 | UI-R-C0-hl-e-03-0-UI | G protein-coupled receptor kinase interactor 1 | AA997450 |
| 1414 | UI-R-C0-hs-c-11-0-UI | 2',5'-oligoadenylate synthetase 1, 40/46kDa | BF550665 |
| 1415 | UI-R-CU0s-cbw-g-08-0-UI | Mitochondrial ribosomal protein L36 | BI282120 |
| 1416 | UI-R-CW0s-ccb-g-08-0-UI | Chondroitin sulfate proteoglycan 4 | BI285578 |
| 1417 | UI-R-DK0-cdq-g-11-0-UI | RIKEN cDNA 1200014M14 | BI293466 |
| 1418 | UI-R-DK0-ceb-a-02-0-UI | Membrane-associated DHHC5 zinc finger protein | BI294693 |
| 1419 | UI-R-DK0-cee-a-03-0-UI | COMM domain containing 8 | BI294948 |
| 1420 | UI-R-DK0-ceg-f-01-0-UI | Msx2-interacting protein (SPEN homolog) | BI295169 |
| 1421 | UI-R-BJ2-bqw-e-03-0-UI | Sirtuin 5 (silent mating type information regulation 2 homolog) 5 | BF407170 |
| 1422 | UI-R-BJ2-brd-a-06-0-UI | Mediator of DNA damage checkpoint 1 | BF407741 |
| 1423 | UI-R-DK0-cfi-b-07-0-UI | RT1 class II, locus Da | BI289622 |
| 1424 | UI-R-DK0-cfr-h-10-0-UI | Aldehyde dehydrogenase family 1, subfamily A7 | BI290108 |
| 1425 | UI-R-BJ2-box-f-02-0-UI | Proteasome (prosome, macropain) 26S subunit, ATPase, 6 | CK843070 |
| 1426 | UI-R-BJ2-bpk-g-08-0-UI | ST8 alpha-N-acetyl-neuraminide alpha-2,8-sialyltransferase 4 | CK844620 |
| 1427 | UI-R-BJ2-bps-h-12-0-UI | Transcribed locus | BF406986 |
| 1428 | UI-R-BJ2-bqh-b-12-0-UI | Protein kinase C and casein kinase substrate in neurons 1 | CK843676 |
| 1429 | UI-R-CX0-bwx-b-03-0-UI | Large subunit ribosomal protein L36a | BI278807 |
| 1430 | UI-R-CX0-bxi-d-12-0-UI | Deoxynucleotidyltransferase, terminal, interacting protein 2 | BI275956 |
| 1431 | UI-R-CY0-bxp-a-02-0-UI | Phosphohistidine phosphatase 1 | BI276995 |
| 1432 | UI-R-CZ0-byb-d-06-0-UI | Vascular endothelial zinc finger 1 | BI277775 |
| 1433 | UI-R-CT0s-car-c-10-0-UI | AU RNA binding protein/enoyl-coenzyme A hydratase | BI286749 |
| 1434 | UI-R-CS0s-cbn-e-07-0-UI | Transcobalamin 2 | BI287325 |
| 1435 | UI-R-CA1-bih-h-02-0-UI | Potassium voltage gated channel, Shaw-related subfamily, member 2 | BF404636 |
| 1436 | UI-R-CA1-bix-d-09-0-UI | Dnaj-like protein | BF403814 |
| 1437 | UI-R-CV1-bsl-b-10-0-UI | Membrane protein, palmitoylated 4 (MAGUK p55 subfamily member 4) | BG373990 |
| 1438 | UI-R-CV1-bta-a-03-0-UI | Serine incorporator 1 | BG374610 |
| 1439 | UI-R-CS0-btt-b-04-0-UI | RIKEN cDNA 2810428I15 | BG381352 |
| 1440 | UI-R-CT0-btz-g-06-0-UI | 3-hydroxy-3-methylglutaryl-Coenzyme A reductase | BG380649 |
| 1441 | UI-R-CA1-bjr-f-11-0-UI | Trinucleotide repeat containing 6 | BF409864 |
| 1442 | UI-R-CA0-bkq-d-11-0-UI | Zinc finger protein 672 | BF416119 |
| 1443 | UI-R-CN0-bld-c-09-0-UI | NECAP endocytosis associated 2 | BF417361 |
| 1444 | UI-R-CN0-blt-a-10-0-UI | Fasting-inducible integral membrane protein TM6P1 | BF418150 |
| 1445 | UI-R-BS1-azb-b-09-0-UI | XP_001094087.1 Protein KIAA1219 | BE109118 |
| 1446 | UI-R-BS1-azn-d-12-0-UI | Insulin receptor substrate 2 | BE118080 |
| 1447 | UI-R-CA0-azz-f-12-0-UI | Proprotein convertase subtilisin/kexin type 1 | BE119208 |
| 1448 | UI-R-CA0-bao-f-08-0-UI | Potassium voltage gated channel, Shal-related family, member 3 | BE120020 |
| 1449 | UI-R-CA1-bcx-f-05-0-UI | Nuclear transcription factor-Y beta | BF391453 |
| 1450 | UI-R-BS2-bdj-h-09-0-UI | Paired box gene 6 | BF389448 |
| 1451 | UI-R-BS2-bdv-f-10-0-UI | Epidermal Langerhans cell protein LCP1 | BF396181 |
| 1452 | UI-R-BS2-bec-e-04-0-UI | Calcitonin gene-related peptide-receptor component protein | BF397860 |
| 1453 | UI-R-CA0-bhc-g-07-0-UI | N-acetyl galactosaminidase, alpha | BF394262 |
| 1454 | UI-R-CA0-bhr-d-09-0-UI | Glucokinase | BF402525 |
| 1455 | UI-R-BT1-aqn-d-06-0-UI | Eph receptor A7 | BE102501 |
| 1456 | UI-R-BX0-are-g-08-0-UI | XP_001117363.1 potassium inwardly-rectifying channel subfamily J9 | BE103125 |
| 1457 | UI-R-BJ1-ato-e-03-0-UI | Endothelial differentiation, lysophosphatidic acid G-protein-coupled receptor 4 | CK842936 |
| 1458 | UI-R-BJ1-ats-h-03-0-UI | RIKEN cDNA 1200011I18 | CK841869 |
| 1459 | UI-R-BJ1-aua-g-07-0-UI | Thymidylate kinase family LPS-inducible member | CK839410 |
| 1460 | UI-R-BJ1-aui-b-11-0-UI | Cullin-associated and neddylation-dissociated 2 | BE101279 |
| 1461 | UI-R-BJ1-awa-g-04-0-UI | Transcribed locus | BE112953 |
| 1462 | UI-R-BJ1-awg-d-02-0-UI | polybromo-1 | BE113614 |
| 1463 | UI-R-BJ1-awq-a-06-0-UI | BRCA2 and CDKN1A interacting protein | BE114844 |
| 1464 | UI-R-CA0-awy-e-11-0-UI | Solute carrier family 6 (neurotransmitter transporter, dopamine), member 3 | BE107942 |
| 1465 | UI-R-BO1-ajb-h-08-0-UI | Regulating synaptic membrane exocytosis 2 | AW526422 |
| 1466 | UI-R-BO1-aji-e-03-0-UI | NP_071707.1 R | BF563291 |
| 1467 | UI-R-BO1-ajm-c-07-0-UI | XP_001085913.1 hypothetical protein | AW527166 |
| 1468 | UI-R-BO1-ajs-g-04-0-UI | Kelch domain containing 8A | AW527302 |
| 1469 | UI-R-C4-ald-c-07-0-UI | Tripartite motif protein 23 | BF564085 |
| 1470 | UI-R-C4-all-c-11-0-UI | Hypothetical protein XP_237985 | AW532259 |
| 1471 | UI-R-C4-alr-e-12-0-UI | Prolactin family 3, subfamily a, member 1 | AW533989 |
| 1472 | UI-R-BT1-amf-h-07-0-UI | RIKEN cDNA 1700088E04 | BE096976 |
| 1473 | UI-R-BO1-apo-e-10-0-UI | Myelin transcription factor 1-like | BE095649 |
| 1474 | UI-R-BO1-aqb-h-10-0-UI | Formin binding protein 1 | BE097806 |
| 1475 | UI-R-AA1-aac-g-01-0-UI | Peptidylprolyl isomerase (cyclophilin)-like 3 | AI706907 |
| 1476 | UI-R-AF1-aas-b-02-0-UI | LSM8 homolog, U6 small nuclear RNA associated | AI712577 |
| 1477 | UI-R-Y0-aco-d-01-0-UI | Optineurin | AI717374 |
| 1478 | UI-R-BJ0-acz-a-11-0-UI | TAF6-like RNA polymerase II, p300/CBP-associated factor (PCAF)-associated factor | AW253760 |
| 1479 | UI-R-BJ0-adp-b-12-0-UI | AT rich interactive domain 1A (Swi1 like) | AW251646 |
| 1480 | UI-R-BJ0-ael-g-06-0-UI | PE responsive protein c64 | AW252878 |
| 1481 | UI-R-BO0-agn-a-04-0-UI | Glutamate receptor, ionotropic, kainate 4 | BF566550 |
| 1482 | UI-R-BO0-agr-d-07-0-UI | Solute carrier family 22 (organic cation transporter), member 5 | BF567603 |
| 1483 | UI-R-BO0-ahl-a-06-0-UI | Unc-13 homolog C | BF567769 |
| 1484 | UI-R-BO0-aht-h-08-0-UI | Cholinergic receptor, muscarinic 3 | BF567252 |
| 1485 | UI-R-C3-sv-h-05-0-UI | Transcribed locus | BF543605 |
| 1486 | UI-R-C3-ti-c-06-0-UI | Signal recognition particle receptor, B subunit | BF523222 |
| 1487 | UI-R-C3-tn-c-03-0-UI | Heart and neural crest derivatives expressed transcript 2 | AI709519 |
| 1488 | UI-R-C3-tv-h-08-0-UI | RIKEN cDNA 1500031M22 | AI549047 |
| 1489 | UI-R-AD0-wb-e-09-0-UI | Nitrogen fixation gene 1 | CK838567 |
| 1490 | UI-R-AA0-wp-a-07-0-UI | Calcium and integrin binding 1 (calmyrin) | CK844705 |
| 1491 | UI-R-AG0-wy-b-11-0-UI | Protein regulator of cytokinesis 1 | AI579415 |
| 1492 | UI-R-AE0-xm-g-03-0-UI | Paladin | AI602873 |
| 1493 | UI-R-AD1-zn-h-02-0-UI | ARP1 actin-related protein 1 homolog A | CK839701 |
| 1494 | UI-R-AD1-zt-g-10-0-UI | Transforming, acidic coiled-coil containing protein 3 | CK841048 |
| 1495 | UI-R-C0-hw-a-12-0-UI | Solute carrier family 4 (anion exchanger), member 1, adaptor protein | CK840160 |
| 1496 | UI-R-C0-io-a-11-0-UI | XP_001103469.1 RAB11 family interacting protein 5 (class I) | AI028935 |
| 1497 | UI-R-Y0-mk-c-01-0-UI | Formiminotransferase cyclodeaminase | AI112668 |
| 1498 | UI-R-C2-mu-g-07-0-UI | Extra spindle poles like 1 | AI385371 |
| 1499 | UI-R-C2p-nr-f-03-0-UI | RIKEN cDNA 5230400G24 | AI113129 |
| 1500 | UI-R-C2p-od-a-05-0-UI | ATP-binding cassette, sub-family G (WHITE), member 3 | BF551971 |
| 1501 | UI-R-BT0-qk-g-06-0-UI | Cholecystokinin B receptor | AI454701 |
| 1502 | UI-R-C2p-qq-d-12-0-UI | XP_001091528.1 testis intracellular mediator protein isoform 8 | AI555466 |
| 1503 | UI-R-C2p-qv-d-04-0-UI | F-box only protein 32 | AI555032 |
| 1504 | UI-R-C2p-rg-f-10-0-UI | FMS-like tyrosine kinase 3 | BF523018 |
| 1505 | UI-R-E0-bx-h-07-0-UI | Signal recognition particle 68 | AA859720 |
| 1506 | UI-R-E0-cd-d-01-0-UI | Cell division cycle associated 7 | AA859235 |
| 1507 | UI-R-E0-ck-f-07-0-UI | AKT1 substrate 1 (proline-rich) | AA874908 |
| 1508 | UI-R-E0-ct-c-08-0-UI | Transformed mouse 3T3 cell double minute 2 homolog | AA875509 |
| 1509 | UI-R-A1-dx-e-11-0-UI | RIKEN cDNA 1200013B08 | BF555964 |
| 1510 | UI-R-A1-ec-c-02-0-UI | hypothetical protein D4Ertd89e | AA925702 |
| 1511 | UI-R-A1-eh-b-06-0-UI | Protein C20orf103 precursor | BF555774 |
| 1512 | UI-R-A1-el-g-09-0-UI | Kell protein | AA926320 |
| 1513 | UI-R-C0-ha-c-12-0-UI | Inner nuclear membrane protein Man1 | AA964659 |
| 1514 | UI-R-C0-hp-h-06-0-UI | XP_001089256.1 GREB1 protein isoform a isoform 1 | AA997724 |
| 1515 | UI-R-C2p-nz-e-11-0-UI | Choline dehydrogenase | AI136972 |
| 1516 | UI-R-AB0-vv-f-12-0-UI | DCN1, defective in cullin neddylation 1, domain containing 5 | AI577019 |
| 1517 | UI-R-BO0-aie-h-04-0-UI | Downstream neighbor of SON | AW523951 |
| 1518 | UI-R-BJ1-avn-a-01-0-UI | Transmembrane protein 17 | CK842575 |
| 1519 | UI-R-A0-bl-e-08-0-UI | S100 calcium binding protein A11 (calizzarin) | BF555263 |
| 1520 | UI-R-E0-cj-d-06-0-UI | XP_001117725.1 spinster-like | AA859323 |
| 1521 | UI-R-A1-dq-e-05-0-UI | DNA methyltransferase 2 | AA923969 |
| 1522 | UI-R-A1-el-d-10-0-UI | 2610034M16Rik protein | AA926066 |
| 1523 | UI-R-Y0-lx-c-02-0-UI | hypothetical protein FLJ32658 | AI073050 |
| 1524 | UI-R-C2p-nt-f-07-0-UI | Transcribed locus | AI113270 |
| 1525 | UI-R-C0-ha-h-12-0-UI | XP_001114732.1 flavin adenine dinucleotide synthetase isoform 1 isoform 2 | AA965069 |
| 1526 | UI-R-C0-hg-c-03-0-UI | Retinol dehydrogenase 11 | BF561196 |
| 1527 | UI-R-C0-jd-c-03-0-UI | ATP synthase mitochondrial F1 complex assembly factor 1 | AI030731 |
| 1528 | UI-R-C0-jm-g-10-0-UI | exonuclease NEF-sp | AI043782 |
| 1529 | UI-R-C0-jr-g-11-0-UI | Emopamil binding protein-like | BF545308 |
| 1530 | UI-R-C1-ju-f-03-0-UI | Sirtuin 7 (silent mating type information regulation 2, homolog) 7 | AI045296 |
| 1531 | UI-R-A0-au-h-06-0-UI | Alanine-glyoxylate aminotransferase 2 | AA818440 |
| 1532 | UI-R-C0-gx-a-04-0-UI | Transcribed locus | AA964809 |
| 1533 | UI-R-A0-bi-f-05-0-UI | Transcribed locus | AA858911 |
| 1534 | UI-R-A0-aw-h-04-0-UI | doublecortin-like kinase 2 | AA818566 |
| 1535 | UI-R-E1-fj-e-06-0-UI | Parkinson disease (autosomal recessive, early onset) 7 | AA956407 |
| 1536 | UI-R-Y0-lu-h-07-0-UI | RT1 class Ib, locus Aw2 | BF551911 |
| 1537 | UI-R-A1-er-h-06-0-UI | Membrane associated guanylate kinase, WW and PDZ domain containing 3 | AA925595 |
| 1538 | UI-R-E1-fx-d-02-0-UI | Solute carrier family 38, member 2 | AA963102 |
| 1539 | UI-R-C2-ms-f-09-0-UI | Translocase of outer mitochondrial membrane 40 homolog | AI071108 |
| 1540 | UI-R-C0-ii-e-04-0-UI | Brain expressed myelocytomatosis oncogene | AA998830 |
| 1541 | UI-R-A0-ax-a-01-0-UI | polymerase (RNA) III (DNA directed) (155kD) | BF549865 |
| 1542 | UI-R-A0-ba-b-10-0-UI | Actin related protein 2/3 complex, subunit 5 | BF555353 |
| 1543 | UI-R-E1-gl-a-06-0-UI | Ribonuclease, RNase A family, 1 (pancreatic) | AA964218 |
| 1544 | UI-R-C0-gv-e-03-0-UI | XP_001112869.1 gemin 5 isoform 4 | AA964499 |
| 1545 | UI-R-E0-cb-f-09-0-UI | Solute carrier family 2 (facilitated glucose transporter), member 1 | AA875020 |
| 1546 | UI-R-E0-ch-f-01-0-UI | Neural precursor cell expressed, developmentally down-regulated gene 8 | AA866433 |
| 1547 | UI-R-E1-fc-f-12-0-UI | Apolipoprotein C-I | AA955662 |
| 1548 | UI-R-E1-fn-h-10-0-UI | Dynein, cytoplasmic, intermediate chain 2 | BF560972 |
| 1549 | UI-R-E1-fx-e-09-0-UI | Glucagon receptor | AA962949 |
| 1550 | UI-R-C0-hz-e-11-0-UI | XP_001097639.1 granzyme A (granzyme 1, cytotoxic T-lymphocyte-associated serine esterase 3) | AA998292 |
| 1551 | UI-R-A1-es-b-01-0-UI | Endothelial PAS domain protein 1 | CK840117 |
| 1552 | UI-R-E1-fy-h-12-0-UI | Serine/arginine repetitive matrix 1 | BF562012 |
| 1553 | UI-R-C0-hg-f-02-0-UI | Solute carrier family 23 (nucleobase transporters), member 1 | BF561211 |
| 1554 | UI-R-C0-je-f-11-0-UI | Nuclear receptor coactivator 4 | AI031008 |
| 1555 | UI-R-Y0-lv-g-09-0-UI | MT-protocadherin | BF521772 |
| 1556 | UI-R-C2-mw-e-10-0-UI | FK506 binding protein 14 | AI071633 |
| 1557 | UI-R-C2-nb-a-02-0-UI | Stearoyl-Coenzyme A desaturase 2 | AI071229 |
| 1558 | UI-R-C2-nd-b-06-0-UI | RIKEN cDNA 9430023L20 | AI071995 |
| 1559 | UI-R-C2-nn-f-04-0-UI | CD244 natural killer cell receptor 2B4 | AI072627 |
| 1560 | UI-R-C2p-ns-c-10-0-UI | Transcribed locus | BF553320 |
| 1561 | UI-R-C2p-nw-d-07-0-UI | Neuralized-like 2 | AI136724 |
| 1562 | UI-R-C2p-ny-f-03-0-UI | t-complex 11 protein | BF552343 |
| 1563 | UI-R-E0-bo-d-10-0-UI | Complement component 1, q subcomponent binding protein | AA858513 |
| 1564 | UI-R-E0-bv-e-07-0-UI | 7-dehydrocholesterol reductase | AA859471 |
| 1565 | UI-R-C0-if-b-08-0-UI | ATP-binding cassette, sub-family A (ABC1), member 2 | AA998560 |
| 1566 | UI-R-C0-ij-g-02-0-UI | Transcribed locus | AI028856 |
| 1567 | UI-R-C0-jp-b-02-0-UI | CCCTC-binding factor | BF550973 |
| 1568 | UI-R-C1-jw-h-12-0-UI | XP_001111390.1 phosphofurin acidic cluster sorting protein 1 | AI044507 |
| 1569 | UI-R-C1-jy-c-09-0-UI | G protein-coupled receptor 19 | AI045718 |
| 1570 | UI-R-C1-kb-c-08-0-UI | Bcl2-associated X protein | BF542229 |
| 1571 | UI-R-C1-kq-b-07-0-UI | Notchless homolog 1 | AI059154 |
| 1572 | UI-R-C1-kr-f-05-0-UI | Chromatin assembly factor 1, subunit B (p60) | AI059934 |
| 1573 | UI-R-C1-kw-c-04-0-UI | Interleukin 2 receptor, gamma (severe combined immunodeficiency) | AI059518 |
| 1574 | UI-R-C1-ky-d-02-0-UI | novel protein | BF546399 |
| 1575 | UI-R-A0-aj-e-01-0-UI | CDNA clone IMAGE:7374368 | BF548732 |
| 1576 | UI-R-A0-am-e-12-0-UI | ATPase family, AAA domain containing 3A | BF549139 |
| 1577 | UI-R-A0-aq-g-07-0-UI | Zinc finger CCCH type, antiviral 1 | BF549169 |
| 1578 | UI-R-A0-au-h-12-0-UI | RIKEN cDNA 1700034M03 gene | AA818446 |
| 1579 | UI-R-E1-fn-f-12-0-UI | Huntingtin interacting protein 2 | AI112792 |
| 1580 | UI-R-E1-fq-e-03-0-UI | Coiled-coil domain containing 53 | AA957265 |
| 1581 | UI-R-E1-fu-h-03-0-UI | Growth arrest specific 6 | BF556153 |
| 1582 | UI-R-C0-gr-a-09-0-UI | TERF1 (TRF1)-interacting nuclear factor 2 | AA964858 |
| 1583 | UI-R-C0-hz-d-11-0-UI | Dymeclin | BF561467 |
| 1584 | UI-R-C0-ic-a-08-0-UI | expressed sequence C77668 | AA998514 |
| 1585 | UI-R-C0-gw-a-09-0-UI | Histone cluster 1, H2bl | AA964055 |
| 1586 | UI-R-C0-hb-b-11-0-UI | Annexin A1 | BF557705 |
| 1587 | UI-R-C0-jg-g-03-0-UI | Death effector domain-containing | AI045697 |
| 1588 | UI-R-C0-jo-b-09-0-UI | Ribosomal protein L30 | AI030687 |
| 1589 | UI-R-C1-jv-c-02-0-UI | Arachidonate 5-lipoxygenase | AI044102 |
| 1590 | UI-R-C1-kb-f-02-0-UI | Pro-opiomelanocortin | CK840699 |
| 1591 | UI-R-C1-ll-c-01-0-UI | Cell division cycle 5-like | CK845244 |
| 1592 | UI-R-C2-mr-c-08-0-UI | RAB3A interacting protein | BF559466 |
| 1593 | UI-R-C2-na-d-08-0-UI | Acidic (leucine-rich) nuclear phosphoprotein 32 family, member A | AI070967 |
| 1594 | UI-R-C2-nh-g-06-0-UI | Protein tyrosine phosphatase, receptor type, J | AI071864 |
| 1595 | UI-R-DK0-cfw-c-12-0-UI | Potassium voltage-gated channel subfamily KQT member 5 (Voltage-gated potassium channel subunit Kv7.5) (Potassium channel alpha subunit KvLQT5) (KQT-like 5) | BI290441 |
| 1596 | UI-R-DK0-cga-f-12-0-UI | RIKEN cDNA 2610528J11 | BI290814 |
| 1597 | UI-R-DK0-cgj-g-08-0-UI | Transcribed locus | BI296977 |
| 1598 | UI-R-CV2-cgt-d-08-0-UI | mitogen-activated protein kinase kinase kinase kinase 5 isoform 2 | BI296624 |
| 1599 | UI-R-DQ0-cja-i-08-0-UI | Core binding factor beta | BI303479 |
| 1600 | UI-R-E0-dl-c-11-0-UI | Demethyl-Q 7 | BF558335 |
| 1601 | UI-R-A1-dr-c-09-0-UI | Proliferating cell nuclear antigen | AA924358 |
| 1602 | UI-R-A1-du-e-07-0-UI | RT1 class Ib, locus Aw2 | AA924062 |
| 1603 | UI-R-E1-gh-f-04-0-UI | Insulin-like growth factor 1 | AA963258 |
| 1604 | UI-R-E1-go-h-02-0-UI | CEA-related cell adhesion molecule 10 | BF556893 |
| 1605 | UI-R-DB0-byz-f-02-0-UI | Secretoglobin, family 2A, member 2 | BI285396 |
| 1606 | UI-R-DD0-bzu-a-11-0-UI | Cysteine-rich secretory protein 1 | BI281439 |
| 1607 | UI-R-CW0s-cce-b-12-0-UI | Ribophorin I | BI282703 |
| 1608 | UI-R-CX0s-cco-c-01-0-UI | Ribosomal protein L4 | BI284357 |
| 1609 | UI-R-CX0s-cct-d-03-0-UI | Intercellular adhesion molecule 1 | BI284881 |
| 1610 | UI-R-DK0-cdc-b-03-0-UI | Stromal antigen 1 | BI288112 |
| 1611 | UI-R-DK0-cen-c-06-0-UI | RAD50 homolog | BI294652 |
| 1612 | UI-R-DK0-cez-a-03-0-UI | Jumonji domain containing 2A | BI296316 |
| 1613 | UI-R-DK0-cfe-b-11-0-UI | Adiponectin receptor 1 | BI289063 |
| 1614 | UI-R-CM0-bjl-b-12-0-UI | Actin, alpha 1, skeletal muscle | BF395208 |
| 1615 | UI-R-CA0-bma-g-08-0-UI | Potassium large conductance calcium-activated channel, subfamily M, beta member 4 | BF410654 |
| 1616 | UI-R-BT1-bmv-e-09-0-UI | Cyclin-dependent kinase-like 3 | BF411681 |
| 1617 | UI-R-BT1-bnq-h-10-0-UI | CBFA2T1 identified gene homolog | BF412687 |
| 1618 | UI-R-CA0-boj-a-12-0-UI | Zinc finger, FYVE domain containing 20 | BF413827 |
| 1619 | UI-R-BJ2-bqi-g-05-0-UI | Retinoblastoma 1 | BF418684 |
| 1620 | UI-R-BJ2-bqp-a-02-0-UI | WD repeat domain 5 | BF419213 |
| 1621 | UI-R-CW0-bvv-h-11-0-UI | Lysophospholipase | BG371487 |
| 1622 | UI-R-CW0-bwg-g-06-0-UI | X83328 protein | BI274253 |
| 1623 | UI-R-CZ0-byf-h-07-0-UI | RIKEN cDNA 2410022L05 | BI277736 |
| 1624 | UI-R-DA0-byn-e-04-0-UI | Parotid secretory protein | BI279361 |
| 1625 | UI-R-CA0-bfx-a-12-0-UI | Protein phosphatase 2 (formerly 2A), regulatory subunit A (PR 65), alpha isoform | BF394544 |
| 1626 | UI-R-CA0-bgj-e-09-0-UI | SH2 domain binding protein 1 (tetratricopeptide repeat containing) | BF401181 |
| 1627 | UI-R-CA0-biy-f-12-0-UI | Transcribed locus | BF403075 |
| 1628 | UI-R-CA1-bjd-e-12-0-UI | HLA-B associated transcript-2 isoform a | BF403999 |
| 1629 | UI-R-CA1-bjf-j-09-0-UI | Disabled homolog 1 | BF406318 |
| 1630 | UI-R-CV1-brv-h-06-0-UI | RIKEN cDNA E030034P13 | BG372587 |
| 1631 | UI-R-CT0-bud-f-10-0-UI | Actin related protein 2/3 complex, subunit 5-like | BG381641 |
| 1632 | UI-R-CT0-buo-g-11-0-UI | Hippocalcin | BG376381 |
| 1633 | UI-R-CU0-buv-f-11-0-UI | Heterogeneous nuclear ribonucleoprotein M | BG376766 |
| 1634 | UI-R-CU0-bvh-c-04-0-UI | Cell division cycle 20 homolog | BG377963 |
| 1635 | UI-R-CA0-axd-c-02-0-UI | Checkpoint suppressor 1 | BE112826 |
| 1636 | UI-R-CA0-axj-h-06-0-UI | WD repeat domain 44 | BE110086 |
| 1637 | UI-R-BS1-axx-f-10-0-UI | NP_066564.1 general control of amino-acid synthesis 5-like 2 | BE115942 |
| 1638 | UI-R-BS1-ayo-b-08-0-UI | XP_001092853.1 ubiquitin A-52 residue ribosomal protein fusion product 1 | BE117466 |
| 1639 | UI-R-CA0-baq-e-01-0-UI | Calsenilin, presenilin binding protein, EF hand transcription factor | BE120356 |
| 1640 | UI-R-CA0-baz-d-02-0-UI | Hepatocyte growth factor activator | BE119649 |
| 1641 | UI-R-CA1-bbp-f-05-0-UI | Dystrophin, muscular dystrophy | BF387048 |
| 1642 | UI-R-CA1-bcc-d-03-0-UI | Dishevelled 3, dsh homolog | BF390548 |
| 1643 | UI-R-BS2-bek-h-08-0-UI | ataxin 2-binding protein 1 isoform 2 | BF397315 |
| 1644 | UI-R-BS2-bez-d-10-0-UI | RIKEN cDNA 1300007B12; clone MNCb-2755 | BF397521 |
| 1645 | UI-R-BU0-ang-a-08-0-UI | Low density lipoprotein receptor-related protein 6 | BF562488 |
| 1646 | UI-R-BU0-aoz-a-12-0-UI | FERM, RhoGEF and pleckstrin domain protein 2 | BE095759 |
| 1647 | UI-R-BX0-arm-e-04-0-UI | FKSG26 protein | BE104242 |
| 1648 | UI-R-BO1-asj-b-05-0-UI | Moesin | BE105866 |
| 1649 | UI-R-BO1-asq-e-07-0-UI | Wingless-related MMTV integration site 7A | BE106651 |
| 1650 | UI-R-BJ1-ata-h-04-0-UI | RIKEN cDNA 3110040N11 | CK842671 |
| 1651 | UI-R-BJ1-auo-e-08-0-UI | Sortilin 1 | BE110483 |
| 1652 | UI-R-BJ1-avb-a-09-0-UI | F-box only protein 21 | CK844022 |
| 1653 | UI-R-BJ1-avm-g-09-0-UI | Nudix (nucleoside diphosphate linked moiety X)-type motif 9 | BE112448 |
| 1654 | UI-R-BJ1-avt-f-05-0-UI | Fatty acid desaturase 1 | BE110579 |
| 1655 | UI-R-BO0-ahy-a-03-0-UI | Camello-like 3 | BF568013 |
| 1656 | UI-R-BO0-aie-c-03-0-UI | Synaptosomal-associated protein 25 | BF562850 |
| 1657 | UI-R-BJ0p-aik-g-06-0-UI | PTPRF interacting protein, binding protein 1 (liprin beta 1) | AW523994 |
| 1658 | UI-R-BJ0p-air-f-10-0-UI | BPY2 interacting protein 1 | CK842010 |
| 1659 | UI-R-BO1-ajv-c-12-0-UI | Suppression of tumorigenicity 7-like | AW527529 |
| 1660 | UI-R-BT1-akc-d-05-0-UI | LOC363015 | AW532346 |
| 1661 | UI-R-BT1-akj-f-03-0-UI | HIRA interacting protein 3 | AW528874 |
| 1662 | UI-R-BT1-akq-d-11-0-UI | Cyclin-dependent kinase 9 (CDC2-related kinase) | AW530704 |
| 1663 | UI-R-BU0-ams-e-07-0-UI | Sodium channel, voltage-gated, type 10, alpha polypeptide | BF564427 |
| 1664 | UI-R-BU0-ana-b-04-0-UI | Hypothetical LOC310781 | BF564898 |
| 1665 | UI-R-AC0-yp-f-09-0-UI | 5'-nucleotidase, cytosolic III-like | BF525253 |
| 1666 | UI-R-AB1-yy-d-02-0-UI | Stress-induced phosphoprotein 1 | CK840907 |
| 1667 | UI-R-AF1-aau-h-08-0-UI | Bone morphogenic protein receptor, type II (serine/threonine kinase) | BF543176 |
| 1668 | UI-R-Y0-abb-g-07-0-UI | Signal transducer and activator of transcription 5A | BF522093 |
| 1669 | UI-R-Y0-abp-b-08-0-UI | Transcribed locus |  |
| 1670 | UI-R-Y0-acb-b-10-0-UI | Phosphodiesterase 4D interacting protein (myomegalin) | BF521977 |
| 1671 | UI-R-BJ0p-aex-f-01-0-UI | Cytoplasmic linker 2 | CK841158 |
| 1672 | UI-R-BJ0p-afg-d-10-0-UI | Lipoic acid synthetase | CK841419 |
| 1673 | UI-R-BJ0p-afn-c-03-0-UI | Docking protein 1 | CK841544 |
| 1674 | UI-R-BJ0p-afw-h-06-0-UI | TM2 domain containing 3 | CK841265 |
| 1675 | UI-R-C2p-rj-c-02-0-UI | Pogo transposable element with ZNF domain | AI556679 |
| 1676 | UI-R-C2p-rq-g-10-0-UI | RIKEN cDNA 1810022C23 | AI500837 |
| 1677 | UI-R-C2p-sb-c-03-0-UI | Forty-two-three domain containing 1 | AI501447 |
| 1678 | UI-R-C3-si-a-09-0-UI | Cyclin dependent kinase 2 | BF542448 |
| 1679 | UI-R-C3-tz-e-10-0-UI | Poliovirus receptor | BF522416 |
| 1680 | UI-R-G0-uj-g-02-0-UI | Myosin, heavy polypeptide 4 | AI577921 |
| 1681 | UI-R-G0-uu-h-10-0-UI | Microsomal glutathione S-transferase 3 | AI705105 |
| 1682 | UI-R-Y0-vc-g-10-0-UI | Transcribed locus | AI575287 |
| 1683 | UI-R-AC1-xs-c-06-0-UI | Bardet-Biedl syndrome 1 homolog | BF524806 |
| 1684 | UI-R-AC0-yh-h-10-0-UI | Eukaryotic translation initiation factor 3, subunit 6 interacting protein | BF525098 |
| 1685 | UI-R-E1-fs-c-05-0-UI | NP_083376.2 N, 1 | AA957097 |
| 1686 | UI-R-E1-ge-h-08-0-UI | Neurexophilin 4 | AA957158 |
| 1687 | UI-R-C0-jc-b-02-0-UI | Excision repair cross-complementing rodent repair deficiency, complementation group 6 | AI030476 |
| 1688 | UI-R-C1-ju-d-11-0-UI | Prr6 protein | BF554152 |
| 1689 | UI-R-C1-kq-a-08-0-UI | Syntaxin 3 | AI502922 |
| 1690 | UI-R-C1-li-h-05-0-UI | Zinc finger, MYM-type 3 | BF546722 |
| 1691 | UI-R-C2p-oh-d-02-0-UI | XP_001104609.1 glucosidase, alpha; neutral C | AI172595 |
| 1692 | UI-R-BT0-pl-g-11-0-UI | Schlafen 3 | AI144671 |
| 1693 | UI-R-BT0-ps-c-02-0-UI | RIKEN cDNA 2510049I19 | AI145607 |
| 1694 | UI-R-BT0-pz-h-05-0-UI | putative protein, with at least 6 transmembrane domains, of ancient origin | AI145867 |
| 1695 | UI-R-A0-ag-h-10-0-UI | Thioredoxin 1 | BF549222 |
| 1696 | UI-R-A0-ax-g-04-0-UI | Breast cancer metastasis-suppressor 1-like | BF549715 |
| 1697 | UI-R-A0-bl-f-10-0-UI | CD52 antigen | BF547876 |
| 1698 | UI-R-E0-br-g-05-0-UI | Mitochondrial ribosomal protein L42 | AA866469 |
| 1699 | UI-R-E0-cw-c-07-0-UI | SET domain-containing protein | AI502201 |
| 1700 | UI-R-E0-dd-f-03-0-UI | Guanine nucleotide binding protein, beta 1 | BF544114 |
| 1701 | UI-R-E0-dk-e-09-0-UI | Optic atrophy 1 homolog | BF558324 |
| 1702 | UI-R-A1-dp-g-04-0-UI | ezrin-binding partner PACE-1 | BF558651 |
| 1703 | UI-R-A1-ep-d-05-0-UI | Transformation related protein 53 binding protein 1 | CK840114 |
| 1704 | UI-R-E1-fb-g-03-0-UI | Eukaryotic translation initiation factor 5A2 | BF559352 |
| 1705 | UI-R-CA0-bhp-e-04-0-UI | Myocardial ischemic preconditioning upregulated 1 | BF402458 |
| 1706 | UI-R-CX0-bws-d-07-0-UI | ATP/GTP-binding protein | BI274651 |
| 1707 | UI-R-CV2-chv-a-03-0-UI | McKusick-Kaufman syndrome protein | BI298816 |
| 1708 | UI-R-E1-gb-b-05-0-UI | Transcribed locus | AA957596 |
| 1709 | UI-R-E1-go-b-03-0-UI | XP_001093409.1 cerebral cavernous malformation 2 isoform 2 | AA964553 |
| 1710 | UI-R-C0-ib-d-09-0-UI | ring finger protein 170 | AA998224 |
| 1711 | UI-R-C1-jt-c-01-0-UI | XP_001113514.1 zinc finger protein, subfamily 1A, 4 isoform 1 | AI044781 |
| 1712 | UI-R-C2p-qw-c-12-0-UI | Serine (or cysteine) peptidase inhibitor, clade C (antithrombin), member 1 | AI555505 |
| 1713 | UI-R-C2p-rm-b-06-0-UI | paired-Ig-like receptor B | BF547395 |
| 1714 | UI-R-C0-ip-h-12-0-UI | Ras association (RalGDS/AF-6) domain family 6 | AI029149 |
| 1715 | UI-R-C0-iw-f-11-0-UI | Gene model 784 | BF551614 |
| 1716 | UI-R-C1-kl-d-07-0-UI | p150 target of rapamycin (TOR)-scaffold protein containing WD-repeats | AI058297 |
| 1717 | UI-R-C1-ko-b-03-0-UI | Myeloid/lymphoid or mixed-lineage leukemia 5 | AI071279 |
| 1718 | UI-R-C1-lg-h-04-0-UI | Chaperonin subunit 8 (theta) | AI059499 |
| 1719 | UI-R-C1-lm-a-05-0-UI | Dachsous 1 | AI060199 |
| 1720 | UI-R-E1-fw-e-10-0-UI | Proteasome (prosome, macropain) 26S subunit, non-ATPase, 12 | BF561939 |
| 1721 | UI-R-C0-ih-g-05-0-UI | WD repeat domain 79 | BF554024 |
| 1722 | UI-R-C1-jz-d-03-0-UI | Transcribed locus | AI045537 |
| 1723 | UI-R-C1-lq-c-02-0-UI | hypothetical protein from BCRA2 region | AI058990 |
| 1724 | UI-R-C0-hh-b-01-0-UI | Transcribed locus | AA996869 |
| 1725 | UI-R-C0-ir-e-10-0-UI | Asparaginase like 1 | AI029368 |
| 1726 | UI-R-C1-js-c-04-0-UI | Transcribed locus | AI044042 |
| 1727 | UI-R-C1-lg-f-07-0-UI | Origin recognition complex, subunit 1-like | AI059490 |
| 1728 | UI-R-A1-eu-f-09-0-UI | Trk-fused gene | BF556943 |
| 1729 | UI-R-E1-fc-h-12-0-UI | TRAF-interacting protein | AA955684 |
| 1730 | UI-R-E1-fv-e-04-0-UI | WD repeat domain 11 protein | AA957762 |
| 1731 | UI-R-E1-fy-f-10-0-UI | Hect (homologous to the E6-AP (UBE3A) carboxyl terminus) domain and RCC1 (CHC1)-like domain (RLD) 1 | BF557160 |
| 1732 | UI-R-C0-hl-d-10-0-UI | WD repeat domain 3 | AA997374 |
| 1733 | UI-R-C0-il-h-02-0-UI | Ariadne ubiquitin-conjugating enzyme E2 binding protein homolog 1 | AI028926 |
| 1734 | UI-R-E0-di-h-04-0-UI | Mevalonate kinase | AA900678 |
| 1735 | UI-R-A1-ew-b-07-0-UI | Glycerol-3-phosphate acyltransferase, mitochondrial | AI146190 |
| 1736 | UI-R-C0-in-f-12-0-UI | Solute carrier family 6 (neurotransmitter transporter, creatine), member 8 | BF545211 |
| 1737 | UI-R-C0-iv-f-02-0-UI | Reticulon 1 | BF551597 |
| 1738 | UI-R-C0-ir-e-11-0-UI | Ribosomal protein S15 | AI029369 |
| 1739 | UI-R-BT0-qb-b-06-0-UI | Phosphatidylinositol 4-kinase, catalytic, alpha polypeptide | AI146154 |
| 1740 | UI-R-C1-ko-g-07-0-UI | Complement component 5 | BF551806 |
| 1741 | UI-R-C2-ms-b-09-0-UI | Integrin linked kinase | AI071097 |
| 1742 | UI-R-E0-bq-g-08-0-UI | CTD-binding SR-like rA1 | BF547976 |
| 1743 | UI-R-E0-cw-h-10-0-UI | Hydroxysteroid dehydrogenase-1, delta<5>-3-beta | BF555326 |
| 1744 | UI-R-C2-nf-a-07-0-UI | Serine/arginine-rich protein specific kinase 3 | AI072058 |
| 1745 | UI-R-C2-ng-h-10-0-UI | Acyl-CoA thioesterase 8 | BF549660 |
| 1746 | UI-R-C2-nj-g-03-0-UI | ATPase, H+ transporting, V1 subunit A, isoform 1 | AI071788 |
| 1747 | UI-R-C2-nl-g-05-0-UI | Transcribed locus | AI071975 |
| 1748 | UI-R-A0-ag-h-02-0-UI | Trafficking protein particle complex 3 | BF542237 |
| 1749 | UI-R-A0-aq-c-10-0-UI | Synuclein, alpha | AA819897 |
| 1750 | UI-R-A0-az-e-09-0-UI | Beta galactoside alpha 2,6 sialyltransferase 1 | AA818636 |
| 1751 | UI-R-A0-bh-f-04-0-UI | CD53 antigen | BF548059 |
| 1752 | UI-R-E0-cp-b-02-0-UI | Peptidylprolyl isomerase F (cyclophilin F) | BF555112 |
| 1753 | UI-R-E0-cz-b-03-0-UI | Deiodinase, iodothyronine, type III | BF555595 |
| 1754 | UI-R-C0-jg-a-04-0-UI | AT rich interactive domain 4B (Rbp1 like) | AI045666 |
| 1755 | UI-R-C0-jj-b-02-0-UI | RIKEN cDNA 1700034P14 | BF550726 |
| 1756 | UI-R-C1-kd-d-02-0-UI | RIKEN cDNA 2810002N01 | AI044693 |
| 1757 | UI-R-C1-kf-e-04-0-UI | GTPase, IMAP family member 5 | AI045041 |
| 1758 | UI-R-C1-ki-f-03-0-UI | Rho GTPase activating protein 20 | BF560734 |
| 1759 | UI-R-C1-kk-d-10-0-UI | Butyrate-induced transcript 1 | BF546125 |
| 1760 | UI-R-C1-lb-d-10-0-UI | Corticotropin releasing hormone receptor 1 | BF547183 |
| 1761 | UI-R-C1-le-g-08-0-UI | XP_001105117.1 splicing factor, arginine/serine-rich 8 isoform 1 isoform 4 | AI059650 |
| 1762 | UI-R-C1-lo-c-04-0-UI | N-glycanase 1 | AI071925 |
| 1763 | UI-R-C1-lq-a-10-0-UI | MGC52019 protein | AI058978 |
| 1764 | UI-R-A0-bf-e-12-0-UI | Flotillin 2 | AA819529 |
| 1765 | UI-R-E1-fa-e-07-0-UI | retinoid x receptor interacting protein | AA955520 |
| 1766 | UI-R-E1-fg-f-11-0-UI | Transducin (beta)-like 1 X-linked | AA955760 |
| 1767 | UI-R-E1-fl-b-08-0-UI | Cgi67 serine protease precursor | AA956933 |
| 1768 | UI-R-C0-gx-e-11-0-UI | Flightless I homolog | BF557843 |
| 1769 | UI-R-C0-hd-h-09-0-UI | Zinc finger protein 91 | AA996615 |
| 1770 | UI-R-C0-hi-d-05-0-UI | Popeye domain containing 2 | BF560788 |
| 1771 | UI-R-C0-hu-d-07-0-UI | Stromal cell derived factor 4 | AA997861 |
| 1772 | UI-R-C0-it-f-03-0-UI | XP_001101765.1 haloacid dehalogenase-like hydrolase domain containing 3 isoform 1 | AI030259 |
| 1773 | UI-R-C0-iy-g-11-0-UI | Checkpoint with forkhead and ring finger domains | AI029628 |
| 1774 | UI-R-C0-hu-c-07-0-UI | Protein phosphatase 1, regulatory (inhibitor) subunit 1A | AA997855 |
| 1775 | UI-R-C0-iz-c-05-0-UI | Adenylate cyclase 5 | BF552755 |
| 1776 | UI-R-C1-kg-h-03-0-UI | Inositol 1,4,5-trisphosphate 3-kinase A | AI045139 |
| 1777 | UI-R-C1-ko-d-11-0-UI | Coagulation factor II | AI071305 |
| 1778 | UI-R-C1-kw-h-01-0-UI | Heparin-binding EGF-like growth factor | AI059540 |
| 1779 | UI-R-C1-lf-c-03-0-UI | Enoyl Coenzyme A hydratase, short chain, 1, mitochondrial | BF549537 |
| 1780 | UI-R-C2p-nv-f-06-0-UI | Gamma crystallin F (Gamma crystallin 4-1) | BF553419 |
| 1781 | UI-R-C2p-og-a-10-0-UI | Rho-associated coiled-coil forming kinase 1 | AI137331 |
| 1782 | UI-R-BT0-pn-h-04-0-UI | A disintegrin and metalloproteinase domain 17 (tumor necrosis factor, alpha, converting enzyme) | AI144608 |
| 1783 | UI-R-A0-ae-e-08-0-UI | Regulator of chromosome condensation 2 | BF548172 |
| 1784 | UI-R-CV2-cht-b-03-0-UI | Transcribed locus | BI299261 |
| 1785 | UI-R-DL0-cip-l-14-0-UI | Brain protein 44-like | BI302222 |
| 1786 | UI-R-DN0-ciu-k-19-0-UI | XP_001089737.1 F35A5.1 isoform 1 | BI291914 |
| 1787 | UI-R-DO0-ciw-k-21-0-UI | Caveolin 2 | BI292516 |
| 1788 | UI-R-A1-dz-f-02-0-UI | Argininosuccinate synthetase | BF559599 |
| 1789 | UI-R-A1-ec-g-01-0-UI | Transcription factor 2 | AA925724 |
| 1790 | UI-R-A1-eg-c-07-0-UI | Mitogen-activated protein kinase 12 | BF556108 |
| 1791 | UI-R-A1-el-a-01-0-UI | Clathrin, light polypeptide (Lca) | AA926025 |
| 1792 | UI-R-C0-hi-b-07-0-UI | Solute carrier family 32 (GABA vesicular transporter), member 1 | AA996551 |
| 1793 | UI-R-C0-hp-b-12-0-UI | Nucleophosmin 1 | AA998895 |
| 1794 | UI-R-CU0s-cbs-b-01-0-UI | Cytochrome P450, family 2, subfamily e, polypeptide 1 | BI281751 |
| 1795 | UI-R-CU0s-cby-f-06-0-UI | Hexosaminidase A | BI282274 |
| 1796 | UI-R-DK0-cdh-h-10-0-UI | Reproductive homeobox on X chromosome, 9 | BI289049 |
| 1797 | UI-R-DK0-cdu-a-03-0-UI | BTB (POZ) domain containing 10 | BI293641 |
| 1798 | UI-R-DK0-cec-e-04-0-UI | CD320 antigen | BI294818 |
| 1799 | UI-R-DK0-cef-c-10-0-UI | Solute carrier family 12, member 4 | BI295055 |
| 1800 | UI-R-CA0-blz-b-02-0-UI | Protein tyrosine phosphatase, non-receptor type 4 | BF418071 |
| 1801 | UI-R-BJ2-bra-c-03-0-UI | Katanin p60 subunit A-like 1 | CK843397 |
| 1802 | UI-R-BJ2-bre-h-04-0-UI | Laminin alpha-4 chain precursor | BF408530 |
| 1803 | UI-R-DK0-cfo-c-05-0-UI | Mesoderm development candidate 1 | BI289792 |
| 1804 | UI-R-BJ2-bos-b-01-0-UI | Methyltransferase-like 3 | BF414795 |
| 1805 | UI-R-BJ2-bph-e-02-0-UI | ST3 beta-galactoside alpha-2,3-sialyltransferase 6 | CK844574 |
| 1806 | UI-R-BJ2-bpp-b-04-0-UI | RNA polymerase III subunit RPC2 | CK843129 |
| 1807 | UI-R-BJ2-bpx-b-05-0-UI | CDNA clone IMAGE:7302535, with apparent retained intron | CK843595 |
| 1808 | UI-R-CW0-bwp-e-12-0-UI | RAB21, member RAS oncogene family | BI276387 |
| 1809 | UI-R-CX0-bxd-c-04-0-UI | hypothetical protein | BI274722 |
| 1810 | UI-R-CX0-bxk-f-04-0-UI | Solute carrier family 30 (zinc transporter), member 2 | BI277879 |
| 1811 | UI-R-CY0-bxs-e-02-0-UI | Myosin, heavy polypeptide 2, skeletal muscle, adult | BI277545 |
| 1812 | UI-R-DE0-cag-d-01-0-UI | Ribosomal protein S4, X-linked | BI280302 |
| 1813 | UI-R-CT0s-cav-d-10-0-UI | Cortistatin | BI281700 |
| 1814 | UI-R-CA1-bhz-g-01-0-UI | Serine (or cysteine) peptidase inhibitor, clade B (ovalbumin), member 10 | BF399855 |
| 1815 | UI-R-CA1-bip-h-02-0-UI | hypothetical protein FLJ23033 | BF405115 |
| 1816 | UI-R-CS0-bsd-e-08-0-UI | RIKEN cDNA 2210016L21 gene | BG373502 |
| 1817 | UI-R-CV1-bsp-g-04-0-UI | Retinal pigment epithelium 65 | BG374468 |
| 1818 | UI-R-CS0-btj-g-02-0-UI | RIKEN cDNA 2700002I20 | BG379747 |
| 1819 | UI-R-CT0-btw-f-07-0-UI | Latexin | BG380392 |
| 1820 | UI-R-CV1-bvq-b-07-0-UI | Glutamate-ammonia ligase (glutamine synthetase) domain containing 1 | BG378276 |
| 1821 | UI-R-CA0-bkj-c-06-0-UI | NP_037146.1 | BF416337 |
| 1822 | UI-R-CN0-bkx-c-04-0-UI | chemokine (C-X-C motif) ligand 16 | BF417122 |
| 1823 | UI-R-CN0-blg-f-01-0-UI | Integrin-linked kinase-associated serine/threonine phosphatase 2C | BF417477 |
| 1824 | UI-R-BS1-ayx-a-07-0-UI | Tubulin, beta 3 | BE108242 |
| 1825 | UI-R-BS1-aze-b-12-0-UI | Purinergic receptor P2Y, G-protein coupled 1 | BE117641 |
| 1826 | UI-R-BS1-azr-d-01-0-UI | Zinc finger protein 386 (Kruppel-like) | BE118665 |
| 1827 | UI-R-CA0-bai-h-08-0-UI | RIKEN cDNA 2700059L22 | BE119777 |
| 1828 | UI-R-CA1-bco-g-12-0-UI | Tensin | BF390735 |
| 1829 | UI-R-BS2-bdd-e-02-0-UI | Vacuolar protein sorting 4a | BF388609 |
| 1830 | UI-R-BS2-bdo-d-09-0-UI | Transcribed locus | BF389759 |
| 1831 | UI-R-BS2-bdz-b-11-0-UI | WD repeat domain 6 | BF396530 |
| 1832 | UI-R-CA0-bgw-d-10-0-UI | NP_001847.3 1 type XVI collagen precursor | BF393905 |
| 1833 | UI-R-CA0-bhg-h-01-0-UI | RIKEN cDNA 1200016B17 | BF400873 |
| 1834 | UI-R-BT1-aqh-d-05-0-UI | Ankyrin repeat domain 5 | BE101788 |
| 1835 | UI-R-BT1-aqu-a-05-0-UI | Solute carrier family 14 (urea transporter), member 1 | BE102916 |
| 1836 | UI-R-BJ1-atl-a-01-0-UI | RAB10, member RAS oncogene family | CK844926 |
| 1837 | UI-R-BJ1-atq-c-04-0-UI | MIF4G domain containing | CK842974 |
| 1838 | UI-R-BJ1-atw-e-01-0-UI | Deltex 2 homolog | CK839339 |
| 1839 | UI-R-BJ1-auf-b-03-0-UI | Zinc finger protein 644 | CK842108 |
| 1840 | UI-R-BJ1-avy-a-03-0-UI | Topoisomerase (DNA) I | BE115617 |
| 1841 | UI-R-BJ1-awc-e-10-0-UI | Tp53rk binding protein | CK844356 |
| 1842 | UI-R-BJ1-awk-b-11-0-UI | CD5 antigen | CK839968 |
| 1843 | UI-R-CA0-awv-d-02-0-UI | Matrix extracellular phosphoglycoprotein with ASARM motif | BE107579 |
| 1844 | UI-R-BO1-aiy-b-12-0-UI | Transcribed locus | BF563065 |
| 1845 | UI-R-BO1-ajg-b-01-0-UI | Echinoderm microtubule associated protein like 5 | BF563182 |
| 1846 | UI-R-BO1-ajj-a-07-0-UI | Limb expression 1 homolog (chicken) | BF565235 |
| 1847 | UI-R-BO1-ajp-e-05-0-UI | Transcribed locus | AW529141 |
| 1848 | UI-R-C4-aky-g-11-0-UI | Oxysterol binding protein 2 | AW531685 |
| 1849 | UI-R-C4-alh-a-11-0-UI | Solute carrier family 4, sodium bicarbonate cotransporter, member 9 | BF563564 |
| 1850 | UI-R-C4-alo-b-11-0-UI | Immunoglobulin G heavy chain | AW534335 |
| 1851 | UI-R-C4-alx-a-11-0-UI | Transcribed locus | AW535510 |
| 1852 | UI-R-BU0-apg-f-04-0-UI | Small glutamine-rich tetratricopeptide repeat (TPR)-containing, beta | BE096481 |
| 1853 | UI-R-BO1-apv-b-11-0-UI | Membrane protein, palmitoylated 6 (MAGUK p55 subfamily member 6) | BE097259 |
| 1854 | UI-R-AA1-zy-c-01-0-UI | Pleckstrin homology domain containing, family B (evectins) member 2 | BF543969 |
| 1855 | UI-R-AG1-aam-b-01-0-UI | Importin 4 | AI710603 |
| 1856 | UI-R-Y0-aci-a-02-0-UI | Complement component factor H | AI716854 |
| 1857 | UI-R-Y0-acu-d-03-0-UI | One cut domain, family member 1 | AI717049 |
| 1858 | UI-R-BJ0-adc-c-05-0-UI | Fibrillin 1 | CK839216 |
| 1859 | UI-R-BJ0-aec-f-08-0-UI | Motile sperm domain containing 3 | AW253898 |
| 1860 | UI-R-BJ0p-age-a-12-0-UI | Retinoid X receptor beta | AW520838 |
| 1861 | UI-R-BO0-agl-e-01-0-UI | Synaptotagmin VII | AW521348 |
| 1862 | UI-R-BO0-agy-d-08-0-UI | Nuclear factor I/X | BF567335 |
| 1863 | UI-R-BO0-aho-c-01-0-UI | Zyg-11 homolog B -like | BF567873 |
| 1864 | UI-R-C3-sq-g-10-0-UI | Pleckstrin homology domain containing, family F (with FYVE domain) member 2 | BF523177 |
| 1865 | UI-R-C3-sz-d-07-0-UI | XP_001084389.1 family with sequence similarity 57, member A isoform 2 | BF543482 |
| 1866 | UI-R-C3-tk-d-01-0-UI | Zinc metallopeptidase, STE24 homolog | AI578082 |
| 1867 | UI-R-C3-ts-c-03-0-UI | Quaking homolog, KH domain RNA binding | AI549152 |
| 1868 | UI-R-AB0-vt-b-06-0-UI | Exosome component 3 | AI601932 |
| 1869 | UI-R-AD0-wg-a-07-0-UI | Acid phosphatase 6, lysophosphatidic | CK842327 |
| 1870 | UI-R-AG0-wt-g-09-0-UI | NADH dehydrogenase (ubiquinone) 1 beta subcomplex, 9 | CK840457 |
| 1871 | UI-R-AG0-xc-e-05-0-UI | Calmodulin 3 | CK845157 |
| 1872 | UI-R-AE1-zj-c-07-0-UI | Proteasome (prosome, macropain) 26S subunit, non-ATPase, 9 | AI710949 |
| 1873 | UI-R-AD1-zp-d-05-0-UI | Sterol regulatory element binding factor 1 | BF524909 |
| 1874 | UI-R-C0-hr-f-06-0-UI | Ethanolamine kinase 2 | BF561410 |
| 1875 | UI-R-C0-id-g-07-0-UI | chromosome 6 open reading frame 188 | AA998169 |
| 1876 | UI-R-Y0-lz-d-11-0-UI | Glycoprotein hormone alpha 2 | AI111460 |
| 1877 | UI-R-Y0-mo-g-09-0-UI | Procollagen, type IV, alpha 4 | AI111986 |
| 1878 | UI-R-C2-nc-h-01-0-UI | Transcribed locus |  |
| 1879 | UI-R-C2p-nw-e-11-0-UI | AF4/FMR2 family, member 4 | BF553607 |
| 1880 | UI-R-BT0-qh-g-04-0-UI | Phosphoribosyl transferase domain containing 1 | AI146137 |
| 1881 | UI-R-C2p-qn-e-05-0-UI | mKIAA0376 protein | BF547282 |
| 1882 | UI-R-C2p-qt-f-02-0-UI | UDP-glucose ceramide glucosyltransferase | AI555169 |
| 1883 | UI-R-C2p-rc-f-11-0-UI | Synaptic vesicle glycoprotein 2a | AI555976 |
| 1884 | UI-R-E0-bv-c-10-0-UI | Proprotein convertase subtilisin/kexin type 5 | AA859578 |
| 1885 | UI-R-E0-ca-d-01-0-UI | Protein kinase C and casein kinase substrate in neurons 2 | BF550004 |
| 1886 | UI-R-E0-ch-f-06-0-UI | Cell division cycle 26 | AA866438 |
| 1887 | UI-R-E0-cn-g-01-0-UI | Septin 9 | AA875313 |
| 1888 | UI-R-A1-dv-c-11-0-UI | hypothetical protein FLJ20309 | BF555714 |
| 1889 | UI-R-A1-dy-h-02-0-UI | hypothetical protein | BF556002 |
| 1890 | UI-R-A1-ee-h-06-0-UI | Leucine rich repeat containing 42 | AA925289 |
| 1891 | UI-R-A1-ej-b-09-0-UI | HECT, C2 and WW domain containing E3 ubiquitin protein ligase 2 isoform 1 | BF544098 |
| 1892 | UI-R-E1-gq-h-09-0-UI | Transcribed locus | AA964802 |
| 1893 | UI-R-C0-hl-f-01-0-UI | Mitogen activated protein kinase kinase kinase kinase 1 | AA997459 |
| 1894 | UI-R-C2p-oe-d-10-0-UI | Transmembrane emp24 protein transport domain containing 5 | AI137113 |
| 1895 | UI-R-AF1-aav-a-01-0-UI | Interferon-induced protein 35 | BF543178 |
| 1896 | UI-R-C4-akw-c-03-0-UI | Solute carrier family 17 (anion/sugar transporter), member 5 | BF563945 |
| 1897 | UI-R-CA1-bci-d-03-0-UI | Spermatogenesis associated 21 | BF390864 |
| 1898 | UI-R-E0-cb-a-11-0-UI | Leucine-rich repeat-containing 8 | AA875002 |
| 1899 | UI-R-E0-dh-a-06-0-UI | XP_001118943.1 zinc finger protein 8, partial | BF558039 |
| 1900 | UI-R-A1-dy-a-08-0-UI | Carbohydrate sulfotransferase D4ST1 (Dermatan 4-sulfotransferase 1) (D4ST-1) | BF555974 |
| 1901 | UI-R-E1-fd-a-02-0-UI | Transcribed locus | AA955889 |
| 1902 | UI-R-C2-mu-d-06-0-UI | Pdz-containing protein | AI072232 |
| 1903 | UI-R-C2p-oe-e-08-0-UI | Regulatory factor X, 3 (influences HLA class II expression) | AI137120 |
| 1904 | UI-R-C0-hc-d-10-0-UI | Zinc finger protein 105 | BF557743 |
| 1905 | UI-R-C0-hk-a-11-0-UI | ArsA arsenite transporter, ATP-binding, homolog 1 (bacterial) | AA997032 |
| 1906 | UI-R-C0-je-h-06-0-UI | Interferon gamma receptor 2 | AI030843 |
| 1907 | UI-R-C0-jo-c-04-0-UI | double homeobox protein | AI030773 |
| 1908 | UI-R-C1-jt-d-02-0-UI | N-acetylglucosamine kinase | AI044789 |
| 1909 | UI-R-C1-jz-f-08-0-UI | RAD1 homolog | AI045460 |
| 1910 | UI-R-E0-ci-g-07-0-UI | Heme oxygenase (decycling) 1 | AA874884 |
| 1911 | UI-R-A0-ae-d-05-0-UI | CD44 antigen | AA817820 |
| 1912 | UI-R-A0-ap-g-09-0-UI | Progesterone receptor membrane component 2 | BF548285 |
| 1913 | UI-R-E1-fa-g-02-0-UI | Zinc responsive protein ZD7 | BF559317 |
| 1914 | UI-R-E1-gb-h-08-0-UI | Brain-enriched guanylate kinase-associated | AA957534 |
| 1915 | UI-R-A0-bh-a-06-0-UI | Ras homolog gene family, member A | BF548046 |
| 1916 | UI-R-E1-fb-d-09-0-UI | BCL2/adenovirus E1B 19kDa-interacting protein 1 | BF559338 |
| 1917 | UI-R-C0-gt-d-12-0-UI | NP_446134.2 1 | AA963763 |
| 1918 | UI-R-A0-az-f-01-0-UI | XP_001090113.1 ribosomal protein S18 isoform 1 | AA818640 |
| 1919 | UI-R-A0-bh-e-12-0-UI | Mitochondrial ribosomal protein L3 | BF547719 |
| 1920 | UI-R-A0-ay-b-11-0-UI | Dehydrodolichyl diphosphate synthase | BF549737 |
| 1921 | UI-R-A1-es-e-06-0-UI | Hypothetical LOC301124 | BF558385 |
| 1922 | UI-R-C0-gt-g-11-0-UI | Tetraspanin 4 | BF557245 |
| 1923 | UI-R-C0-gz-d-01-0-UI | Zinc finger protein 444 | AA965102 |
| 1924 | UI-R-E0-cf-a-06-0-UI | Nuclear pore membrane glycoprotein 210 | AA874962 |
| 1925 | UI-R-E0-ck-g-06-0-UI | Biglycan | AA874917 |
| 1926 | UI-R-E1-fg-g-12-0-UI | Somatostatin | AA955767 |
| 1927 | UI-R-E1-ft-f-02-0-UI | Lamin B1 | AA957201 |
| 1928 | UI-R-E1-ge-b-07-0-UI | Alpha-fetoprotein | AA957057 |
| 1929 | UI-R-C0-ig-h-08-0-UI | Leptin receptor | AA998983 |
| 1930 | UI-R-E1-fc-h-07-0-UI | Thioredoxin reductase 1 | BF556766 |
| 1931 | UI-R-C0-gs-g-05-0-UI | Transcribed locus | AA963995 |
| 1932 | UI-R-C0-iv-h-12-0-UI | V-raf-1 murine leukemia viral oncogene homolog 1 | BF552670 |
| 1933 | UI-R-C1-jv-b-01-0-UI | EF hand calcium binding protein 2 | BF551174 |
| 1934 | UI-R-C2-mv-g-10-0-UI | XP_001139181.1 CIN85-associated multi-domain containing RhoGAP 1 isoform 7 [Pan troglodytes] | AI070642 |
| 1935 | UI-R-C2-na-a-01-0-UI | Polymerase (DNA directed), epsilon 2 (p59 subunit) | AI070935 |
| 1936 | UI-R-C2-nb-h-10-0-UI | Nuclear factor, erythroid derived 2,-like 1 | AI071430 |
| 1937 | UI-R-C2-ne-a-04-0-UI | Mitochondrial ribosomal protein L54 | AI072256 |
| 1938 | UI-R-C2p-nq-a-09-0-UI | Ectonucleoside triphosphate diphosphohydrolase 1 | AI136665 |
| 1939 | UI-R-C2p-nu-e-07-0-UI | Procollagen, type VII, alpha 1 | BF553365 |
| 1940 | UI-R-C2p-nx-g-02-0-UI | Ectonucleoside triphosphate diphosphohydrolase 6 | BF553658 |
| 1941 | UI-R-C2p-ob-b-03-0-UI | scaffold attachment factor B2 | AI136591 |
| 1942 | UI-R-E0-bt-e-06-0-UI | Follistatin | AA858520 |
| 1943 | UI-R-E0-bz-a-06-0-UI | Transketolase | AA899042 |
| 1944 | UI-R-C0-ii-f-11-0-UI | XP_001083483.1 cleavage stimulation factor subunit 3 isoform 1 | AA998749 |
| 1945 | UI-R-C0-ik-g-06-0-UI | Rho GTPase activating protein 9 | AI029056 |
| 1946 | UI-R-C0-jp-g-09-0-UI | Ubiquitin specific peptidase 2 | AI031057 |
| 1947 | UI-R-C1-jx-f-04-0-UI | Transcribed locus | BF550863 |
| 1948 | UI-R-C1-jz-a-10-0-UI | AP1 gamma subunit binding protein 1 | AI045438 |
| 1949 | UI-R-C1-kc-a-01-0-UI | hypothetical protein 3010020C06 | AI044595 |
| 1950 | UI-R-C1-kq-g-04-0-UI | Transmembrane protein 27 | BF550608 |
| 1951 | UI-R-C1-ks-f-05-0-UI | Pleckstrin homology domain-containing, family A (phosphoinositide binding specific) member 3 | BF552567 |
| 1952 | UI-R-C1-kx-c-06-0-UI | Mediator of RNA polymerase II transcription, subunit 19 homolog | BF546364 |
| 1953 | UI-R-C1-kz-d-07-0-UI | Zinc finger protein 637 | BF547111 |
| 1954 | UI-R-A0-al-f-10-0-UI | Cytochrome c, somatic | BF549065 |
| 1955 | UI-R-A0-ap-h-08-0-UI | XP_001099027.1 heat shock 90kDa protein 1, beta isoform 6 | AA819777 |
| 1956 | UI-R-A0-as-d-10-0-UI | SEC13-like 1 | AA818945 |
| 1957 | UI-R-A0-be-b-05-0-UI | hypothetical protein FLJ10241 | AA858719 |
| 1958 | UI-R-E1-fo-g-08-0-UI | Fatty acid desaturase 2 | BF560999 |
| 1959 | UI-R-E1-fu-a-10-0-UI | XP_001086849.1 mitochondrial carrier triple repeat 1 | AA957383 |
| 1960 | UI-R-E1-gp-g-09-0-UI | Nipsnap homolog 3A | AI113030 |
| 1961 | UI-R-C0-gw-c-12-0-UI | LSM4 homolog, U6 small nuclear RNA associated | AA964080 |
| 1962 | UI-R-C0-ib-a-10-0-UI | Transcribed locus |  |
| 1963 | UI-R-C0-id-f-05-0-UI | RalBP1 associated Eps domain containing protein | AA998078 |
| 1964 | UI-R-C0-gx-h-10-0-UI | Glycogen synthase kinase 3 beta | BF557900 |
| 1965 | UI-R-C0-he-d-04-0-UI | POU domain, class 3, transcription factor 4 | AA996525 |
| 1966 | UI-R-C0-jl-e-10-0-UI | Growth associated protein 43 | AI043649 |
| 1967 | UI-R-C1-js-b-05-0-UI | Runt related transcription factor 1 | AI044037 |
| 1968 | UI-R-C1-jx-b-09-0-UI | XP_001104964.1 glycogen synthase kinase 3 alpha isoform 2 | AI044641 |
| 1969 | UI-R-C1-ke-c-01-0-UI | Prostaglandin E receptor 4 (subtype EP4) | BF546471 |
| 1970 | UI-R-C1-lp-b-12-0-UI | Inositol 1,4,5-triphosphate receptor 2 | BF559954 |
| 1971 | UI-R-C2-mu-e-03-0-UI | P21 (CDKN1A)-activated kinase 1 | AI072234 |
| 1972 | UI-R-C2-ne-c-04-0-UI | Arylsulfatase B | AI072268 |
| 1973 | UI-R-C1-ks-b-10-0-UI | Protein phosphatase 1, regulatory subunit 10 | AI059047 |
| 1974 | UI-R-DK0-cfy-h-01-0-UI | Transforming, acidic coiled-coil containing protein 2 | BI290740 |
| 1975 | UI-R-DK0-cgf-b-12-0-UI | Spastic paraplegia 7 homolog | BI291078 |
| 1976 | UI-R-CV2-cgp-d-02-0-UI | mKIAA1377 protein | BI291375 |
| 1977 | UI-R-CV2-chb-g-12-0-UI | ATPase, Cu++ transporting, beta polypeptide | BI297889 |
| 1978 | UI-R-DR0-cjb-p-06-0-UI | Proline arginine-rich end leucine-rich repeat protein | BI303936 |
| 1979 | UI-R-A1-do-e-12-0-UI | Sertoli cell protein 1 | BF558619 |
| 1980 | UI-R-A1-dt-a-08-0-UI | XP_001114034.1 Glyceraldehyde-3-phosphate dehydrogenase (GAPDH) isoform 1 | AA924111 |
| 1981 | UI-R-A1-dw-b-08-0-UI | Sterol carrier protein 2 | BF555917 |
| 1982 | UI-R-E1-gj-e-04-0-UI | Selenoprotein P, plasma, 1 | AA963445 |
| 1983 | UI-R-C0-gt-g-12-0-UI | Syntaxin binding protein 5 (tomosyn) | AA964120 |
| 1984 | UI-R-DC0-bzj-e-07-0-UI | Deoxyribonuclease II beta | BI281394 |
| 1985 | UI-R-DD0-caa-d-01-0-UI | Interleukin 18 | BI285455 |
| 1986 | UI-R-CW0s-cci-b-02-0-UI | Ribosomal protein L19 | BI287499 |
| 1987 | UI-R-CX0s-ccq-h-09-0-UI | Regulator of G-protein signaling 5 | BI284331 |
| 1988 | UI-R-CX0s-ccx-c-01-0-UI | Cathepsin Q | BI283831 |
| 1989 | UI-R-DK0-cde-e-03-0-UI | Protein kinase C, epsilon | BI288749 |
| 1990 | UI-R-DK0-cev-f-04-0-UI | Protein kinase, AMP-activated, beta 2 non-catalytic subunit | BI295543 |
| 1991 | UI-R-DK0-cfb-h-10-0-UI | hypothetical protein FLJ20898 | BI295889 |
| 1992 | UI-R-DK0-cfg-b-10-0-UI | LOC360807 | BI289373 |
| 1993 | UI-R-CA1-bjt-a-12-0-UI | Karyopherin (importin) beta 1 | BF410061 |
| 1994 | UI-R-BT1-bmp-h-04-0-UI | XP_001083858.1 DDHD domain containing 1 isoform 2 | BF411640 |
| 1995 | UI-R-BT1-bnj-f-07-0-UI | XP_001103515.1 pentraxin-related gene, rapidly induced by IL-1 beta | BG378695 |
| 1996 | UI-R-CA0-bod-b-03-0-UI | XP_001106227.1 WW domain-containing adapter with a coiled-coil region isoform 1 | BF413013 |
| 1997 | UI-R-BJ2-boo-b-08-0-UI | Cyclin H | CK844518 |
| 1998 | UI-R-BJ2-bqm-b-09-0-UI | Zinc finger and BTB domain containing 17 | BF418981 |
| 1999 | UI-R-BJ2-bqq-b-12-0-UI | C1q and tumor necrosis factor related protein 4 | CK843795 |
| 2000 | UI-R-CW0-bvz-e-08-0-UI | DEAH (Asp-Glu-Ala-His) box polypeptide 30 | CK845496 |
| 2001 | UI-R-CW0-bwj-e-05-0-UI | Translocase of inner mitochondrial membrane 23 homolog | BI274493 |
| 2002 | UI-R-DA0-byj-e-04-0-UI | Cytochrome c oxidase subunit IV isoform 1 | BI279120 |
| 2003 | UI-R-DB0-byu-c-08-0-UI | Metadherin | BI284940 |
| 2004 | UI-R-CA0-bgd-h-08-0-UI | Fucosyltransferase 2 (secretor status included) | BF393612 |
| 2005 | UI-R-CA0-bgr-f-04-0-UI | Transcribed locus | BF394328 |
| 2006 | UI-R-CA1-bjb-j-14-0-UI | Mitogen-activated protein kinase 8 interacting protein 3 | BF399528 |
| 2007 | UI-R-CA1-bje-k-13-0-UI | Zinc finger, CCHC domain containing 12 | BF405776 |
| 2008 | UI-R-CV0-brk-h-01-0-UI | Lens intrinsic membrane protein 2 | BG371983 |
| 2009 | UI-R-CV1-brz-d-12-0-UI | Snf2-related CBP activator protein | BG372695 |
| 2010 | UI-R-CT0-buk-d-06-0-UI | Transcribed locus | BG381531 |
| 2011 | UI-R-CU0-bus-e-02-0-UI | SMT3 suppressor of mif two 3 homolog 1 | BG376263 |
| 2012 | UI-R-CU0-bvd-b-05-0-UI | Selenoprotein | BG377471 |
| 2013 | UI-R-CS0-bvm-b-09-0-UI | DnaJ (Hsp40) homolog, subfamily C, member 15 | BG377008 |
| 2014 | UI-R-CA0-axf-h-03-0-UI | Plasticity related protein 4 | BE108576 |
| 2015 | UI-R-CA0-axr-f-02-0-UI | Sema domain, immunoglobulin domain (Ig), transmembrane domain (TM) and short cytoplasmic domain, (semaphorin) 4F | BE115296 |
| 2016 | UI-R-BS1-ayd-h-02-0-UI | PHD finger protein 12 | BE116664 |
| 2017 | UI-R-BS1-ays-h-05-0-UI | Hemoglobin, epsilon 1 | BE107364 |
| 2018 | UI-R-CA0-baw-c-09-0-UI | Ret proto-oncogene | BE121300 |
| 2019 | UI-R-CA1-bbh-e-08-0-UI | erythrocyte membrane protein band 4.1 (elliptocytosis 1, RH-linked) isoform 1 | BF386793 |
| 2020 | UI-R-CA1-bby-e-04-0-UI | RIKEN cDNA 2700085E05 | BF388122 |
| 2021 | UI-R-CA1-bcg-e-05-0-UI | Unc-13 homolog A | BF390396 |
| 2022 | UI-R-BS2-ber-e-05-0-UI | hypothetical protein | BF398669 |
| 2023 | UI-R-CA0-bfl-f-04-0-UI | solute carrier family 25 (mitochondrial carrier; phosphate carrier), member 23 | BF392646 |
| 2024 | UI-R-BS0-aoe-a-02-0-UI | Tripartite motif protein 26 | AW535339 |
| 2025 | UI-R-BU0-apd-h-01-0-UI | RIKEN cDNA 1500031L02 | BE095973 |
| 2026 | UI-R-BX0-ary-g-04-0-UI | Cytoplasmic FMR1 interacting protein 2 | BE105837 |
| 2027 | UI-R-BO1-asl-b-08-0-UI | RAR-related orphan receptor alpha | BE106199 |
| 2028 | UI-R-BJ1-asw-g-05-0-UI | Chemokine (C-X-C motif) ligand 11 | CK844826 |
| 2029 | UI-R-BJ1-atg-g-10-0-UI | D8Ertd354e protein | CK842859 |
| 2030 | UI-R-BJ1-aus-c-06-0-UI | Transcribed locus | CK839820 |
| 2031 | UI-R-BJ1-avf-f-12-0-UI | Potential phospholipid-transporting ATPase IIB | BE110154 |
| 2032 | UI-R-BJ1-avp-d-09-0-UI | FYVE, RhoGEF and PH domain containing 1 | BE109546 |
| 2033 | UI-R-BJ1-avv-b-12-0-UI | Ninjurin 2 | CK845542 |
| 2034 | UI-R-BO0-aia-e-05-0-UI | Transmembrane protein 24 | BF567474 |
| 2035 | UI-R-BO0-aih-c-08-0-UI | Sterol regulatory element binding factor 2 | BF562965 |
| 2036 | UI-R-BJ0p-aip-g-05-0-UI | N-myc downstream regulated gene 4 | AW525304 |
| 2037 | UI-R-BJ0p-aiu-g-05-0-UI | ATPase, H+ transporting, V1 subunit C, isoform 2 | AW525693 |
| 2038 | UI-R-BT1-ajy-c-10-0-UI | NK6 transcription factor related, locus 1 | AW527709 |
| 2039 | UI-R-BT1-akg-d-10-0-UI | Glutamate receptor interacting protein 1 | AW527989 |
| 2040 | UI-R-BT1-akm-c-02-0-UI | T-cell receptor gamma chain (TCRg; C4L) | BF565578 |
| 2041 | UI-R-BT1-akv-c-04-0-UI | XP_001099877.1 mitochondrial isoleucine tRNA synthetase | BF563902 |
| 2042 | UI-R-BU0-amv-a-11-0-UI | RIKEN cDNA 2310042G06 | BF564685 |
| 2043 | UI-R-BU0-and-b-05-0-UI | Calcitonin-related polypeptide, beta | BF562364 |
| 2044 | UI-R-AB1-yu-e-02-0-UI | Dopamine beta hydroxylase | BF525224 |
| 2045 | UI-R-AE1-zb-f-04-0-UI | XP_001100436.1 parkin isoform 1 | CK840944 |
| 2046 | UI-R-AF1-aay-c-06-0-UI | Hypothetical protein LOC690120 | CK845672 |
| 2047 | UI-R-Y0-abh-d-12-0-UI | Type I keratin KA16 | AI763472 |
| 2048 | UI-R-Y0-abv-e-01-0-UI | Keratin complex 1, acidic, gene 18 | AI764567 |
| 2049 | UI-R-Y0-acd-h-04-0-UI | Advanced glycosylation end product-specific receptor | AI716132 |
| 2050 | UI-R-BJ0p-afd-g-05-0-UI | Kua homolog | CK839250 |
| 2051 | UI-R-BJ0p-afl-d-08-0-UI | Myotrophin | CK841489 |
| 2052 | UI-R-BJ0p-afq-g-01-0-UI | WD repeat domain 24 | CK845311 |
| 2053 | UI-R-BJ0p-aga-f-11-0-UI | Transcription elongation factor B (SIII), polypeptide 1 | CK841696 |
| 2054 | UI-R-C2p-rm-c-05-0-UI | Zinc finger protein 395 | BF547400 |
| 2055 | UI-R-C2p-rw-c-08-0-UI | N-myristoyltransferase 2 | BF522952 |
| 2056 | UI-R-C2p-se-a-10-0-UI | Proteosome (prosome, macropain) subunit, beta type 8 | BF542642 |
| 2057 | UI-R-C3-sm-f-09-0-UI | Epidermal growth factor receptor pathway substrate 8 | BF542677 |
| 2058 | UI-R-G0-uc-g-06-0-UI | SRY-box containing gene 17 | AI574719 |
| 2059 | UI-R-G0-uq-b-03-0-UI | K+ voltage-gated channel, subfamily S, 1 | AI576510 |
| 2060 | UI-R-Y0-uz-b-09-0-UI | UPF3 regulator of nonsense transcripts homolog A | BF524182 |
| 2061 | UI-R-Y0-vk-d-08-0-UI | RAS protein-specific guanine nucleotide-releasing factor 1 | AI577569 |
| 2062 | UI-R-AF0-yb-e-10-0-UI | Procollagen-proline, 2-oxoglutarate 4-dioxygenase (proline 4-hydroxylase), alpha polypeptide III | CK838808 |
| 2063 | UI-R-AC0-yl-g-05-0-UI | Potassium voltage-gated channel, subfamily H (eag-related), member 2 | BF525130 |
| 2064 | UI-R-E1-gb-e-12-0-UI | Peripheral myelin protein 22 | AA957503 |
| 2065 | UI-R-E1-gk-g-08-0-UI | Chromodomain helicase DNA binding protein 4 | AA963793 |
| 2066 | UI-R-C0-jk-g-04-0-UI | Stress 70 protein chaperone, microsome-associated, 60kD human homolog | AI043908 |
| 2067 | UI-R-C1-kh-g-02-0-UI | Nuclear receptor coactivator 5 | BF546940 |
| 2068 | UI-R-C1-lb-d-09-0-UI | Splicing factor, arginine/serine-rich 15 | BF523610 |
| 2069 | UI-R-C1-lq-c-01-0-UI | Ectonucleoside triphosphate diphosphohydrolase 2 | CK840738 |
| 2070 | UI-R-C2p-oj-f-02-0-UI | XP_001107578.1 potassium channel tetramerisation domain containing 11 | AI137059 |
| 2071 | UI-R-BT0-pq-b-08-0-UI | RNA binding motif protein 8 | AI145282 |
| 2072 | UI-R-BT0-pv-a-03-0-UI | CGI-112 protein | AI145182 |
| 2073 | UI-R-BT0-qe-g-03-0-UI | RIKEN cDNA 1700012G19 gene | AI145412 |
| 2074 | UI-R-A0-ap-e-03-0-UI | Transcribed locus |  |
| 2075 | UI-R-A0-bc-b-12-0-UI | XP_001071789.1 RAP2A, member of RAS oncogene family | BF549372 |
| 2076 | UI-R-E0-bo-h-03-0-UI | Transmembrane and ubiquitin-like domain containing 1 | BF547936 |
| 2077 | UI-R-E0-bt-g-01-0-UI | RIKEN cDNA 2310057D15 | CK845253 |
| 2078 | UI-R-E0-cz-f-07-0-UI | hypothetical protein FLJ10496 | BF555603 |
| 2079 | UI-R-E0-dg-b-07-0-UI | ARP3 actin-related protein 3 homolog | BF558541 |
| 2080 | UI-R-A1-do-d-07-0-UI | CD163 antigen | AA901107 |
| 2081 | UI-R-A1-ds-c-11-0-UI | hypothetical protein FLJ23514 | BF558744 |
| 2082 | UI-R-A1-ew-a-04-0-UI | hypothetical protein MGC6696 | AA955303 |
| 2083 | UI-R-E1-fi-b-09-0-UI | Hypothetical LOC289568 | BF560928 |
| 2084 | UI-R-CV1-bvq-c-10-0-UI | Phosphomevalonate kinase | BG378288 |
| 2085 | UI-R-DK0-cde-e-05-0-UI | Amino acid transporter | BI288751 |
| 2086 | UI-R-E1-gh-e-11-0-UI | growth arrest-specific 2 like 3 | BF560247 |
| 2087 | UI-R-C0-hp-g-04-0-UI | Protocadherin 19 | AA997710 |
| 2088 | UI-R-C0-ix-c-09-0-UI | RIKEN cDNA 1700027M01 | AI501945 |
| 2089 | UI-R-C1-kw-f-09-0-UI | ATP-binding cassette transporter sub-family A member 9 | AI059440 |
| 2090 | UI-R-C2p-re-b-01-0-UI | Pre-B-cell leukemia transcription factor 3 (Homeobox protein PBX3) | AI555823 |
| 2091 | UI-R-C2p-rt-c-04-0-UI | hypothetical protein B230397C21 | BF523111 |
| 2092 | UI-R-C0-ir-f-02-0-UI | Glutamyl-prolyl-tRNA synthetase | AI029450 |
| 2093 | UI-R-C0-jb-b-12-0-UI | Mitochondrial ribosomal protein L4 | AI030210 |
| 2094 | UI-R-C1-km-c-04-0-UI | ADP-ribosylation factor guanine nucleotide-exchange factor 1(brefeldin A-inhibited) | AI045928 |
| 2095 | UI-R-C1-ku-g-04-0-UI | EMG1 nucleolar protein homolog | CK840717 |
| 2096 | UI-R-C1-lj-h-07-0-UI | AF4/FMR2 family, member 3 | AI059612 |
| 2097 | UI-R-C1-ln-g-11-0-UI | hypothetical protein DKFZp434G156 | BF559937 |
| 2098 | UI-R-C0-hc-g-07-0-UI | RIKEN cDNA 1810043G02; DNA segment, Chr 10, Johns Hopkins University 13, expressed | AA965140 |
| 2099 | UI-R-C0-jb-h-04-0-UI | Dendritic cell inhibitory receptor 3 | BF551827 |
| 2100 | UI-R-C1-ku-f-02-0-UI | Enoyl Coenzyme A hydratase domain containing 1 | AI071462 |
| 2101 | UI-R-Y0-lu-c-12-0-UI | Shwachman-Bodian-Diamond syndrome homolog | AI070122 |
| 2102 | UI-R-C0-ih-c-09-0-UI | Sirtuin (silent mating type information regulation 2 homolog) 2 | AA999000 |
| 2103 | UI-R-C0-jd-e-12-0-UI | Mitochondrial ribosomal protein L16 | AI030663 |
| 2104 | UI-R-C1-kl-d-02-0-UI | Exocyst complex component 3 | BF551697 |
| 2105 | UI-R-Y0-ls-c-01-0-UI | TRNA selenocysteine associated protein | AI070074 |
| 2106 | UI-R-A1-ev-g-02-0-UI | ADP-ribosyltransferase 3 | BF556968 |
| 2107 | UI-R-E1-fr-h-11-0-UI | XP_001082398.1 mab-21-like protein 2 | AA956798 |
| 2108 | UI-R-E1-fx-a-04-0-UI | Coatomer protein complex, subunit epsilon | AA963071 |
| 2109 | UI-R-E1-gk-c-02-0-UI | mKIAA0674 protein | BF559848 |
| 2110 | UI-R-C0-ih-f-12-0-UI | Mitochondrial ribosomal protein S5 | BF554023 |
| 2111 | UI-R-C0-in-b-04-0-UI | KIAA1822 protein | AA999078 |
| 2112 | UI-R-A1-es-f-04-0-UI | Phospholipase A2, group IIA (platelets, synovial fluid) | AA925908 |
| 2113 | UI-R-A1-ey-f-05-0-UI | Lipoprotein lipase | AI385256 |
| 2114 | UI-R-C0-ip-c-10-0-UI | Cadherin EGF LAG seven-pass G-type receptor 3 | AI029122 |
| 2115 | UI-R-Y0-lu-f-03-0-UI | Cytochrom c oxidase subunit VIII-H (heart/muscle) | AI070222 |
| 2116 | UI-R-BT0-ps-f-09-0-UI | Solute carrier family 22, member 3 | AI145645 |
| 2117 | UI-R-A0-ax-c-08-0-UI | Ribosomal protein S3 | AA818272 |
| 2118 | UI-R-C1-ln-c-03-0-UI | Notch gene homolog 1 | BF552226 |
| 2119 | UI-R-A0-ap-e-12-0-UI | Phosphoribosylglycinamide formyltransferase | BF548279 |
| 2120 | UI-R-E0-cf-b-08-0-UI | Nuclear protein E3-3 | AA874973 |
| 2121 | UI-R-A1-ew-d-01-0-UI | B-cell translocation gene 2, anti-proliferative | AI146192 |
| 2122 | UI-R-C2-ng-b-04-0-UI | DnaJ (Hsp40) homolog, subfamily B, member 10 isoform 2 | AI072308 |
| 2123 | UI-R-C2-ni-d-09-0-UI | Plasminogen | AI072536 |
| 2124 | UI-R-C2-nk-c-11-0-UI | Nuclear distribution gene E homolog 1 | AI072673 |
| 2125 | UI-R-C2-nm-f-07-0-UI | Fragile X mental retardation gene 2, autosomal homolog | BF552837 |
| 2126 | UI-R-A0-ak-a-09-0-UI | Solute carrier family 2 (facilitated glucose transporter), member 4 | AA859020 |
| 2127 | UI-R-A0-aw-c-05-0-UI | Branched chain ketoacid dehydrogenase kinase | AA819287 |
| 2128 | UI-R-A0-bd-c-07-0-UI | Tubulin, beta 5 | AA858888 |
| 2129 | UI-R-A0-bl-c-10-0-UI | Actin related protein 2/3 complex, subunit 1B | AA819506 |
| 2130 | UI-R-E0-ct-h-03-0-UI | FXYD domain-containing ion transport regulator 4 | AA875655 |
| 2131 | UI-R-E0-dd-f-06-0-UI | NP_005942.1 1H | AA900218 |
| 2132 | UI-R-C0-jh-e-12-0-UI | SNAP-associated protein | AI030622 |
| 2133 | UI-R-C0-jl-c-12-0-UI | Phospholipase A2, group IVB (cytosolic) | AI043639 |
| 2134 | UI-R-C1-ke-c-11-0-UI | Sushi domain containing 2 | AI044986 |
| 2135 | UI-R-C1-kh-a-06-0-UI | Beta-galactosidase-like protein | BF554322 |
| 2136 | UI-R-C1-kj-f-01-0-UI | Sorbin and SH3 domain containing 3 | BF547002 |
| 2137 | UI-R-C1-kl-b-02-0-UI | STEAP family member 3 | BF546154 |
| 2138 | UI-R-C1-ld-c-04-0-UI | Actin-like 6A | AI059237 |
| 2139 | UI-R-C1-lf-f-07-0-UI | Kalirin, RhoGEF kinase | AI059340 |
| 2140 | UI-R-C1-lp-c-12-0-UI | Aminoacylase 1 | AI070169 |
| 2141 | UI-R-Y0-lv-b-10-0-UI | Zinc finger protein 110 | AI070500 |
| 2142 | UI-R-A1-ez-d-02-0-UI | T-box 2 | AA955150 |
| 2143 | UI-R-E1-ff-g-10-0-UI | hypothetical protein FLJ20003 | AA955875 |
| 2144 | UI-R-E1-fk-c-08-0-UI | Enhancer of zeste homolog 2 (ENX-1) | AA956585 |
| 2145 | UI-R-E1-fm-g-02-0-UI | Macrophage erythroblast attacher | AI113101 |
| 2146 | UI-R-C0-hd-b-10-0-UI | PHF21A protein | BF560036 |
| 2147 | UI-R-C0-hh-d-05-0-UI | Nedd4 binding protein 1 | BF561235 |
| 2148 | UI-R-C0-hj-d-06-0-UI | MRS2-like, magnesium homeostasis factor | BF554048 |
| 2149 | UI-R-C0-hv-f-07-0-UI | jumonji domain containing 2B | BF554356 |
| 2150 | UI-R-C0-iu-g-08-0-UI | Tumor necrosis factor receptor superfamily, member 1b | BF552637 |
| 2151 | UI-R-C0-ja-c-09-0-UI | Immunoglobulin superfamily, member 4A | AI029931 |
| 2152 | UI-R-C0-hw-b-03-0-UI | Sodium channel, voltage-gated, type I, beta | AA997543 |
| 2153 | UI-R-C0-jd-a-01-0-UI | Casein kinase 1, alpha 1 | AI030720 |
| 2154 | UI-R-C1-kl-a-12-0-UI | Secreted frizzled-related protein 4 | BF546153 |
| 2155 | UI-R-C1-kt-e-03-0-UI | LIM motif-containing protein kinase 2 | BF552599 |
| 2156 | UI-R-C1-la-a-06-0-UI | TAF9-like RNA polymerase II, TATA box binding protein (TBP)-associated factor, 31kDa | AI059951 |
| 2157 | UI-R-C1-li-b-03-0-UI | Nuclear distribution gene C homolog | AI060169 |
| 2158 | UI-R-C2p-nz-b-10-0-UI | Jumonji domain containing 1A | AI136954 |
| 2159 | UI-R-C2p-om-f-07-0-UI | Transcribed locus | AI138116 |
| 2160 | UI-R-A0-ac-f-08-0-UI | Replication factor C (activator 1) 5 | BF522356 |
| 2161 | UI-R-A0-ah-g-01-0-UI | Coiled-coil domain containing 58 | AA818221 |
| 2162 | UI-R-CV2-cif-a-08-0-UI | DD6A4-12(7)-2 mRNA, partial sequence | BI300441 |
| 2163 | UI-R-DN0-cit-i-09-0-UI | EPS8-like 2 | BI301477 |
| 2164 | UI-R-DN0-civ-m-20-0-UI | Lectin, galactose binding, soluble 4 | BI292295 |
| 2165 | UI-R-DO0-cix-p-02-0-UI | Kinetochore associated 2 | BI302714 |
| 2166 | UI-R-A1-ea-g-11-0-UI | Tumor necrosis factor receptor superfamily, member 8 | BF556053 |
| 2167 | UI-R-A1-ee-c-03-0-UI | Thioredoxin reductase 2 | AA925357 |
| 2168 | UI-R-A1-ei-a-04-0-UI | Carboxypeptidase D | AI111925 |
| 2169 | UI-R-A1-eo-d-12-0-UI | Transcribed locus |  |
| 2170 | UI-R-C0-hl-d-07-0-UI | MAD homolog 4 | BF558126 |
| 2171 | UI-R-C0-hr-h-02-0-UI | Nuclear receptor subfamily 2, group F, member 6 | AA997437 |
| 2172 | UI-R-CU0s-cbw-f-04-0-UI | Low density lipoprotein receptor-related protein associated protein 1 | BI282106 |
| 2173 | UI-R-CW0s-ccb-c-06-0-UI | S100 calcium binding protein A16 | BI285535 |
| 2174 | UI-R-DK0-cdq-g-07-0-UI | ATP synthase, H+ transporting, mitochondrial F0 complex, subunit c (subunit 9) isoform 3 | BI293457 |
| 2175 | UI-R-DK0-cea-f-02-0-UI | Claudin 7 | BI294265 |
| 2176 | UI-R-DK0-ced-g-06-0-UI | Lectin, galactoside-binding, soluble 8 | BI294929 |
| 2177 | UI-R-DK0-ceg-d-05-0-UI | Cytoskeleton associated protein 2 | BI295150 |
| 2178 | UI-R-BJ2-bqw-d-12-0-UI | Receptor-interacting serine-threonine kinase 3 | BF407168 |
| 2179 | UI-R-BJ2-brc-f-09-0-UI | Dual-specificity tyrosine-(Y)-phosphorylation regulated kinase 1A | CK845450 |
| 2180 | UI-R-DK0-cfh-h-12-0-UI | Baculoviral IAP repeat-containing 2 | BI289513 |
| 2181 | UI-R-DK0-cfr-h-09-0-UI | Purinergic receptor P2X, ligand-gated ion channel, 3 | BI290107 |
| 2182 | UI-R-BJ2-box-a-05-0-UI | Guanine nucleotide binding protein-like 3 (nucleolar) | CK843063 |
| 2183 | UI-R-BJ2-bpk-g-04-0-UI | DNA methyltransferase 1-associated protein 1 | BF420258 |
| 2184 | UI-R-BJ2-bpr-h-08-0-UI | ARP8 actin-related protein 8 homolog | BF407125 |
| 2185 | UI-R-BJ2-bqg-d-11-0-UI | Suppressor of fused | CK843667 |
| 2186 | UI-R-CX0-bwx-a-06-0-UI | Microspherule protein 1 | BI278800 |
| 2187 | UI-R-CX0-bxi-b-11-0-UI | RIKEN cDNA 6030446I19 gene | BI275933 |
| 2188 | UI-R-CW0-bxo-g-08-0-UI | Protein tyrosine phosphatase 4a3 | BI276983 |
| 2189 | UI-R-CY0-bxy-e-12-0-UI | NADH dehydrogenase (ubiquinone) 1 beta subcomplex, 7 | BI277244 |
| 2190 | UI-R-CT0s-car-b-12-0-UI | Hyaluronoglucosaminidase 2 | BI286741 |
| 2191 | UI-R-CS0s-cbn-c-07-0-UI | PTEN induced putative kinase 1 | BI287302 |
| 2192 | UI-R-CA1-bih-d-03-0-UI | Poly(A) binding protein, cytoplasmic 1 | BF404600 |
| 2193 | UI-R-CA1-bix-c-04-0-UI | Protease, serine, 21 | BF405556 |
| 2194 | UI-R-CV1-bsl-a-10-0-UI | Decapping enzyme, scavenger | BG373978 |
| 2195 | UI-R-CV1-bsz-f-12-0-UI | M-phase phosphoprotein 10 (U3 small nucleolar ribonucleoprotein) | BG375380 |
| 2196 | UI-R-CS0-btt-a-10-0-UI | Ribosomal protein L32 | BG381342 |
| 2197 | UI-R-CT0-btz-f-08-0-UI | Capping protein (actin filament) muscle Z-line, beta | BG380639 |
| 2198 | UI-R-CA1-bjr-f-05-0-UI | Sodium channel, voltage-gated, type 2, alpha 1 polypeptide | BF409860 |
| 2199 | UI-R-CA0-bkp-e-03-0-UI | RAB8A, member RAS oncogene family | BF415969 |
| 2200 | UI-R-CN0-bld-a-02-0-UI | Goliath | BF417334 |
| 2201 | UI-R-CN0-blr-g-06-0-UI | Tumor protein D52-like 2 | BF417753 |
| 2202 | UI-R-BS1-azb-a-07-0-UI | Transcribed locus | BE109104 |
| 2203 | UI-R-BS1-azm-d-03-0-UI | Hemochromatosis type 2 (juvenile) homolog | BE117905 |
| 2204 | UI-R-CA0-azz-e-04-0-UI | Disabled homolog 2 interacting protein | BE119188 |
| 2205 | UI-R-CA0-bao-e-10-0-UI | Growth arrest-specific 2 like 1 | BE120011 |
| 2206 | UI-R-CA1-bcx-a-11-0-UI | Adrenergic receptor kinase, beta 2 | BF391408 |
| 2207 | UI-R-BS2-bdj-f-11-0-UI | HemK methyltransferase family member 1 | BF389426 |
| 2208 | UI-R-BS2-bdv-f-05-0-UI | Ribosomal protein L8 | BF396177 |
| 2209 | UI-R-BS2-bec-d-10-0-UI | XP_001111802.1 heterogeneous nuclear ribonucleoprotein R | BF397855 |
| 2210 | UI-R-CA0-bhb-f-09-0-UI | Development and differentiation enhancing factor 2 | BF394200 |
| 2211 | UI-R-CA0-bhp-c-07-0-UI | Synaptotagmin IV | BF402440 |
| 2212 | UI-R-BT1-aqn-a-12-0-UI | Hypothetical protein LOC361335 | BE102474 |
| 2213 | UI-R-BX0-ard-f-07-0-UI | Heterogeneous nuclear ribonucleoprotein A3 | BE103558 |
| 2214 | UI-R-BJ1-ato-c-12-0-UI | Loss of heterozygosity, 12, chromosomal region 1 homolog | CK842932 |
| 2215 | UI-R-BJ1-ats-d-11-0-UI | RIKEN cDNA 5730470L24 | CK841857 |
| 2216 | UI-R-BJ1-aua-f-02-0-UI | Striatin, calmodulin binding protein 4 | BE101233 |
| 2217 | UI-R-BJ1-auh-g-02-0-UI | Inosine 5-monophosphate dehydrogenase 2 | BE101155 |
| 2218 | UI-R-BJ1-awa-f-10-0-UI | Transmembrane protein 106C | BE112947 |
| 2219 | UI-R-BJ1-awg-c-10-0-UI | Surfeit 1 | BE113610 |
| 2220 | UI-R-BJ1-awp-d-05-0-UI | Synaptotagmin V | CK839999 |
| 2221 | UI-R-CA0-awy-e-04-0-UI | Solute carrier family 27 (fatty acid transporter), member 5 | BE107935 |
| 2222 | UI-R-BO1-ajb-b-06-0-UI | Cytochrome P450, family 2, subfamily J, polypeptide 4 | AW526350 |
| 2223 | UI-R-BO1-aji-c-04-0-UI | Autophagy-related 12 | BF563278 |
| 2224 | UI-R-BO1-ajm-c-02-0-UI | FK506 binding protein 1b | AW527161 |
| 2225 | UI-R-BO1-ajs-e-11-0-UI | XP_001062898.1 Protein C20orf160 | AW527286 |
| 2226 | UI-R-C4-ald-c-04-0-UI | Ataxin 2-like | BF564084 |
| 2227 | UI-R-C4-alk-e-05-0-UI | Glycine N-methyltransferase | BF564232 |
| 2228 | UI-R-C4-alq-h-08-0-UI | Sodium channel, voltage-gated, type IV, beta | AW534238 |
| 2229 | UI-R-BT1-ame-d-11-0-UI | Corticotropin releasing hormone | BE107668 |
| 2230 | UI-R-BU0-apm-d-10-0-UI | Sodium channel, voltage-gated, type I, alpha | BE096756 |
| 2231 | UI-R-BO1-aqb-g-12-0-UI | hypothetical protein FLJ31413 | BE097796 |
| 2232 | UI-R-AA1-aac-d-09-0-UI | Ankyrin repeat domain 1 (cardiac muscle) | CK843253 |
| 2233 | UI-R-AF1-aar-g-02-0-UI | Phospholipase D family, member 3 | CK840630 |
| 2234 | UI-R-Y0-acn-c-10-0-UI | Glutamate receptor, metabotropic 4 | AI763723 |
| 2235 | UI-R-Y0-acx-h-12-0-UI | Hypothetical protein LOC685888 | AI764357 |
| 2236 | UI-R-BJ0-adp-a-11-0-UI | Activating signal cointegrator 1 complex subunit 3-like 1 | AW251633 |
| 2237 | UI-R-BJ0-ael-e-04-0-UI | Dr1 associated protein 1 (negative cofactor 2 alpha) | AW252854 |
| 2238 | UI-R-BO0-agn-a-02-0-UI | Apelin, AGTRL1 ligand | BF566549 |
| 2239 | UI-R-BO0-agr-b-08-0-UI | Neuroglobin | AW521735 |
| 2240 | UI-R-BO0-ahk-e-06-0-UI | Polymerase (DNA-directed), delta 3, accessory subunit | AW522725 |
| 2241 | UI-R-BO0-aht-b-12-0-UI | Endothelial differentiation, sphingolipid G-protein-coupled receptor, 8 | AW523260 |
| 2242 | UI-R-C3-sv-d-02-0-UI | RAP1, GTP-GDP dissociation stimulator 1 | AI511284 |
| 2243 | UI-R-C3-th-h-12-0-UI | Adenosine monophosphate deaminase 2 (isoform L) | AI548001 |
| 2244 | UI-R-C3-tm-h-01-0-UI | ELL associated factor 1 | AI710045 |
| 2245 | UI-R-C3-tv-e-02-0-UI | Alpha-2,6-sialyltransferase ST6GalNAc IV | AI549013 |
| 2246 | UI-R-AD0-vz-e-03-0-UI | SCF apoptosis response protein 1 | CK838544 |
| 2247 | UI-R-AA0-wm-b-02-0-UI | Myosin-binding protein H (MyBP-H) (H-protein) | AI578796 |
| 2248 | UI-R-AG0-wx-g-09-0-UI | Heart and neural crest derivatives expressed transcript 1 | CK838606 |
| 2249 | UI-R-AE0-xm-c-06-0-UI | XP_001108236.1 small nuclear ribonucleoprotein D2 | CK844811 |
| 2250 | UI-R-AD1-zn-f-03-0-UI | ADP-ribosylation factor-like 2 | CK839697 |
| 2251 | UI-R-AD1-zt-f-12-0-UI | SCAN domain-containing 1 | CK841044 |
| 2252 | UI-R-C0-hu-c-11-0-UI | Megalencephalic leukoencephalopathy with subcortical cysts 1 homolog | BF550715 |
| 2253 | UI-R-C0-in-h-08-0-UI | RIB43A domain with coiled-coils 2 | AA999065 |
| 2254 | UI-R-Y0-mj-a-10-0-UI | SPI6 | AI112862 |
| 2255 | UI-R-C2-mu-f-12-0-UI | Splicing factor, arginine/serine-rich 3 (SRp20) | AI385370 |
| 2256 | UI-R-C2p-nr-f-01-0-UI | solute carrier family 25 (mitochondrial carrier, Aralar), member 12 | AI113127 |
| 2257 | UI-R-C2p-od-a-02-0-UI | Solute carrier family 30 (zinc transporter), member 7 | BF551969 |
| 2258 | UI-R-BT0-qk-f-01-0-UI | Telomeric repeat binding factor 2 | AI454691 |
| 2259 | UI-R-C2p-qq-c-10-0-UI | Voltage-dependent anion channel 1 | AI555458 |
| 2260 | UI-R-C2p-qv-a-03-0-UI | Slit homolog 3 | BF546784 |
| 2261 | UI-R-C2p-rg-d-08-0-UI | Protein-L-isoaspartate (D-aspartate) O-methyltransferase domain containing 2 | AI556351 |
| 2262 | UI-R-E0-bx-g-04-0-UI | CG13379-PA | BF547829 |
| 2263 | UI-R-E0-cd-c-04-0-UI | FCH domain only 2 | AI454404 |
| 2264 | UI-R-E0-ck-e-11-0-UI | Phospholipase A2, group XIIA | BF559420 |
| 2265 | UI-R-E0-cs-h-07-0-UI | DAZ associated protein 2 | AA875435 |
| 2266 | UI-R-A1-dx-c-12-0-UI | Peroxisomal membrane protein 4 | AA924231 |
| 2267 | UI-R-A1-ec-a-09-0-UI | C-src tyrosine kinase | AA925427 |
| 2268 | UI-R-A1-eh-a-12-0-UI | XP_001058647.1 Pappalysin-1 precursor (Pregnancy-associated plasma protein-A) (PAPP-A) (Insulin-like growth factor-dependent IGF-binding protein 4 protease) (IGF-dependent IGFBP-4 protease) (IGFBP-4ase) [Ra | AA924978 |
| 2269 | UI-R-A1-el-d-07-0-UI | Transcribed locus | AA926063 |
| 2270 | UI-R-C0-ha-a-09-0-UI | RGD1562079 | BF556728 |
| 2271 | UI-R-C0-ho-f-12-0-UI | RIKEN cDNA A430005L14 | AA997017 |
| 2272 | UI-R-C2p-ny-c-07-0-UI | Transcribed locus | AI136113 |
| 2273 | UI-R-G0-ui-f-10-0-UI | Potassium voltage-gated channel, Shal-related family, member 1 | AI578266 |
| 2274 | UI-R-BO0-ahp-a-02-0-UI | RGD1564982 | AW522894 |
| 2275 | UI-R-BJ1-avl-a-10-0-UI | Mucin 4 | CK842534 |
| 2276 | UI-R-A0-bk-g-02-0-UI | RIKEN cDNA D530033C11 | BF559289 |
| 2277 | UI-R-E0-cj-c-11-0-UI | hypothetical protein FLJ31810 | AA859317 |
| 2278 | UI-R-A1-dp-f-10-0-UI | Protein phosphatase 1 (formerly 2C)-like | BF558649 |
| 2279 | UI-R-A1-el-c-03-0-UI | hypothetical protein FLJ10986 | AA926048 |
| 2280 | UI-R-Y0-ls-a-03-0-UI | RIKEN cDNA 4931419K03 | AI070065 |
| 2281 | UI-R-C2p-nr-g-07-0-UI | Bruton agammaglobulinemia tyrosine kinase | AI113328 |
| 2282 | UI-R-C0-ha-h-01-0-UI | Hypothetical LOC305552 | AA965058 |
| 2283 | UI-R-C0-hg-c-11-0-UI | Transcribed locus | AA996749 |
| 2284 | UI-R-C0-jd-b-06-0-UI | Mitochondrial 28S ribosomal protein S28 (S28mt) (MRP-S28) | AI030729 |
| 2285 | UI-R-C0-jm-f-03-0-UI | Tax1 (human T-cell leukemia virus type I) binding protein 3 | AI043857 |
| 2286 | UI-R-C0-jr-c-09-0-UI | Outer dense fiber of sperm tails 4 | BF550808 |
| 2287 | UI-R-C1-ju-e-08-0-UI | Signal sequence receptor, beta | BF554156 |
| 2288 | UI-R-A0-au-d-09-0-UI | Nuclear pore associated protein | BF548292 |
| 2289 | UI-R-C0-gw-b-06-0-UI | Staphylococcal nuclease domain containing 1 | AA964063 |
| 2290 | UI-R-A0-bh-c-03-0-UI | Kinesin light chain 4 | BF547710 |
| 2291 | UI-R-A0-aw-e-11-0-UI | Hypothetical LOC308869 | AA818540 |
| 2292 | UI-R-E1-fj-b-06-0-UI | Rabaptin, RAB GTPase binding effector protein 1 | AA956332 |
| 2293 | UI-R-Y0-ls-h-01-0-UI | H3 histone, family 3B | BF545574 |
| 2294 | UI-R-A0-ba-h-09-0-UI | Leucine zipper transcription regulator 2 | CK845180 |
| 2295 | UI-R-E1-fw-h-05-0-UI | SUMO/sentrin specific protease 2 | AA957708 |
| 2296 | UI-R-C2-ms-c-04-0-UI | Bone morphogenetic protein 1 | BF542283 |
| 2297 | UI-R-E1-gb-a-11-0-UI | Transcribed locus | AA957590 |
| 2298 | UI-R-A0-aw-g-04-0-UI | Sec61, alpha subunit 2 | AA818555 |
| 2299 | UI-R-A0-az-h-10-0-UI | Syntaxin 18 | AA818672 |
| 2300 | UI-R-E1-gl-a-05-0-UI | Minichromosome maintenance deficient 3 | AA964217 |
| 2301 | UI-R-C0-gv-e-02-0-UI | RAB2B, member RAS oncogene family | AA964498 |
| 2302 | UI-R-E0-cb-e-02-0-UI | Proprotein convertase subtilisin/kexin type3 | AA875053 |
| 2303 | UI-R-E0-ch-e-07-0-UI | Epoxide hydrolase 1, microsomal | AA900551 |
| 2304 | UI-R-E1-fc-d-12-0-UI | Signal transducer and activator of transcription 5B | AA955730 |
| 2305 | UI-R-E1-fn-g-09-0-UI | Cyclin G1 | AA956549 |
| 2306 | UI-R-E1-fx-c-01-0-UI | Peptidylprolyl isomerase G | BF561963 |
| 2307 | UI-R-C0-hz-d-03-0-UI | Uncoupling protein 2 (mitochondrial, proton carrier) | AA998366 |
| 2308 | UI-R-A1-es-a-08-0-UI | Glyoxylase 1 | AA925987 |
| 2309 | UI-R-E1-fy-c-02-0-UI | Transforming growth factor beta 1 induced transcript 1 | AA957362 |
| 2310 | UI-R-C0-hf-d-08-0-UI | Casein kinase 1, epsilon | BF557886 |
| 2311 | UI-R-C0-je-e-04-0-UI | DEAD (Asp-Glu-Ala-Asp) box polypeptide 52 | AI030825 |
| 2312 | UI-R-Y0-lv-g-06-0-UI | Neuron-glia-CAM-related cell adhesion molecule | AI070357 |
| 2313 | UI-R-C2-mw-d-06-0-UI | BCL2/adenovirus E1B 19 kDa-interacting protein 3 | AI385294 |
| 2314 | UI-R-C2-na-h-03-0-UI | Docking protein 3 | AI071005 |
| 2315 | UI-R-C2-nd-a-12-0-UI | Nuclear import 7 homolog | BF553564 |
| 2316 | UI-R-C2-nn-f-03-0-UI | autophagy-related 10-like | BF552880 |
| 2317 | UI-R-C2p-nq-h-07-0-UI | La ribonucleoprotein domain family, member 1 | CN540039 |
| 2318 | UI-R-C2p-nw-d-05-0-UI | Orthopedia homolog | AI136560 |
| 2319 | UI-R-C2p-ny-d-12-0-UI | Ribonuclease P 40 subunit | BF552334 |
| 2320 | UI-R-E0-bo-b-09-0-UI | MAD homolog 2 | AA858489 |
| 2321 | UI-R-E0-bv-e-12-0-UI | Cofilin 1, non-muscle | BF549935 |
| 2322 | UI-R-C0-if-b-10-0-UI | Asparagine-linked glycosylation 8 homolog (yeast, alpha-1,3-glucosyltransferase) | AA998562 |
| 2323 | UI-R-C0-ij-f-04-0-UI | RGD1564171 | BF524350 |
| 2324 | UI-R-C0-jp-a-09-0-UI | YEATS domain containing 4 | AI031024 |
| 2325 | UI-R-C1-jw-g-09-0-UI | Hypothetical LOC302884 | AI044498 |
| 2326 | UI-R-C1-jy-b-07-0-UI | Protein disulfide isomerase associated 4 | AI045710 |
| 2327 | UI-R-C1-kb-c-06-0-UI | Ubiquitin specific protease 39 | AI044269 |
| 2328 | UI-R-C1-kq-a-06-0-UI | RIKEN cDNA 0610039J04 | BF550597 |
| 2329 | UI-R-C1-kr-f-10-0-UI | Scotin | AI059854 |
| 2330 | UI-R-C1-kw-c-03-0-UI | Oxidative-stress responsive 1 | BF547088 |
| 2331 | UI-R-C1-ky-d-11-0-UI | Transcribed locus | AI071556 |
| 2332 | UI-R-A0-aj-d-09-0-UI | Solute carrier family 34 (sodium phosphate), member 2 | AA858973 |
| 2333 | UI-R-A0-am-c-09-0-UI | Paraoxonase 3 | AA818163 |
| 2334 | UI-R-A0-aq-g-03-0-UI | DNA segment, Chr 13, Wayne State University 50, expressed | AA819805 |
| 2335 | UI-R-A0-au-h-11-0-UI | Insulin-like growth factor binding protein 7 | AA818445 |
| 2336 | UI-R-E1-fn-d-07-0-UI | Nephronophthisis 1 (juvenile) homolog | BF560909 |
| 2337 | UI-R-E1-fq-e-10-0-UI | Tumor necrosis factor receptor superfamily, member 12a | AA957270 |
| 2338 | UI-R-E1-fu-h-10-0-UI | Peptidylprolyl isomerase D (cyclophilin D) | AA957342 |
| 2339 | UI-R-C0-gr-a-12-0-UI | Interleukin 3 receptor, alpha chain | BF561113 |
| 2340 | UI-R-C0-hz-d-10-0-UI | Transcribed locus | AA998288 |
| 2341 | UI-R-C0-ic-a-11-0-UI | Cyclin A2 | AA998516 |
| 2342 | UI-R-C0-gw-a-12-0-UI | Ribosomal protein L37 | BF557788 |
| 2343 | UI-R-C0-ha-b-06-0-UI | Synaptonemal complex protein SC65 | AI137639 |
| 2344 | UI-R-C0-jg-g-10-0-UI | Cytochrome P450, family 7, subfamily a, polypeptide 1 | AI029671 |
| 2345 | UI-R-C0-jo-b-11-0-UI | Carboxypeptidase B1 (tissue) | AI030689 |
| 2346 | UI-R-C1-jv-b-11-0-UI | Chymotrypsinogen B | AI043606 |
| 2347 | UI-R-C1-kb-f-10-0-UI | Protein kinase C, beta 1 | BF550933 |
| 2348 | UI-R-C1-ll-a-05-0-UI | Calcitonin receptor-like | AI060121 |
| 2349 | UI-R-Y0-ls-d-05-0-UI | Hydroxyacyl-Coenzyme A dehydrogenase/3-ketoacyl-Coenzyme A thiolase/enoyl-Coenzyme A hydratase (trifunctional protein), beta subunit | AI070082 |
| 2350 | UI-R-C2-na-a-04-0-UI | Purinergic receptor P2X, ligand-gated ion channel, 1 | AI070938 |
| 2351 | UI-R-C2-nh-g-02-0-UI | Regulator of G-protein signaling 14 | BF553116 |
| 2352 | UI-R-DK0-cfw-c-10-0-UI | Apolipoprotein A-I | BI290439 |
| 2353 | UI-R-DK0-cga-e-04-0-UI | Bcl2-like 2 | BI290795 |
| 2354 | UI-R-DK0-cgj-f-06-0-UI | AlkB, alkylation repair homolog 4 | BI296965 |
| 2355 | UI-R-CV2-cgt-c-06-0-UI | Inhibitor of kappaB kinase beta | BI296613 |
| 2356 | UI-R-DQ0-cja-f-12-0-UI | Leukotriene B4 12-hydroxydehydrogenase | BI303615 |
| 2357 | UI-R-E0-dl-b-05-0-UI | Jagged 1 | BF558333 |
| 2358 | UI-R-A1-dr-b-08-0-UI | hypothetical protein MGC36831 | AA924345 |
| 2359 | UI-R-A1-du-e-03-0-UI | Transcribed locus | AA924058 |
| 2360 | UI-R-E1-gh-a-08-0-UI | Synaptojanin 2 | AA963164 |
| 2361 | UI-R-E1-go-d-06-0-UI | Annexin A2 | BF556888 |
| 2362 | UI-R-DB0-byz-d-10-0-UI | XP_001110088.1 steroid 5 alpha-reductase 2-like 2 | BI285382 |
| 2363 | UI-R-DD0-bzt-h-08-0-UI | Keratinocytes proline-rich protein | BI286122 |
| 2364 | UI-R-CW0s-ccd-d-01-0-UI | Solute carrier family 39 (zinc transporter), member 3 | BI282635 |
| 2365 | UI-R-CX0s-cco-b-02-0-UI | Hexosaminidase B | BI284347 |
| 2366 | UI-R-CX0s-ccs-h-03-0-UI | Prostaglandin E synthase | BI284840 |
| 2367 | UI-R-DK0-cdb-h-01-0-UI | Chloride channel 3 | BI288519 |
| 2368 | UI-R-DK0-cem-a-07-0-UI | Breast cancer 2 | BI294583 |
| 2369 | UI-R-DK0-cey-h-05-0-UI | FCH domain only 1 | BI296308 |
| 2370 | UI-R-DK0-cfe-b-05-0-UI | Solute carrier family 6 (neurotransmitter transporter, GABA), member 13 | BI289072 |
| 2371 | UI-R-CM0-bjk-d-07-0-UI | Crystallin, alpha B | BF395419 |
| 2372 | UI-R-CA0-bly-d-08-0-UI | Deleted in bladder cancer chromosome region candidate 1 | BF417945 |
| 2373 | UI-R-BT1-bmv-a-05-0-UI | Membrane associated guanylate kinase, WW and PDZ domain containing 2 | BF411649 |
| 2374 | UI-R-BT1-bnq-d-07-0-UI | XP_001107786.1 CGI58 protein | BF412650 |
| 2375 | UI-R-CA0-boi-h-04-0-UI | Slit homolog 1 | BF413811 |
| 2376 | UI-R-BJ2-bqi-e-10-0-UI | RIKEN cDNA D330045A20 | BF418665 |
| 2377 | UI-R-BJ2-bqo-g-01-0-UI | LIM domain only 3 | BF419154 |
| 2378 | UI-R-CW0-bvv-f-01-0-UI | Replication protein A2 | CK845492 |
| 2379 | UI-R-CW0-bwg-b-11-0-UI | Interleukin enhancer binding factor 2 | BI274203 |
| 2380 | UI-R-CZ0-byf-g-08-0-UI | L-3-hydroxyacyl-Coenzyme A dehydrogenase, short chain | BI277727 |
| 2381 | UI-R-DA0-byn-d-05-0-UI | Kallikrein, submaxillary gland S3 | BI279353 |
| 2382 | UI-R-CA0-bfu-h-10-0-UI | Thrombospondin 2 | BF393523 |
| 2383 | UI-R-CA0-bgi-a-03-0-UI | postmeiotic segregation increased 1 | BF400964 |
| 2384 | UI-R-CA1-biy-f-07-0-UI | Ring finger protein 40 | BF403369 |
| 2385 | UI-R-CA1-bjd-e-11-0-UI | Nitric oxide synthase 1, neuronal | BF404281 |
| 2386 | UI-R-CA1-bjf-j-06-0-UI | Transcribed locus | BF406403 |
| 2387 | UI-R-CV1-brv-g-06-0-UI | A disintegrin and metalloprotease domain 10 | BG372575 |
| 2388 | UI-R-CT0-bud-e-12-0-UI | Transcribed locus | BG381631 |
| 2389 | UI-R-CT0-bun-c-08-0-UI | Tripartite motif protein 41 | BG376167 |
| 2390 | UI-R-CU0-buv-c-11-0-UI | Period homolog 3 | BG376734 |
| 2391 | UI-R-CU0-bvh-c-01-0-UI | RIKEN cDNA 4921521J11 | BG377960 |
| 2392 | UI-R-CA0-axd-b-11-0-UI | Distal-less homeobox 1 | BE112823 |
| 2393 | UI-R-CA0-axj-b-05-0-UI | Phospholipase C, beta 1 | BE110022 |
| 2394 | UI-R-BS1-axx-c-05-0-UI | XP_001106724.1 hippocampus abundant transcript 1 | BE115866 |
| 2395 | UI-R-BS1-ayo-a-01-0-UI | B-cell CLL/lymphoma 7C | BE117453 |
| 2396 | UI-R-CA0-baq-d-06-0-UI | Forkhead box G1 | BE120349 |
| 2397 | UI-R-CA0-baz-a-09-0-UI | Protein tyrosine phosphatase, non-receptor type 9 | BE119621 |
| 2398 | UI-R-CA1-bbn-e-01-0-UI | Transcribed locus | BF387532 |
| 2399 | UI-R-CA1-bcb-f-10-0-UI | Echinoderm microtubule associated protein like 2 | BF396132 |
| 2400 | UI-R-BS2-bek-d-06-0-UI | putative repair and recombination helicase RAD26L | BF397269 |
| 2401 | UI-R-BS2-bex-g-01-0-UI | Glutamine and serine rich 1 | BF397383 |
| 2402 | UI-R-BU0-anf-f-08-0-UI | CG5805-PA | BF562431 |
| 2403 | UI-R-BU0-aoz-a-10-0-UI | hypothetical protein FLJ22175 | BE095751 |
| 2404 | UI-R-BX0-arm-d-04-0-UI | CG9346-PA | BE104233 |
| 2405 | UI-R-BO1-asi-f-09-0-UI | Prodynorphin | BE105714 |
| 2406 | UI-R-BO1-asq-d-12-0-UI | Protein interacting with C kinase 1 | BE106646 |
| 2407 | UI-R-BJ1-asz-h-01-0-UI | RAS-related protein 1a | CK842660 |
| 2408 | UI-R-BJ1-aun-b-11-0-UI | HTGN29 protein; keratinocytes associated transmembrane protein 2 | BE110370 |
| 2409 | UI-R-BJ1-ava-e-07-0-UI | U1 small nuclear ribonucleoprotein polypeptide A | BE111329 |
| 2410 | UI-R-BJ1-avm-b-08-0-UI | hypothetical protein FLJ30973 | CK842556 |
| 2411 | UI-R-BJ1-avs-h-03-0-UI | Transporter 1, ATP-binding cassette, sub-family B (MDR/TAP) | CK844159 |
| 2412 | UI-R-BO0-ahx-h-12-0-UI | Hypothetical protein LOC502414 | AW524480 |
| 2413 | UI-R-BO0-aid-h-08-0-UI | CDKN1A interacting zinc finger protein 1 | AW524927 |
| 2414 | UI-R-BJ0p-aik-f-01-0-UI | Mesoderm specific transcript | CK843953 |
| 2415 | UI-R-BJ0p-air-f-03-0-UI | acyl-CoA thioesterase | CK841967 |
| 2416 | UI-R-BO1-aju-e-10-0-UI | Leucine rich repeat containing 23 | BF565343 |
| 2417 | UI-R-BT1-akc-c-10-0-UI | Myotubularin related protein 4 | BF566287 |
| 2418 | UI-R-BT1-akj-d-10-0-UI | RSA-14-44 protein | AW528859 |
| 2419 | UI-R-BT1-akq-b-03-0-UI | ATP-binding cassette, sub-family C (CFTR/MRP), member 5 | AW530677 |
| 2420 | UI-R-BU0-ams-b-09-0-UI | Filamin A interacting protein 1 | AW532783 |
| 2421 | UI-R-BU0-ana-a-10-0-UI | LIM homeobox protein 4 | BF564897 |
| 2422 | UI-R-AC0-yp-d-11-0-UI | Phosphatidylinositol glycan, class T | CK840800 |
| 2423 | UI-R-AB1-yy-d-01-0-UI | Nitrilase family, member 2 | CK840906 |
| 2424 | UI-R-AF1-aau-d-09-0-UI | Cytochrome c oxidase subunit VIIa polypeptide 2 like | CK838454 |
| 2425 | UI-R-Y0-abb-d-12-0-UI | Cholinergic receptor, nicotinic, delta polypeptide | AI712947 |
| 2426 | UI-R-Y0-abp-a-08-0-UI | Brain abundant, membrane attached signal protein 1 | AI715241 |
| 2427 | UI-R-Y0-acb-b-08-0-UI | yippee-like 3 | AI717442 |
| 2428 | UI-R-BJ0p-aex-e-04-0-UI | Mitochondrial ribosomal protein L44 | CK841155 |
| 2429 | UI-R-BJ0p-afg-c-10-0-UI | Pelota homolog | CK839315 |
| 2430 | UI-R-BJ0p-afn-b-07-0-UI | RIKEN cDNA E430021N18 | CK841543 |
| 2431 | UI-R-BJ0p-afw-d-05-0-UI | Arginine vasopressin-induced 1 | AW434282 |
| 2432 | UI-R-C2p-ri-f-08-0-UI | High mobility group protein 1 (HMG-1) (High mobility group protein B1) (Amphoterin) (Heparin-binding protein p30) | BF546277 |
| 2433 | UI-R-C2p-rq-f-07-0-UI | Tousled-like kinase 2 | BF546068 |
| 2434 | UI-R-C2p-sb-c-01-0-UI | Matrix metallopeptidase 7 | AI501445 |
| 2435 | UI-R-C3-sh-h-04-0-UI | Integrin, alpha D | BF542442 |
| 2436 | UI-R-C3-tz-e-01-0-UI | Cylindromatosis (turban tumor syndrome) | AI548849 |
| 2437 | UI-R-G0-uj-a-02-0-UI | Forkhead box D3 | BF523876 |
| 2438 | UI-R-G0-uu-g-11-0-UI | Polymerase (DNA directed), epsilon 3 (p17 subunit) | BF522722 |
| 2439 | UI-R-Y0-vc-g-01-0-UI | Leucine rich repeat protein 3, neuronal | BF523757 |
| 2440 | UI-R-AC1-xs-c-04-0-UI | Fragile X mental retardation syndrome 1 homolog | AI705393 |
| 2441 | UI-R-AC0-yh-e-05-0-UI | Transcribed locus |  |
| 2442 | UI-R-E1-fq-d-11-0-UI | Male-specific lethal-3 homolog 1 | AA957005 |
| 2443 | UI-R-E1-ge-h-02-0-UI | Heterogeneous nuclear ribonucleoprotein A2/B1 | AA957156 |
| 2444 | UI-R-C0-jb-b-08-0-UI | XP_001107022.1 mediator of RNA polymerase II transcription, subunit 12 homolog | AI501968 |
| 2445 | UI-R-C1-ju-d-08-0-UI | RAB11 family interacting protein 4 (class II) | AI045200 |
| 2446 | UI-R-C1-ko-d-12-0-UI | Glucosidase, alpha, acid | AI071306 |
| 2447 | UI-R-C1-li-g-01-0-UI | RRP22 | BF546718 |
| 2448 | UI-R-C2p-oh-a-11-0-UI | Nuclear receptor subfamily 5, group A, member 2 | AI137152 |
| 2449 | UI-R-BT0-pl-f-12-0-UI | DKFZP547E1010 protein | BF521864 |
| 2450 | UI-R-BT0-ps-b-10-0-UI | F-box only protein 11 | AI145603 |
| 2451 | UI-R-BT0-py-g-05-0-UI | XP_574033.1 Serine/threonine-protein kinase Kist (Kinase interacting with stathmin) (U2AF homology motif kinase 1) (PAM COOH-terminal interactor protein 2) (P-CIP2) | AI145543 |
| 2452 | UI-R-A0-ag-f-09-0-UI | Trafficking protein particle complex 3 | AA817958 |
| 2453 | UI-R-A0-ax-a-07-0-UI | Heat shock factor 2 | BF549866 |
| 2454 | UI-R-A0-bl-e-10-0-UI | Peroxiredoxin 4 | AA819406 |
| 2455 | UI-R-E0-br-d-06-0-UI | hypothetical protein FLJ10925 | AA859545 |
| 2456 | UI-R-E0-cw-a-04-0-UI | NADH dehydrogenase (ubiquinone) 1 alpha subcomplex, 6 (B14) | BF550168 |
| 2457 | UI-R-E0-dd-b-07-0-UI | Placental growth factor | AA900295 |
| 2458 | UI-R-E0-dk-b-01-0-UI | Dynactin 6 | AA900856 |
| 2459 | UI-R-A1-dp-g-01-0-UI | Cullin 3 | BF555537 |
| 2460 | UI-R-A1-ep-b-11-0-UI | Histidine triad nucleotide binding protein 3 | AA925490 |
| 2461 | UI-R-E1-fb-f-01-0-UI | XP_001088275.1 Kif19A CG9913-PB, isoform B isoform 1 | AA955984 |
| 2462 | UI-R-CA0-bhi-h-07-0-UI | Hypothetical LOC316091 | BF402904 |
| 2463 | UI-R-CW0-bvy-e-02-0-UI | Harvey rat sarcoma oncogene, subgroup R | BI273737 |
| 2464 | UI-R-CV2-chf-h-07-0-UI | Leo1, Paf1/RNA polymerase II complex component, homolog | BI298145 |
| 2465 | UI-R-E1-ga-d-12-0-UI | Transmembrane protein 98 | BF560441 |
| 2466 | UI-R-E1-gn-e-02-0-UI | Transcribed locus | AA963583 |
| 2467 | UI-R-C0-ia-g-02-0-UI | over-expressed breast tumor protein | AA998421 |
| 2468 | UI-R-C1-jt-b-10-0-UI | Transcribed locus | BF554104 |
| 2469 | UI-R-C2p-qv-g-11-0-UI | RGD, leucine-rich repeat, tropomodulin and proline-rich containing protein | BF546811 |
| 2470 | UI-R-C2p-rl-g-10-0-UI | LOC360555 | AI556770 |
| 2471 | UI-R-C0-ip-f-10-0-UI | RNA binding motif, single stranded interacting protein 2 | AI029139 |
| 2472 | UI-R-C0-iw-e-05-0-UI | XP_001101460.1 vacuolar protein sorting 41 (yeast homolog) | BF551610 |
| 2473 | UI-R-C1-kb-a-12-0-UI | Tumor necrosis factor receptor superfamily, member 17 | CK840697 |
| 2474 | UI-R-C1-kn-g-09-0-UI | Aristaless 3 | AI058446 |
| 2475 | UI-R-C1-lg-e-05-0-UI | XP_001103000.1 D-lactate dehydrogenase isoform 2 precursor | BF546611 |
| 2476 | UI-R-C1-ll-h-03-0-UI | Ribosomal protein, mitochondrial, L12 | BF552196 |
| 2477 | UI-R-E1-fv-g-03-0-UI | hypothetical protein MGC2574 | AA957879 |
| 2478 | UI-R-C0-ih-b-08-0-UI | RIKEN cDNA 5430437P03 | BF553999 |
| 2479 | UI-R-C1-jx-d-06-0-UI | expressed sequence AI597479 | AI044317 |
| 2480 | UI-R-C1-ln-g-07-0-UI | Protease, serine, 15 | AI070052 |
| 2481 | UI-R-C0-hc-d-08-0-UI | Chaperonin subunit 3 (gamma) | AA965201 |
| 2482 | UI-R-C0-ir-b-12-0-UI | Troponin T1, skeletal, slow | AI029355 |
| 2483 | UI-R-C0-jr-f-11-0-UI | Bicaudal D homolog 2 | CK840689 |
| 2484 | UI-R-C1-kv-e-10-0-UI | Annexin A7 | AI058585 |
| 2485 | UI-R-A1-eu-e-06-0-UI | Ankyrin repeat domain 13 family, member D | BF556937 |
| 2486 | UI-R-E1-fc-e-08-0-UI | Ras homolog gene family, member D | BF559374 |
| 2487 | UI-R-E1-fv-e-12-0-UI | General transcription factor II H, polypeptide 2 | AA957770 |
| 2488 | UI-R-E1-fy-e-09-0-UI | RAN binding protein 1 | BF561999 |
| 2489 | UI-R-C0-hl-c-11-0-UI | RNA polymerase II associated protein 1 | BF558121 |
| 2490 | UI-R-C0-il-g-08-0-UI | MEGF11 protein | BF551321 |
| 2491 | UI-R-E0-di-g-05-0-UI | ADP-ribosylation factor 6 | BF558595 |
| 2492 | UI-R-A1-ew-a-01-0-UI | Mannoside acetylglucosaminyltransferase 2 | AA955301 |
| 2493 | UI-R-C0-im-g-11-0-UI | Complement component 9 | BF561787 |
| 2494 | UI-R-C0-iv-c-05-0-UI | Ribosomal protein L41 | AI029242 |
| 2495 | UI-R-C0-ig-e-10-0-UI | Nuclear receptor subfamily 1, group D, member 1 | AA998969 |
| 2496 | UI-R-BT0-pz-f-09-0-UI | Transition protein 2 | AI146016 |
| 2497 | UI-R-C1-ko-e-03-0-UI | Tissue factor pathway inhibitor | AI071308 |
| 2498 | UI-R-C2-mr-f-02-0-UI | N-myristoyltransferase 1 | BF542263 |
| 2499 | UI-R-E0-bp-f-07-0-UI | Fractured callus expressed transcript 1 | BF550462 |
| 2500 | UI-R-E0-cu-h-04-0-UI | Nucleosome assembly protein 1-like 1 | AA899456 |
| 2501 | UI-R-C2-ne-h-10-0-UI | Protein-O-mannosyltransferase 1 | AI072418 |
| 2502 | UI-R-C2-ng-g-05-0-UI | RIKEN cDNA 2810451A06 | AI072361 |
| 2503 | UI-R-C2-nj-g-12-0-UI | Casein kinase 1, delta | BF553198 |
| 2504 | UI-R-C2-nl-e-09-0-UI | Dihydrouridine synthase 3-like | AI072198 |
| 2505 | UI-R-A0-ae-b-08-0-UI | Protein kinase C, zeta | AA817801 |
| 2506 | UI-R-A0-ap-f-07-0-UI | Basigin | AA819719 |
| 2507 | UI-R-A0-az-e-10-0-UI | Transthyretin | AA818637 |
| 2508 | UI-R-A0-bh-f-03-0-UI | Tumor protein, translationally-controlled 1 | AA900176 |
| 2509 | UI-R-E0-cp-b-10-0-UI | Benzodiazepine receptor, peripheral | AA875456 |
| 2510 | UI-R-E0-cz-a-09-0-UI | MYB binding protein (P160) 1a | BF555593 |
| 2511 | UI-R-C0-jg-a-10-0-UI | Serine/threonine kinase 2 | AI029638 |
| 2512 | UI-R-C0-jj-a-09-0-UI | RIKEN cDNA 2210010N04 gene | BF550724 |
| 2513 | UI-R-C1-kd-c-08-0-UI | XP_001099864.1 son of sevenless homolog 2 | AI044857 |
| 2514 | UI-R-C1-kf-d-08-0-UI | Interferon stimulated exonuclease 20 | AI045075 |
| 2515 | UI-R-C1-ki-f-12-0-UI | Protein phosphatase 1, regulatory (inhibitor) subunit 14c | CK840707 |
| 2516 | UI-R-C1-kk-c-07-0-UI | RIKEN cDNA 5730449L18 | AI045154 |
| 2517 | UI-R-C1-lb-b-04-0-UI | Hypothetical protein MGC19163 | BF558491 |
| 2518 | UI-R-C1-le-g-03-0-UI | RAE1 RNA export 1 homolog | BF549492 |
| 2519 | UI-R-C1-lo-c-02-0-UI | SET binding factor 1 | AI071923 |
| 2520 | UI-R-C1-lp-h-02-0-UI | mastermind-like 2 | AI070014 |
| 2521 | UI-R-A0-bf-d-02-0-UI | Vacuolar protein sorting 36 | AA858649 |
| 2522 | UI-R-E1-fa-e-03-0-UI | Heat shock protein | BF557349 |
| 2523 | UI-R-E1-fg-e-11-0-UI | Ng23 protein | AA956385 |
| 2524 | UI-R-E1-fl-b-01-0-UI | Angiopoietin 2 | BF556496 |
| 2525 | UI-R-C0-gx-e-01-0-UI | Podocalyxin-like | AA964715 |
| 2526 | UI-R-C0-hd-h-06-0-UI | Nexilin | AA996612 |
| 2527 | UI-R-C0-hi-d-03-0-UI | Muscle and microspikes RAS | AA996570 |
| 2528 | UI-R-C0-hu-d-10-0-UI | SH3 and multiple ankyrin repeat domains 1 | AA997864 |
| 2529 | UI-R-C0-it-d-04-0-UI | Praja 2, RING-H2 motif containing | BF551561 |
| 2530 | UI-R-C0-iy-g-01-0-UI | Dihydrouridine synthase 2-like, SMM1 homolog | BF552741 |
| 2531 | UI-R-C0-hu-b-03-0-UI | Ena-vasodilator stimulated phosphoprotein | AA997968 |
| 2532 | UI-R-C0-iz-b-05-0-UI | Protein tyrosine phosphatase, non-receptor type 21 | AI029768 |
| 2533 | UI-R-C1-kg-f-08-0-UI | Myh11 protein | AI045419 |
| 2534 | UI-R-C1-kn-g-11-0-UI | Coenzyme Q3 homolog, methyltransferase | AI058447 |
| 2535 | UI-R-C1-kw-g-05-0-UI | Purinergic receptor P2X-like 1, orphan receptor | AI059538 |
| 2536 | UI-R-C1-le-f-12-0-UI | Angiotensinogen (serpin peptidase inhibitor, clade A, member 8) | BF549490 |
| 2537 | UI-R-C2p-nu-c-11-0-UI | Interleukin 1 receptor, type II | BF553357 |
| 2538 | UI-R-C2p-oe-g-11-0-UI | Matrix metallopeptidase 13 | AI137141 |
| 2539 | UI-R-BT0-pn-g-09-0-UI | Regulator of G-protein signaling 8 | AI144709 |
| 2540 | UI-R-A0-ae-e-05-0-UI | Hemogen | BF548171 |
| 2541 | UI-R-CV2-cho-g-01-0-UI | Ubiquitin-conjugating enzyme E2I | BI298387 |
| 2542 | UI-R-DL0-cip-i-22-0-UI | Transcribed locus | BI302112 |
| 2543 | UI-R-DN0-ciu-g-13-0-UI | Regulated endocrine-specific protein 18 | BI291891 |
| 2544 | UI-R-DO0-ciw-j-12-0-UI | Beclin 1 (coiled-coil, myosin-like BCL2-interacting protein) | BI292341 |
| 2545 | UI-R-A1-dz-e-04-0-UI | Lysophospholipase 1 | AA924534 |
| 2546 | UI-R-A1-ec-f-01-0-UI | V-ral simian leukemia viral oncogene homolog B | AA925718 |
| 2547 | UI-R-A1-eg-c-02-0-UI | Ribosomal protein L6 | CK845765 |
| 2548 | UI-R-A1-ek-h-01-0-UI | Ribosomal protein S10 | BF521635 |
| 2549 | UI-R-C0-hh-f-05-0-UI | Interleukin 1 receptor-like 1 | BF561246 |
| 2550 | UI-R-C0-ho-d-06-0-UI | Lecithin cholesterol acyltransferase | BF557056 |
| 2551 | UI-R-CU0s-cbr-h-06-0-UI | XP_517029.2 stabilin 1 [Pan troglodytes] | BI279869 |
| 2552 | UI-R-CU0s-cby-c-01-0-UI | XP_001083784.1 solute carrier family 25 member 24 isoform 1 isoform 3 | BI282234 |
| 2553 | UI-R-DK0-cdg-e-11-0-UI | Hypothetical LOC287798 | BI288583 |
| 2554 | UI-R-DK0-cdt-e-08-0-UI | Secretory carrier membrane protein 5 | BI297117 |
| 2555 | UI-R-DK0-cec-d-06-0-UI | Myocyte enhancer factor 2D | BI294808 |
| 2556 | UI-R-DK0-cef-b-08-0-UI | Glutathione S-transferase, mu type 3 | BI295046 |
| 2557 | UI-R-CA0-blz-a-03-0-UI | Chymotrypsin-like | BF418064 |
| 2558 | UI-R-BJ2-bra-a-05-0-UI | Kelch repeat and BTB (POZ) domain containing 10 | BF408134 |
| 2559 | UI-R-BJ2-bre-e-02-0-UI | CAMP responsive element modulator | CK845198 |
| 2560 | UI-R-DK0-cfo-a-08-0-UI | Tyrosine hydroxylase | BI289772 |
| 2561 | UI-R-BJ2-bor-f-01-0-UI | EGF-like-domain, multiple 4 | BF414671 |
| 2562 | UI-R-BJ2-bph-d-03-0-UI | SEC14-like 1 | CK844571 |
| 2563 | UI-R-BJ2-bpp-a-07-0-UI | RAB24, member RAS oncogene family | CK845389 |
| 2564 | UI-R-BJ2-bpw-h-03-0-UI | HLA-B associated transcript 2 | BF420514 |
| 2565 | UI-R-CW0-bwo-a-06-0-UI | Angiotensin I converting enzyme (peptidyl-dipeptidase A) 1 | BI276266 |
| 2566 | UI-R-CX0-bxd-b-11-0-UI | Olfactory receptor 1280 | BI274717 |
| 2567 | UI-R-CX0-bxk-a-04-0-UI | Unc-93 homolog B1 | BI277821 |
| 2568 | UI-R-CY0-bxr-g-03-0-UI | Uncoupling protein 1 (mitochondrial, proton carrier) | BI278292 |
| 2569 | UI-R-DE0-cag-b-04-0-UI | hypothetical protein FLJ20272 | BI280283 |
| 2570 | UI-R-CT0s-cau-e-05-0-UI | CASK-interacting protein CIP98 | BI281619 |
| 2571 | UI-R-CA1-bhz-d-05-0-UI | Clone UI-R-FJ0-cqa-k-03-0-UI unknown mRNA | BF399826 |
| 2572 | UI-R-CA1-bip-a-07-0-UI | EGF, latrophilin and seven transmembrane domain containing 1 | BF405051 |
| 2573 | UI-R-CS0-bsd-e-05-0-UI | Potassium channel tetramerization domain containing 10 | BG373499 |
| 2574 | UI-R-CV1-bsp-f-07-0-UI | Cyclic nucleotide gated channel beta 1 | BG374460 |
| 2575 | UI-R-CS0-btj-b-05-0-UI | Mitochondrial ribosomal protein L2 | BG379697 |
| 2576 | UI-R-CT0-btw-f-05-0-UI | RS21-C6 protein | BG380390 |
| 2577 | UI-R-CV1-bvp-e-11-0-UI | Mitochondrial ribosomal protein S30 | BG378230 |
| 2578 | UI-R-CA0-bkh-e-01-0-UI | Vitamin A-deficient testicular protein 11-like | BF416040 |
| 2579 | UI-R-CN0-bkw-d-12-0-UI | Probasin | BF416988 |
| 2580 | UI-R-CN0-blg-b-05-0-UI | Leucine rich repeat containing 41 | BF417438 |
| 2581 | UI-R-BS1-ayw-b-02-0-UI | G1 to S phase transition 1 | BE108192 |
| 2582 | UI-R-BS1-aze-a-06-0-UI | Glypican 2 (cerebroglycan) | BE117625 |
| 2583 | UI-R-BJ1-azq-f-11-0-UI | Transcribed locus | CK842258 |
| 2584 | UI-R-CA0-bag-g-05-0-UI | Zinc finger, DHHC domain containing 13 | BE119597 |
| 2585 | UI-R-CA1-bco-f-12-0-UI | General transcription factor IIIC, polypeptide 2, beta 110kDa | BF390724 |
| 2586 | UI-R-BS2-bdd-d-07-0-UI | Homeodomain interacting protein kinase 2 | BF388602 |
| 2587 | UI-R-BS2-bdn-a-05-0-UI | XP_001134577.1 acetyl-CoA transporter [Dictyostelium discoideum AX4] | BF389636 |
| 2588 | UI-R-BS2-bdz-b-09-0-UI | Transcribed locus | BF396528 |
| 2589 | UI-R-CA0-bgw-b-07-0-UI | GRP1 (general receptor for phosphoinositides 1)-associated scaffold protein | BF393885 |
| 2590 | UI-R-CA0-bhg-g-01-0-UI | Zinc finger protein 384 | BF400864 |
| 2591 | UI-R-BT1-aqh-b-07-0-UI | Calsyntenin 3 | BE101768 |
| 2592 | UI-R-BT1-aqt-g-03-0-UI | RIM binding protein 2 | BE102826 |
| 2593 | UI-R-BJ1-atk-e-10-0-UI | Cyclin-dependent kinase 6 | BE099723 |
| 2594 | UI-R-BJ1-atq-b-05-0-UI | ubiquitin specific protease 46 | BE099777 |
| 2595 | UI-R-BJ1-atw-c-09-0-UI | Mesothelin | BE099462 |
| 2596 | UI-R-BJ1-auf-a-05-0-UI | Fas (TNFRSF6)-associated via death domain | CK842107 |
| 2597 | UI-R-BJ1-avx-h-08-0-UI | D4, zinc and double PHD fingers family 2 | BE111797 |
| 2598 | UI-R-BJ1-awc-c-04-0-UI | General transcription factor II H, polypeptide 1 | BE113163 |
| 2599 | UI-R-BJ1-awk-b-10-0-UI | NudC domain containing 1 | CK839967 |
| 2600 | UI-R-BJ1-awu-g-09-0-UI | Fragile histidine triad gene | BE115062 |
| 2601 | UI-R-BO1-aiy-b-08-0-UI | Amyloid beta (A4) precursor protein-binding, family B, member 1 | BF563064 |
| 2602 | UI-R-BO1-ajf-e-10-0-UI | RIKEN cDNA 5730469D23 | BF563153 |
| 2603 | UI-R-BO1-aji-h-12-0-UI | Transcribed locus | AW531522 |
| 2604 | UI-R-BO1-ajo-g-06-0-UI | IQ motif and WD repeats 1 | BF565683 |
| 2605 | UI-R-C4-aky-g-10-0-UI | NP_065704.1 finger protein 287 | BF563982 |
| 2606 | UI-R-C4-alg-g-04-0-UI | Microtubule associated serine/threonine kinase 2 | AW531105 |
| 2607 | UI-R-C4-alo-b-06-0-UI | Interleukin-1 receptor-associated kinase 2 | AW534333 |
| 2608 | UI-R-C4-alw-e-10-0-UI | Transgelin 2 | BF564545 |
| 2609 | UI-R-BU0-apg-e-12-0-UI | XP_001085737.1 Transcriptional activator protein Pur-alpha (Purine-rich single-stranded DNA-binding protein alpha) isoform 2 | BE096477 |
| 2610 | UI-R-BO1-apv-a-08-0-UI | hypothetical protein FLJ10233 | BE097245 |
| 2611 | UI-R-AA1-zy-b-08-0-UI | Heterogeneous nuclear ribonucleoprotein A1 | CK839186 |
| 2612 | UI-R-AG1-aal-d-05-0-UI | Etoposide induced 2.4 mRNA | CK838414 |
| 2613 | UI-R-Y0-ach-h-07-0-UI | Transcription factor 4 | AI716507 |
| 2614 | UI-R-Y0-acu-d-01-0-UI | Camello-like 5 | AI717047 |
| 2615 | UI-R-BJ0-adb-d-02-0-UI | Serine/threonine kinase 11 | AW251605 |
| 2616 | UI-R-BJ0-aeb-c-07-0-UI | KIAA1161 protein | AW252675 |
| 2617 | UI-R-BJ0p-age-a-10-0-UI | Protein phosphatase 2C, magnesium dependent, catalytic subunit | AW520836 |
| 2618 | UI-R-BO0-agl-c-12-0-UI | Endoplasmic reticulum chaperone SIL1 homolog | AW521335 |
| 2619 | UI-R-BO0-agv-f-06-0-UI | Chromodomain helicase DNA binding protein 6 | BF567314 |
| 2620 | UI-R-BO0-aho-a-10-0-UI | Ethylmalonic encephalopathy 1 | BF567869 |
| 2621 | UI-R-C3-sq-d-07-0-UI | Putative scaffolding protein POSH | AI535513 |
| 2622 | UI-R-C3-sz-b-10-0-UI | Dihydrouridine synthase 4-like | BF543477 |
| 2623 | UI-R-C3-tk-a-08-0-UI | RIKEN cDNA 2310047O13 | BF523288 |
| 2624 | UI-R-C3-tr-h-11-0-UI | Tripartite motif protein 50 | CK840325 |
| 2625 | UI-R-AB0-vs-h-04-0-UI | Myosin phosphatase-Rho interacting protein | AI577789 |
| 2626 | UI-R-AD0-wf-h-04-0-UI | Transient receptor potential cation channel, subfamily M, member 4 | CK842324 |
| 2627 | UI-R-AG0-wt-e-12-0-UI | Neural precursor cell expressed, developmentally down-regulated gene 4A | CK840452 |
| 2628 | UI-R-AG0-xc-d-01-0-UI | Ribosomal protein L13A | CK838652 |
| 2629 | UI-R-AE1-zj-c-02-0-UI | XP_001090451.1 acylphosphatase 1, erythrocyte (common) type | CK839120 |
| 2630 | UI-R-AD1-zp-c-11-0-UI | Solute carrier family 22 (organic cation transporter), member 17 | CK842377 |
| 2631 | UI-R-C0-hr-c-09-0-UI | RIKEN cDNA 1300010M03 | BF561400 |
| 2632 | UI-R-C0-id-g-01-0-UI | Lipase, gastric | AA998080 |
| 2633 | UI-R-Y0-ly-a-12-0-UI | Leucine rich repeat containing 21 | AI072952 |
| 2634 | UI-R-Y0-mo-f-03-0-UI | Arrestin 3, retinal | AI112106 |
| 2635 | UI-R-C2-nc-d-12-0-UI | Pantothenate kinase 4 | AI071709 |
| 2636 | UI-R-C2p-nw-c-12-0-UI | GLI-Kruppel family member HKR3 | AI136723 |
| 2637 | UI-R-BT0-qh-f-10-0-UI | Arx homeoprotein | AI146251 |
| 2638 | UI-R-C2p-qn-d-04-0-UI | XP_001083516.1 Histone H1.1 | BF547278 |
| 2639 | UI-R-C2p-qt-e-06-0-UI | Wingless-related MMTV integration site 6 | AI555162 |
| 2640 | UI-R-C2p-rc-e-04-0-UI | Mitofusin 1 | BF523002 |
| 2641 | UI-R-E0-bv-c-04-0-UI | Bol, boule-like | BF549922 |
| 2642 | UI-R-E0-bz-f-10-0-UI | Occludin | BF547858 |
| 2643 | UI-R-E0-ch-d-04-0-UI | tetracycline transporter-like protein | BF555062 |
| 2644 | UI-R-E0-cn-f-03-0-UI | Apolipoprotein M | AA875303 |
| 2645 | UI-R-A1-dv-b-08-0-UI | Solute carrier family 35, member E4 | BF555709 |
| 2646 | UI-R-A1-dy-g-01-0-UI | Mitogen activated protein kinase 1 | BF555997 |
| 2647 | UI-R-A1-ee-e-04-0-UI | Transcribed locus | AA925256 |
| 2648 | UI-R-A1-ej-a-07-0-UI | Bcl2-like 1 | BF544095 |
| 2649 | UI-R-E1-gq-h-04-0-UI | Complement component 1, r subcomponent | BF561182 |
| 2650 | UI-R-C0-hk-e-10-0-UI | hypothetical protein FLJ14803 | AA996958 |
| 2651 | UI-R-C2-mw-g-12-0-UI | DEAD (Asp-Glu-Ala-Asp) box polypeptide 59 | BF521750 |
| 2652 | UI-R-AG1-aal-e-05-0-UI | Intercellular adhesion molecule 2 | CK843340 |
| 2653 | UI-R-BT1-akr-g-11-0-UI | Nuclear receptor subfamily 6, group A, member 1 | BF563760 |
| 2654 | UI-R-CA0-bat-g-07-0-UI | LysM, putative peptidoglycan-binding, domain containing 3 | BE120551 |
| 2655 | UI-R-E0-ca-h-03-0-UI | zinc finger protein 740 | BF550016 |
| 2656 | UI-R-E0-df-g-08-0-UI | SGT1 protein homolog (Ecdysoneless homolog) | BF555446 |
| 2657 | UI-R-A1-dw-h-05-0-UI | Heterogeneous nuclear ribonucleoprotein L-like | BF555938 |
| 2658 | UI-R-E1-fc-g-10-0-UI | myocyte enhancer factor 2C | AA955670 |
| 2659 | UI-R-C2-mu-c-02-0-UI | Transcribed locus | AI072226 |
| 2660 | UI-R-C2p-oe-d-10-0-UI | Transmembrane emp24 protein transport domain containing 5 | AI137113 |
| 2661 | UI-R-C0-hc-c-05-0-UI | Interferon regulatory factor 7 | AA965186 |
| 2662 | UI-R-C0-hj-h-07-0-UI | Ring finger protein 8 | BF554058 |
| 2663 | UI-R-C0-je-h-03-0-UI | ARP10 actin-related protein 10 homolog | CK840672 |
| 2664 | UI-R-C0-jo-c-12-0-UI | Ubiquinol-cytochrome c reductase hinge protein | AI030694 |
| 2665 | UI-R-C1-jt-b-08-0-UI | Sh3 domain YSC-like 1 | AI045974 |
| 2666 | UI-R-C1-jz-e-11-0-UI | RNA-binding region (RNP1, RRM) containing 2 | AI045458 |
| 2667 | UI-R-E0-ch-g-05-0-UI | Cytochrome c, somatic | AA866442 |
| 2668 | UI-R-Y0-ls-d-12-0-UI | Mucin 5, subtypes A and C, tracheobronchial/gastric | AI070255 |
| 2669 | UI-R-A0-am-f-05-0-UI | Cytokine induced apoptosis inhibitor 1 | AA818105 |
| 2670 | UI-R-A1-ez-g-07-0-UI | Secretory carrier membrane protein 2 | AA955100 |
| 2671 | UI-R-E1-ga-h-08-0-UI | Solute carrier family 12, member 2 | AA963375 |
| 2672 | UI-R-A0-bg-g-11-0-UI | DEAD (Asp-Glu-Ala-Asp) box polypeptide 1 | AA866245 |
| 2673 | UI-R-A1-ev-c-07-0-UI | Spermatogenesis associated 6 | AA926274 |
| 2674 | UI-R-C0-gs-b-07-0-UI | Membrane bound C2 domain containing protein | AA963812 |
| 2675 | UI-R-A0-ay-c-06-0-UI | CDNA clone MGC:116225 IMAGE:7458604 | AA818770 |
| 2676 | UI-R-A0-bh-e-01-0-UI | Rho GTPase activating protein 18 | AA900163 |
| 2677 | UI-R-A0-ay-b-01-0-UI | Telomeric repeat binding factor 1 | AA818755 |
| 2678 | UI-R-A1-es-c-07-0-UI | XP_001096323.1 ATPase type 13A3 | BF558380 |
| 2679 | UI-R-C0-gt-e-07-0-UI | Sestrin 1 | AA964097 |
| 2680 | UI-R-C0-gz-c-08-0-UI | Processing of precursor 4, ribonuclease P/MRP family, | BF556700 |
| 2681 | UI-R-E0-ce-g-03-0-UI | Ras-related GTP binding A | BF555009 |
| 2682 | UI-R-E0-ck-f-11-0-UI | Adaptor-related protein complex 2, mu 1 subunit | AA874911 |
| 2683 | UI-R-E1-fg-f-06-0-UI | S100 protein, beta polypeptide | AA956175 |
| 2684 | UI-R-E1-ft-b-07-0-UI | Adenylate kinase 2 | AA956815 |
| 2685 | UI-R-E1-gd-a-04-0-UI | Carbonyl reductase 1 | AA962987 |
| 2686 | UI-R-C0-if-b-06-0-UI | Aminoadipate aminotransferase | BF561631 |
| 2687 | UI-R-E1-fc-f-01-0-UI | Interleukin 6 signal transducer | BF559375 |
| 2688 | UI-R-C0-gs-b-09-0-UI | N-myc downstream regulated gene 2 | AA963814 |
| 2689 | UI-R-C0-is-f-03-0-UI | Jun oncogene | BF554546 |
| 2690 | UI-R-C1-ju-c-02-0-UI | Hermansky-Pudlak syndrome 1 homolog | AI045279 |
| 2691 | UI-R-C2-mv-f-05-0-UI | XP_347075.2 hypothetical protein XP_347074 | BF549604 |
| 2692 | UI-R-C2-mz-g-09-0-UI | Transcription elongation regulator 1 (CA150) | BF552916 |
| 2693 | UI-R-C2-nb-g-08-0-UI | aquaporin 12 | AI071423 |
| 2694 | UI-R-C2-ne-a-12-0-UI | XP_001090986.1 60S ribosomal protein L22 (Heparin-binding protein HBp15) isoform 1 | AI072382 |
| 2695 | UI-R-C2-no-h-09-0-UI | NADH dehydrogenase (ubiquinone) 1 alpha subcomplex, 7 (B14.5a) | AI071086 |
| 2696 | UI-R-C2p-nu-e-06-0-UI | hypothetical protein FLJ20254 | BF553364 |
| 2697 | UI-R-C2p-nx-f-01-0-UI | WD repeat domain 61 | AI136056 |
| 2698 | UI-R-C2p-oa-h-09-0-UI | XP_001088942.1 intraflagellar transport 140 isoform 1 | AI136341 |
| 2699 | UI-R-E0-bt-b-09-0-UI | Vimentin | BF547727 |
| 2700 | UI-R-E0-by-e-03-0-UI | Endothelin converting enzyme 1 | AA900686 |
| 2701 | UI-R-C0-ii-e-09-0-UI | Frizzled homolog 5 | AA998744 |
| 2702 | UI-R-C0-ik-g-05-0-UI | Polymerase (RNA) II (DNA directed) polypeptide C | BF551107 |
| 2703 | UI-R-C0-jp-g-01-0-UI | Twist homolog 2 | BF550979 |
| 2704 | UI-R-C1-jx-f-03-0-UI | dendritic cell-derived ubiquitin-like protein | AI044323 |
| 2705 | UI-R-C1-jz-a-01-0-UI | Spermatogenesis associated 7 | BF550881 |
| 2706 | UI-R-C1-kb-h-05-0-UI | XP_238562.4 upstream binding protein 1 (LBP-1a) | AI044295 |
| 2707 | UI-R-C1-kq-g-02-0-UI | Cytidine and dCMP deaminase domain containing 1 | AI058883 |
| 2708 | UI-R-C1-ks-f-02-0-UI | Transcribed locus | AI058921 |
| 2709 | UI-R-C1-kx-b-04-0-UI | P-Rex1 | BF546357 |
| 2710 | UI-R-C1-kz-d-11-0-UI | Guanine nucleotide binding protein-like 2 (nucleolar) | AI060050 |
| 2711 | UI-R-A0-al-e-05-0-UI | Sulfotransferase family 2A, dehydroepiandrosterone (DHEA)-preferring, member 1 | AA819200 |
| 2712 | UI-R-A0-ap-h-05-0-UI | hypothetical protein FLJ11305 | AA819774 |
| 2713 | UI-R-A0-as-c-03-0-UI | Ribosomal protein L23 | BF548820 |
| 2714 | UI-R-A0-be-b-03-0-UI | CD276 antigen | BF559009 |
| 2715 | UI-R-E1-fo-f-10-0-UI | Der1-like domain family, member 2 | AA956743 |
| 2716 | UI-R-E1-fu-a-01-0-UI | Guanine nucleotide-binding protein beta subunit-like protein 1 (G protein beta subunit-like protein 1) (WD40 repeat-containing protein deleted in VCFS) (WDVCF protein) | BF556562 |
| 2717 | UI-R-E1-gp-c-05-0-UI | UDP-glucuronate decarboxylase 1 | BF561138 |
| 2718 | UI-R-C0-gw-b-08-0-UI | Phospholipase A2 receptor 1 | AA964065 |
| 2719 | UI-R-C0-ia-h-05-0-UI | Adrenergic receptor kinase, beta 1 | BF561652 |
| 2720 | UI-R-C0-id-d-01-0-UI | FXYD domain-containing ion transport regulator 5 | AA998066 |
| 2721 | UI-R-C0-gx-e-05-0-UI | CaM kinase-like vesicle-associated | BF557840 |
| 2722 | UI-R-C0-hd-g-10-0-UI | POU domain, class 3, transcription factor 3 | AA996605 |
| 2723 | UI-R-C0-jl-d-09-0-UI | Mal, T-cell differentiation protein 2 | BF550749 |
| 2724 | UI-R-C1-js-b-10-0-UI | Caveolin 3 | BF551397 |
| 2725 | UI-R-C1-jw-f-03-0-UI | Aminolevulinic acid synthase 2 | AI044581 |
| 2726 | UI-R-C1-ke-a-01-0-UI | C-terminal PDZ domain ligand of neuronal nitric oxide synthase | BF546462 |
| 2727 | UI-R-C1-lo-h-06-0-UI | Cortactin | BF552269 |
| 2728 | UI-R-C2-mu-b-02-0-UI | Adenosine A2B receptor | AI072220 |
| 2729 | UI-R-C2-nd-c-11-0-UI | Orthodenticle homolog 1 | AI072009 |
| 2730 | UI-R-C1-km-e-02-0-UI | Calponin 1 | AI045937 |
| 2731 | UI-R-DK0-cfy-c-06-0-UI | Protein kinase C, delta binding protein | BI290692 |
| 2732 | UI-R-DK0-cge-b-04-0-UI | Carcinoembryonic antigen-related cell adhesion molecule 3 | BI290993 |
| 2733 | UI-R-CV2-cgp-a-02-0-UI | Nuclear receptor coactivator 6 | BI291345 |
| 2734 | UI-R-CV2-chb-e-08-0-UI | transthyretin (4L369) | BI297863 |
| 2735 | UI-R-DR0-cjb-n-07-0-UI | Melanoma antigen, family D, 1 | BI303355 |
| 2736 | UI-R-A1-do-c-02-0-UI | Pregnancy-zone protein | BF558609 |
| 2737 | UI-R-A1-ds-h-01-0-UI | Ribosomal protein L22 | AA924274 |
| 2738 | UI-R-A1-dw-a-02-0-UI | Epidermal growth factor | BF558859 |
| 2739 | UI-R-E1-gj-e-10-0-UI | Opioid binding protein/cell adhesion molecule-like | AA963451 |
| 2740 | UI-R-C0-gt-e-11-0-UI | Nuclear receptor subfamily 1, group H, member 4 | BF557237 |
| 2741 | UI-R-DC0-bzj-d-09-0-UI | ATPase, H+ transporting, V0 subunit E isoform 1 | BI281384 |
| 2742 | UI-R-DD0-caa-b-10-0-UI | Putative GTP-binding protein | BI285445 |
| 2743 | UI-R-CW0s-cch-e-05-0-UI | RT1 class Ib, locus Aw2 | BI283769 |
| 2744 | UI-R-CX0s-ccq-f-03-0-UI | EH-domain containing 4 | BI284307 |
| 2745 | UI-R-CX0s-ccw-f-10-0-UI | Prolactin family 8, subfamily a, member 5 | BI287989 |
| 2746 | UI-R-DK0-cdd-g-03-0-UI | Scavenger receptor class B, member 2 | BI288684 |
| 2747 | UI-R-DK0-ceu-h-09-0-UI | ATPase type 13A1 | BI295729 |
| 2748 | UI-R-DK0-cfb-f-04-0-UI | Solute carrier family 39 (iron-regulated transporter), member 1 | BI295872 |
| 2749 | UI-R-DK0-cfg-a-05-0-UI | Eukaryotic translation initiation factor 2B, subunit 5 epsilon | BI289358 |
| 2750 | UI-R-CA1-bjt-a-07-0-UI | MICAL CG33208-PB, isoform B | BF410056 |
| 2751 | UI-R-BT1-bmn-h-06-0-UI | Urotensin 2 | BF411310 |
| 2752 | UI-R-BT1-bni-g-07-0-UI | Proteasome (prosome, macropain) 26S subunit, ATPase 3 | BG379124 |
| 2753 | UI-R-CA0-boc-f-09-0-UI | Chloride channel 7 | BF412947 |
| 2754 | UI-R-BJ2-boo-b-06-0-UI | Thymocyte nuclear protein 1 | CK845371 |
| 2755 | UI-R-BJ2-bqm-a-11-0-UI | Cytochrome P450 monooxygenase CYP2T1 | CK843737 |
| 2756 | UI-R-BJ2-bqq-b-10-0-UI | expressed sequence AA415817 | CK843794 |
| 2757 | UI-R-CW0-bvz-d-12-0-UI | mKIAA0804 protein | BI273829 |
| 2758 | UI-R-CW0-bwj-c-11-0-UI | Mitochondrial ribosomal protein S9 | BI274476 |
| 2759 | UI-R-CZ0-byi-h-02-0-UI | Nephroblastoma overexpressed gene | BI279072 |
| 2760 | UI-R-DB0-byu-b-02-0-UI | Zinc finger, FYVE domain containing 27 | BI284925 |
| 2761 | UI-R-CA0-bgd-d-10-0-UI | Alpha-spectrin 2 | BF393570 |
| 2762 | UI-R-CA0-bgp-d-08-0-UI | Gonadotropin-releasing hormone 1 | BF393045 |
| 2763 | UI-R-CA1-bjb-i-13-0-UI | 4921517L17Rik protein | BF399192 |
| 2764 | UI-R-CA1-bje-k-08-0-UI | Calcium channel, voltage-dependent, alpha 2/delta 3 subunit | BF405807 |
| 2765 | UI-R-CV0-brk-c-09-0-UI | Guanine nucleotide binding protein, beta 3 | BG371945 |
| 2766 | UI-R-CV1-brz-d-01-0-UI | Nuclear receptor subfamily 4, group A, member 1 | BG372684 |
| 2767 | UI-R-CT0-buk-c-03-0-UI | Proteasome (prosome, macropain) subunit, alpha type 6 | BG381517 |
| 2768 | UI-R-CU0-bus-c-11-0-UI | V-ets erythroblastosis virus E26 oncogene homolog 2 | BG376251 |
| 2769 | UI-R-CU0-bvc-b-05-0-UI | Hepatocellular carcinoma-associated antigen 58 homolog | BG377325 |
| 2770 | UI-R-CS0-bvm-a-05-0-UI | Open reading frame 19 | BG376997 |
| 2771 | UI-R-CA0-axf-c-10-0-UI | Hexokinase 2 | BE108528 |
| 2772 | UI-R-CA0-axq-a-11-0-UI | Down-regulator of transcription 1 | BE113500 |
| 2773 | UI-R-BS1-ayd-g-07-0-UI | Autocrine motility factor receptor | BE116658 |
| 2774 | UI-R-BS1-ays-g-05-0-UI | U6 snRNA-associated Sm-like protein LSm6 (Sm protein F) | BE107353 |
| 2775 | UI-R-CA0-baw-b-02-0-UI | Spermidine/spermine N1-acetyl transferase | BE121281 |
| 2776 | UI-R-CA1-bbf-f-03-0-UI | Hexokinase 3 | BF386623 |
| 2777 | UI-R-CA1-bbx-g-10-0-UI | G protein-coupled receptor 64 | BF388060 |
| 2778 | UI-R-CA1-bcg-b-02-0-UI | Protein tyrosine phosphatase, non-receptor type 23 | BF390363 |
| 2779 | UI-R-BS2-ber-e-04-0-UI | Transcribed locus |  |
| 2780 | UI-R-CA0-bfl-c-04-0-UI | Ras homolog gene family, member V | BF392615 |
| 2781 | UI-R-BS0-any-c-10-0-UI | Nuclear receptor subfamily 4, group A, member 2 | AW534944 |
| 2782 | UI-R-BU0-apd-c-11-0-UI | Tumor protein D52 | BE095930 |
| 2783 | UI-R-BX0-arx-g-12-0-UI | Active BCR-related gene | BE105687 |
| 2784 | UI-R-BO1-asl-a-12-0-UI | MAP/microtubule affinity-regulating kinase 1 | BE106192 |
| 2785 | UI-R-BJ1-asw-c-08-0-UI | hypothetical protein FLJ38426 | BE098052 |
| 2786 | UI-R-BJ1-atg-c-02-0-UI | Splicing factor 3b, subunit 2 | BE098910 |
| 2787 | UI-R-BJ1-aus-b-07-0-UI | Melanoma associated antigen (mutated) 1 | CK839769 |
| 2788 | UI-R-BJ1-avf-c-10-0-UI | Cytochrome P450 4F6 | BE110122 |
| 2789 | UI-R-BJ1-avp-d-06-0-UI | A kinase (PRKA) anchor protein 7 | BE109543 |
| 2790 | UI-R-BJ1-auv-a-12-0-UI | RIKEN cDNA 1500002O20 | BE111141 |
| 2791 | UI-R-BO0-aia-c-04-0-UI | Transcribed locus | AW524598 |
| 2792 | UI-R-BO0-aih-a-06-0-UI | POU domain, class 6, transcription factor 1 | AW523687 |
| 2793 | UI-R-BJ0p-aip-f-06-0-UI | Actin related protein 2/3 complex, subunit 4 | CK841928 |
| 2794 | UI-R-BJ0p-aiu-e-10-0-UI | CG9240-PA | AW525681 |
| 2795 | UI-R-BT1-ajx-h-05-0-UI | NCK interacting protein with SH3 domain | BF566063 |
| 2796 | UI-R-BT1-akg-c-07-0-UI | Ring finger (C3HC4 type) and KH domain containing 1 | AW527974 |
| 2797 | UI-R-BT1-akm-b-01-0-UI | Aquaporin 4 | BF565573 |
| 2798 | UI-R-BT1-aku-c-04-0-UI | Glutamate receptor, ionotropic, 4 | BF563855 |
| 2799 | UI-R-BU0-amv-a-04-0-UI | Zinc finger protein 213 | AW530299 |
| 2800 | UI-R-BU0-anc-g-08-0-UI | Tankyrase, TRF1-interacting ankyrin-related ADP-ribose polymerase | BF562351 |
| 2801 | UI-R-AB1-yu-d-07-0-UI | Ubiquitin-conjugating enzyme E2L 3 | CK840851 |
| 2802 | UI-R-AE1-zb-b-11-0-UI | Multimerin 2 | AI709829 |
| 2803 | UI-R-AF1-aay-a-03-0-UI | Biliverdin reductase A | AI714288 |
| 2804 | UI-R-Y0-abg-h-02-0-UI | Bone marrow stromal cell antigen 2 | AI716425 |
| 2805 | UI-R-Y0-abv-b-02-0-UI | Inositol hexaphosphate kinase 2 | BF523691 |
| 2806 | UI-R-Y0-acd-f-07-0-UI | Kinesin family member 13A | AI716069 |
| 2807 | UI-R-BJ0p-afd-c-06-0-UI | V-ets erythroblastosis virus E26 oncogene like | BF566705 |
| 2808 | UI-R-BJ0p-afl-d-06-0-UI | Wolfram syndrome 1 homolog | AW434371 |
| 2809 | UI-R-BJ0p-afq-f-08-0-UI | Myomesin 2 | BF566858 |
| 2810 | UI-R-BJ0p-aga-d-11-0-UI | RNA terminal phosphate cyclase-like 1 | BF566988 |
| 2811 | UI-R-C2p-rm-b-02-0-UI | Ras association (RalGDS/AF-6) domain family 4 | AI556545 |
| 2812 | UI-R-C2p-rv-d-10-0-UI | Baculoviral IAP repeat-containing 3 | AI500971 |
| 2813 | UI-R-C2p-sd-a-02-0-UI | Endosulfine alpha | AI501840 |
| 2814 | UI-R-C3-sm-d-09-0-UI | Poly(A) polymerase gamma | BF542666 |
| 2815 | UI-R-G0-uc-b-06-0-UI | Pleckstrin homology domain containing, family B (evectins) member 1 | AI574682 |
| 2816 | UI-R-G0-up-h-07-0-UI | RAB3A, member RAS oncogene family | BF525298 |
| 2817 | UI-R-Y0-uz-a-08-0-UI | Potassium inwardly-rectifying channel, subfamily J, member 14 | AI575918 |
| 2818 | UI-R-Y0-vj-c-10-0-UI | XP_001084706.1 KIAA0020 isoform 1 | AI577478 |
| 2819 | UI-R-AF0-yb-c-05-0-UI | eukaryotic translation initiation factor 1A, Y-linked | CK838807 |
| 2820 | UI-R-AC0-yl-g-02-0-UI | Caveolin | BF525123 |
| 2821 | UI-R-E1-gb-e-03-0-UI | Mitochondrial ribosomal protein L50 | AA957494 |
| 2822 | UI-R-E1-gj-g-06-0-UI | Protein kinase LYK5 | AA963467 |
| 2823 | UI-R-C0-jk-b-03-0-UI | Follistatin-like 3 | AI043879 |
| 2824 | UI-R-C1-kh-d-08-0-UI | Staufen, RNA binding protein, homolog 2 | BF554336 |
| 2825 | UI-R-C1-la-c-11-0-UI | receptor-interacting factor 1 | BF547147 |
| 2826 | UI-R-C1-lp-f-07-0-UI | Neurogranin | AI070181 |
| 2827 | UI-R-C2p-oj-e-04-0-UI | Phosphodiesterase 1A, calmodulin-dependent | AI137055 |
| 2828 | UI-R-BT0-pq-b-04-0-UI | RIKEN cDNA B930096L08 | AI145279 |
| 2829 | UI-R-BT0-pu-e-04-0-UI | testis specific gene A13 | BF544384 |
| 2830 | UI-R-BT0-qe-b-12-0-UI | CAP, adenylate cyclase-associated protein, 2 | BF545855 |
| 2831 | UI-R-A0-ao-d-11-0-UI | High density lipoprotein binding protein | AI454602 |
| 2832 | UI-R-A0-bc-a-01-0-UI | Apolipoprotein H | AA819305 |
| 2833 | UI-R-E0-bo-g-11-0-UI | Cdc2-related kinase, arginine/serine-rich | AA858456 |
| 2834 | UI-R-E0-bt-f-11-0-UI | General transcription factor III A | AA858535 |
| 2835 | UI-R-E0-cy-g-10-0-UI | Ribonuclease P 25 subunit | BF555589 |
| 2836 | UI-R-E0-dg-a-11-0-UI | Kinesin family member 5B | AA899705 |
| 2837 | UI-R-A1-do-d-04-0-UI | Fibroblast growth factor binding protein 1 | AA901105 |
| 2838 | UI-R-A1-ds-c-03-0-UI | Immunoglobulin superfamily, member 4B | BF558740 |
| 2839 | UI-R-A1-ev-h-11-0-UI | Aldehyde oxidase 1 | BF556976 |
| 2840 | UI-R-E1-fh-h-04-0-UI | Transcribed locus | AA956307 |
| 2841 | UI-R-CU0-bvi-g-04-0-UI | RIKEN cDNA 0610012D14 | BG378444 |
| 2842 | UI-R-CW0s-ccc-h-08-0-UI | Chemokine (C-C motif) ligand 21b (serine) | BI282920 |
| 2843 | UI-R-E1-gg-g-09-0-UI | 1110065L07Rik protein | AA963670 |
| 2844 | UI-R-C0-hn-h-08-0-UI | RIKEN cDNA 5830482F20 gene | AA998868 |
| 2845 | UI-R-C0-ir-h-12-0-UI | Cordon-bleu | BF554531 |
| 2846 | UI-R-C1-kv-g-12-0-UI | Leucine rich repeat neuronal 6A | BF547075 |
| 2847 | UI-R-C2p-re-a-02-0-UI | Vitelliform macular dystrophy 2-like protein 1 | BF546037 |
| 2848 | UI-R-C2p-rt-b-08-0-UI | 3632451O06Rik protein | AI501165 |
| 2849 | UI-R-C0-ir-f-11-0-UI | Phospholipid scramblase 3 | BF554520 |
| 2850 | UI-R-C0-ix-h-01-0-UI | es 64 | AI029340 |
| 2851 | UI-R-C1-km-b-06-0-UI | RNA binding motif protein 24 | AI045926 |
| 2852 | UI-R-C1-ku-f-06-0-UI | Abhydrolase domain containing 3 | AI071466 |
| 2853 | UI-R-C1-lj-g-01-0-UI | Cytoskeleton-associated protein 1 | AI059596 |
| 2854 | UI-R-C1-ln-e-12-0-UI | DNA segment, Chr 4, Brigham & Womens Genetics 0951 expressed | AI070045 |
| 2855 | UI-R-C0-hc-d-06-0-UI | BC002216 protein | BF557741 |
| 2856 | UI-R-C0-it-c-12-0-UI | Kruppel-like factor 2 (lung) | BF554562 |
| 2857 | UI-R-C1-kr-g-09-0-UI | XP_001100653.1 cryptochrome 1 (photolyase-like) | AI059856 |
| 2858 | UI-R-Y0-lu-a-02-0-UI | Glycerol-3-phosphate dehydrogenase 1 (soluble) | AI070199 |
| 2859 | UI-R-C0-ig-h-05-0-UI | Kruppel-like factor 15 | BF553990 |
| 2860 | UI-R-C0-jd-d-11-0-UI | Septin 5 | BF546915 |
| 2861 | UI-R-C1-ka-g-06-0-UI | Killer cell lectin-like receptor, family E, member 1 | AI045386 |
| 2862 | UI-R-C1-lr-b-11-0-UI | Erythrocyte protein band 4.1-like 3 | AI058943 |
| 2863 | UI-R-A1-ev-f-10-0-UI | Lactamase, beta | BF556966 |
| 2864 | UI-R-E1-fr-f-11-0-UI | XP_001118306.1 60S ribosomal protein L7 | BF562096 |
| 2865 | UI-R-E1-fx-a-01-0-UI | Membrane-spanning 4-domains, subfamily A, member 11 | AA963068 |
| 2866 | UI-R-E1-gj-g-03-0-UI | Sulfide:quinone oxidoreductase, mitochondrial precursor | AA963465 |
| 2867 | UI-R-C0-ih-e-04-0-UI | Topoisomerase I binding, arginine/serine-rich | AA998705 |
| 2868 | UI-R-C0-in-a-03-0-UI | TAR DNA binding protein | AA999071 |
| 2869 | UI-R-A1-es-f-03-0-UI | Cysteine and glycine-rich protein 3 | AA925907 |
| 2870 | UI-R-A1-ey-a-12-0-UI | Alpha-2-HS-glycoprotein | AA955349 |
| 2871 | UI-R-C0-io-h-08-0-UI | Neurogenic differentiation 3 | AI028971 |
| 2872 | UI-R-Y0-lu-a-01-0-UI | Crystallin, beta A4 | AI070198 |
| 2873 | UI-R-BT0-py-g-01-0-UI | Glutamate receptor, ionotropic, kainate 1 | AI145540 |
| 2874 | UI-R-A0-bi-g-08-0-UI | Protein kinase, lysine deficient 1 | AA858923 |
| 2875 | UI-R-C1-lm-d-11-0-UI | Cd36 antigen | AI060301 |
| 2876 | UI-R-A0-an-b-05-0-UI | Rat senescence marker protein 2A gene, exons 1 and 2 | AA819605 |
| 2877 | UI-R-E0-cd-c-03-0-UI | Ubiquitin-conjugating enzyme E2B, RAD6 homolog | AA859229 |
| 2878 | UI-R-A1-ev-h-06-0-UI | Major histocompatibility complex, class II, DM beta | BF560068 |
| 2879 | UI-R-C2-ng-b-12-0-UI | Transcription factor B2, mitochondrial | BF549816 |
| 2880 | UI-R-C2-ni-d-02-0-UI | RGD1566320 | AI072531 |
| 2881 | UI-R-C2-nk-c-01-0-UI | Heat shock 27kDa protein 3 | AI072433 |
| 2882 | UI-R-C2-nm-f-06-0-UI | Cbp/p300-interacting transactivator, with Glu/Asp-rich carboxy-terminal domain, 2 | AI111617 |
| 2883 | UI-R-A0-aj-g-05-0-UI | SPARC-like 1 (mast9, hevin) | AA866397 |
| 2884 | UI-R-A0-aw-c-11-0-UI | Guanylate cyclase 1, soluble, alpha 3 | AA819293 |
| 2885 | UI-R-A0-bd-c-01-0-UI | Thymoma viral proto-oncogene 1 | AA858883 |
| 2886 | UI-R-A0-bl-a-01-0-UI | Serum amyloid P-component | AA819477 |
| 2887 | UI-R-E0-ct-e-03-0-UI | Caspase 2 | AA875622 |
| 2888 | UI-R-E0-dd-e-11-0-UI | G protein-coupled receptor 116 | AA900213 |
| 2889 | UI-R-C0-jh-b-12-0-UI | Vaccinia related kinase 3 | AI030608 |
| 2890 | UI-R-C0-jl-b-11-0-UI | RIKEN cDNA 2510027N19 | CK840680 |
| 2891 | UI-R-C1-ke-b-08-0-UI | Golgi autoantigen, golgin subfamily b, macrogolgin 1 | AI044978 |
| 2892 | UI-R-C1-kg-h-06-0-UI | A kinase (PRKA) anchor protein 13 | BF554315 |
| 2893 | UI-R-C1-kj-e-05-0-UI | Meprin 1 alpha | BF560742 |
| 2894 | UI-R-C1-kl-a-06-0-UI | Asparaginyl-tRNA synthetase | AI045751 |
| 2895 | UI-R-C1-ld-b-03-0-UI | Proteasome (prosome, macropain) 26S subunit, non-ATPase, 8 | BF558505 |
| 2896 | UI-R-C1-lf-e-09-0-UI | Crystallin, zeta | BF549540 |
| 2897 | UI-R-C1-lp-c-10-0-UI | ATP-binding cassette, sub-family D (ALD), member 2 | AI070167 |
| 2898 | UI-R-Y0-lu-h-02-0-UI | Ubiquitin specific protease 19 | BF551908 |
| 2899 | UI-R-A1-ez-c-09-0-UI | Jumonji/ARID domain-containing protein 1C | AA955145 |
| 2900 | UI-R-E1-ff-f-09-0-UI | Zinc finger, CCHC domain containing 7 | AA955862 |
| 2901 | UI-R-E1-fk-c-12-0-UI | Staufen RNA binding protein homolog 1 | AA956589 |
| 2902 | UI-R-E1-fm-f-04-0-UI | Endomucin | BF556515 |
| 2903 | UI-R-C0-hd-a-08-0-UI | PHD finger protein 20-like 1 isoform 1 | AA965250 |
| 2904 | UI-R-C0-hh-d-10-0-UI | XP_001106227.1 WW domain-containing adapter with a coiled-coil region isoform 1 | AA996900 |
| 2905 | UI-R-C0-hj-d-03-0-UI | RIKEN cDNA 2410127L17 | AA996652 |
| 2906 | UI-R-C0-hv-f-10-0-UI | RGD1564450 | AA997819 |
| 2907 | UI-R-C0-iu-f-06-0-UI | Intraflagellar transport 80 homolog | BF551584 |
| 2908 | UI-R-C0-ja-b-10-0-UI | Small nuclear RNA activating complex, polypeptide 1 | AI029926 |
| 2909 | UI-R-C0-hw-b-01-0-UI | Squalene epoxidase | AA997541 |
| 2910 | UI-R-C0-jc-e-03-0-UI | Cystatin 8 | AI030491 |
| 2911 | UI-R-C1-kk-g-12-0-UI | Signal transducer and activator of transcription 3 | AI045179 |
| 2912 | UI-R-C1-kt-d-05-0-UI | CDP-diacylglycerol synthase 1 | BF550628 |
| 2913 | UI-R-C1-la-a-03-0-UI | Citron | BF558465 |
| 2914 | UI-R-C1-li-a-03-0-UI | Lipase, hormone sensitive | CK840731 |
| 2915 | UI-R-C2p-nz-a-12-0-UI | Delta-like 1 | AI136950 |
| 2916 | UI-R-C2p-om-a-10-0-UI | SRY-box containing gene 11 | AI138071 |
| 2917 | UI-R-A0-ac-c-05-0-UI | TTF-I interacting peptide 20; TIP20; Transcription Termination Factor I Interacting Peptide 20 | BF548659 |
| 2918 | UI-R-A0-ah-d-07-0-UI | XP_001111088.1 C10 protein isoform 3 | AA818015 |
| 2919 | UI-R-CV2-cie-h-10-0-UI | WNT1 inducible signaling pathway protein 1 | BI300434 |
| 2920 | UI-R-DN0-cit-f-22-0-UI | Vacuolar protein sorting 72 | BI291803 |
| 2921 | UI-R-DN0-civ-m-03-0-UI | Yip1 domain family, member 1 | BI292208 |
| 2922 | UI-R-DO0-cix-n-16-0-UI | Vacuolar protein sorting 35 | BI302711 |
| 2923 | UI-R-A1-ea-f-09-0-UI | Transcribed locus | AA924597 |
| 2924 | UI-R-A1-ee-c-01-0-UI | Complement component 1, q subcomponent, beta polypeptide | AA925356 |
| 2925 | UI-R-A1-ei-a-11-0-UI | Ribosomal protein L29 | AA925096 |
| 2926 | UI-R-A1-eo-a-09-0-UI | Branched chain keto acid dehydrogenase E1, beta polypeptide | AA925943 |
| 2927 | UI-R-C0-hl-d-05-0-UI | Prion protein | BF558124 |
| 2928 | UI-R-C0-hq-h-10-0-UI | Four and a half LIM domains 2 | AA997401 |
| 2929 | UI-R-CU0s-cbw-e-02-0-UI | XP_001111705.1 LIM homeobox protein 5 | BI282093 |
| 2930 | UI-R-CW0s-ccb-c-05-0-UI | Hemoglobin alpha, adult chain 1 | BI285534 |
| 2931 | UI-R-DK0-cdq-e-09-0-UI | Solute carrier family 4, sodium bicarbonate transporter-like, member 11 | BI293444 |
| 2932 | UI-R-DK0-cea-d-07-0-UI | Immature colon carcinoma transcript 1 | BI294246 |
| 2933 | UI-R-DK0-ced-g-02-0-UI | hypothetical protein BC011981 | BI294925 |
| 2934 | UI-R-DK0-ceg-b-02-0-UI | Rho GTPase activating protein 10 | BI295127 |
| 2935 | UI-R-BJ2-bqw-b-10-0-UI | Hairy/enhancer-of-split related with YRPW motif 1 | BF407145 |
| 2936 | UI-R-BJ2-brc-e-11-0-UI | Yamaguchi sarcoma viral (v-yes-1) oncogene homolog | BF408423 |
| 2937 | UI-R-DK0-cfh-h-10-0-UI | Chromatin modifying protein 6 | BI289511 |
| 2938 | UI-R-DK0-cfr-g-07-0-UI | SA rat hypertension-associated gene | BI290095 |
| 2939 | UI-R-BJ2-bow-c-01-0-UI | UDP-Gal:betaGlcNAc beta 1,3-galactosyltransferase, polypeptide 4 | CK843058 |
| 2940 | UI-R-BJ2-bpk-g-03-0-UI | Solute carrier family 2, (facilitated glucose transporter) member 8 | BF420257 |
| 2941 | UI-R-BJ2-bpr-h-03-0-UI | FYN binding protein | BF407121 |
| 2942 | UI-R-BJ2-bqg-c-08-0-UI | U2 small nuclear ribonucleoprotein auxiliary factor (U2AF) 2 | BF418480 |
| 2943 | UI-R-CX0-bww-a-07-0-UI | Glycoprotein 49b | BI275396 |
| 2944 | UI-R-CX0-bxi-b-06-0-UI | Cysteine-rich secretory protein LCCL domain containing 2 | BI275928 |
| 2945 | UI-R-CW0-bxo-d-12-0-UI | Casein kinase II, alpha 2, polypeptide | CK845509 |
| 2946 | UI-R-CY0-bxy-b-07-0-UI | Myeloid leukemia factor 2 | BI277208 |
| 2947 | UI-R-CT0s-cam-h-10-0-UI | Mitochondrial ribosomal protein L11 | BI286875 |
| 2948 | UI-R-CS0s-cbn-c-05-0-UI | Beta-globin | BI287300 |
| 2949 | UI-R-CA1-big-d-03-0-UI | Activity regulated cytoskeletal-associated protein | BF404552 |
| 2950 | UI-R-CA1-bix-b-04-0-UI | Solute carrier family 5 (sodium iodide symporter), member 5 | BF403644 |
| 2951 | UI-R-CV1-bsl-a-08-0-UI | Neurotrimin | BG373976 |
| 2952 | UI-R-CV1-bsz-d-07-0-UI | Interleukin-1 receptor-associated kinase 1 binding protein 1 | BG375351 |
| 2953 | UI-R-CS0-bts-f-05-0-UI | chromosome 1 open reading frame 2 | BG376030 |
| 2954 | UI-R-CT0-btz-d-09-0-UI | XP_001099518.1 ankyrin repeat containing protein RGD1359242 isoform 1 | BG380618 |
| 2955 | UI-R-CV1-bvu-g-02-0-UI | Arginyl aminopeptidase (aminopeptidase B) | BG378931 |
| 2956 | UI-R-BT1-bkn-h-01-0-UI | Parvin, alpha | BF409252 |
| 2957 | UI-R-CN0-blc-h-07-0-UI | BRF1 homolog, subunit of RNA polymerase III transcription initiation factor IIIB | BF417258 |
| 2958 | UI-R-CN0-blq-b-01-0-UI | Hematological and neurological expressed sequence 1 | BF417607 |
| 2959 | UI-R-BS1-aza-g-08-0-UI | Protein phosphatase 2, regulatory subunit B (B56), epsilon isoform | BE108994 |
| 2960 | UI-R-BS1-azm-c-11-0-UI | XP_001109504.1 hypothetical protein | BE117901 |
| 2961 | UI-R-CA0-azz-c-10-0-UI | Thioredoxin domain containing 13 | BE119170 |
| 2962 | UI-R-CA0-bao-e-04-0-UI | HCDI protein | BE120005 |
| 2963 | UI-R-CA1-bcw-g-08-0-UI | NP_660092.1 protein isoform e | BF391644 |
| 2964 | UI-R-BS2-bdh-h-02-0-UI | KIAA0980 protein | BF389101 |
| 2965 | UI-R-BS2-bdv-c-11-0-UI | SMC2 structural maintenance of chromosomes 2-like 1 | BF396223 |
| 2966 | UI-R-BS2-beb-g-06-0-UI | Homeo box B8 | BF397007 |
| 2967 | UI-R-CA0-bha-d-02-0-UI | Solute carrier family 1 (glial high affinity glutamate transporter), member 2 | BF394151 |
| 2968 | UI-R-CA0-bhp-a-09-0-UI | Notch gene homolog 2 | BF402421 |
| 2969 | UI-R-BT1-aqn-a-02-0-UI | DnaJ (Hsp40) homolog, subfamily C, member 6 | BE102465 |
| 2970 | UI-R-BX0-arc-e-01-0-UI | Nuclear receptor coactivator 1 | BE103470 |
| 2971 | UI-R-BJ1-ato-b-09-0-UI | Aminopeptidase puromycin sensitive | BE099326 |
| 2972 | UI-R-BJ1-ats-d-07-0-UI | Neuromedin U | BE100159 |
| 2973 | UI-R-BJ1-aua-a-11-0-UI | Farnesyl diphosphate farnesyl transferase 1 | BE101186 |
| 2974 | UI-R-BJ1-auh-f-01-0-UI | CPSF4 protein | BE101143 |
| 2975 | UI-R-BJ1-awa-f-06-0-UI | G-rich RNA sequence binding factor 1 | BE112943 |
| 2976 | UI-R-BJ1-awf-f-04-0-UI | HESB like domain containing 2 | BE113034 |
| 2977 | UI-R-BJ1-awp-d-03-0-UI | small nuclear RNA activating complex, polypeptide 5 | CK839998 |
| 2978 | UI-R-CA0-awy-b-12-0-UI | Transmembrane protein 35 | BE107908 |
| 2979 | UI-R-BO1-aja-g-09-0-UI | XP_001061644.1 Rad50-interacting protein 1 | AW526331 |
| 2980 | UI-R-BO1-ajh-b-04-0-UI | Transcribed locus |  |
| 2981 | UI-R-BO1-ajl-g-06-0-UI | Transcribed locus |  |
| 2982 | UI-R-BO1-ajs-d-04-0-UI | CWF19-like 2, cell cycle control | AW527267 |
| 2983 | UI-R-C4-alc-g-02-0-UI | Cytochrome P450, subfamily 3A, polypeptide 62 | BF564066 |
| 2984 | UI-R-C4-alk-e-02-0-UI | Transcribed locus |  |
| 2985 | UI-R-C4-alq-h-06-0-UI | hypothetical protein F730001J03 | AW534236 |
| 2986 | UI-R-BT1-ame-d-06-0-UI | Megakaryocyte-associated tyrosine kinase | BE107666 |
| 2987 | UI-R-BU0-apk-b-11-0-UI | Complexin 2 | BE096002 |
| 2988 | UI-R-BO1-aqb-f-02-0-UI | Activating signal cointegrator 1 complex subunit 3 | BE097776 |
| 2989 | UI-R-AA1-aac-c-04-0-UI | RT1 class Ib, locus Aw2 | AI706862 |
| 2990 | UI-R-AF1-aar-f-03-0-UI | CDNA sequence BC028440 | AI714021 |
| 2991 | UI-R-Y0-acn-a-01-0-UI | Serine (or cysteine) peptidase inhibitor, clade I, member 1 | AI763691 |
| 2992 | UI-R-Y0-acx-d-11-0-UI | Crystallin, gamma D | AI764316 |
| 2993 | UI-R-BJ0-ado-d-11-0-UI | Adenylate kinase 1 | AW251547 |
| 2994 | UI-R-BJ0-aej-h-08-0-UI | Translin | AW254570 |
| 2995 | UI-R-BJ0p-agi-b-03-0-UI | Spermatid perinuclear RNA binding protein | CK841406 |
| 2996 | UI-R-BO0-agq-e-08-0-UI | Cell growth regulator with ring finger domain 1 | BF567570 |
| 2997 | UI-R-BO0-ahk-d-12-0-UI | Beta-1,4-N-acetyl-galactosaminyl transferase 1 | BF567749 |
| 2998 | UI-R-BO0-aht-a-08-0-UI | Sterile alpha motif domain containing 4B | AW523246 |
| 2999 | UI-R-C3-sv-b-03-0-UI | Natural cytotoxicity triggering receptor 3 | AI511264 |
| 3000 | UI-R-C3-th-h-05-0-UI | C-type lectin domain family 4, member f | AI547716 |
| 3001 | UI-R-C3-tm-g-09-0-UI | hypothetical protein MGC4189 | BF524474 |
| 3002 | UI-R-C3-tv-a-09-0-UI | Cationic trypsinogen | BF522564 |
| 3003 | UI-R-AD0-vz-d-12-0-UI | Small nuclear ribonucleoprotein polypeptide A | CK838543 |
| 3004 | UI-R-AA0-wl-f-05-0-UI | FIP1 like 1 | AI578557 |
| 3005 | UI-R-AG0-wx-f-05-0-UI | RIKEN cDNA D030012E24 gene | AI579771 |
| 3006 | UI-R-AE0-xl-e-06-0-UI | RIKEN cDNA D930015E06 | BF524632 |
| 3007 | UI-R-AD1-zn-c-04-0-UI | Contactin 1 | CK839690 |
| 3008 | UI-R-AD1-zs-a-04-0-UI | Bystin-like | AI713339 |
| 3009 | UI-R-C0-ht-e-09-0-UI | Nuclear factor of kappa light polypeptide gene enhancer in B-cells inhibitor-like 1 | AA997909 |
| 3010 | UI-R-C0-in-a-09-0-UI | Potassium channel, subfamily K, member 2 | BF551345 |
| 3011 | UI-R-Y0-mj-a-06-0-UI | Adrenergic receptor, alpha 2c | AI112735 |
| 3012 | UI-R-C2-mu-f-08-0-UI | Cyclin-dependent kinase inhibitor 1A | AI385366 |
| 3013 | UI-R-C2p-nr-d-08-0-UI | Solute carrier family 39 (zinc transporter), member 10 | BF522184 |
| 3014 | UI-R-C2p-oc-f-09-0-UI | Nuclear receptor subfamily 3, group C, member 1 | AI137538 |
| 3015 | UI-R-BT0-qk-c-05-0-UI | Calcium channel, voltage-dependent, L type, alpha 1S subunit | AI454679 |
| 3016 | UI-R-C2p-qq-c-06-0-UI | Cytochrome P450, subfamily 3A, polypeptide 3 | AI555291 |
| 3017 | UI-R-C2p-qu-h-03-0-UI | Lmo6 protein | AI555009 |
| 3018 | UI-R-C2p-rf-e-09-0-UI | Dynein light chain roadblock-type 2 | AI556261 |
| 3019 | UI-R-E0-bx-b-11-0-UI | Ubiquitin specific protease 48 | BF547815 |
| 3020 | UI-R-E0-cc-e-06-0-UI | Transgelin | AA859813 |
| 3021 | UI-R-E0-ck-e-04-0-UI | chromosome 10 open reading frame 4 | AA874896 |
| 3022 | UI-R-E0-cs-e-06-0-UI | Cyclin-dependent kinase inhibitor 3 | AI502179 |
| 3023 | UI-R-A1-dw-h-07-0-UI | Apolipoprotein B editing complex 2 | BF555940 |
| 3024 | UI-R-A1-eb-g-12-0-UI | UDP-N-acetyl-alpha-D-galactosamine:polypeptide N-acetylgalactosaminyltransferase 11 (GalNAc-T11) | BF556095 |
| 3025 | UI-R-A1-eh-a-11-0-UI | Cell death-inducing DNA fragmentation factor, alpha subunit-like effector A | AA924977 |
| 3026 | UI-R-A1-el-c-08-0-UI | Mannan-binding lectin serine peptidase 1 | AA926053 |
| 3027 | UI-R-C0-gz-h-11-0-UI | Procollagen, type VI, alpha 2 | AA965241 |
| 3028 | UI-R-C0-ho-e-06-0-UI | solute carrier family 30 (zinc transporter), member 9 | AA997326 |
| 3029 | UI-R-C2p-nw-a-04-0-UI | Adaptor-related protein complex 1, gamma 1 subunit | AI136545 |
| 3030 | UI-R-C3-ta-h-04-0-UI | A disintegrin and metallopeptidase domain 23 | AI535213 |
| 3031 | UI-R-BO0-ahl-b-12-0-UI | Cytochrome b5 domain containing 2 | BF567776 |
| 3032 | UI-R-BJ1-aux-b-05-0-UI | Fascin homolog 3, actin-bundling protein, testicular | CK839859 |
| 3033 | UI-R-A0-bb-a-09-0-UI | Transcribed locus | AI501773 |
| 3034 | UI-R-E0-cj-c-05-0-UI | RIKEN cDNA 2010110K16 | AA859311 |
| 3035 | UI-R-A1-dp-e-07-0-UI | Zinc finger protein 692 | BF555530 |
| 3036 | UI-R-A1-ek-h-06-0-UI | Rab40b, member RAS oncogene family | AA925328 |
| 3037 | UI-R-C1-lm-d-10-0-UI | CG18661-PA | AI060300 |
| 3038 | UI-R-C2p-nr-g-03-0-UI | Transcribed locus | AI113133 |
| 3039 | UI-R-C0-ha-g-03-0-UI | Nuclear prelamin A recognition factor | BF556761 |
| 3040 | UI-R-C0-hg-b-02-0-UI | Protein disulfide isomerase associated 2 | BF561190 |
| 3041 | UI-R-C0-jd-a-08-0-UI | ADP-ribosylation factor-like 12 | AI030641 |
| 3042 | UI-R-C0-jm-e-12-0-UI | WD repeat domain 45 | BF550580 |
| 3043 | UI-R-C0-jr-b-11-0-UI | Hypothetical LOC290577 | AI029970 |
| 3044 | UI-R-C1-ju-e-02-0-UI | NADH dehydrogenase (ubiquinone) Fe-S protein 2 | AI045289 |
| 3045 | UI-R-A0-au-d-10-0-UI | Tachykinin 1 | AA818532 |
| 3046 | UI-R-E1-fd-b-04-0-UI | Phosphatidylinositol transfer protein, beta | AA955903 |
| 3047 | UI-R-C2-nc-e-02-0-UI | MAS1 oncogene | AI071498 |
| 3048 | UI-R-A0-aw-d-01-0-UI | Transmembrane and coiled-coil domains 1 | AA819295 |
| 3049 | UI-R-E1-fi-e-05-0-UI | Type II keratin Kb1 | BF556447 |
| 3050 | UI-R-C0-ix-f-09-0-UI | Vesicle-associated membrane protein 1 | CK840226 |
| 3051 | UI-R-A0-ba-e-12-0-UI | Putative ISG12(b) protein | AA819034 |
| 3052 | UI-R-E1-fw-e-07-0-UI | Src family associated phosphoprotein 2 | AA957679 |
| 3053 | UI-R-C2-mr-a-09-0-UI | Protein phosphatase 1B, magnesium dependent, beta isoform | CK840243 |
| 3054 | UI-R-E1-fv-f-08-0-UI | Nuclear transport factor 2 | AA957777 |
| 3055 | UI-R-A0-aw-e-05-0-UI | Ceroid-lipofuscinosis, neuronal 8 | AA818537 |
| 3056 | UI-R-A0-az-g-01-0-UI | ankyrin repeat domain protein 17 isoform b | AA818652 |
| 3057 | UI-R-E1-gl-a-04-0-UI | Tropomodulin 3 | BF557530 |
| 3058 | UI-R-C0-gv-d-09-0-UI | COP9 (constitutive photomorphogenic) homolog, subunit 3 | BF557307 |
| 3059 | UI-R-E0-cb-e-01-0-UI | Tumor protein p53 | BF554812 |
| 3060 | UI-R-E0-ch-b-06-0-UI | Retinoic acid receptor, alpha | AA866415 |
| 3061 | UI-R-E1-fb-b-05-0-UI | Farnesyltransferase, CAAX box, beta | AA955617 |
| 3062 | UI-R-E1-fn-a-08-0-UI | ST6 (alpha-N-acetyl-neuraminyl-2,3-beta-galactosyl-1,3)-N-acetylgalactosaminide alpha-2,6-sialyltransferase 3 | AA956521 |
| 3063 | UI-R-E1-fw-d-02-0-UI | Tetraspanin 8 | AA957483 |
| 3064 | UI-R-C0-hz-d-02-0-UI | Purinergic receptor P2X, ligand-gated ion channel 4 | AA998365 |
| 3065 | UI-R-A0-ba-g-08-0-UI | Ribosomal protein S16 | BF558971 |
| 3066 | UI-R-E1-fy-b-07-0-UI | YY1 transcription factor | AA957358 |
| 3067 | UI-R-C0-he-h-07-0-UI | Phospholipase C, delta 1 | AA996438 |
| 3068 | UI-R-C0-jd-f-08-0-UI | B-cell CLL/lymphoma 10 | BF546929 |
| 3069 | UI-R-Y0-lv-g-05-0-UI | Erythrocyte protein band 4.1-like 4a | AI070356 |
| 3070 | UI-R-C2-mw-d-01-0-UI | Engulfment and cell motility 3, ced-12 homolog | AI071381 |
| 3071 | UI-R-C2-na-g-08-0-UI | Polymerase (DNA directed), gamma | AI070998 |
| 3072 | UI-R-C2-nd-a-11-0-UI | Neuronal PAS domain protein 3 | BF549624 |
| 3073 | UI-R-C2-nn-f-12-0-UI | Polypyrimidine tract binding protein 2 | AI072635 |
| 3074 | UI-R-C2p-nq-h-06-0-UI | Claudin 19 | BF521861 |
| 3075 | UI-R-C2p-nw-b-09-0-UI | NADH dehydrogenase (ubiquinone) 1 alpha subcomplex, 2 | BF553588 |
| 3076 | UI-R-C2p-ny-d-11-0-UI | TBC1 domain family, member 10b | BF522229 |
| 3077 | UI-R-E0-bo-a-08-0-UI | Ribosomal protein S2 | AA858477 |
| 3078 | UI-R-E0-bv-d-06-0-UI | Calcineurin binding protein 1 | AA859586 |
| 3079 | UI-R-C0-if-a-11-0-UI | XP_001106271.1 endosulfine alpha isoform 6 | BF561630 |
| 3080 | UI-R-C0-ij-f-12-0-UI | Rho GTPase activating protein 4 | AI028822 |
| 3081 | UI-R-C0-jp-a-12-0-UI | Ssu72 RNA polymerase II CTD phosphatase homolog | AI031026 |
| 3082 | UI-R-C1-jw-g-06-0-UI | T-box 3 | AI044589 |
| 3083 | UI-R-C1-jy-b-06-0-UI | RAB25, member RAS oncogene family | AI058820 |
| 3084 | UI-R-C1-jz-d-06-0-UI | tripartite motif-containing 62 | AI045540 |
| 3085 | UI-R-C1-kq-a-04-0-UI | Centromere protein J | AI058854 |
| 3086 | UI-R-C1-kr-e-03-0-UI | Zic family member 4 | BF554431 |
| 3087 | UI-R-C1-kw-b-06-0-UI | XP_346966.2 hypothetical protein XP_346965 | BF547082 |
| 3088 | UI-R-C1-ky-b-09-0-UI | COX11 homolog, cytochrome c oxidase assembly protein | AI071535 |
| 3089 | UI-R-A0-aj-d-05-0-UI | Mitochondrial ribosomal protein L34 | AA858969 |
| 3090 | UI-R-A0-am-b-11-0-UI | Alcohol dehydrogenase, iron containing, 1 | AA818154 |
| 3091 | UI-R-A0-aq-f-05-0-UI | Transcribed locus | AA819795 |
| 3092 | UI-R-A0-au-g-07-0-UI | Syndecan binding protein | AA818429 |
| 3093 | UI-R-E1-fn-d-06-0-UI | Inner membrane protein, mitochondrial | AA956621 |
| 3094 | UI-R-E1-fq-d-08-0-UI | Carbonic anhydrase 4 | AA957002 |
| 3095 | UI-R-E1-fu-h-01-0-UI | Lymphoblastic leukemia derived sequence 1 | AA957333 |
| 3096 | UI-R-E1-gq-h-01-0-UI | Blocked early in transport 1 homolog like | BF561181 |
| 3097 | UI-R-C0-hz-b-09-0-UI | microrchidia | BF557660 |
| 3098 | UI-R-C0-ic-a-10-0-UI | Integrin beta 1 binding protein 1 | BF561709 |
| 3099 | UI-R-C0-gv-h-02-0-UI | ADP-ribosylation factor 2 | AA964530 |
| 3100 | UI-R-C0-ha-a-01-0-UI | Glucose-6-phosphatase, catalytic | BF556723 |
| 3101 | UI-R-C0-jg-e-05-0-UI | Hexokinase 1 | BF545064 |
| 3102 | UI-R-C0-jo-a-12-0-UI | Nestin | BF554673 |
| 3103 | UI-R-C1-ju-h-01-0-UI | Coronin 7 | BF551428 |
| 3104 | UI-R-C1-kb-e-11-0-UI | Glutathione S-transferase theta 1 | AI044236 |
| 3105 | UI-R-C1-lk-g-02-0-UI | Aldehyde dehydrogenase family 1, subfamily A2 | BF546771 |
| 3106 | UI-R-Y0-ls-c-04-0-UI | Cathepsin D | AI070076 |
| 3107 | UI-R-C2-mz-g-05-0-UI | Max dimerization protein 3 | AI071912 |
| 3108 | UI-R-C2-nh-a-06-0-UI | Rab geranylgeranyl transferase, a subunit | BF549663 |
| 3109 | UI-R-DK0-cfv-d-04-0-UI | Isochorismatase domain containing 1 | BI290362 |
| 3110 | UI-R-DK0-cga-c-06-0-UI | Signal recognition particle 54 | BI290777 |
| 3111 | UI-R-DK0-cgi-h-10-0-UI | Hypothetical LOC287173 | BI290905 |
| 3112 | UI-R-CV2-cgt-c-03-0-UI | Glutamate-ammonia ligase (glutamine synthase) | BI296610 |
| 3113 | UI-R-DQ0-cja-e-17-0-UI | hypothetical protein MGC40841; hypothetical protein MGC4707 | BI303387 |
| 3114 | UI-R-E0-dk-e-04-0-UI | Junction plakoglobin | BF556619 |
| 3115 | UI-R-A1-dq-g-02-0-UI | Apolipoprotein E | AA923989 |
| 3116 | UI-R-A1-du-d-05-0-UI | Aquaporin 1 | AA955200 |
| 3117 | UI-R-E1-gf-h-12-0-UI | Telomerase associated protein 1 | AA957581 |
| 3118 | UI-R-E1-go-d-04-0-UI | Zinc finger protein 265 | BF557090 |
| 3119 | UI-R-DB0-byy-a-02-0-UI | XP_001098453.1 DEAD (Asp-Glu-Ala-Asp) box polypeptide 55 | BI285256 |
| 3120 | UI-R-DD0-bzt-h-07-0-UI | Regulator of G-protein signaling 4 | BI286121 |
| 3121 | UI-R-CW0s-ccd-c-03-0-UI | Uroporphyrinogen decarboxylase | BI282626 |
| 3122 | UI-R-CX0s-cco-a-01-0-UI | Ribosomal protein L3 | BI284335 |
| 3123 | UI-R-CX0s-ccs-d-02-0-UI | Guanine nucleotide binding protein beta 4 subunit | BI284800 |
| 3124 | UI-R-DK0-cdb-a-03-0-UI | RAD23a homolog | BI288445 |
| 3125 | UI-R-DK0-cel-f-05-0-UI | Interferon regulatory factor 2 | BI294557 |
| 3126 | UI-R-DK0-cey-d-02-0-UI | ASF1 anti-silencing function 1 homolog A | BI296271 |
| 3127 | UI-R-DK0-cfe-a-04-0-UI | Stonin 2 | BI289055 |
| 3128 | UI-R-CM0-bji-h-08-0-UI | CD74 antigen (invariant polypeptide of major histocompatibility complex, class II antigen-associated) | BF395143 |
| 3129 | UI-R-CN0-blx-d-03-0-UI | RIKEN cDNA 1810047C23 | BF417800 |
| 3130 | UI-R-BT1-bmu-e-01-0-UI | CD3 antigen, epsilon polypeptide | BF412154 |
| 3131 | UI-R-BT1-bnq-a-05-0-UI | SV2 related protein | BF412623 |
| 3132 | UI-R-CA0-boi-c-08-0-UI | hypothetical protein 4930503F14 | BF413765 |
| 3133 | UI-R-BJ2-bqi-a-01-0-UI | Glucose phosphate isomerase | BF418615 |
| 3134 | UI-R-BJ2-bqo-e-06-0-UI | Processing of precursor 5, ribonuclease P/MRP family | BF419136 |
| 3135 | UI-R-CW0-bvv-d-11-0-UI | DiGeorge syndrome critical region gene 6 | BG371463 |
| 3136 | UI-R-CW0-bwf-f-06-0-UI | Vasodilator-stimulated phosphoprotein | BI274155 |
| 3137 | UI-R-CZ0-byf-e-12-0-UI | Keratin complex 2, basic, gene 5 | BI277713 |
| 3138 | UI-R-DA0-bym-e-04-0-UI | Cell growth regulator with EF hand domain 1 | BI279285 |
| 3139 | UI-R-CA0-bfu-e-03-0-UI | Amyloid beta (A4) precursor protein-binding, family A, member 2 | BF393487 |
| 3140 | UI-R-CA0-bgh-e-08-0-UI | hypothetical protein FLJ33868 | BF401093 |
| 3141 | UI-R-CA1-biy-e-16-0-UI | Hypothetical protein LOC685087 | BF403325 |
| 3142 | UI-R-CA1-bjd-d-23-0-UI | Heme binding protein 1 | BF403904 |
| 3143 | UI-R-CA1-bjf-i-03-0-UI | Ectonucleotide pyrophosphatase/phosphodiesterase 2 | BF406159 |
| 3144 | UI-R-CV1-brv-d-09-0-UI | XP_001084658.1 60S ribosomal protein L23a | BG372545 |
| 3145 | UI-R-CT0-bud-c-08-0-UI | Amyloid beta (A4) precursor protein | BG381608 |
| 3146 | UI-R-CT0-bun-c-03-0-UI | Sodium channel modifier 1 | BG376162 |
| 3147 | UI-R-CU0-buv-a-12-0-UI | Solute carrier family 7 (cationic amino acid transporter, y+ system), member 7 | BG376713 |
| 3148 | UI-R-CU0-bvh-a-05-0-UI | Interferon induced transmembrane protein 2 (1-8D) | BG377943 |
| 3149 | UI-R-CA0-axd-a-07-0-UI | Secretagogin, EF-hand calcium binding protein | BE112808 |
| 3150 | UI-R-CA0-axi-f-07-0-UI | kelch domain containing 2 | BE109897 |
| 3151 | UI-R-BS1-axx-b-12-0-UI | FK506 binding protein 12-rapamycin associated protein 1 | BE115861 |
| 3152 | UI-R-BS1-ayn-h-05-0-UI | Nipped-B homolog | BE117361 |
| 3153 | UI-R-CA0-bap-h-11-0-UI | Transcribed locus | BE120220 |
| 3154 | UI-R-CA0-bay-f-08-0-UI | Putative regulation protein GS3 | BE120711 |
| 3155 | UI-R-CA1-bbn-c-10-0-UI | Solute carrier family 6 (neurotransmitter transporter), member 15 | BF387518 |
| 3156 | UI-R-CA1-bcb-f-03-0-UI | Potassium voltage-gated channel, subfamily H (eag-related), member 4 | BF396125 |
| 3157 | UI-R-BS2-beh-c-06-0-UI | GLI-Kruppel family member GLI3 | BF397040 |
| 3158 | UI-R-BS2-bex-d-04-0-UI | chromodomain helicase DNA binding protein 9 | BF397354 |
| 3159 | UI-R-BU0-anf-e-04-0-UI | Fatty acid desaturase 3 | AW533321 |
| 3160 | UI-R-BU0-aoz-a-09-0-UI | Carnitine palmitoyltransferase 1c | BE095750 |
| 3161 | UI-R-BX0-arl-b-03-0-UI | S-adenosylmethionine decarboxylase 1 | BE104130 |
| 3162 | UI-R-BX0-asg-g-08-0-UI | Stathmin 1 | BE105129 |
| 3163 | UI-R-BO1-asq-a-08-0-UI | Cd200 antigen | BE106615 |
| 3164 | UI-R-BJ1-asz-g-04-0-UI | Transcribed locus |  |
| 3165 | UI-R-BJ1-aun-a-03-0-UI | Transcription factor 21 | BE110350 |
| 3166 | UI-R-BJ1-ava-b-11-0-UI | Calpain 2 | BE111301 |
| 3167 | UI-R-BJ1-avm-b-04-0-UI | Cdc42 binding protein kinase beta | BE112391 |
| 3168 | UI-R-BJ1-avr-c-09-0-UI | Tight junction associated protein 1 | CK844141 |
| 3169 | UI-R-BO0-ahx-f-07-0-UI | ACN9 homolog | AW524458 |
| 3170 | UI-R-BO0-aid-f-12-0-UI | HECT domain and ankyrin repeat containing, E3 ubiquitin protein ligase 1 | BF562740 |
| 3171 | UI-R-BJ0p-aik-e-01-0-UI | Zinc finger and BTB domain containing 9 | CK843950 |
| 3172 | UI-R-BJ0p-air-e-09-0-UI | Methionine sulfoxide reductase A | CK841965 |
| 3173 | UI-R-BO1-aju-e-07-0-UI | Heat shock protein 70kDa 12B | BF565341 |
| 3174 | UI-R-BT1-akb-h-03-0-UI | Transient receptor potential cation channel, subfamily V, member 2 | AW527937 |
| 3175 | UI-R-BT1-akj-b-08-0-UI | Calcium/calmodulin-dependent protein kinase IV | BF563485 |
| 3176 | UI-R-BT1-akp-f-12-0-UI | ZW10 interactor | AW530572 |
| 3177 | UI-R-BS0-amq-c-09-0-UI | Protein arginine N-methyltransferase 4 (Heterogeneous nuclear ribonucleoprotein methyltransferase-like protein 4) | BF564993 |
| 3178 | UI-R-BU0-amz-f-11-0-UI | Lin-7 homolog b | BF564887 |
| 3179 | UI-R-AC0-yp-c-12-0-UI | Syntaxin 4A (placental) | AI704544 |
| 3180 | UI-R-AB1-yy-c-09-0-UI | Ca2+-dependent secretion activator | CK838951 |
| 3181 | UI-R-AF1-aau-d-07-0-UI | Wolf-Hirschhorn syndrome candidate 2 | CK841084 |
| 3182 | UI-R-Y0-abb-d-09-0-UI | Beaded filament structural protein 1 | AI712944 |
| 3183 | UI-R-Y0-abo-b-05-0-UI | Seizure related 6 homolog -like 2 | BF523475 |
| 3184 | UI-R-Y0-acb-a-05-0-UI | Hairless homolog | AI717428 |
| 3185 | UI-R-BJ0p-aex-e-03-0-UI | BTB (PO)Z domain containing 2 | BF566659 |
| 3186 | UI-R-BJ0p-afg-c-08-0-UI | novel protein | AW434893 |
| 3187 | UI-R-BJ0p-afm-h-07-0-UI | RNA binding motif protein 17 | BF566807 |
| 3188 | UI-R-BJ0p-afw-d-04-0-UI | RIKEN cDNA 2610204K14 | AW434281 |
| 3189 | UI-R-C2p-ri-f-06-0-UI | Dual adaptor for phosphotyrosine and 3-phosphoinositides 1 | AI556635 |
| 3190 | UI-R-C2p-rq-e-08-0-UI | PML-RAR alpha-regulated adaptor molecule-1 | AI500814 |
| 3191 | UI-R-C2p-sa-h-12-0-UI | Msh homeo box homolog 2 | BF546222 |
| 3192 | UI-R-C3-sh-d-11-0-UI | Y box protein 1 | AI511445 |
| 3193 | UI-R-C3-tz-d-10-0-UI | Rho-associated coiled-coil forming kinase 2 | AI548846 |
| 3194 | UI-R-G0-ui-a-07-0-UI | HCV NS3-transactivated protein 1 | AI578211 |
| 3195 | UI-R-G0-uu-e-06-0-UI | Prostaglandin E receptor 2, subtype EP2 | BF522716 |
| 3196 | UI-R-Y0-vb-e-02-0-UI | ATP-binding cassette, sub-family A (ABC1), member 4 | BF523721 |
| 3197 | UI-R-AC1-xr-e-05-0-UI | RIKEN cDNA 2900073H19 | BF524802 |
| 3198 | UI-R-AF0-yg-d-04-0-UI | Growth differentiation factor 1 | CK840577 |
| 3199 | UI-R-E1-fq-a-10-0-UI | D site albumin promoter binding protein | AI112834 |
| 3200 | UI-R-E1-ge-f-09-0-UI | Destrin | AA957145 |
| 3201 | UI-R-C0-ja-e-08-0-UI | NP_001012047.1 | AI029940 |
| 3202 | UI-R-C1-jt-f-11-0-UI | P34 protein | BF554123 |
| 3203 | UI-R-C1-ko-d-02-0-UI | Protein tyrosine phosphatase, receptor type, R | AI071298 |
| 3204 | UI-R-C1-lh-d-12-0-UI | Arrestin domain containing 1 | AI502294 |
| 3205 | UI-R-C2p-og-h-11-0-UI | THO complex 3 | BF552052 |
| 3206 | UI-R-BT0-pl-e-05-0-UI | Glutamate receptor, ionotropic, kainate 5 | BF553684 |
| 3207 | UI-R-BT0-pr-g-10-0-UI | OCIA domain containing 1 | AI145033 |
| 3208 | UI-R-BT0-py-f-01-0-UI | KIAA1919 protein | AI145534 |
| 3209 | UI-R-A0-af-a-01-0-UI | Actin related protein 2/3 complex, subunit 1A | BF548865 |
| 3210 | UI-R-A0-aw-g-11-0-UI | Urinary protein 3 precursor (RUP-3) | AA818561 |
| 3211 | UI-R-A0-bk-h-11-0-UI | CCR4-NOT transcription complex, subunit 2 | AA819395 |
| 3212 | UI-R-E0-br-d-02-0-UI | Enhancer of rudimentary homolog | AA859541 |
| 3213 | UI-R-E0-cw-a-02-0-UI | Lecithin-retinol acyltransferase (phosphatidylcholine-retinol-O-acyltransferase) | AA899076 |
| 3214 | UI-R-E0-dd-b-02-0-UI | CCR4 carbon catabolite repression 4-like | BF555641 |
| 3215 | UI-R-E0-di-g-03-0-UI | Guanine nucleotide binding protein (G protein), gamma 10 | BF556593 |
| 3216 | UI-R-A1-dp-f-02-0-UI | Transmembrane protein 138 | AA901211 |
| 3217 | UI-R-A1-eo-a-04-0-UI | RIKEN cDNA A430093J20 gene | AA925938 |
| 3218 | UI-R-E1-fa-a-08-0-UI | Spindlin-like | AA955567 |
| 3219 | UI-R-CA0-bhf-d-02-0-UI | Leucine rich repeat containing 46 | BF400766 |
| 3220 | UI-R-BJ2-bqq-c-01-0-UI | 2'-5' oligoadenylate synthetase-like 1 | CK843796 |
| 3221 | UI-R-DK0-cfi-h-07-0-UI | Threonyl-tRNA synthetase | BI289683 |
| 3222 | UI-R-E1-ga-d-03-0-UI | Transcribed locus | AA963931 |
| 3223 | UI-R-E1-gn-a-10-0-UI | Solute carrier family 35, member C2 | BF559697 |
| 3224 | UI-R-C0-hz-c-11-0-UI | ELK1, member of ETS oncogene family | AI137804 |
| 3225 | UI-R-C1-js-f-06-0-UI | Transcribed locus | AI044442 |
| 3226 | UI-R-C2p-qv-c-06-0-UI | ATP-binding cassette, sub-family F (GCN20), member 2 | BF546792 |
| 3227 | UI-R-C2p-rl-f-05-0-UI | RIKEN cDNA D930036F22 gene | AI556530 |
| 3228 | UI-R-C0-ip-c-09-0-UI | Interferon dependent positive acting transcription factor 3 gamma | AI029121 |
| 3229 | UI-R-C0-iw-d-11-0-UI | Pleckstrin homology-like domain, family A, member 2 | AI029402 |
| 3230 | UI-R-C1-ka-g-09-0-UI | Replication factor C (activator 1) 3 | AI045639 |
| 3231 | UI-R-C1-kn-g-03-0-UI | CDNA clone IMAGE:7127075, containing frame-shift errors | BF551788 |
| 3232 | UI-R-C1-lg-e-03-0-UI | transcription factor 7-like 2, T-cell specific, HMG-box | AI059480 |
| 3233 | UI-R-C1-ll-f-07-0-UI | XP_001105593.1 mesogenin 1 | AI060267 |
| 3234 | UI-R-E1-fu-b-09-0-UI | Insulin responsive sequence DNA binding protein-1 | AA957394 |
| 3235 | UI-R-C0-if-h-03-0-UI | Rho guanine nucleotide exchange factor (GEF) 5 | BF551245 |
| 3236 | UI-R-C1-jt-a-07-0-UI | Hypothetical LOC294390 | BF551404 |
| 3237 | UI-R-C1-ln-a-02-0-UI | Potassium channel tetramerisation domain containing 3 | AI071931 |
| 3238 | UI-R-C0-hb-a-12-0-UI | UBX domain containing 5 | AA964950 |
| 3239 | UI-R-C0-ip-h-02-0-UI | Frizzled homolog 3 | AI029226 |
| 3240 | UI-R-C0-jq-f-08-0-UI | RNA polymerase 1-2 | AI045820 |
| 3241 | UI-R-C1-ku-g-02-0-UI | Transcribed locus | AI071468 |
| 3242 | UI-R-A1-eu-d-06-0-UI | Tissue specific transplantation antigen P35B | BF556933 |
| 3243 | UI-R-E1-fb-h-05-0-UI | Golgi associated, gamma adaptin ear containing, ARF binding protein 3 | AA956006 |
| 3244 | UI-R-E1-fv-e-01-0-UI | OTU domain, ubiquitin aldehyde binding 1 | AA957759 |
| 3245 | UI-R-E1-fy-e-04-0-UI | Lymphocyte specific 1 | AA957420 |
| 3246 | UI-R-C0-hl-a-02-0-UI | Component of oligomeric golgi complex 8 | BF558104 |
| 3247 | UI-R-C0-il-f-11-0-UI | Hect domain and RLD 4 | BF551318 |
| 3248 | UI-R-E0-dh-h-10-0-UI | Platelet-activating factor acetylhydrolase, isoform 1b, alpha2 subunit | BF558582 |
| 3249 | UI-R-A1-ev-h-04-0-UI | Glutamate receptor interacting protein 2 | BF556974 |
| 3250 | UI-R-C0-im-d-11-0-UI | Apolipoprotein C-IV | AA998783 |
| 3251 | UI-R-C0-iv-b-03-0-UI | Hydroxysteroid 11-beta dehydrogenase 2 | BF551591 |
| 3252 | UI-R-C0-ib-h-02-0-UI | Transforming growth factor, beta 3 | AA998464 |
| 3253 | UI-R-BT0-pz-b-04-0-UI | Nuclear receptor subfamily 4, group A, member 3 | AI145834 |
| 3254 | UI-R-C1-ko-e-11-0-UI | Transcribed locus |  |
| 3255 | UI-R-C2-mr-b-11-0-UI | Protein phosphatase 1, regulatory (inhibitor) subunit 1B | AI070780 |
| 3256 | UI-R-A0-bk-h-08-0-UI | B-cell translocation gene 1, anti-proliferative | AA819392 |
| 3257 | UI-R-E0-cs-g-08-0-UI | Aldo-keto reductase family 1, member B4 (aldose reductase) | BF555165 |
| 3258 | UI-R-C2-ne-g-11-0-UI | Netrin 2-like (chicken) | AI072413 |
| 3259 | UI-R-C2-ng-f-09-0-UI | Alpha thalassemia/mental retardation syndrome X-linked homolog | BF553021 |
| 3260 | UI-R-C2-nj-g-11-0-UI | RIKEN cDNA 2310061F22 | BF553197 |
| 3261 | UI-R-C2-nl-e-03-0-UI | Transcribed locus |  |
| 3262 | UI-R-A0-ae-b-11-0-UI | Secretoglobin, family 1A, member 1 (uteroglobin) | AA817804 |
| 3263 | UI-R-A0-ap-a-10-0-UI | Epoxide hydrolase 2, cytoplasmic | AA819830 |
| 3264 | UI-R-A0-az-d-05-0-UI | Ribosomal protein L36 | AA818709 |
| 3265 | UI-R-A0-bh-d-03-0-UI | Protein phosphatase 2 (formerly 2A), regulatory subunit B (PR 52), alpha isoform | AA819691 |
| 3266 | UI-R-E0-cp-a-11-0-UI | Mercaptopyruvate sulfurtransferase | AA875446 |
| 3267 | UI-R-E0-cx-g-12-0-UI | NAD(P)H dehydrogenase, quinone 1 | BF555573 |
| 3268 | UI-R-C0-jf-h-08-0-UI | putative alpha-mannosidase | BF558000 |
| 3269 | UI-R-C0-jj-a-07-0-UI | Family with sequence similarity 51, member A1 homolog | AI043712 |
| 3270 | UI-R-C1-kd-c-12-0-UI | Ral guanine nucleotide dissociation stimulator | AI044860 |
| 3271 | UI-R-C1-kf-d-07-0-UI | Galactosidase, beta 1 | AI045074 |
| 3272 | UI-R-C1-ki-f-10-0-UI | SPARC-related modular calcium binding protein 1 | BF546923 |
| 3273 | UI-R-C1-kk-b-04-0-UI | Par-6 (partitioning defective 6,) homolog alpha | AI044894 |
| 3274 | UI-R-C1-la-h-02-0-UI | Carbamoyl-phosphate synthetase 1, mitochondrial | BF547163 |
| 3275 | UI-R-C1-le-f-07-0-UI | Four and a half LIM domains 1 | AI059644 |
| 3276 | UI-R-C1-lo-b-09-0-UI | Jumonji domain containing 1C | AI071920 |
| 3277 | UI-R-C1-lp-g-06-0-UI | PX domain containing serine/threonine kinase | BF552293 |
| 3278 | UI-R-A0-bf-c-08-0-UI | Chromodomain helicase DNA binding protein 1-like | AA858645 |
| 3279 | UI-R-E1-fa-d-02-0-UI | Sorting nexin associated golgi protein 1 | BF559305 |
| 3280 | UI-R-E1-fg-d-08-0-UI | Saccharopine dehydrogenase | AA955746 |
| 3281 | UI-R-E1-fk-h-06-0-UI | Mitochondrial ribosomal protein S12 | AA956515 |
| 3282 | UI-R-C0-gx-b-09-0-UI | Nucleoporin 188 | AA964826 |
| 3283 | UI-R-C0-hd-g-07-0-UI | Melan-A | BF557915 |
| 3284 | UI-R-C0-hi-d-12-0-UI | RIKEN cDNA 1810065E05 | AA996579 |
| 3285 | UI-R-C0-hu-d-01-0-UI | Protein phosphatase 1J | AA997978 |
| 3286 | UI-R-C0-it-d-02-0-UI | Mitochondrial ribosomal protein L27 | AI030248 |
| 3287 | UI-R-C0-iy-f-05-0-UI | Glutathione peroxidase 2 | BF551735 |
| 3288 | UI-R-C0-ht-h-08-0-UI | Asialoglycoprotein receptor 2 | AA997920 |
| 3289 | UI-R-C0-iy-h-08-0-UI | XP_001097034.1 Immunoglobulin lambda-like polypeptide 1 precursor (Immunoglobulin-related protein 14.1) (Immunoglobulin omega polypeptide) (Ig lambda-5) (CD179b antigen) | AI029631 |
| 3290 | UI-R-C1-kg-b-07-0-UI | Purkinje cell protein 4 | BF551551 |
| 3291 | UI-R-C1-kn-f-03-0-UI | Mannosidase, alpha, class 2C, member 1 | BF551785 |
| 3292 | UI-R-C1-kw-g-03-0-UI | Ly6-C antigen | AI059537 |
| 3293 | UI-R-C1-le-f-10-0-UI | GATA binding protein 4 | AI059647 |
| 3294 | UI-R-C2p-nu-b-07-0-UI | Frequenin homolog | BF553302 |
| 3295 | UI-R-C2p-oe-g-08-0-UI | Gamma-glutamyl carboxylase | AI137138 |
| 3296 | UI-R-BT0-pn-e-01-0-UI | Dynactin 1 | AI144590 |
| 3297 | UI-R-A0-ae-a-02-0-UI | Apolipoprotein A-I binding protein | BF548079 |
| 3298 | UI-R-CV2-chm-f-06-0-UI | Retinitis pigmentosa GTPase regulator interacting protein 1 | BI298693 |
| 3299 | UI-R-DL0-cip-h-14-0-UI | Brain-specific identifier sequence RNA, clone p1b224 | BI302202 |
| 3300 | UI-R-DN0-ciu-f-07-0-UI | TCF3 (E2A) fusion partner | BI291614 |
| 3301 | UI-R-DO0-ciw-j-06-0-UI | Eukaryotic translation elongation factor 1 alpha 1 | BI292340 |
| 3302 | UI-R-A1-dz-e-10-0-UI | Xanthine dehydrogenase | AA924540 |
| 3303 | UI-R-A1-ec-e-10-0-UI | Sorbitol dehydrogenase | AA925452 |
| 3304 | UI-R-A1-eg-c-01-0-UI | Myosin, heavy polypeptide 6, cardiac muscle, alpha | BF555757 |
| 3305 | UI-R-A1-ek-g-05-0-UI | Solute carrier family 27 (fatty acid transporter), member 1 | AI112906 |
| 3306 | UI-R-C0-hh-f-11-0-UI | Carnitine palmitoyltransferase 1b, muscle | BF561250 |
| 3307 | UI-R-C0-hn-h-09-0-UI | Protein tyrosine phosphatase, non-receptor type substrate 1 | AA998869 |
| 3308 | UI-R-CU0s-cbr-g-04-0-UI | Fasciculation and elongation protein zeta 2 (zygin II) | BI279856 |
| 3309 | UI-R-CU0s-cby-b-09-0-UI | 60S ribosomal protein L18a | BI282231 |
| 3310 | UI-R-DK0-cdg-e-10-0-UI | Procollagen, type XVIII, alpha 1 | BI288582 |
| 3311 | UI-R-DK0-cdt-a-08-0-UI | Centrin 3 | BI297078 |
| 3312 | UI-R-DK0-cec-a-11-0-UI | SR rich protein | BI294781 |
| 3313 | UI-R-DK0-cef-a-06-0-UI | RIKEN cDNA 1700027M01 | BI295035 |
| 3314 | UI-R-CA1-bjy-f-04-0-UI | Cytochrome P450, subfamily 11B, polypeptide 1 | BF409942 |
| 3315 | UI-R-BJ2-bqz-h-07-0-UI | Titin | BF408117 |
| 3316 | UI-R-BJ2-bre-c-11-0-UI | Phospholipase B | CK845196 |
| 3317 | UI-R-DK0-cfn-g-09-0-UI | mKIAA0673 protein | BI289753 |
| 3318 | UI-R-BJ2-bor-d-07-0-UI | Putative C3orf6 protein homolog | BF414658 |
| 3319 | UI-R-BJ2-bph-b-06-0-UI | Notch-regulated ankyrin repeat protein | BF419865 |
| 3320 | UI-R-BJ2-bpo-f-06-0-UI | hypothetical protein 6030458H05 | BF406746 |
| 3321 | UI-R-BJ2-bpw-d-12-0-UI | Dynein, cytoplasmic, heavy polypeptide 2 | BF420435 |
| 3322 | UI-R-CW0-bwn-f-01-0-UI | Eukaryotic translation initiation factor 3, subunit 12 | BI276057 |
| 3323 | UI-R-CX0-bxc-c-12-0-UI | NHP2-like protein 1 | BI275165 |
| 3324 | UI-R-CX0-bxj-h-04-0-UI | Solute carrier family 30 (zinc transporter), member 1 | BI276736 |
| 3325 | UI-R-CY0-bxr-e-12-0-UI | Allograft inflammatory factor 1 | BI278279 |
| 3326 | UI-R-DE0-caf-g-10-0-UI | Adrenergic receptor, beta 2 | BI279712 |
| 3327 | UI-R-CT0s-cau-d-06-0-UI | Ribosomal protein S5 | BI281610 |
| 3328 | UI-R-CA0-bhx-h-01-0-UI | Adenylate cyclase 2 | BF399729 |
| 3329 | UI-R-CA1-bio-g-07-0-UI | Phosducin-like | BF405033 |
| 3330 | UI-R-CS0-bsd-e-03-0-UI | Zyxin | BG373497 |
| 3331 | UI-R-CV1-bso-g-01-0-UI | Superoxide dismutase 3, extracellular | BG374384 |
| 3332 | UI-R-CS0-bth-f-12-0-UI | Pyridoxine 5'-phosphate oxidase | BG379506 |
| 3333 | UI-R-CT0-btw-e-08-0-UI | G protein-coupled receptor 162 | BG380383 |
| 3334 | UI-R-CV1-bvp-e-09-0-UI | C-terminal binding protein 1 | BG378228 |
| 3335 | UI-R-CA0-bkg-h-05-0-UI | Fbxw17 protein | BF415920 |
| 3336 | UI-R-CA0-bkv-d-10-0-UI | Itchy homolog E3 ubiquitin protein ligase | BF416821 |
| 3337 | UI-R-CN0-blg-a-12-0-UI | WD repeat domain 18 | BF417433 |
| 3338 | UI-R-BS1-ayv-e-10-0-UI | Zinc finger protein 498 | BE108375 |
| 3339 | UI-R-BS1-azd-h-04-0-UI | Ubiquitin ligase protein DZIP3 (DAZ-interacting protein 3 homolog) | BE109271 |
| 3340 | UI-R-BJ1-azq-f-06-0-UI | Cyclin-dependent kinase inhibitor 2C (p18, inhibits CDK4) | CK845367 |
| 3341 | UI-R-CA0-bag-c-06-0-UI | Coagulation factor VIII | BE119566 |
| 3342 | UI-R-CA1-bco-a-11-0-UI | G protein-coupled receptor 37 | BF390681 |
| 3343 | UI-R-BS2-bdd-a-04-0-UI | Dicer1, Dcr-1 homolog | BF388566 |
| 3344 | UI-R-BS2-bdm-g-08-0-UI | Kinesin family member 15 | BF389349 |
| 3345 | UI-R-BS2-bdy-h-06-0-UI | Ubiquitin-conjugating enzyme E2D 3 (UBC4/5 homolog, yeast) | BF396503 |
| 3346 | UI-R-CA0-bgw-b-02-0-UI | Annexin V-binding protein ABP-10 | BF393882 |
| 3347 | UI-R-CA0-bhg-c-01-0-UI | Protein tyrosine phosphatase, non-receptor type 7 | BF400789 |
| 3348 | UI-R-BT1-aqg-g-06-0-UI | Solute carrier family 31, member 2 | BE101666 |
| 3349 | UI-R-BT1-aqt-e-12-0-UI | Lck-interacting transmembrane adaptor protein LIME | BE102815 |
| 3350 | UI-R-BJ1-atk-d-12-0-UI | Cystatin E/M | CK842912 |
| 3351 | UI-R-BJ1-atq-a-12-0-UI | Breast carcinoma amplified sequence 3 homolog (K20D4) | BE099772 |
| 3352 | UI-R-BJ1-atw-a-12-0-UI | Dner protein | BE099443 |
| 3353 | UI-R-BJ1-aue-g-02-0-UI | Peroxisome biogenesis factor 16 | CK839504 |
| 3354 | UI-R-BJ1-avx-h-06-0-UI | Glutaredoxin 2 (thioltransferase) | BE111795 |
| 3355 | UI-R-BJ1-awc-b-09-0-UI | EGL nine homolog 2 | BE113156 |
| 3356 | UI-R-BJ1-awj-c-09-0-UI | Proline synthetase co-transcribed | BE114116 |
| 3357 | UI-R-BJ1-awu-b-12-0-UI | Nebulette | BE115010 |
| 3358 | UI-R-BO1-aiy-b-01-0-UI | Acyl-CoA synthetase long-chain family member 3 | AW525996 |
| 3359 | UI-R-BO1-ajf-d-02-0-UI | Transcribed locus | AW526901 |
| 3360 | UI-R-BO1-aji-h-01-0-UI | Butyrylcholinesterase | AW531512 |
| 3361 | UI-R-BO1-ajo-f-08-0-UI | Solute carrier family 2 (facilitated glucose transporter), member 6 | BF565679 |
| 3362 | UI-R-C4-aky-e-05-0-UI | High mobility group box 2-like 1 | AW531658 |
| 3363 | UI-R-C4-alg-f-08-0-UI | Hypothetical LOC287938 | AW531098 |
| 3364 | UI-R-C4-alo-b-03-0-UI | CG8841-PA | AW534331 |
| 3365 | UI-R-C4-alw-e-01-0-UI | Eukaryotic translation initiation factor 4 gamma, 2 | BF564540 |
| 3366 | UI-R-BU0-apg-e-06-0-UI | Kinesin light chain 1 | BE096471 |
| 3367 | UI-R-BO1-apu-f-07-0-UI | Homeodomain interacting protein kinase 3 | BE097216 |
| 3368 | UI-R-AA1-zy-a-02-0-UI | Proliferation-associated 2G4 | CK839184 |
| 3369 | UI-R-AG1-aal-c-08-0-UI | beta 3-glycosyltransferase-like | CK838410 |
| 3370 | UI-R-Y0-ach-c-09-0-UI | Hyaluronan and proteoglycan link protein 4 | AI716459 |
| 3371 | UI-R-Y0-acu-c-07-0-UI | Leucine aminopeptidase 3 | AI717041 |
| 3372 | UI-R-BJ0-adb-a-02-0-UI | Ras-induced senescence 1 | AW251591 |
| 3373 | UI-R-BJ0-aeb-b-04-0-UI | Testis expressed gene 264 homolog | AW252660 |
| 3374 | UI-R-BJ0p-agd-g-05-0-UI | Ser/Thr-rich protein T10 in DGCR region | BF567018 |
| 3375 | UI-R-BO0-agl-c-11-0-UI | Protein kinase C, gamma | AW521334 |
| 3376 | UI-R-BO0-agu-h-08-0-UI | Glutaminyl-peptide cyclotransferase-like | AW521959 |
| 3377 | UI-R-BO0-ahn-f-08-0-UI | Serine carboxypeptidase 1 | AW522867 |
| 3378 | UI-R-C3-sq-d-05-0-UI | Glutamic pyruvate transaminase (alanine aminotransferase) 2 | AI535168 |
| 3379 | UI-R-C3-sz-b-03-0-UI | Bifunctional apoptosis regulator | BF543475 |
| 3380 | UI-R-C3-tj-e-12-0-UI | PDZ and LIM domain 5 | BF523272 |
| 3381 | UI-R-C3-tr-c-09-0-UI | CG11030-PA | AI548760 |
| 3382 | UI-R-AB0-vr-h-07-0-UI | Ubiquitin-conjugating enzyme E2C | CK840362 |
| 3383 | UI-R-AD0-wf-g-06-0-UI | A kinase (PRKA) anchor protein 3 | CK842323 |
| 3384 | UI-R-AG0-wt-e-06-0-UI | Myosin, heavy polypeptide 7, cardiac muscle, beta | AI579057 |
| 3385 | UI-R-AG0-xc-a-04-0-UI | Glypican 4 | BF524293 |
| 3386 | UI-R-AE1-zi-g-01-0-UI | Receptor-associated protein of the synapse | CK839111 |
| 3387 | UI-R-AD1-zo-g-11-0-UI | Cholinergic receptor, nicotinic, beta polypeptide 1 | CK839718 |
| 3388 | UI-R-C0-hr-b-03-0-UI | COP9 (constitutive photomorphogenic) homolog, subunit 6 | BF561392 |
| 3389 | UI-R-C0-ib-g-08-0-UI | Spire homolog 2 | BF561697 |
| 3390 | UI-R-Y0-lx-f-08-0-UI | Proprotein convertase subtilisin/kexin type 7 | AI073232 |
| 3391 | UI-R-Y0-mo-d-11-0-UI | mKIAA0256 protein | AI111971 |
| 3392 | UI-R-C2-na-h-05-0-UI | DiGeorge syndrome critical region gene 2 | BF521786 |
| 3393 | UI-R-C2p-nv-g-07-0-UI | Paired-like homeodomain transcription factor 1 | BF553427 |
| 3394 | UI-R-BT0-qh-c-12-0-UI | F-box protein FBL2 | BF522267 |
| 3395 | UI-R-C2p-qn-d-01-0-UI | XP_001113124.1 Ras-related GTP binding C | BF547277 |
| 3396 | UI-R-C2p-qs-a-09-0-UI | Selectin, platelet | AI555742 |
| 3397 | UI-R-C2p-rc-d-10-0-UI | Amyloid beta (A4) precursor protein-binding, family B, member 1 interacting protein | AI555954 |
| 3398 | UI-R-E0-bv-b-05-0-UI | Transcribed locus |  |
| 3399 | UI-R-E0-bz-d-10-0-UI | px19-like protein | BF547851 |
| 3400 | UI-R-E0-cg-e-11-0-UI | RIKEN cDNA 4930556P03 | AA874826 |
| 3401 | UI-R-E0-cm-h-12-0-UI | Ancient ubiquitous protein 1 precursor | BF554999 |
| 3402 | UI-R-A1-dv-a-02-0-UI | Vesicle-associated membrane protein 3 | BF555703 |
| 3403 | UI-R-A1-dy-f-08-0-UI | Ribosomal protein L35a | BF559578 |
| 3404 | UI-R-A1-ee-c-12-0-UI | Transient receptor potential-related protein, ChaK | AA925366 |
| 3405 | UI-R-A1-ei-g-09-0-UI | Transcribed locus | BF555393 |
| 3406 | UI-R-E1-gq-f-10-0-UI | XP_001097255.1 zinc finger and BTB domain containing 37 | BF561175 |
| 3407 | UI-R-C0-hj-g-10-0-UI | Adenosine deaminase | AA996935 |
| 3408 | UI-R-Y0-ma-g-04-0-UI | Heat shock 70kDa protein 14 | AI111366 |
| 3409 | UI-R-AG1-aak-d-02-0-UI | ATP-binding cassette, sub-family B (MDR/TAP), member 8 | BF543109 |
| 3410 | UI-R-BT1-akr-e-04-0-UI | RNA (guanine-9-) methyltransferase domain containing 1 | AW530025 |
| 3411 | UI-R-CA0-baj-d-08-0-UI | Aspartate beta-hydroxylase domain containing 2 | BE119910 |
| 3412 | UI-R-E0-bz-g-11-0-UI | ASF1 anti-silencing function 1 homolog B | BF547865 |
| 3413 | UI-R-E0-de-b-12-0-UI | Dystrobrevin, beta | BF544124 |
| 3414 | UI-R-A1-dv-b-01-0-UI | S100 calcium binding protein A13 | BF555708 |
| 3415 | UI-R-E1-fb-e-06-0-UI | nidogen 2 | AA955978 |
| 3416 | UI-R-C2-mt-d-03-0-UI | Bridging integrator 2 | AI070908 |
| 3417 | UI-R-C2p-oe-a-10-0-UI | Mitochondrial ribosomal protein L55 | AI137082 |
| 3418 | UI-R-C0-hc-a-07-0-UI | Transmembrane protein 54 | AA965164 |
| 3419 | UI-R-C0-hh-c-07-0-UI | nitrogen fixation cluster-like | AA996887 |
| 3420 | UI-R-C0-je-g-09-0-UI | Putative ATP-dependent Clp protease proteolytic subunit, mitochondrial precursor | AI031012 |
| 3421 | UI-R-C0-jo-b-06-0-UI | Transcription factor CP2-like 2 | BF550778 |
| 3422 | UI-R-C1-jt-b-07-0-UI | ADP-ribosylation factor interacting protein 2 | AI045973 |
| 3423 | UI-R-C1-jw-d-03-0-UI | Moloney leukemia virus 10 | AI044570 |
| 3424 | UI-R-E0-br-e-05-0-UI | Actin alpha cardiac 1 | AA866452 |
| 3425 | UI-R-C1-kq-h-01-0-UI | Carcinoembryonic antigen gene family (CGM3) | AI058888 |
| 3426 | UI-R-A0-am-e-02-0-UI | RIKEN cDNA 1700048E23 | AA818091 |
| 3427 | UI-R-A1-ey-g-10-0-UI | ATP-binding cassette, sub-family E (OABP), member 1 | AA955066 |
| 3428 | UI-R-E1-ga-a-11-0-UI | RNA binding motif protein 16 | BF560418 |
| 3429 | UI-R-A0-bg-g-01-0-UI | Hyperpolarization-activated cyclic nucleotide-gated potassium channel 1 | AA866238 |
| 3430 | UI-R-A1-ev-a-07-0-UI | Unc-5 homolog C | AA926250 |
| 3431 | UI-R-C0-gs-b-04-0-UI | XP_001111106.1 KH domain-containing, RNA-binding, signal transduction-associated protein 2 | AA963967 |
| 3432 | UI-R-A0-av-h-06-0-UI | S100 calcium binding protein A11 (calizzarin) | AA819765 |
| 3433 | UI-R-A0-bh-d-05-0-UI | ATP synthase mitochondrial F1 complex assembly factor 2 | BF548057 |
| 3434 | UI-R-A0-ay-a-10-0-UI | 60S ribosomal protein L12 | AA818753 |
| 3435 | UI-R-A1-es-c-12-0-UI | zinc finger protein 198 | AA926013 |
| 3436 | UI-R-C0-gt-e-10-0-UI | Histone cell cycle regulation defective interacting protein 5 | BF557236 |
| 3437 | UI-R-C0-gz-c-10-0-UI | Ras association (RalGDS/AF-6) domain family 2 | BF556702 |
| 3438 | UI-R-E0-ce-f-04-0-UI | Ribosomal protein L35a | BF555006 |
| 3439 | UI-R-E0-ck-d-01-0-UI | Phosphatidylinositol 4-kinase, catalytic, beta polypeptide | BF554951 |
| 3440 | UI-R-E1-fg-d-04-0-UI | G protein-coupled receptor kinase 5 | AA956162 |
| 3441 | UI-R-E1-fs-a-03-0-UI | Eukaryotic translation initiation factor 2B, subunit 1 alpha | AA957084 |
| 3442 | UI-R-E1-gc-h-07-0-UI | CD24 antigen | AA958018 |
| 3443 | UI-R-C0-ie-g-10-0-UI | Defensin beta 1 | BF561623 |
| 3444 | UI-R-E1-fb-c-03-0-UI | Calpain 10 | AA955623 |
| 3445 | UI-R-C0-gs-a-11-0-UI | Dishevelled, dsh homolog 1 | BF559628 |
| 3446 | UI-R-C0-ir-e-04-0-UI | Hairy and enhancer of split 5 | AI029446 |
| 3447 | UI-R-C1-jt-f-01-0-UI | G-protein coupled receptor 88 | AI044798 |
| 3448 | UI-R-C2-mv-e-07-0-UI | Fragile X mental retardation gene 1, autosomal homolog | BF553097 |
| 3449 | UI-R-C2-mz-g-08-0-UI | PHD zinc finger containing protein JUNE1 | AI072164 |
| 3450 | UI-R-C2-nb-g-04-0-UI | Retinol dehydrogenase 10 (all-trans) | AI071260 |
| 3451 | UI-R-C2-ne-a-11-0-UI | Hypothetical protein MGC:15854 | BF549634 |
| 3452 | UI-R-C2-no-h-02-0-UI | Interferon gamma receptor 1 | AI070768 |
| 3453 | UI-R-C2p-nu-d-07-0-UI | Hypoxia induced gene 1 | BF553360 |
| 3454 | UI-R-C2p-nx-e-06-0-UI | porcupine-D | BF553647 |
| 3455 | UI-R-C2p-oa-h-06-0-UI | XP_001099397.1 HBxAg transactivated protein 2 isoform 5 | AI136338 |
| 3456 | UI-R-E0-bs-g-03-0-UI | Serine (or cysteine) proteinase inhibitor, clade H, member 1 | BF548013 |
| 3457 | UI-R-E0-by-c-04-0-UI | Ribosomal protein L5 | AA900622 |
| 3458 | UI-R-C0-ii-e-11-0-UI | transcription factor (p38 interacting protein) | AA998746 |
| 3459 | UI-R-C0-ik-g-04-0-UI | TSC22 domain family 3 | AI029054 |
| 3460 | UI-R-C0-jp-f-09-0-UI | Calcyphosine-like | BF550978 |
| 3461 | UI-R-C1-jx-f-02-0-UI | Proteasome (prosome, macropain) 26S subunit, non-ATPase, 12 | AI044322 |
| 3462 | UI-R-C1-jy-h-11-0-UI | hypothetical protein BC013949 | BF550880 |
| 3463 | UI-R-C1-kb-h-04-0-UI | Serine/threonine kinase 39, STE20/SPS1 homolog | BF550938 |
| 3464 | UI-R-C1-kq-g-12-0-UI | YY1-associated factor 2 | AI059183 |
| 3465 | UI-R-C1-ks-e-06-0-UI | Transcribed locus | BF552562 |
| 3466 | UI-R-C1-kx-b-02-0-UI | RIKEN cDNA 1110059G10 | BF546356 |
| 3467 | UI-R-C1-kz-c-05-0-UI | Dentin matrix protein 1 | BF558455 |
| 3468 | UI-R-A0-al-e-11-0-UI | NHP2-like protein 1 | BF548986 |
| 3469 | UI-R-A0-ap-g-08-0-UI | Nucleotide binding protein 1 | AA819729 |
| 3470 | UI-R-A0-as-b-04-0-UI | RIKEN cDNA 2010001H14 | BF548813 |
| 3471 | UI-R-A0-be-b-02-0-UI | Sec11-like 3 | AA858716 |
| 3472 | UI-R-E1-fo-e-03-0-UI | A disintegrin-like and metallopeptidase (reprolysin type) with thrombospondin type 1 motif, 7 | BF560987 |
| 3473 | UI-R-E1-ft-h-09-0-UI | Microfibrillar associated protein 5 | AI137744 |
| 3474 | UI-R-E1-gp-c-01-0-UI | cisplatin resistance-associated overexpressed protein | AA964393 |
| 3475 | UI-R-C0-gw-b-07-0-UI | Transmembrane 9 superfamily member 1 | BF557792 |
| 3476 | UI-R-C0-ia-h-10-0-UI | XP_221310.4 putative membrane protein Re9 | BF561654 |
| 3477 | UI-R-C0-id-c-05-0-UI | hypothetical protein MGC2494 | AA998064 |
| 3478 | UI-R-C0-gx-e-04-0-UI | Casein beta | BF557839 |
| 3479 | UI-R-C0-hd-b-02-0-UI | Myosin, light polypeptide 1 | AA965256 |
| 3480 | UI-R-C0-jl-b-10-0-UI | CD82 antigen | AI043632 |
| 3481 | UI-R-C0-jr-h-03-0-UI | Expressed in non-metastatic cells 1 | AI030088 |
| 3482 | UI-R-C1-jw-e-10-0-UI | Ferredoxin 1 | AI044488 |
| 3483 | UI-R-C1-kd-g-05-0-UI | Interleukin 1 beta | BF546451 |
| 3484 | UI-R-C1-ln-h-02-0-UI | Nucleoporin 88 | AI071037 |
| 3485 | UI-R-C2-mu-b-01-0-UI | Granzyme K | AI072219 |
| 3486 | UI-R-C2-nb-h-11-0-UI | Splicing factor, arginine/serine-rich 10 (transformer 2 homolog, Drosophila) | AI071431 |
| 3487 | UI-R-C2-nn-h-12-0-UI | Glutamate receptor, ionotropic, AMPA2 | BF542314 |
| 3488 | UI-R-DK0-cfy-c-01-0-UI | Glucosamine (N-acetyl)-6-sulfatase | BI290687 |
| 3489 | UI-R-DK0-cgd-f-01-0-UI | Diamine oxidase-like protein 1 | BI290553 |
| 3490 | UI-R-CV2-cgn-e-09-0-UI | mKIAA1002 protein | BI291270 |
| 3491 | UI-R-CV2-chb-e-01-0-UI | 5-hydroxytryptamine (serotonin) receptor 1F | BI297856 |
| 3492 | UI-R-DR0-cjb-m-24-0-UI | Pannexin 3 | BI303803 |
| 3493 | UI-R-A1-do-a-07-0-UI | Transforming growth factor beta 1 induced transcript 4 | AI137902 |
| 3494 | UI-R-A1-ds-g-04-0-UI | Dipeptidase 1 (renal) | BF558761 |
| 3495 | UI-R-A1-dv-g-09-0-UI | Serine (or cysteine) peptidase inhibitor, clade A, member 3N | AA901070 |
| 3496 | UI-R-E1-gj-d-03-0-UI | G protein-coupled receptor kinase 2, groucho gene related | AA963235 |
| 3497 | UI-R-C0-gt-b-02-0-UI | Calcium channel, voltage-dependent, beta 2 subunit | BF557229 |
| 3498 | UI-R-DA0-bze-f-07-0-UI | Proline-rich proteoglycan 2 | BI283615 |
| 3499 | UI-R-DD0-bzy-h-07-0-UI | keratin Kb40 | BI282595 |
| 3500 | UI-R-CW0s-ccg-f-07-0-UI | Creatine kinase, muscle | BI283696 |
| 3501 | UI-R-CX0s-ccq-d-04-0-UI | Prolactin family 8, subfamily a, member 3 | BI284287 |
| 3502 | UI-R-CX0s-ccw-e-08-0-UI | THAP domain containing 7 | CK845529 |
| 3503 | UI-R-DK0-cdd-g-01-0-UI | Spindlin | BI288682 |
| 3504 | UI-R-DK0-ceu-g-11-0-UI | Ion transporter protein | BI295720 |
| 3505 | UI-R-DK0-cfb-c-11-0-UI | RIKEN cDNA 1110007C09 | BI295844 |
| 3506 | UI-R-DK0-cff-g-08-0-UI | Wiskott-Aldrich syndrome protein interacting protein | BI289588 |
| 3507 | UI-R-CM0-bjq-g-02-0-UI | Nuclear factor of kappa light polypeptide gene enhancer in B-cells inhibitor, zeta | BF395369 |
| 3508 | UI-R-BT1-bmm-a-09-0-UI | RAS-like, family 10, member B | BF411117 |
| 3509 | UI-R-BT1-bng-g-06-0-UI | CDC91 cell division cycle 91-like 1 | BG378980 |
| 3510 | UI-R-CA0-boc-a-11-0-UI | Hypothetical protein | BF412896 |
| 3511 | UI-R-BJ2-boo-b-04-0-UI | FKSG24 | BF414244 |
| 3512 | UI-R-BJ2-bql-h-04-0-UI | Arginine/serine-rich coiled-coil 1 | CK843734 |
| 3513 | UI-R-BJ2-bqq-a-01-0-UI | DNA primase, p49 subunit | CK843791 |
| 3514 | UI-R-CW0-bvz-c-11-0-UI | Transcribed locus |  |
| 3515 | UI-R-CW0-bwj-b-06-0-UI | Methylcrotonoyl-Coenzyme A carboxylase 1 (alpha) | BI274460 |
| 3516 | UI-R-CZ0-byi-e-05-0-UI | Leucine zipper domain protein | BI279047 |
| 3517 | UI-R-DB0-byt-f-11-0-UI | Secretoglobin, family 1D, member 2 | BI278989 |
| 3518 | UI-R-CA0-bgb-h-02-0-UI | XP_001098218.1 GLI-Kruppel family member HKR1 | BF393779 |
| 3519 | UI-R-CA0-bgp-c-06-0-UI | Calcium/calmodulin-dependent protein kinase kinase 2, beta | BF393036 |
| 3520 | UI-R-CA1-bjb-f-02-0-UI | RIKEN cDNA 2810427I04; DNA segment, Chr 8, ERATO Doi 590, expressed | BF399501 |
| 3521 | UI-R-CA1-bje-c-13-0-UI | MAS-related GPR, member F | BF405725 |
| 3522 | UI-R-CV0-brk-a-03-0-UI | XP_001114732.1 flavin adenine dinucleotide synthetase isoform 1 isoform 2 | BG371921 |
| 3523 | UI-R-CV1-brz-b-11-0-UI | Interleukin 4 receptor, alpha | BG372672 |
| 3524 | UI-R-CT0-bui-h-11-0-UI | Brain-specific angiogenesis inhibitor 1-associated protein 2 | BG381333 |
| 3525 | UI-R-CU0-bus-c-03-0-UI | Transcribed locus | BG376245 |
| 3526 | UI-R-CU0-bvc-a-07-0-UI | Ras-related C3 botulinum toxin substrate 1 | BG377315 |
| 3527 | UI-R-CU0-bvl-h-03-0-UI | Discs, large homolog-associated protein 4 | BG376984 |
| 3528 | UI-R-CA0-axf-b-03-0-UI | Polyamine modulated factor 1 binding protein 1 | BE108510 |
| 3529 | UI-R-CA0-axp-d-06-0-UI | Calbindin 2 | BE114366 |
| 3530 | UI-R-BS1-ayc-h-12-0-UI | PHD finger protein 14 isoform 1 | BE116411 |
| 3531 | UI-R-BS1-ays-d-09-0-UI | Wolf-Hirschhorn syndrome candidate 1 | BE107322 |
| 3532 | UI-R-CA0-bav-h-12-0-UI | COP9 (constitutive photomorphogenic) homolog, subunit 4 | BE120649 |
| 3533 | UI-R-CA1-bbf-d-09-0-UI | Solute carrier organic anion transporter family, member 1a5 | BF386606 |
| 3534 | UI-R-CA1-bbx-e-11-0-UI | Cancer susceptibility candidate 4 | BF388040 |
| 3535 | UI-R-CA1-bcf-g-02-0-UI | Calcium channel, voltage-dependent, beta 4 subunit | BF391063 |
| 3536 | UI-R-BS2-ber-d-06-0-UI | Phosphatidylinositol glycan, class S | BF398659 |
| 3537 | UI-R-CA0-bfi-c-06-0-UI | Solute carrier family 7 (cationic amino acid transporter, y+ system), member 5 | BF392363 |
| 3538 | UI-R-BS0-anv-c-08-0-UI | Ryanodine receptor 1, skeletal muscle | AW534727 |
| 3539 | UI-R-BU0-apd-a-10-0-UI | Interleukin 11 receptor, alpha chain 1 | BE095910 |
| 3540 | UI-R-BX0-arw-g-12-0-UI | cell division cycle and apoptosis regulator 1 | BE104971 |
| 3541 | UI-R-BO1-ask-h-11-0-UI | Heterogeneous nuclear ribonucleoprotein U | BE106102 |
| 3542 | UI-R-BT1-asv-d-07-0-UI | SplA/ryanodine receptor domain and SOCS box containing 1 | BE106389 |
| 3543 | UI-R-BJ1-atg-b-12-0-UI | Discoidin, CUB and LCCL domain containing 2 | BE098908 |
| 3544 | UI-R-BJ1-aus-a-11-0-UI | Cyclin D3 | BE110796 |
| 3545 | UI-R-BJ1-ave-e-11-0-UI | Glutaminase | CK844098 |
| 3546 | UI-R-BJ1-avp-b-11-0-UI | Euchromatic histone lysine N-methyltransferase 2 | CK842603 |
| 3547 | UI-R-BJ1-avu-g-12-0-UI | Annexin A5 | BE110683 |
| 3548 | UI-R-BO0-aia-a-12-0-UI | paraneoplastic antigen | AW524583 |
| 3549 | UI-R-BO0-aig-f-11-0-UI | hypothetical protein FLJ22490 | AW524299 |
| 3550 | UI-R-BJ0p-ain-g-02-0-UI | neuron navigator 1 | BF562806 |
| 3551 | UI-R-BJ0p-aiu-e-04-0-UI | Cyclin-dependent kinase 5 | AW525675 |
| 3552 | UI-R-BT1-ajx-g-01-0-UI | Gem (nuclear organelle) associated protein 6 | AW528374 |
| 3553 | UI-R-BT1-akg-b-05-0-UI | Histidine ammonia lyase | AW527961 |
| 3554 | UI-R-BT1-akm-a-08-0-UI | Ubiquitin specific protease 5 (isopeptidase T) | BF565570 |
| 3555 | UI-R-BT1-aku-a-12-0-UI | Neurexophilin 1 | BF563847 |
| 3556 | UI-R-BU0-amu-h-03-0-UI | downregulated in renal cell carcinoma | BF564677 |
| 3557 | UI-R-BU0-anc-f-07-0-UI | mKIAA0056 protein | BF562345 |
| 3558 | UI-R-AB1-yt-h-08-0-UI | Kinesin family member C1 | CK845100 |
| 3559 | UI-R-AB1-za-g-05-0-UI | XP_001084401.1 60S ribosomal protein L26 | CK840932 |
| 3560 | UI-R-AF1-aax-g-11-0-UI | Neurotrophin receptor associated death domain | CK845657 |
| 3561 | UI-R-Y0-abg-e-12-0-UI | Intersectin 1 | CK845355 |
| 3562 | UI-R-Y0-abt-g-09-0-UI | Tau tubulin kinase 1 | BF523667 |
| 3563 | UI-R-Y0-acd-d-08-0-UI | hypothetical protein FLJ10233 | AI716048 |
| 3564 | UI-R-BJ0p-afd-c-01-0-UI | Dynein cytoplasmic 1 heavy chain 1 | CK839239 |
| 3565 | UI-R-BJ0p-afl-c-09-0-UI | cDNA sequence BC005537 | CK841487 |
| 3566 | UI-R-BJ0p-afq-e-12-0-UI | Early growth response 2 | BF566855 |
| 3567 | UI-R-BJ0p-aga-c-12-0-UI | BMP and activin membrane-bound inhibitor, homolog | CK841692 |
| 3568 | UI-R-C2p-rl-h-08-0-UI | Butyryl Coenzyme A synthetase 1 | BF547389 |
| 3569 | UI-R-C2p-ru-a-01-0-UI | Cardiomyopathy associated 3 | BF547469 |
| 3570 | UI-R-C2p-sc-g-03-0-UI | Pecanex homolog | AI501572 |
| 3571 | UI-R-C3-sl-b-11-0-UI | 1-acylglycerol-3-phosphate O-acyltransferase 7 (lysophosphatidic acid acyltransferase, eta) | AI547459 |
| 3572 | UI-R-G0-uc-a-04-0-UI | Melanoma antigen, family E, 1 | AI574673 |
| 3573 | UI-R-G0-up-e-07-0-UI | Transcribed locus |  |
| 3574 | UI-R-Y0-uy-g-09-0-UI | Kinesin heavy chain family, member 2 | AI575858 |
| 3575 | UI-R-Y0-vi-f-05-0-UI | Myelin and lymphocyte protein, T-cell differentiation protein | BF522779 |
| 3576 | UI-R-AF0-ya-g-05-0-UI | elongation factor 1 homolog | CK838804 |
| 3577 | UI-R-AC0-yl-e-06-0-UI | retinoid x receptor interacting protein | AI705987 |
| 3578 | UI-R-E1-gb-d-09-0-UI | Serpine1 mRNA binding protein 1 | AA957624 |
| 3579 | UI-R-E1-gj-c-07-0-UI | Lysyl-tRNA synthetase | AA963227 |
| 3580 | UI-R-C0-jk-a-11-0-UI | Splicing factor, arginine/serine-rich 8 (suppressor-of-white-apricot homolog, Drosophila) | AI043919 |
| 3581 | UI-R-C1-kh-b-11-0-UI | Low density lipoprotein receptor-related protein 1 | AI045233 |
| 3582 | UI-R-C1-kz-d-10-0-UI | Stomatin | BF547114 |
| 3583 | UI-R-C1-lp-c-03-0-UI | Kelch-like 2, Mayven | BF559955 |
| 3584 | UI-R-C2p-oj-d-01-0-UI | Nibrin | AI137049 |
| 3585 | UI-R-BT0-po-h-02-0-UI | Tripartite motif protein 45 | BF544228 |
| 3586 | UI-R-BT0-pu-c-06-0-UI | Regulator of G-protein signalling 10 | BF544372 |
| 3587 | UI-R-BT0-qc-f-04-0-UI | T-complex associated-testis-expressed 1-like (Protein 91/23) | AI145967 |
| 3588 | UI-R-A0-ao-c-05-0-UI | 3-oxoacid CoA transferase 1 | AA819092 |
| 3589 | UI-R-A0-bb-f-02-0-UI | 9530008L14Rik protein | AA858758 |
| 3590 | UI-R-E0-bo-d-05-0-UI | mKIAA0863 protein | AA858509 |
| 3591 | UI-R-E0-bt-e-12-0-UI | Zinc finger, DHHC domain containing 8 | BF547736 |
| 3592 | UI-R-E0-cy-f-04-0-UI | DIP13 alpha | AA899599 |
| 3593 | UI-R-E0-dg-a-08-0-UI | Vascular endothelial growth factor C | BF558537 |
| 3594 | UI-R-A1-do-c-01-0-UI | Poliovirus receptor-related 3 | BF558608 |
| 3595 | UI-R-A1-ds-a-12-0-UI | RIKEN cDNA 1500016H10 | BF558736 |
| 3596 | UI-R-A1-ev-g-10-0-UI | XP_001101752.1 immunoglobulin kappa constant | AA926189 |
| 3597 | UI-R-E1-fh-g-08-0-UI | RIKEN cDNA 5830446M03 | BF556432 |
| 3598 | UI-R-CU0-bvh-d-03-0-UI | RIKEN cDNA 1110005A03 | BG377972 |
| 3599 | UI-R-CW0s-ccb-g-11-0-UI | Histone aminotransferase 1 | BI285581 |
| 3600 | UI-R-E1-gg-d-02-0-UI | XP_001100384.1 ring finger protein 138 isoform 1 isoform 3 | BF557494 |
| 3601 | UI-R-C0-hn-b-11-0-UI | Transcribed locus | AI137966 |
| 3602 | UI-R-C0-in-h-07-0-UI | Spermine oxidase | AA999064 |
| 3603 | UI-R-C1-kq-e-07-0-UI | XP_001116098.1 TPR domain, ankyrin-repeat and coiled-coil-containing | BF523583 |
| 3604 | UI-R-C2p-rd-a-03-0-UI | Polymerase (DNA-directed), delta interacting protein 3 | AI556134 |
| 3605 | UI-R-C2p-rt-b-07-0-UI | Oncostatin M receptor | AI501164 |
| 3606 | UI-R-C0-ir-d-11-0-UI | Tyrosyl-tRNA synthetase | AI029364 |
| 3607 | UI-R-C0-ix-g-02-0-UI | HECT domain containing 1 | AI029335 |
| 3608 | UI-R-C1-km-b-03-0-UI | Lines homolog 2 | AI045923 |
| 3609 | UI-R-C1-ku-d-06-0-UI | KIAA1712 protein | AI071454 |
| 3610 | UI-R-C1-lj-d-06-0-UI | Chymotrypsin C (caldecrin) | BF549571 |
| 3611 | UI-R-C1-ln-c-11-0-UI | CG9643-PA | AI070033 |
| 3612 | UI-R-C0-hb-h-02-0-UI | Eph receptor B6 | BF557722 |
| 3613 | UI-R-C0-ir-h-07-0-UI | Ubiquitin-activating enzyme E1-domain containing 1 | AI029378 |
| 3614 | UI-R-C1-kp-a-09-0-UI | Aspartoacylase (aminoacylase) 3 | AI058641 |
| 3615 | UI-R-Y0-lt-d-10-0-UI | programmed cell death 10 | AI070383 |
| 3616 | UI-R-C0-hl-d-11-0-UI | Zinc finger protein 36, C3H type-like 2 | BF561280 |
| 3617 | UI-R-C0-jb-d-01-0-UI | Histone deacetylase 5 | AI030290 |
| 3618 | UI-R-C1-ka-e-07-0-UI | FBJ murine osteosarcoma viral oncogene homolog | BF547581 |
| 3619 | UI-R-C1-lq-f-03-0-UI | Ectonucleoside triphosphate diphosphohydrolase 5 | BF552313 |
| 3620 | UI-R-A1-ev-f-01-0-UI | PR-domain zinc finger protein 6 | CK840125 |
| 3621 | UI-R-E1-fr-e-06-0-UI | XP_001079320.1 mondoA isoform 1 | AA956761 |
| 3622 | UI-R-E1-fw-f-01-0-UI | Mitochondrial ribosomal protein L48 | AA957685 |
| 3623 | UI-R-E1-gj-f-01-0-UI | phospholipid scramblase 2 | AA963454 |
| 3624 | UI-R-C0-ih-c-06-0-UI | A disintegrin and metalloprotease domain 8 | AA998698 |
| 3625 | UI-R-C0-in-a-12-0-UI | XP_001104796.1 ring finger and WD repeat domain 2 isoform a isoform 2 | AA999035 |
| 3626 | UI-R-A1-es-c-09-0-UI | Acyl-CoA synthetase long-chain family member 1 | AA926010 |
| 3627 | UI-R-A1-ex-h-09-0-UI | Adenylate cyclase 3 | AA955508 |
| 3628 | UI-R-C0-io-h-02-0-UI | Cysteine and glycine-rich protein 2 | AI029014 |
| 3629 | UI-R-Y0-lt-d-01-0-UI | Growth arrest and DNA-damage-inducible 45 alpha | AI070295 |
| 3630 | UI-R-C2p-nt-a-12-0-UI | Eukaryotic translation initiation factor 2B, subunit 4 delta | AI113226 |
| 3631 | UI-R-A0-bi-g-05-0-UI | Napsin A aspartic peptidase | AA858922 |
| 3632 | UI-R-C1-ll-h-02-0-UI | Synaptotagmin III | BF559906 |
| 3633 | UI-R-A0-ai-a-05-0-UI | Sulfotransferase family 2A, dehydroepiandrosterone (DHEA)-preferring, member 1 | AA818024 |
| 3634 | UI-R-E0-ca-f-11-0-UI | Cytochrome c oxidase, subunit VIc | AA859963 |
| 3635 | UI-R-A1-ev-c-08-0-UI | Transcribed locus |  |
| 3636 | UI-R-C2-ng-b-10-0-UI | AarF domain containing kinase 2 | AI072314 |
| 3637 | UI-R-C2-ni-c-03-0-UI | Hypothetical protein LOC311254 | AI072522 |
| 3638 | UI-R-C2-nk-b-09-0-UI | F-box only protein 7 | AI072666 |
| 3639 | UI-R-C2-nm-f-10-0-UI | Fragile X mental retardation 1 neighbor | BF552839 |
| 3640 | UI-R-A0-aj-f-07-0-UI | Retinol dehydrogenase 7 | AA866390 |
| 3641 | UI-R-A0-aw-a-04-0-UI | Golgi reassembly stacking protein 1 | AA818617 |
| 3642 | UI-R-A0-bd-a-07-0-UI | 5' nucleotidase, ecto | AA858866 |
| 3643 | UI-R-A0-bk-h-01-0-UI | Syndecan 4 | AA819385 |
| 3644 | UI-R-E0-cs-d-11-0-UI | Guanine nucleotide binding protein, alpha inhibiting 2 | AA875418 |
| 3645 | UI-R-E0-dd-e-01-0-UI | Transcription elongation factor B (SIII), polypeptide 3 | BF555648 |
| 3646 | UI-R-C0-jg-h-02-0-UI | Growth arrest specific 7 | AI045701 |
| 3647 | UI-R-C0-jl-a-08-0-UI | Unknown mRNA sequence | BF550566 |
| 3648 | UI-R-C1-ke-b-05-0-UI | R31449_3 | BF546468 |
| 3649 | UI-R-C1-kg-f-06-0-UI | XP_001089482.1 secernin 3 isoform 4 | AI045132 |
| 3650 | UI-R-C1-kj-e-10-0-UI | Ataxia telangiectasia mutated homolog | AI045502 |
| 3651 | UI-R-C1-kl-a-11-0-UI | G protein-coupled receptor 43 | AI058283 |
| 3652 | UI-R-C1-lc-c-03-0-UI | Signal-transducing adaptor protein-2 | BF558502 |
| 3653 | UI-R-C1-lf-d-03-0-UI | solute carrier family 25, member 36 | AI059325 |
| 3654 | UI-R-C1-lp-c-01-0-UI | 2410001C21Rik protein | AI069986 |
| 3655 | UI-R-Y0-lu-h-12-0-UI | DNA-damage-inducible transcript 4-like | BF551915 |
| 3656 | UI-R-A1-ez-c-04-0-UI | splicing factor, arginine/serine-rich 2, interacting protein | AA955140 |
| 3657 | UI-R-E1-ff-f-05-0-UI | Tubulin, gamma 1 | AA955858 |
| 3658 | UI-R-E1-fk-a-02-0-UI | Core-binding factor, runt domain, alpha subunit 2; translocated to, 2 | AA956558 |
| 3659 | UI-R-E1-fm-e-05-0-UI | Non-POU domain containing, octamer-binding | AI113095 |
| 3660 | UI-R-C0-hd-a-05-0-UI | Vacuolar ATP synthase subunit S1 precursor (V-ATPase S1 subunit) | AA965247 |
| 3661 | UI-R-C0-he-h-11-0-UI | Transcribed locus | AA996440 |
| 3662 | UI-R-C0-hj-c-06-0-UI | GrpE-like 1, mitochondrial | AA996644 |
| 3663 | UI-R-C0-hv-f-01-0-UI | Transcribed locus | BF560580 |
| 3664 | UI-R-C0-iu-f-10-0-UI | Transmembrane protein 33 | BF552634 |
| 3665 | UI-R-C0-ja-a-05-0-UI | Leucine rich repeat containing 28 | AI030006 |
| 3666 | UI-R-C0-hv-h-07-0-UI | Neogenin | AA997838 |
| 3667 | UI-R-C0-jc-e-01-0-UI | Guanine nucleotide binding protein, alpha inhibiting 3 | AI030489 |
| 3668 | UI-R-C1-kk-d-03-0-UI | Annexin A6 | AI044904 |
| 3669 | UI-R-C1-ks-a-11-0-UI | PDGFA associated protein 1 | AI059042 |
| 3670 | UI-R-C1-kz-h-08-0-UI | Fatty acid binding protein 3 | AI060068 |
| 3671 | UI-R-C1-lh-e-05-0-UI | Folate hydrolase | AI059788 |
| 3672 | UI-R-C2p-ny-e-09-0-UI | Propionyl coenzyme A carboxylase, beta polypeptide | AI136137 |
| 3673 | UI-R-C2p-ol-a-08-0-UI | Peroxisomal biogenesis factor 6 | AI137451 |
| 3674 | UI-R-A0-ac-c-04-0-UI | Noggin | BF548658 |
| 3675 | UI-R-A0-ah-a-08-0-UI | Choline/ethanolamine phosphotransferase 1 | BF549228 |
| 3676 | UI-R-CV2-cie-b-10-0-UI | Ubiquitin specific protease 8 | BI300369 |
| 3677 | UI-R-DN0-cit-e-21-0-UI | Rab9 effector protein with kelch motifs | BI301464 |
| 3678 | UI-R-DN0-civ-m-02-0-UI | Chromatin modifying protein 5 | BI292286 |
| 3679 | UI-R-DO0-cix-n-12-0-UI | Kinectin 1 | BI302709 |
| 3680 | UI-R-A1-ea-f-06-0-UI | Cytochrome P450, family 1, subfamily a, polypeptide 2 | AA924594 |
| 3681 | UI-R-A1-ee-b-07-0-UI | XP_001118634.1 insulin-like growth factor binding protein, acid labile subunit | BF556234 |
| 3682 | UI-R-A1-eh-h-08-0-UI | Sodium channel, voltage-gated, type VII, alpha | BF550437 |
| 3683 | UI-R-A1-en-h-02-0-UI | Multiple PDZ domain protein | AA925078 |
| 3684 | UI-R-C0-hl-d-02-0-UI | P21 (CDKN1A)-activated kinase 3 | AA997367 |
| 3685 | UI-R-C0-hq-f-11-0-UI | Cortexin | BF551293 |
| 3686 | UI-R-CU0s-cbw-d-01-0-UI | Activating transcription factor 4 | BI282081 |
| 3687 | UI-R-CW0s-ccb-b-06-0-UI | Hypertrophic agonist responsive protein B64 | BI285524 |
| 3688 | UI-R-DK0-cdq-d-05-0-UI | Pyruvate dehydrogenase phosphatase isoenzyme 2 | BI293430 |
| 3689 | UI-R-DK0-cea-d-06-0-UI | mucin 7, salivary | BI294245 |
| 3690 | UI-R-DK0-ced-e-10-0-UI | Carbonic anhydrase 8 | BI294910 |
| 3691 | UI-R-DK0-ceg-a-12-0-UI | Laminin, alpha 3 | BI295125 |
| 3692 | UI-R-BJ2-bqw-a-10-0-UI | Fucokinase | CK843876 |
| 3693 | UI-R-BJ2-brc-e-04-0-UI | Protein phosphatase 2A, regulatory subunit B (PR 53) | BF408416 |
| 3694 | UI-R-DK0-cfh-h-09-0-UI | RIKEN cDNA 3110001D03 | BI289510 |
| 3695 | UI-R-DK0-cfr-a-02-0-UI | Inosine triphosphatase (nucleoside triphosphate pyrophosphatase) | BI290027 |
| 3696 | UI-R-BJ2-bow-a-12-0-UI | Translocase of outer mitochondrial membrane 70 homolog A | CK843056 |
| 3697 | UI-R-BJ2-bpk-e-10-0-UI | Transcribed locus |  |
| 3698 | UI-R-BJ2-bpr-e-10-0-UI | Holocytochrome c synthetase | BF407092 |
| 3699 | UI-R-BJ2-bqf-g-10-0-UI | Ring finger and FYVE like domain containing protein | BF418455 |
| 3700 | UI-R-CX0-bwv-f-08-0-UI | Cdig2 protein | BI276552 |
| 3701 | UI-R-CX0-bxi-a-12-0-UI | Phosphatidylinositol-4-phosphate 5-kinase, type II, gamma | BI275922 |
| 3702 | UI-R-CW0-bxo-d-01-0-UI | hypothetical protein BC002942 | BI276947 |
| 3703 | UI-R-CY0-bxw-h-09-0-UI | Prominin 2 | BI277991 |
| 3704 | UI-R-DE0-cal-h-06-0-UI | Inositol polyphosphate-5-phosphatase D | BI286504 |
| 3705 | UI-R-CS0s-cbn-c-03-0-UI | High mobility group nucleosomal binding domain 2 | BI287298 |
| 3706 | UI-R-CA1-big-a-05-0-UI | Amphiphysin 1 | BF404492 |
| 3707 | UI-R-CA1-biw-k-11-0-UI | Zinc finger protein 291 | BF405311 |
| 3708 | UI-R-CV1-bsk-g-03-0-UI | Tubulin, beta 2b | BG373863 |
| 3709 | UI-R-CV1-bsz-a-08-0-UI | G protein-coupled receptor kinase 1 | BG375319 |
| 3710 | UI-R-CS0-bts-b-12-0-UI | Integrin, beta 5 | BG375999 |
| 3711 | UI-R-CT0-btz-c-02-0-UI | hypothetical protein MGC14151 | BG380601 |
| 3712 | UI-R-CV1-bvu-a-10-0-UI | Leucine-rich repeat-containing G protein-coupled receptor 4 | BG378889 |
| 3713 | UI-R-BT1-bkn-g-09-0-UI | Sperm mitochondria-associated cysteine-rich protein | BF409248 |
| 3714 | UI-R-CN0-blc-f-07-0-UI | Oncomodulin | BF417236 |
| 3715 | UI-R-CN0-blp-a-08-0-UI | Complement receptor related protein | BF417517 |
| 3716 | UI-R-BS1-aza-f-10-0-UI | Neuroblastoma ras oncogene | BE108986 |
| 3717 | UI-R-BS1-azk-h-10-0-UI | Brain zinc finger protein | BE118378 |
| 3718 | UI-R-CA0-azy-f-02-0-UI | Protein tyrosine phosphatase, receptor type, f polypeptide (PTPRF), interacting protein (liprin), alpha 4 | BE119109 |
| 3719 | UI-R-CA0-bao-d-02-0-UI | FERM and PDZ domain containing 1 | BE119991 |
| 3720 | UI-R-CA1-bcw-e-11-0-UI | Spastic paraplegia 3A homolog | BF391624 |
| 3721 | UI-R-BS2-bdh-g-07-0-UI | Pseudouridylate synthase 3 | BF389095 |
| 3722 | UI-R-BS2-bdv-b-09-0-UI | Retinoic acid induced 1 | BF396211 |
| 3723 | UI-R-BS2-beb-e-07-0-UI | antigenic determinant of rec-A protein | BF396990 |
| 3724 | UI-R-CA0-bha-d-01-0-UI | Stonin 2 | BF394150 |
| 3725 | UI-R-CA0-bho-g-01-0-UI | BM88 antigen | BF402711 |
| 3726 | UI-R-BT1-aqm-g-10-0-UI | Odd Oz/ten-m homolog 2 | BE102452 |
| 3727 | UI-R-BX0-arc-a-07-0-UI | Probable phospholipid-transporting ATPase ID (ATPase class I type 8B member 2) | BE103434 |
| 3728 | UI-R-BJ1-ato-a-07-0-UI | Forkhead box J3 | BE099314 |
| 3729 | UI-R-BJ1-ats-b-05-0-UI | timeless-interacting protein | BE100135 |
| 3730 | UI-R-BJ1-aua-a-05-0-UI | Sulfite oxidase | CK839426 |
| 3731 | UI-R-BJ1-auh-e-12-0-UI | Calcium binding protein 39 | BE101142 |
| 3732 | UI-R-BJ1-awa-e-10-0-UI | Transcribed locus | BE112936 |
| 3733 | UI-R-BJ1-awf-a-12-0-UI | XP_001072079.1 LSM16 homolog (EDC3, S. cerevisiae) isoform 1 | BE112984 |
| 3734 | UI-R-BJ1-awp-d-02-0-UI | XP_001095030.1 zinc finger protein 710 | CK839997 |
| 3735 | UI-R-CA0-awy-a-05-0-UI | Neuropathy target esterase like 1 | BE107890 |
| 3736 | UI-R-BO1-aja-d-10-0-UI | Thyrotropin releasing hormone receptor 2 | AW526300 |
| 3737 | UI-R-BO1-aji-a-02-0-UI | RIKEN cDNA 3110007P09 | BF563265 |
| 3738 | UI-R-BO1-ajl-e-03-0-UI | Contactin 2 | AW528446 |
| 3739 | UI-R-BO1-ajs-a-05-0-UI | Neuropilin (NRP) and tolloid (TLL)-like 2 | AW527233 |
| 3740 | UI-R-C4-alb-h-11-0-UI | Splicing factor 4 | AW530829 |
| 3741 | UI-R-C4-alk-d-07-0-UI | Endothelial-specific receptor tyrosine kinase | BF564227 |
| 3742 | UI-R-C4-alq-h-03-0-UI | Gap junction membrane channel protein alpha 4 | AW534233 |
| 3743 | UI-R-BT1-ame-d-04-0-UI | XP_001086601.1 membrane-spanning 4-domains, subfamily A, member 5 isoform 2 | BE107659 |
| 3744 | UI-R-BU0-apk-b-09-0-UI | Solute carrier family 16, member 8 | BE096000 |
| 3745 | UI-R-BO1-aqb-e-11-0-UI | Beta-1,3-glucuronyltransferase 1 (glucuronosyltransferase P) | BE097773 |
| 3746 | UI-R-AA1-aac-a-09-0-UI | PHD finger protein 5A | CK845092 |
| 3747 | UI-R-AF1-aar-d-10-0-UI | coiled-coil domain containing 8 | BF543098 |
| 3748 | UI-R-Y0-acm-f-01-0-UI | Solute carrier family 25 (mitochondrial carrier, glutamate), member 22 | AI717223 |
| 3749 | UI-R-Y0-acx-a-08-0-UI | Integrin beta 2 | AI764280 |
| 3750 | UI-R-BJ0-ado-b-03-0-UI | Netrin 1 | AW251519 |
| 3751 | UI-R-BJ0-aej-g-03-0-UI | Prohibitin 2 | AW254559 |
| 3752 | UI-R-BJ0p-agh-h-12-0-UI | Transcription factor 8 | AW520827 |
| 3753 | UI-R-BO0-agq-d-10-0-UI | Dihydropyrimidine dehydrogenase | BF567565 |
| 3754 | UI-R-BO0-ahk-d-09-0-UI | Secretogranin III | BF567748 |
| 3755 | UI-R-BO0-ahs-e-08-0-UI | RAB11 family interacting protein 2 (class I) | BF567218 |
| 3756 | UI-R-C3-st-c-03-0-UI | Nucleolar protein 9 | AI535235 |
| 3757 | UI-R-C3-th-c-01-0-UI | Testin gene | BF522511 |
| 3758 | UI-R-C3-tm-g-08-0-UI | RIKEN cDNA 4833435D08 | AI710041 |
| 3759 | UI-R-C3-tu-e-07-0-UI | Aquaporin 2 | AI549497 |
| 3760 | UI-R-AD0-vz-b-06-0-UI | Mitochondrial ribosomal protein S25 | AI602088 |
| 3761 | UI-R-AA0-wj-c-10-0-UI | Glutathione peroxidase 3 | CK844662 |
| 3762 | UI-R-AG0-wx-a-04-0-UI | hypothetical protein MGC19604 | CK838602 |
| 3763 | UI-R-AE0-xh-d-10-0-UI | Thioredoxin-like 5 | CK844765 |
| 3764 | UI-R-AD1-zm-f-04-0-UI | Hect (homologous to the E6-AP (UBE3A) carboxyl terminus) domain and RCC1 (CHC1)-like domain (RLD) 2 | BF524882 |
| 3765 | UI-R-AD1-zr-g-09-0-UI | Cofactor required for Sp1 transcriptional activation, subunit 3 | CK841018 |
| 3766 | UI-R-C0-ht-e-02-0-UI | V-raf murine sarcoma 3611 viral oncogene homolog | BF524093 |
| 3767 | UI-R-C0-im-e-02-0-UI | Lymphocyte cytosolic protein 2 | AA998926 |
| 3768 | UI-R-Y0-mh-e-09-0-UI | NADH dehydrogenase (ubiquinone) 1 beta subcomplex, 2 | AI112237 |
| 3769 | UI-R-C2-mt-f-04-0-UI | Exocyst complex component 4 | BF549457 |
| 3770 | UI-R-C2p-nq-e-01-0-UI | Development and differentiation enhancing | CN540015 |
| 3771 | UI-R-C2p-oc-e-09-0-UI | Choline phosphotransferase 1 | BF522250 |
| 3772 | UI-R-BT0-qk-b-08-0-UI | Transcribed locus |  |
| 3773 | UI-R-C2p-qq-a-09-0-UI | Myoferlin (Fer-1 like protein 3) | AI555446 |
| 3774 | UI-R-C2p-qu-f-12-0-UI | Serine (or cysteine) proteinase inhibitor, clade B, member 1a | AI555228 |
| 3775 | UI-R-C2p-re-h-05-0-UI | Leucocyte specific transcript 1 | AI555859 |
| 3776 | UI-R-E0-bx-b-04-0-UI | Protein tyrosine phosphatase, receptor type, f polypeptide (PTPRF), interacting protein, alpha 1 | BF549963 |
| 3777 | UI-R-E0-cc-e-02-0-UI | RuvB-like 2 | AA859809 |
| 3778 | UI-R-E0-ck-d-08-0-UI | Lipopolysaccharide binding protein | BF554954 |
| 3779 | UI-R-E0-cr-h-08-0-UI | Mitochondrial ribosomal protein L41 | BF555153 |
| 3780 | UI-R-A1-dw-g-04-0-UI | LSM3 homolog, U6 small nuclear RNA associated | AA901259 |
| 3781 | UI-R-A1-eb-g-10-0-UI | Cardiac ankyrin repeat kinase | CK845755 |
| 3782 | UI-R-A1-eg-h-02-0-UI | Coilin | BF555770 |
| 3783 | UI-R-A1-el-b-12-0-UI | Protease, serine, 25 | AA926045 |
| 3784 | UI-R-C0-gy-c-12-0-UI | Yip1 interacting factor homolog B | AA964285 |
| 3785 | UI-R-C0-ho-b-06-0-UI | N-acylsphingosine amidohydrolase (acid ceramidase)-like | AI137596 |
| 3786 | UI-R-C2p-nv-c-06-0-UI | Heterogeneous nuclear ribonucleoprotein A3 | AI136231 |
| 3787 | UI-R-C3-sy-e-02-0-UI | Interferon-induced protein with tetratricopeptide repeats 3 | BF543460 |
| 3788 | UI-R-BO0-agl-a-09-0-UI | Host cell factor C2 | AW521309 |
| 3789 | UI-R-BJ1-aur-a-04-0-UI | NADH dehydrogenase (ubiquinone) Fe-S protein 7 | BE109352 |
| 3790 | UI-R-A0-ay-c-02-0-UI | Fibroblast growth factor receptor substrate 3 | BF549739 |
| 3791 | UI-R-E0-ci-h-01-0-UI | Transcribed locus | AI454336 |
| 3792 | UI-R-A1-dp-b-05-0-UI | Ring finger protein 3 | CK845721 |
| 3793 | UI-R-A1-ek-c-06-0-UI | hypothetical protein FLJ13910 | BF521625 |
| 3794 | UI-R-C1-lk-h-10-0-UI | RNA binding motif protein 22 | AI502355 |
| 3795 | UI-R-C2p-nr-g-02-0-UI | DCN1, defective in cullin neddylation 1, domain containing 3 | BF522194 |
| 3796 | UI-R-C0-ha-f-08-0-UI | CD300A antigen | BF556758 |
| 3797 | UI-R-C0-hf-g-02-0-UI | B-cell leukemia/lymphoma 3 | AA996726 |
| 3798 | UI-R-C0-jc-h-12-0-UI | N-myc (and STAT) interactor | BF560702 |
| 3799 | UI-R-C0-jm-d-03-0-UI | Elongation factor RNA polymerase II 2 | BF550577 |
| 3800 | UI-R-C0-jr-b-01-0-UI | Chromobox homolog 5 (Drosophila HP1a) | BF545282 |
| 3801 | UI-R-C1-ju-e-01-0-UI | Blood vessel epicardial substance | AI045288 |
| 3802 | UI-R-A0-ar-e-06-0-UI | Sushi-repeat-containing protein | AA818824 |
| 3803 | UI-R-E1-fc-b-03-0-UI | Palmitoyl-protein thioesterase 2 | AA955698 |
| 3804 | UI-R-BT0-py-e-10-0-UI | 3-hydroxybutyrate dehydrogenase, type 1 | AI145769 |
| 3805 | UI-R-A0-aw-b-03-0-UI | Insulin induced gene 2 | AA818627 |
| 3806 | UI-R-E1-fg-e-12-0-UI | Survival of motor neuron 1, telomeric | AA955755 |
| 3807 | UI-R-C0-iv-c-11-0-UI | Orosomucoid 1 | BF552651 |
| 3808 | UI-R-A0-ba-d-06-0-UI | Protein transport protein SEC61 gamma subunit | AA819150 |
| 3809 | UI-R-E1-fw-c-05-0-UI | Hypothetical protein MGC:72616 | AA957475 |
| 3810 | UI-R-C2-mq-h-08-0-UI | B-cell leukemia/lymphoma 2 related protein A1 | AI070487 |
| 3811 | UI-R-E1-ft-b-10-0-UI | Histone 2a | AA956818 |
| 3812 | UI-R-A0-aw-e-01-0-UI | MUS81 endonuclease homolog | CK845700 |
| 3813 | UI-R-A0-az-f-11-0-UI | IlvB (bacterial acetolactate synthase)-like | AA818650 |
| 3814 | UI-R-E1-gk-h-02-0-UI | LIM domains containing 1 | BF559872 |
| 3815 | UI-R-C0-gv-c-09-0-UI | Bleomycin hydrolase | BF557302 |
| 3816 | UI-R-E0-cb-b-11-0-UI | Heat shock protein, alpha-crystallin-related, B6 | AA875007 |
| 3817 | UI-R-E0-cg-h-08-0-UI | Matrix Gla protein | AA874853 |
| 3818 | UI-R-E1-fa-h-03-0-UI | Cytochrome c oxidase subunit Vb | AA955550 |
| 3819 | UI-R-E1-fm-e-04-0-UI | Malic enzyme 1 | AA956687 |
| 3820 | UI-R-E1-fv-e-06-0-UI | Transmembrane emp24-like trafficking protein 10 | AA957764 |
| 3821 | UI-R-C0-hz-c-04-0-UI | Vesicle-associated membrane protein 2 | BF561458 |
| 3822 | UI-R-A0-ba-g-07-0-UI | Mitochondrial ribosomal protein L23 | AA819050 |
| 3823 | UI-R-E1-fx-d-10-0-UI | Rhesus blood group-associated A glycoprotein | BF556584 |
| 3824 | UI-R-C0-hc-a-09-0-UI | Pyrophosphatase | AA965166 |
| 3825 | UI-R-C0-jd-e-03-0-UI | Fos-like antigen 2 | BF546918 |
| 3826 | UI-R-Y0-lv-f-05-0-UI | Receptor accessory protein 6 | BF521767 |
| 3827 | UI-R-C2-mw-c-07-0-UI | Procollagen, type X, alpha 1 | AI071616 |
| 3828 | UI-R-C2-na-g-07-0-UI | Synapse associated protein 1 | AI070997 |
| 3829 | UI-R-C2-nc-g-07-0-UI | ATPase, (Na+)/K+ transporting, beta 4 polypeptide | AI071721 |
| 3830 | UI-R-C2-nn-e-05-0-UI | Triple functional domain (PTPRF interacting) | AI072616 |
| 3831 | UI-R-C2p-nq-g-11-0-UI | nuclear cap binding protein subunit 2 | AI136699 |
| 3832 | UI-R-C2p-nw-b-06-0-UI | DEAD (Asp-Glu-Ala-Asp) box polypeptide 21a | AI136551 |
| 3833 | UI-R-C2p-ny-d-09-0-UI | Tumor necrosis factor, alpha-induced protein 2 (Primary response gene B94 protein) | AI136126 |
| 3834 | UI-R-A0-bn-f-11-0-UI | Nuclear factor of kappa light chain gene enhancer in B-cells 1, p105 | AA858801 |
| 3835 | UI-R-E0-bv-b-09-0-UI | Thioredoxin interacting protein | AA859566 |
| 3836 | UI-R-C0-ie-g-07-0-UI | Cysteine-rich with EGF-like domains 1 | AA998502 |
| 3837 | UI-R-C0-ij-e-10-0-UI | FXYD domain-containing ion transport regulator 7 | AI028815 |
| 3838 | UI-R-C0-jp-a-01-0-UI | Ectonucleoside triphosphate diphosphohydrolase 1 | BF554695 |
| 3839 | UI-R-C1-jw-f-08-0-UI | Olfactomedin-like 2B | AI044491 |
| 3840 | UI-R-C1-jy-a-12-0-UI | Protein C14orf102 homolog | AI045709 |
| 3841 | UI-R-C1-jz-c-03-0-UI | G protein-coupled receptor 85 | BF545606 |
| 3842 | UI-R-C1-kp-h-02-0-UI | Homeo box C8 | BF554389 |
| 3843 | UI-R-C1-kr-c-09-0-UI | Ubiquitination factor E4B, UFD2 homolog | BF554423 |
| 3844 | UI-R-C1-kt-f-04-0-UI | hypothetical protein DKFZp566N034 | AI058796 |
| 3845 | UI-R-C1-ky-b-07-0-UI | Cd27 binding protein (Hindu God of destruction) | BF546395 |
| 3846 | UI-R-A0-aj-d-04-0-UI | Nuclear receptor subfamily 1, group D, member 2 | AA858968 |
| 3847 | UI-R-A0-am-a-09-0-UI | Aldehyde dehydrogenase 8 family, member A1 | BF549284 |
| 3848 | UI-R-A0-aq-e-09-0-UI | Receptor transporter protein 4 | AA819788 |
| 3849 | UI-R-A0-au-g-11-0-UI | cDNA sequence AF155546 | BF548312 |
| 3850 | UI-R-E1-fn-c-08-0-UI | Nin one binding protein | AA956531 |
| 3851 | UI-R-E1-fq-d-04-0-UI | Myosin binding protein C, slow type | AA956999 |
| 3852 | UI-R-E1-fu-g-03-0-UI | Homocysteine-inducible, endoplasmic reticulum stress-inducible, ubiquitin-like domain member 1 | AA957323 |
| 3853 | UI-R-E1-gq-g-06-0-UI | Claudin domain containing 1 | AA964787 |
| 3854 | UI-R-C0-hy-h-11-0-UI | Cadherin 5 | AA997619 |
| 3855 | UI-R-C0-ib-h-09-0-UI | FK506 binding protein 2 | BF561706 |
| 3856 | UI-R-C0-gv-g-09-0-UI | Myosin 5B | AA964525 |
| 3857 | UI-R-C0-gz-h-01-0-UI | Serine (or cysteine) peptidase inhibitor, clade E, member 1 | BF556719 |
| 3858 | UI-R-C0-jg-d-07-0-UI | S100 calcium binding protein G | AI029652 |
| 3859 | UI-R-C0-jn-h-12-0-UI | Zinc finger protein 148 | AI030160 |
| 3860 | UI-R-C1-ju-b-01-0-UI | Procollagen-lysine, 2-oxoglutarate 5-dioxygenase 1 | AI045272 |
| 3861 | UI-R-C1-kb-a-06-0-UI | Mucin 13, epithelial transmembrane | BF550919 |
| 3862 | UI-R-C1-lk-f-09-0-UI | Luteinizing hormone/choriogonadotropin receptor | BF546768 |
| 3863 | UI-R-Y0-ls-c-02-0-UI | Renin binding protein | AI070075 |
| 3864 | UI-R-C2-my-f-09-0-UI | Fanconi anemia, complementation group C | AI071218 |
| 3865 | UI-R-C2-ng-e-10-0-UI | Rabphilin 3A-like (without C2 domains) | BF549655 |
| 3866 | UI-R-DK0-cfv-a-04-0-UI | phosphatidylinositol-3-phosphate/phosphatidylinositol 5-kinase, type III isoform 2 | BI290334 |
| 3867 | UI-R-DK0-cga-b-03-0-UI | protein kinase, lysine deficient 1; kinase deficient protein | BI290763 |
| 3868 | UI-R-DK0-cgi-h-03-0-UI | Tetratricopeptide repeat domain 8 | BI290899 |
| 3869 | UI-R-CV2-cgs-g-08-0-UI | Cadherin 22 | BI296578 |
| 3870 | UI-R-DQ0-cja-c-24-0-UI | COMM domain containing 9 | BI303453 |
| 3871 | UI-R-E0-dk-d-06-0-UI | Branched chain aminotransferase 1, cytosolic | AA900881 |
| 3872 | UI-R-A1-dq-e-01-0-UI | Aldo-keto reductase family 7, member A3 (aflatoxin aldehyde reductase) | BF558673 |
| 3873 | UI-R-A1-du-d-02-0-UI | SUB1 homolog | BF555679 |
| 3874 | UI-R-E1-gf-c-09-0-UI | Cytochrome P450, family 4, subfamily b, polypeptide 1 | AA957658 |
| 3875 | UI-R-E1-go-d-01-0-UI | Acetyl-Coenzyme A acyltransferase 2 (mitochondrial 3-oxoacyl-Coenzyme A thiolase) | BF557089 |
| 3876 | UI-R-DB0-byx-h-02-0-UI | Fucosyltransferase 4 | BI285246 |
| 3877 | UI-R-DD0-bzs-b-10-0-UI | Growth factor receptor bound protein 7 | BI283190 |
| 3878 | UI-R-CW0s-ccc-g-05-0-UI | Profilin 1 | BI282907 |
| 3879 | UI-R-CX0s-ccn-h-05-0-UI | Low-density lipoprotein receptor-related protein 10 | BI284245 |
| 3880 | UI-R-CX0s-ccs-b-11-0-UI | CG9646-PA | BI284785 |
| 3881 | UI-R-DK0-cda-e-11-0-UI | Transcription elongation factor A (SII), 2 | BI288410 |
| 3882 | UI-R-DK0-cej-h-05-0-UI | Angiopoietin 4 | BI294141 |
| 3883 | UI-R-DK0-cey-c-07-0-UI | Glycosyltransferase-like 1B | BI296264 |
| 3884 | UI-R-DK0-cfe-a-03-0-UI | XP_001106148.1 B0432.8 | BI289054 |
| 3885 | UI-R-CM0-bjh-f-09-0-UI | Proenkephalin 1 | BF395740 |
| 3886 | UI-R-CN0-blx-c-12-0-UI | Transcribed locus |  |
| 3887 | UI-R-BT1-bmt-d-03-0-UI | EPM2A (laforin) interacting protein 1 | BF411547 |
| 3888 | UI-R-BT1-bnp-f-02-0-UI | Processing of precursor 1, ribonuclease P/MRP family, | BF412594 |
| 3889 | UI-R-CA0-boi-a-02-0-UI | XP_001107821.1 FK506 binding protein 10, 65 kDa isoform 5 | BF413736 |
| 3890 | UI-R-BJ2-bqh-g-11-0-UI | Peroxisomal biogenesis factor 13 | BF418601 |
| 3891 | UI-R-BJ2-bqo-e-05-0-UI | Tetraspanin 5 | BF419135 |
| 3892 | UI-R-BJ2-bqt-f-09-0-UI | Basophilic leukemia expressed protein BLES03 | BF407558 |
| 3893 | UI-R-CW0-bwf-c-07-0-UI | Tramdorin 1 | BI274126 |
| 3894 | UI-R-CZ0-byf-e-10-0-UI | Lamin A | BI277711 |
| 3895 | UI-R-DA0-bym-a-04-0-UI | Tial1 cytotoxic granule-associated RNA binding protein-like 1 | BI279245 |
| 3896 | UI-R-CA0-bfu-d-06-0-UI | Transforming growth factor, beta receptor II | BF393480 |
| 3897 | UI-R-CA0-bgh-c-08-0-UI | Ankyrin 2, neuronal | BF401072 |
| 3898 | UI-R-CA1-biy-e-06-0-UI | Fat tumor suppressor homolog 2 | BF403321 |
| 3899 | UI-R-CA1-bjd-d-04-0-UI | UDP-N-acteylglucosamine pyrophosphorylase 1-like 1 | BF403568 |
| 3900 | UI-R-CA1-bjf-h-18-0-UI | Phosphoserine aminotransferase 1 | BF406397 |
| 3901 | UI-R-CV1-brv-d-03-0-UI | RAS protein-specific guanine nucleotide-releasing factor 2 | BG372540 |
| 3902 | UI-R-CT0-bub-e-03-0-UI | ATPase, H+ transporting, V1 subunit E isoform 1 | BG380794 |
| 3903 | UI-R-CT0-bun-b-06-0-UI | Interleukin-1 receptor-associated kinase 1 | BG376154 |
| 3904 | UI-R-CU0-buv-a-11-0-UI | Gamma-glutamyltransferase 1 | BG376712 |
| 3905 | UI-R-CU0-bvg-d-11-0-UI | Meprin 1 beta | BG377893 |
| 3906 | UI-R-CA0-axc-f-09-0-UI | Oligosaccharyl transferase 3 CG7748-PA | BE108475 |
| 3907 | UI-R-CA0-axi-a-08-0-UI | RWD domain containing 2 | BE109844 |
| 3908 | UI-R-BS1-axt-c-09-0-UI | Chloride channel K1 | BE115105 |
| 3909 | UI-R-BS1-ayn-c-01-0-UI | src homology 2 domain-containing transforming protein D | BE117304 |
| 3910 | UI-R-CA0-bap-h-01-0-UI | Transcribed locus | BE120211 |
| 3911 | UI-R-CA0-bay-e-09-0-UI | Cocaine and amphetamine regulated transcript | BE120701 |
| 3912 | UI-R-CA1-bbn-c-04-0-UI | WD repeat domain 47 | BF387512 |
| 3913 | UI-R-CA1-bcb-c-03-0-UI | ATP-binding cassette, sub-family A (ABC1), member 8a | BF396093 |
| 3914 | UI-R-BS2-bef-g-06-0-UI | ADP-ribosylation factor-like 6 interacting protein 2 | BF397970 |
| 3915 | UI-R-BS2-bew-e-06-0-UI | Bromodomain adjacent to zinc finger domain, 2B | BF396904 |
| 3916 | UI-R-BU0-anf-a-06-0-UI | Variable coding sequence A1 | AW533278 |
| 3917 | UI-R-BU0-aoz-a-06-0-UI | Arylalkylamine N-acetyltransferase | BE095747 |
| 3918 | UI-R-BX0-ark-c-10-0-UI | RIKEN cDNA 5730434I03 gene | BE104064 |
| 3919 | UI-R-BX0-asf-a-08-0-UI | Procollagen, type XI, alpha 1 | BE104990 |
| 3920 | UI-R-BO1-asq-a-04-0-UI | HLA-B associated transcript 5 | BE106613 |
| 3921 | UI-R-BJ1-asz-d-06-0-UI | Lysosomal-associated protein transmembrane 5 | CK842658 |
| 3922 | UI-R-BJ1-aum-a-02-0-UI | Phosphatidylinositol 3-kinase catalytic delta polypeptide | BE109926 |
| 3923 | UI-R-BJ1-auy-f-10-0-UI | Tumor necrosis factor ligand superfamily member 12 | CK843985 |
| 3924 | UI-R-BJ1-avl-a-07-0-UI | GLIS family zinc finger 2 | BE112211 |
| 3925 | UI-R-BJ1-avq-f-07-0-UI | T-cell lymphoma invasion and metastasis 1 | BE109656 |
| 3926 | UI-R-BO0-ahw-f-11-0-UI | Transient receptor potential cation channel, subfamily C, member 3 | BF567975 |
| 3927 | UI-R-BO0-aid-b-01-0-UI | Protein kinase, AMP-activated, alpha 1 catalytic subunit | BF562723 |
| 3928 | UI-R-BJ0p-aik-a-06-0-UI | RIKEN cDNA 0610037L13 | CK843943 |
| 3929 | UI-R-BJ0p-air-e-07-0-UI | Von Willebrand factor | CK841964 |
| 3930 | UI-R-BO1-aju-d-10-0-UI | ATPase, Ca++ transporting, plasma membrane 3 | BF565336 |
| 3931 | UI-R-BT1-akb-f-08-0-UI | Transcribed locus | AW527920 |
| 3932 | UI-R-BT1-akj-b-06-0-UI | Latrophilin 3 | AW528831 |
| 3933 | UI-R-BT1-ako-f-07-0-UI | XP_001067235.1 ALK tyrosine kinase receptor precursor (Anaplastic lymphoma kinase) (CD246 antigen) | AW528550 |
| 3934 | UI-R-BS0-amp-h-09-0-UI | Snf2-related CBP activator protein | AW532570 |
| 3935 | UI-R-BU0-amz-f-06-0-UI | Neuroligin 2 | AW533081 |
| 3936 | UI-R-AC0-yp-c-07-0-UI | Vezatin, adherens junctions transmembrane protein | CK840796 |
| 3937 | UI-R-AB1-yy-c-01-0-UI | XP_001109210.1 Transcription initiation factor TFIID subunit 10 (Transcription initiation factor TFIID 30 kDa subunit) (TAF(II)30) (TAFII-30) (TAFII30) (STAF28) isoform 2 | CK838947 |
| 3938 | UI-R-AF1-aat-h-08-0-UI | Hepatoma-derived growth factor | CK840657 |
| 3939 | UI-R-Y0-abb-a-10-0-UI | Chondroadherin | BF522077 |
| 3940 | UI-R-Y0-abn-c-02-0-UI | Receptor-associated protein of the synapse | AI715176 |
| 3941 | UI-R-Y0-aca-g-05-0-UI | Mitochondrial ribosomal protein L40 | AI717326 |
| 3942 | UI-R-BJ0p-aex-d-04-0-UI | ubiquitin protein ligase E3 component n-recognin 2 | AW520324 |
| 3943 | UI-R-BJ0p-afg-c-04-0-UI | mKIAA1208 protein | AW434889 |
| 3944 | UI-R-BJ0p-afm-g-12-0-UI | RNA binding motif protein 4 | CK841533 |
| 3945 | UI-R-BJ0p-afv-g-01-0-UI | Phosphoribosyl pyrophosphate synthetase 1 | CK841243 |
| 3946 | UI-R-C2p-ri-f-04-0-UI | CG32425-PA | AI556633 |
| 3947 | UI-R-C2p-rq-c-12-0-UI | RNA (guanine-9-) methyltransferase domain containing 3 | BF546009 |
| 3948 | UI-R-C2p-sa-g-02-0-UI | Transient receptor potential cation channel, subfamily C, member 4 | BF522979 |
| 3949 | UI-R-C3-sg-f-06-0-UI | Chemokine (C-C motif) receptor 1 | AI511231 |
| 3950 | UI-R-C3-tz-d-03-0-UI | Ets variant gene 5 (ets-related molecule) | BF522407 |
| 3951 | UI-R-G0-ug-a-05-0-UI | Neurofilament, heavy polypeptide | AI574937 |
| 3952 | UI-R-G0-ut-e-06-0-UI | Transcribed locus | BF522699 |
| 3953 | UI-R-Y0-vb-d-06-0-UI | Recoverin | AI602128 |
| 3954 | UI-R-AC1-xq-h-03-0-UI | Zinc finger protein 551 (Zinc finger protein KOX23) | CK839004 |
| 3955 | UI-R-AF0-yf-d-07-0-UI | RIKEN cDNA 1110059P08 | CK840567 |
| 3956 | UI-R-E1-fp-f-05-0-UI | RNA binding motif protein 4 | BF556538 |
| 3957 | UI-R-E1-ge-f-07-0-UI | AIP1 | AA957143 |
| 3958 | UI-R-C0-ja-c-05-0-UI | Transcribed locus |  |
| 3959 | UI-R-C1-jt-f-09-0-UI | DnaJ (Hsp40) homolog, subfamily C, member 13 | BF551415 |
| 3960 | UI-R-C1-kn-a-04-0-UI | Coiled-coil domain containing 59 | AI058324 |
| 3961 | UI-R-C1-lh-d-01-0-UI | Fibrinogen, gamma polypeptide | AI059777 |
| 3962 | UI-R-C2p-og-d-10-0-UI | Chemokine-like factor (Cklf), mRNA | AI137363 |
| 3963 | UI-R-BT0-pl-c-08-0-UI | RIKEN cDNA 2310035C23 | AI144632 |
| 3964 | UI-R-BT0-pr-f-10-0-UI | Methylmalonic aciduria (cobalamin deficiency) cblA type | AI145027 |
| 3965 | UI-R-BT0-py-b-01-0-UI | Tryptophan hydroxylase 2 | BF544456 |
| 3966 | UI-R-A0-ad-f-10-0-UI | hypothetical protein FLJ14466 | BF524964 |
| 3967 | UI-R-A0-aw-f-11-0-UI | Erythroid spectrin beta | BF549861 |
| 3968 | UI-R-A0-bk-h-04-0-UI | Transcribed locus | AA819388 |
| 3969 | UI-R-E0-br-a-05-0-UI | Adenosine kinase | BF550480 |
| 3970 | UI-R-E0-cv-g-12-0-UI | HtrA serine peptidase 3 | BF550167 |
| 3971 | UI-R-E0-dd-a-08-0-UI | Complement component 1, q subcomponent, gamma polypeptide | AA900285 |
| 3972 | UI-R-E0-di-b-01-0-UI | cisplatin resistance related protein CRR9p | BF555471 |
| 3973 | UI-R-A1-dp-e-08-0-UI | Ectonucleotide pyrophosphatase/phosphodiesterase 6 | AA901205 |
| 3974 | UI-R-A1-en-e-12-0-UI | Transcribed locus | AA925052 |
| 3975 | UI-R-A1-ey-g-02-0-UI | autoantigen | AA955058 |
| 3976 | UI-R-CA0-bgl-c-08-0-UI | Hydroxysteroid (17-beta) dehydrogenase 13 | BF401498 |
| 3977 | UI-R-BJ2-bqk-d-08-0-UI | THAP domain containing 4 | BF418915 |
| 3978 | UI-R-BJ2-bra-f-01-0-UI | Lymphocyte antigen 6 complex, locus G6C | CK843402 |
| 3979 | UI-R-E1-fw-c-06-0-UI | Transcribed locus |  |
| 3980 | UI-R-E1-gm-g-10-0-UI | XP_001107541.1 SRY-box containing gene 12 | BF556869 |
| 3981 | UI-R-C0-hx-b-09-0-UI | Trypsin V-A | AA998109 |
| 3982 | UI-R-C0-jq-g-12-0-UI | Glutamine repeat protein 1 | AI045829 |
| 3983 | UI-R-C2p-qu-d-02-0-UI | Ofd1 protein | AI554988 |
| 3984 | UI-R-C2p-rl-c-10-0-UI | LOC362136 | BF523032 |
| 3985 | UI-R-C0-ip-b-11-0-UI | Golgi associated, gamma adaptin ear containing, ARF binding protein 2 | AI029117 |
| 3986 | UI-R-C0-iw-b-01-0-UI | Aldehyde dehydrogenase family 7, member A1 | AI029471 |
| 3987 | UI-R-C1-ka-g-05-0-UI | Cytochrome b5 reductase 2 | AI045385 |
| 3988 | UI-R-C1-kn-d-08-0-UI | Reversion induced LIM gene | AI058429 |
| 3989 | UI-R-C1-kw-b-04-0-UI | SUMO/sentrin specific peptidase 3 | BF547080 |
| 3990 | UI-R-C1-ll-f-04-0-UI | Phosphatidylinositol 4-kinase type 2 beta | AI060149 |
| 3991 | UI-R-E1-fs-g-10-0-UI | Gastrulation brain homeobox 2 | AI137760 |
| 3992 | UI-R-C0-ic-a-07-0-UI | TAF2 RNA polymerase II, TATA box binding protein (TBP)-associated factor | BF561708 |
| 3993 | UI-R-C1-js-c-06-0-UI | Zinc finger protein 297 | BF551134 |
| 3994 | UI-R-C1-lj-g-09-0-UI | Caspase 3, apoptosis related cysteine protease | AI059604 |
| 3995 | UI-R-C0-ha-e-05-0-UI | Melanoma antigen, family D, 2 | AA965028 |
| 3996 | UI-R-C0-io-h-05-0-UI | Zinc finger, DHHC domain containing 7 | BF551386 |
| 3997 | UI-R-C0-jq-e-08-0-UI | Vanin 1 | BF550801 |
| 3998 | UI-R-C1-ku-e-02-0-UI | EH-domain containing 3 | AI071456 |
| 3999 | UI-R-A1-eu-c-07-0-UI | transcription factor ONECUT2 | AA926096 |
| 4000 | UI-R-E1-fb-h-04-0-UI | Poly(rC) binding protein 3 | AA956005 |
| 4001 | UI-R-E1-ft-b-08-0-UI | NP_536848.1 synthase F0 subunit 6 | AA956816 |
| 4002 | UI-R-E1-fy-c-08-0-UI | breast cancer membrane protein 101 | AA957367 |
| 4003 | UI-R-C0-hk-h-02-0-UI | CD68 antigen | AA996984 |
| 4004 | UI-R-C0-il-c-07-0-UI | XP_001111073.1 C-type lectin domain family 3, member A | AI029075 |
| 4005 | UI-R-E0-dh-g-02-0-UI | Drebrin 1 | BF555466 |
| 4006 | UI-R-A1-ev-f-02-0-UI | Acetyl-coenzyme A acetyltransferase 1 | AA926170 |
| 4007 | UI-R-C0-il-h-11-0-UI | Amiloride-sensitive cation channel 2, neuronal | AI029105 |
| 4008 | UI-R-C0-iu-g-10-0-UI | Steroid sulfatase | BF551588 |
| 4009 | UI-R-E1-ga-a-01-0-UI | Progesterone receptor membrane component 1 | BF560411 |
| 4010 | UI-R-BT0-pz-a-05-0-UI | XP_001101513.1 ribosomal protein S12 | AI145829 |
| 4011 | UI-R-C1-kn-d-04-0-UI | Developmentally regulated RNA-binding protein 1 | AI058342 |
| 4012 | UI-R-C2-mq-h-07-0-UI | Epimorphin | BF559461 |
| 4013 | UI-R-A0-bk-e-10-0-UI | Cytochrome c oxidase, subunit VIIIa | AA819358 |
| 4014 | UI-R-E0-cs-b-09-0-UI | Procollagen C-endopeptidase enhancer protein | BF555158 |
| 4015 | UI-R-C2-ne-f-07-0-UI | Aquaporin 11 | AI072405 |
| 4016 | UI-R-C2-ng-f-06-0-UI | Ribokinase | BF549657 |
| 4017 | UI-R-C2-nj-g-01-0-UI | Transcribed locus | AI071787 |
| 4018 | UI-R-C2-nl-d-07-0-UI | KIAA0368 | AI072190 |
| 4019 | UI-R-A0-ad-h-10-0-UI | ATPase, Na+/K+ transporting, alpha 2 polypeptide | AA818371 |
| 4020 | UI-R-A0-ao-h-01-0-UI | Protease, serine, 2 | AA819012 |
| 4021 | UI-R-A0-az-d-02-0-UI | Group specific component | AA818706 |
| 4022 | UI-R-A0-bh-b-05-0-UI | Splicing factor YT521-B | BF548049 |
| 4023 | UI-R-E0-co-h-12-0-UI | Glycogen synthase 2 | AA875404 |
| 4024 | UI-R-E0-cx-b-09-0-UI | Guanine nucleotide binding protein (G protein), gamma 8 subunit | BF555330 |
| 4025 | UI-R-C0-jf-h-11-0-UI | Adipose differentiation related protein | AI030932 |
| 4026 | UI-R-C0-jj-a-06-0-UI | Type 1 tumor necrosis factor receptor shedding aminopeptidase regulator | AI043793 |
| 4027 | UI-R-C1-kd-b-11-0-UI | UBX domain containing 1 | AI044855 |
| 4028 | UI-R-C1-kf-d-05-0-UI | Zinc finger protein 511 | AI045036 |
| 4029 | UI-R-C1-ki-d-10-0-UI | Guanosine diphosphate dissociation inhibitor 1 | BF546968 |
| 4030 | UI-R-C1-kk-b-03-0-UI | ELAV (embryonic lethal, abnormal vision, Drosophila)-like 3 (Hu antigen C) | BF546116 |
| 4031 | UI-R-C1-la-f-02-0-UI | XP_001108038.1 additional sex combs like 1 isoform 7 | AI059974 |
| 4032 | UI-R-C1-le-e-09-0-UI | NADH dehydrogenase (ubiquinone) 1 alpha subcomplex, 9 | AI059640 |
| 4033 | UI-R-C1-lo-b-06-0-UI | Dual specificity phosphatase 18 | BF559942 |
| 4034 | UI-R-C1-lp-g-10-0-UI | General transcription factor II I | AI070190 |
| 4035 | UI-R-A0-bf-c-07-0-UI | Mitochondrial ribosomal protein S36 | AA859464 |
| 4036 | UI-R-E1-fa-c-09-0-UI | Cleavage and polyadenylation specific factor 5 | AA955588 |
| 4037 | UI-R-E1-fg-c-08-0-UI | N-ethylmaleimide-sensitive factor attachment protein, gamma | AA956372 |
| 4038 | UI-R-E1-fk-h-03-0-UI | RIKEN cDNA 1810054G18 | AA956513 |
| 4039 | UI-R-C0-gw-g-07-0-UI | Ubiquitin specific protease 47 | BF557814 |
| 4040 | UI-R-C0-hd-g-11-0-UI | Cyclin D1 | AA996606 |
| 4041 | UI-R-C0-hi-d-01-0-UI | Microfibrillar-associated protein 4 | BF558008 |
| 4042 | UI-R-C0-hu-c-06-0-UI | XP_346918.1 hypothetical protein XP_346917 | BF550713 |
| 4043 | UI-R-C0-it-d-12-0-UI | Peroxisomal biogenesis factor 3 | AI030183 |
| 4044 | UI-R-C0-iy-d-07-0-UI | Trophinin associated protein (tastin) | AI029610 |
| 4045 | UI-R-C0-ht-g-02-0-UI | Lanosterol synthase | BF524102 |
| 4046 | UI-R-C0-iy-b-05-0-UI | Nucleoporin 155 | AI029686 |
| 4047 | UI-R-C1-kf-d-12-0-UI | Acidic ribosomal phosphoprotein P0 | AI045079 |
| 4048 | UI-R-C1-kn-b-04-0-UI | Decay accelarating factor 1 | AI058330 |
| 4049 | UI-R-C1-kw-e-11-0-UI | Chymase 1, mast cell | AI059436 |
| 4050 | UI-R-C1-le-e-01-0-UI | Solute carrier family 28 (sodium-coupled nucleoside transporter), member 2 | BF558528 |
| 4051 | UI-R-BT0-ps-d-07-0-UI | Tropomodulin 1 | AI145620 |
| 4052 | UI-R-C2p-oe-f-10-0-UI | Sialophorin | AI137130 |
| 4053 | UI-R-BT0-pn-c-12-0-UI | DNA-damage-inducible transcript 4 | AI144692 |
| 4054 | UI-R-A0-ae-a-11-0-UI | Glucose 6 phosphatase, catalytic, 3 | AA817793 |
| 4055 | UI-R-CV2-chm-f-03-0-UI | Cyclin C | BI298690 |
| 4056 | UI-R-CV2-cim-f-11-0-UI | Suppressor of Ty 4 homolog 2 | BI300586 |
| 4057 | UI-R-DN0-ciu-e-14-0-UI | Periaxin | BI292032 |
| 4058 | UI-R-DO0-ciw-f-23-0-UI | Prothymosin alpha | BI292639 |
| 4059 | UI-R-A1-dz-d-10-0-UI | Surfactant associated protein C | AA924666 |
| 4060 | UI-R-A1-ec-d-07-0-UI | Cellular retinoic acid binding protein 2 | AA925443 |
| 4061 | UI-R-A1-ef-h-04-0-UI | Dihydropyrimidinase | AI111911 |
| 4062 | UI-R-A1-ek-e-07-0-UI | Coronin, actin-binding protein, 1B | AA925301 |
| 4063 | UI-R-C0-hh-d-06-0-UI | Ubiquitin-conjugating enzyme E2G 1 (UBC7 homolog, C. elegans) | AA996896 |
| 4064 | UI-R-C0-hn-f-06-0-UI | MOCO sulphurase C-terminal domain containing 2 | AA997628 |
| 4065 | UI-R-CU0s-cbr-e-07-0-UI | Spermidine synthase | BI279837 |
| 4066 | UI-R-CU0s-cby-a-02-0-UI | Ubiquitin specific peptidase 16 | BI282215 |
| 4067 | UI-R-DK0-cdg-c-07-0-UI | CPG2 protein | BI288556 |
| 4068 | UI-R-DK0-cds-g-02-0-UI | Elongation factor RNA polymerase II | BI293622 |
| 4069 | UI-R-DK0-cec-a-07-0-UI | Ubiquitin specific protease 30 | BI294777 |
| 4070 | UI-R-DK0-cee-g-11-0-UI | Phosphoenolpyruvate carboxykinase 2 (mitochondrial) | BI295018 |
| 4071 | UI-R-CA1-bjx-h-12-0-UI | hypothetical protein FLJ25530 | BF410476 |
| 4072 | UI-R-BJ2-bqz-h-05-0-UI | S100 calcium binding protein A6 (calcyclin) | BF408115 |
| 4073 | UI-R-BJ2-bre-c-07-0-UI | RNA binding motif protein 10 | BF408483 |
| 4074 | UI-R-DK0-cfn-f-06-0-UI | AT rich interactive domain 2 (Arid-rfx like) | BI289741 |
| 4075 | UI-R-BJ2-bor-b-02-0-UI | Carbonyl reductase 3 | CK844545 |
| 4076 | UI-R-CA0-bpb-g-05-0-UI | Adaptor protein complex AP-1, sigma 1 | BF414611 |
| 4077 | UI-R-BJ2-bpo-d-03-0-UI | Topoisomerase (DNA) III beta | BF406720 |
| 4078 | UI-R-BJ2-bpw-d-05-0-UI | Potassium channel, subfamily K, member 6 | BF420428 |
| 4079 | UI-R-CW0-bwn-c-12-0-UI | Potassium channel, subfamily T, member 2 | BI276034 |
| 4080 | UI-R-CX0-bxb-g-01-0-UI | Transcribed locus | BI275109 |
| 4081 | UI-R-CX0-bxj-g-12-0-UI | Mucin 1, transmembrane | BI276735 |
| 4082 | UI-R-CY0-bxr-c-01-0-UI | Succinate dehydrogenase complex, subunit C, integral membrane protein | BI278247 |
| 4083 | UI-R-DE0-caf-b-03-0-UI | Peptidyl arginine deiminase, type II | BI279656 |
| 4084 | UI-R-CT0s-cau-a-12-0-UI | XP_001106970.1 small nuclear ribonucleoparticle-associated protein isoform 2 | BI281585 |
| 4085 | UI-R-CA0-bhx-b-05-0-UI | transcriptional regulating protein 132 | BF399673 |
| 4086 | UI-R-CA1-bio-c-10-0-UI | XP_221595.3 Eph receptor A6 | BF405003 |
| 4087 | UI-R-CV1-bsc-f-04-0-UI | Niban protein | BG372428 |
| 4088 | UI-R-CV1-bsn-b-08-0-UI | Ubiquitin-like 1 (sentrin) activating enzyme E1B | BG374252 |
| 4089 | UI-R-CS0-btg-g-01-0-UI | Ribosomal protein L21 | BG379422 |
| 4090 | UI-R-CT0-btw-e-04-0-UI | Vesicle-associated membrane protein 2 | BG380379 |
| 4091 | UI-R-CV1-bvo-f-09-0-UI | Exportin 6 | BG378160 |
| 4092 | UI-R-CA1-bkf-g-06-0-UI | mitochondrial translational release factor 1-like | BF415857 |
| 4093 | UI-R-CA0-bkv-d-04-0-UI | KIAA0456 protein | BF416816 |
| 4094 | UI-R-CA1-blf-f-11-0-UI | Protocadherin 8 | BF409443 |
| 4095 | UI-R-BS1-ayv-d-04-0-UI | Coiled-coil domain containing 43 | BE108360 |
| 4096 | UI-R-BS1-azd-f-01-0-UI | CDNA clone IMAGE:7370517 | BE109246 |
| 4097 | UI-R-BJ1-azq-d-10-0-UI | Caspase 6 | CK842252 |
| 4098 | UI-R-CA0-bag-b-09-0-UI | Solute carrier family 45, member 1 | BE119558 |
| 4099 | UI-R-CA1-bcn-e-09-0-UI | Solute carrier family 4, member 3 | BF390320 |
| 4100 | UI-R-BS2-bdc-h-09-0-UI | Myosin heavy chain Myr 8 | BF388471 |
| 4101 | UI-R-BS2-bdm-e-03-0-UI | Intestinal cell kinase | BF389322 |
| 4102 | UI-R-BS2-bdy-f-09-0-UI | XP_001118308.1 vascular endothelial growth factor B | BF396482 |
| 4103 | UI-R-CA0-bgw-a-03-0-UI | Poly (ADP-ribose) polymerase family, member 1 | BF393871 |
| 4104 | UI-R-CA0-bhg-b-08-0-UI | Ring finger protein 31 | BF400859 |
| 4105 | UI-R-BT1-aqg-c-07-0-UI | Chondroitin polymerizing factor | BE101631 |
| 4106 | UI-R-BT1-aqs-f-06-0-UI | Heme binding protein 2 | BE102666 |
| 4107 | UI-R-BJ1-atk-a-04-0-UI | Divalent cation tolerant protein CUTA | BE099673 |
| 4108 | UI-R-BJ1-atp-h-09-0-UI | Neurotrophin 3 | CK842967 |
| 4109 | UI-R-BJ1-atv-g-09-0-UI | Transmembrane protein 86A | BE100466 |
| 4110 | UI-R-BJ1-aue-e-09-0-UI | ATP-binding cassette, sub-family C (CFTR/MRP), member 4 | CK839501 |
| 4111 | UI-R-BJ1-avx-g-06-0-UI | Peptidase D | CK845603 |
| 4112 | UI-R-BJ1-awc-a-06-0-UI | 3000004C01Rik protein | CK844341 |
| 4113 | UI-R-BJ1-awi-b-05-0-UI | Tyrosine-protein kinase transmembrane receptor ROR1 precursor (Neurotrophic tyrosine kinase, receptor-related 1) | BE113932 |
| 4114 | UI-R-BJ1-awu-a-03-0-UI | Nedd4 family interacting protein 1 | CK840057 |
| 4115 | UI-R-BO1-aiy-a-12-0-UI | Spermatogenesis associated 20 | AW525995 |
| 4116 | UI-R-BO1-ajf-c-10-0-UI | Somatostatin receptor 3 | BF563137 |
| 4117 | UI-R-BO1-aji-g-07-0-UI | Synaptotagmin VI | AW531507 |
| 4118 | UI-R-BO1-ajo-d-07-0-UI | Potassium channel, subfamily T, member 1 | BF565122 |
| 4119 | UI-R-C4-aky-a-10-0-UI | Myeloperoxidase | BF564050 |
| 4120 | UI-R-C4-alg-b-03-0-UI | nuclear receptor binding protein | AW531053 |
| 4121 | UI-R-C4-alo-a-05-0-UI | Steroidogenic acute regulatory protein | AW534326 |
| 4122 | UI-R-C4-alv-h-07-0-UI | Prolactin family 2, subfamily a, member 1 | AW535333 |
| 4123 | UI-R-BU0-apg-d-04-0-UI | Synovial sarcoma translocation gene on chromosome 18-like 1 | BE096458 |
| 4124 | UI-R-BO1-apu-f-05-0-UI | Unc-13 homolog D | BE097215 |
| 4125 | UI-R-AA1-zw-d-02-0-UI | Proteasome (prosome, macropain) 26S subunit, non-ATPase, 2 | CK841008 |
| 4126 | UI-R-AG1-aak-h-10-0-UI | SplA/ryanodine receptor domain and SOCS box containing 3 | CK838406 |
| 4127 | UI-R-Y0-acg-g-07-0-UI | Bcl2-like 1 | BF523847 |
| 4128 | UI-R-Y0-act-d-01-0-UI | Carboxylesterase 1 | AI763895 |
| 4129 | UI-R-BJ0-ada-e-03-0-UI | Calcium/calmodulin-dependent protein kinase I | AW252518 |
| 4130 | UI-R-BJ0-aeb-a-06-0-UI | Hemoglobin alpha, adult chain 1 | AW252651 |
| 4131 | UI-R-BJ0p-agd-d-09-0-UI | Neuron navigator 2 | BF567012 |
| 4132 | UI-R-BO0-agl-c-10-0-UI | BCL2-associated athanogene 5 | AW521333 |
| 4133 | UI-R-BO0-agu-f-02-0-UI | Prostaglandin-endoperoxide synthase 2 | BF567671 |
| 4134 | UI-R-BO0-ahn-f-05-0-UI | Ubiquitin specific peptidase 33 | BF567853 |
| 4135 | UI-R-C3-sq-c-01-0-UI | Eukaryotic translation elongation factor 1 epsilon 1 | BF542762 |
| 4136 | UI-R-C3-sz-a-09-0-UI | Growth factor independent 1 | BF522373 |
| 4137 | UI-R-C3-tj-e-11-0-UI | XP_001096692.1 CG14286-PA | BF523271 |
| 4138 | UI-R-C3-tp-h-02-0-UI | heparan sulfate (glucosamine) 3-O-sulfotransferase 3A1 | AI548686 |
| 4139 | UI-R-AB0-vr-e-01-0-UI | Dynactin 3 | CK840357 |
| 4140 | UI-R-AD0-we-e-08-0-UI | Activity and neurotransmitter-induced early gene 2 (ania-2) mRNA, 3'UTR | CK842305 |
| 4141 | UI-R-AG0-wt-a-01-0-UI | Tetraspanin 18 | AI579018 |
| 4142 | UI-R-AG0-xb-e-07-0-UI | Ferritin light chain 1 | BF524289 |
| 4143 | UI-R-AE1-zg-d-01-0-UI | XP_001107138.1 myeloid-associated differentiation marker | CK839093 |
| 4144 | UI-R-AD1-zo-e-07-0-UI | Niemann Pick type C2 | CK842367 |
| 4145 | UI-R-C0-hr-a-09-0-UI | Three prime repair exonuclease 1 | BF551302 |
| 4146 | UI-R-C0-ib-e-11-0-UI | XP_509106.1 homeo box C9 [Pan troglodytes] | BF561684 |
| 4147 | UI-R-Y0-lx-a-07-0-UI | Actinin alpha 3 | AI073202 |
| 4148 | UI-R-Y0-mn-g-10-0-UI | Paired box gene 4 | AI112569 |
| 4149 | UI-R-C2-my-c-10-0-UI | TM2 domain containing 2 | AI071207 |
| 4150 | UI-R-C2p-nu-c-10-0-UI | Cytochrome P450IIB3 | BF553356 |
| 4151 | UI-R-BT0-qh-a-10-0-UI | NMDA receptor-regulated gene 1 | AI146224 |
| 4152 | UI-R-C2p-qm-f-06-0-UI | Eukaryotic translation initiation factor 4 gamma, 1 | BF522307 |
| 4153 | UI-R-C2p-qr-h-11-0-UI | Glycosyltransferase 25 domain containing 1 | AI555738 |
| 4154 | UI-R-C2p-rc-c-09-0-UI | GTP binding protein 1 | BF547319 |
| 4155 | UI-R-E0-bv-a-09-0-UI | SEC24 related gene family, member B | BF549916 |
| 4156 | UI-R-E0-bz-b-11-0-UI | TMEM9 domain family, member B | AA899055 |
| 4157 | UI-R-E0-cg-d-05-0-UI | CDC23 (cell division cycle 23, yeast, homolog) | AA859935 |
| 4158 | UI-R-E0-cm-c-10-0-UI | Dapper homolog 2, antagonist of beta-catenin | AA875577 |
| 4159 | UI-R-A1-du-e-02-0-UI | Zinc ribbon domain containing, 1 | AA924057 |
| 4160 | UI-R-A1-dy-f-06-0-UI | Fibroblast growth factor receptor 4 | BF559577 |
| 4161 | UI-R-A1-ee-a-06-0-UI | Transcribed locus |  |
| 4162 | UI-R-A1-ei-g-05-0-UI | Transmembrane protease, serine 8 (intestinal) | BF555392 |
| 4163 | UI-R-E1-go-f-10-0-UI | Sterol O-acyltransferase 1 | AA964474 |
| 4164 | UI-R-C0-hj-b-12-0-UI | Transcribed locus | BF554044 |
| 4165 | UI-R-C0-gv-g-07-0-UI | Dehydrogenase/reductase (SDR family) member 7B | BF557778 |
| 4166 | UI-R-AG1-aai-b-10-0-UI | XP_001114048.1 hypothetical protein | AI713860 |
| 4167 | UI-R-BT1-akd-c-04-0-UI | Zinc finger protein 219 | BF566331 |
| 4168 | UI-R-CA0-bai-e-01-0-UI | LRRC36 homolog | BE119743 |
| 4169 | UI-R-E0-by-d-10-0-UI | XP_001095281.1 CG17122-PA | BF549986 |
| 4170 | UI-R-E0-dc-g-04-0-UI | Transcribed locus | AA901178 |
| 4171 | UI-R-A1-du-h-09-0-UI | WT1-interacting protein | BF558832 |
| 4172 | UI-R-A1-ex-g-11-0-UI | Unc-45 homolog A | BF560130 |
| 4173 | UI-R-Y0-mp-d-03-0-UI | Solute carrier family 39 (zinc transporter), member 4 | AI111852 |
| 4174 | UI-R-C2p-od-h-04-0-UI | replication protein-binding trans-activator RBT1 | BF553446 |
| 4175 | UI-R-C0-hc-a-03-0-UI | Cleavage and polyadenylation factor subunit homolog | AA965160 |
| 4176 | UI-R-C0-hh-c-05-0-UI | Chemokine (C-C motif) ligand 19 | AA996885 |
| 4177 | UI-R-C0-je-e-06-0-UI | Small nuclear ribonucleoprotein D1 | AI030826 |
| 4178 | UI-R-C0-jo-a-07-0-UI | Transmembrane 9 superfamily protein member 4 | BF550966 |
| 4179 | UI-R-C1-jt-b-03-0-UI | Signal-induced proliferation-associated 1 like 2 | AI044777 |
| 4180 | UI-R-C1-jw-d-10-0-UI | Trafficking protein particle complex 4 | AI044482 |
| 4181 | UI-R-E0-br-e-03-0-UI | Interleukin 2 receptor, beta chain | AA866450 |
| 4182 | UI-R-C1-ki-e-04-0-UI | Transcribed locus | BF551663 |
| 4183 | UI-R-A0-am-d-04-0-UI | Selenocysteine lyase | BF522125 |
| 4184 | UI-R-A1-ev-c-04-0-UI | EST AA792894 | AA926271 |
| 4185 | UI-R-E1-fz-f-10-0-UI | Cyclic nucleotide phosphodiesterase 1 | AA957953 |
| 4186 | UI-R-BT0-pv-d-02-0-UI | Dynein cytoplasmic 1 intermediate chain 1 | AI145198 |
| 4187 | UI-R-A1-eu-g-08-0-UI | Fibroblast growth factor 13 | AA926362 |
| 4188 | UI-R-E1-gk-d-04-0-UI | Tyro protein tyrosine kinase binding protein | AA963886 |
| 4189 | UI-R-A0-av-f-06-0-UI | Growth hormone receptor | AA819745 |
| 4190 | UI-R-A0-bh-d-11-0-UI | XP_001107951.1 zinc finger, HIT type 1 isoform 2 | AA819697 |
| 4191 | UI-R-A0-ax-h-01-0-UI | Nascent-polypeptide-associated complex alpha polypeptide | BF549721 |
| 4192 | UI-R-A1-es-c-10-0-UI | XP_001113434.1 coronin, actin binding protein, 2A | AA926011 |
| 4193 | UI-R-C0-gt-d-10-0-UI | Proteoglycan 4 | AI113008 |
| 4194 | UI-R-C0-gz-a-03-0-UI | Diaphanous homolog 1 | BF560472 |
| 4195 | UI-R-E0-ce-e-07-0-UI | Calcium channel, voltage-dependent, beta 3 subunit | AA875155 |
| 4196 | UI-R-E0-ck-c-01-0-UI | Parathymosin | AA899878 |
| 4197 | UI-R-E1-fg-b-04-0-UI | Galanin | AA955779 |
| 4198 | UI-R-E1-fr-h-05-0-UI | Early growth response 1 | AA956793 |
| 4199 | UI-R-E1-gc-g-12-0-UI | Mitogen activated protein kinase 14 | AA958011 |
| 4200 | UI-R-C0-ie-c-12-0-UI | Dynamin 2 | BF561608 |
| 4201 | UI-R-A1-ev-e-01-0-UI | Pre-B-cell colony enhancing factor 1 | AA926158 |
| 4202 | UI-R-C0-gr-e-09-0-UI | Preimplantation protein 3 | AA964991 |
| 4203 | UI-R-C0-io-g-06-0-UI | Mitogen-activated protein kinase 8 | AI029013 |
| 4204 | UI-R-C1-jt-e-03-0-UI | Opioid growth factor receptor | BF551413 |
| 4205 | UI-R-C2-mv-c-08-0-UI | SH2 domain containing phosphatase anchor protein 1 | BF553090 |
| 4206 | UI-R-C2-mz-g-04-0-UI | Feminization 1 homolog b | BF552914 |
| 4207 | UI-R-C2-nb-g-03-0-UI | Caspase 7 | BF553536 |
| 4208 | UI-R-C2-ne-a-10-0-UI | XP_001110339.1 biogenesis of lysosome-related organelles complex-1, subunit 3 | AI072380 |
| 4209 | UI-R-C2-no-g-04-0-UI | ADP-ribosyltransferase 5 | AI070764 |
| 4210 | UI-R-C2p-nu-d-05-0-UI | Cystatin A (stefin A) | BF553358 |
| 4211 | UI-R-C2p-nx-e-04-0-UI | Protein UNQ9166/PRO28631 precursor | BF553646 |
| 4212 | UI-R-C2p-oa-h-02-0-UI | hepatic leukemia factor | BF553784 |
| 4213 | UI-R-E0-bs-f-07-0-UI | Sterol-C4-methyl oxidase-like | BF548011 |
| 4214 | UI-R-E0-bx-g-03-0-UI | Adenylate kinase 3 | AA859704 |
| 4215 | UI-R-C0-if-h-08-0-UI | Hypothetical LOC314467 | AA998592 |
| 4216 | UI-R-C0-ik-e-12-0-UI | IMP4, U3 small nucleolar ribonucleoprotein, homolog | AI137860 |
| 4217 | UI-R-C0-jp-f-10-0-UI | RIKEN cDNA 4921537D05 | AI031052 |
| 4218 | UI-R-C1-jx-e-12-0-UI | Protease inhibitor 16 | AI044661 |
| 4219 | UI-R-C1-jy-g-09-0-UI | Cleavage and polyadenylation specificity factor 3 | BF550879 |
| 4220 | UI-R-C1-kb-g-02-0-UI | DKFZP547E1010 protein | AI044287 |
| 4221 | UI-R-C1-kq-f-09-0-UI | Pericentriolar material 1 | AI059175 |
| 4222 | UI-R-C1-ks-d-06-0-UI | Carbamyl phosphatate synthetase 2 | BF550623 |
| 4223 | UI-R-C1-kx-b-11-0-UI | Cell death-inducing DNA fragmentation factor, alpha subunit-like effector B | AI058382 |
| 4224 | UI-R-C1-kz-b-09-0-UI | Interleukin enhancer binding factor 3 | BF547104 |
| 4225 | UI-R-A0-al-c-07-0-UI | SEC22 vesicle trafficking protein-like 1 | AA819253 |
| 4226 | UI-R-A0-ap-g-02-0-UI | Keratin complex 1, acidic, gene 5 | BF548510 |
| 4227 | UI-R-A0-as-b-12-0-UI | Nucleoredoxin | AA818925 |
| 4228 | UI-R-A0-be-a-08-0-UI | Transcribed locus | BF559007 |
| 4229 | UI-R-E1-fo-e-10-0-UI | Transmembrane protein 110 | AA956738 |
| 4230 | UI-R-E1-ft-h-07-0-UI | Quinolinate phosphoribosyltransferase | AA957222 |
| 4231 | UI-R-E1-gp-a-08-0-UI | Methyl-CpG-binding domain protein 2 (Methyl-CpG-binding protein MBD2) | BF557095 |
| 4232 | UI-R-C0-gw-a-04-0-UI | Histone cluster 1, H2bh | AA964051 |
| 4233 | UI-R-C0-ia-f-08-0-UI | Exosome component 7 | BF561514 |
| 4234 | UI-R-C0-id-b-07-0-UI | Dynactin 4 | AA998140 |
| 4235 | UI-R-C0-gx-e-02-0-UI | Inositol 1,4,5-triphosphate receptor 3 | BF557837 |
| 4236 | UI-R-C0-hc-f-04-0-UI | Solute carrier family 22 (organic cation transporter), member 1 | AA965127 |
| 4237 | UI-R-C0-jj-h-12-0-UI | Fc receptor, IgG, low affinity IIb | BF554650 |
| 4238 | UI-R-C0-jq-h-07-0-UI | Aconitase 1 | AI045830 |
| 4239 | UI-R-C1-jw-c-07-0-UI | Par-3 (partitioning defective 3) homolog | AI044473 |
| 4240 | UI-R-C1-kd-g-02-0-UI | Nudix (nucleoside diphosphate linked moiety X)-type motif 1 | AI044704 |
| 4241 | UI-R-C1-ln-g-03-0-UI | Fibrinogen, B beta polypeptide | AI071033 |
| 4242 | UI-R-C2-mt-h-03-0-UI | Bone morphogenetic protein 2 | BF549588 |
| 4243 | UI-R-C2-nc-g-11-0-UI | Protein tyrosine phosphatase, receptor type, N | AI071725 |
| 4244 | UI-R-C2-nn-h-11-0-UI | Matrin 3 | AI072656 |
| 4245 | UI-R-DK0-cfy-a-11-0-UI | Human immunodeficiency virus type I enhancer binding protein 2 | BI290676 |
| 4246 | UI-R-DK0-cgd-e-08-0-UI | CD7 antigen | BI290548 |
| 4247 | UI-R-CV2-cgn-b-08-0-UI | Solute carrier family 5 (inositol transporters), member 3 | BI291239 |
| 4248 | UI-R-CV2-chb-a-08-0-UI | RNA binding protein gene with multiple splicing | BI297816 |
| 4249 | UI-R-DR0-cjb-l-07-0-UI | Protocadherin 3 | BI303345 |
| 4250 | UI-R-E0-dn-g-07-0-UI | D-dopachrome tautomerase | AA900788 |
| 4251 | UI-R-A1-ds-e-04-0-UI | Protein kinase, AMP-activated, beta 1 non-catalytic subunit | BF555889 |
| 4252 | UI-R-A1-dv-g-10-0-UI | Kinase insert domain protein receptor | BF555734 |
| 4253 | UI-R-E1-gj-c-06-0-UI | Defender against cell death 1 | BF557616 |
| 4254 | UI-R-C0-gt-a-02-0-UI | Cytochrome P450, family 2, subfamily f, polypeptide 2 | BF559660 |
| 4255 | UI-R-DA0-bze-b-03-0-UI | Salivary protein 1 | BI283574 |
| 4256 | UI-R-DD0-bzx-h-10-0-UI | Ribosomal protein L34 | BI282526 |
| 4257 | UI-R-CW0s-ccg-e-09-0-UI | Thyroid hormone responsive protein | BI283692 |
| 4258 | UI-R-CX0s-ccq-c-08-0-UI | Kidney predominant protein NCU-G1 | BI284280 |
| 4259 | UI-R-CX0s-ccv-g-01-0-UI | Prolylcarboxypeptidase (angiotensinase C) | BI285860 |
| 4260 | UI-R-DK0-cdd-f-04-0-UI | Leucine zipper, putative tumor suppressor 2 | BI288673 |
| 4261 | UI-R-DK0-ceu-d-05-0-UI | Gamma-glutamyltransferase-like 3 | BI295687 |
| 4262 | UI-R-DK0-cfb-c-06-0-UI | Pleckstrin homology domain containing, family A (phosphoinositide binding specific) member 4 | BI295839 |
| 4263 | UI-R-DK0-cff-e-02-0-UI | SRY-box containing gene 6 | BI289559 |
| 4264 | UI-R-CM0-bjp-f-12-0-UI | Myoglobin | BF395661 |
| 4265 | UI-R-CN0-bml-e-09-0-UI | Pumilio 1 | BF410834 |
| 4266 | UI-R-BT1-bnf-h-07-0-UI | N-acylsphingosine amidohydrolase 1 | BF408756 |
| 4267 | UI-R-BT1-bob-b-09-0-UI | XP_347116.2 hypothetical protein XP_347115 | BF413441 |
| 4268 | UI-R-BJ2-boo-a-02-0-UI | TRM1 tRNA methyltransferase 1 homolog | BF414231 |
| 4269 | UI-R-BJ2-bql-g-10-0-UI | Translocase of inner mitochondrial membrane 8 homolog a | CK843731 |
| 4270 | UI-R-BJ2-bqp-h-05-0-UI | PHD finger protein 1 | BF419293 |
| 4271 | UI-R-CW0-bvz-c-02-0-UI | CREB regulated transcription coactivator 2 | BI273808 |
| 4272 | UI-R-CW0-bwj-a-06-0-UI | XP_001098582.1 hypothetical protein | BI274448 |
| 4273 | UI-R-CZ0-byi-d-06-0-UI | Peroxisomal trans-2-enoyl-CoA reductase | BI279038 |
| 4274 | UI-R-DB0-bys-d-06-0-UI | XP_001103469.1 RAB11 family interacting protein 5 (class I) | BI278908 |
| 4275 | UI-R-CA0-bgb-g-01-0-UI | Sulfatase modifying factor 1 | BF393769 |
| 4276 | UI-R-CA0-bgo-h-05-0-UI | chondroitin beta1,4 N-acetylgalactosaminyltransferase | BF401804 |
| 4277 | UI-R-CA1-bjb-a-20-0-UI | hypothetical protein | BF399316 |
| 4278 | UI-R-CA1-bje-b-11-0-UI | RIKEN cDNA 1810060J02 | BF405960 |
| 4279 | UI-R-CV0-brj-e-09-0-UI | Phosducin | BG371888 |
| 4280 | UI-R-CV1-bry-e-05-0-UI | LUC7-like | BG373449 |
| 4281 | UI-R-CT0-bui-g-01-0-UI | Solute carrier family 13 (sodium-dependent citrate transporter), member 5 | BG381311 |
| 4282 | UI-R-CU0-bus-b-08-0-UI | Hypothetical LOC300361 | BG376240 |
| 4283 | UI-R-CU0-bvb-g-07-0-UI | NADH dehydrogenase (ubiquinone) Fe-S protein 6 | BG377209 |
| 4284 | UI-R-CU0-bvl-g-03-0-UI | Calcium modulating ligand | BG376976 |
| 4285 | UI-R-CA0-axe-g-10-0-UI | F-box only protein 2 | BE113904 |
| 4286 | UI-R-CA0-axp-c-11-0-UI | Lactate dehydrogenase A-like 6B | BE114359 |
| 4287 | UI-R-BS1-ayc-e-04-0-UI | Cell division cycle associated 2 | BE116372 |
| 4288 | UI-R-BS1-ays-b-03-0-UI | SH3-domain binding protein 5 (BTK-associated) | BE107294 |
| 4289 | UI-R-CA0-bav-e-04-0-UI | Microtubule-associated protein 2 | BE120607 |
| 4290 | UI-R-CA1-bbe-f-07-0-UI | Myotubularin related protein 6 | BF386537 |
| 4291 | UI-R-CA1-bbw-h-07-0-UI | Transcribed locus | BF387977 |
| 4292 | UI-R-CA1-bcf-c-12-0-UI | CD6 antigen | BF391027 |
| 4293 | UI-R-BS2-beq-h-07-0-UI | Transcription factor AP-2 beta | BF398617 |
| 4294 | UI-R-CA0-bfh-a-10-0-UI | Interleukin 1 receptor accessory protein | BF391914 |
| 4295 | UI-R-BS0-ans-a-12-0-UI | 5-aminoimidazole-4-carboxamide ribonucleotide formyltransferase/IMP cyclohydrolase | AW534481 |
| 4296 | UI-R-BU0-apd-a-07-0-UI | Myosin, heavy polypeptide 13, skeletal muscle | BE095908 |
| 4297 | UI-R-BX0-aru-e-03-0-UI | XP_001091930.1 fibrillarin | BE104857 |
| 4298 | UI-R-BO1-ask-g-06-0-UI | ATP-binding cassette, sub-family C (CFTR/MRP), member 8 | BE106087 |
| 4299 | UI-R-BT1-asv-c-06-0-UI | Sperm autoantigenic protein 17 | BE106377 |
| 4300 | UI-R-BJ1-atf-g-05-0-UI | HT014 | BE099049 |
| 4301 | UI-R-BJ1-aur-f-05-0-UI | RIKEN cDNA 1110021N07 | CK839759 |
| 4302 | UI-R-BJ1-ave-e-07-0-UI | Protein arginine N-methyltransferase 5 | BE112036 |
| 4303 | UI-R-BJ1-avp-b-08-0-UI | XP_049237.2 Protein KIAA0841 isoform 1 | BE109522 |
| 4304 | UI-R-BJ1-avu-g-07-0-UI | Receptor-like tyrosine kinase | BE110680 |
| 4305 | UI-R-BO0-aia-a-01-0-UI | XP_001103753.1 restin-like 2 isoform 9 | AW524572 |
| 4306 | UI-R-BO0-aig-c-09-0-UI | Cyclin M3 | BF562924 |
| 4307 | UI-R-BJ0p-ain-b-02-0-UI | Poly(A) binding protein, nuclear 1 | BF562801 |
| 4308 | UI-R-BJ0p-ait-h-02-0-UI | Tumor susceptibility gene 101 | AW525454 |
| 4309 | UI-R-BT1-ajx-d-10-0-UI | G protein-coupled receptor 175 | AW528347 |
| 4310 | UI-R-BT1-akg-b-04-0-UI | Transcribed locus | AW527960 |
| 4311 | UI-R-BT1-akl-g-08-0-UI | Purine-rich element binding protein G | BF565559 |
| 4312 | UI-R-BT1-akt-f-07-0-UI | DEAH (Asp-Glu-Ala-His) box polypeptide 40 | BF563823 |
| 4313 | UI-R-BU0-amu-e-09-0-UI | XP_001117874.1 F-box and leucine-rich repeat protein 14 | AW530261 |
| 4314 | UI-R-BU0-anc-e-04-0-UI | Nardilysin, N-arginine dibasic convertase 1 | AW533820 |
| 4315 | UI-R-AB1-yt-f-10-0-UI | Cnksr family member 3 | CK840841 |
| 4316 | UI-R-AB1-za-d-07-0-UI | mitochondrial ribosomal protein S11 | CK840924 |
| 4317 | UI-R-AF1-aax-e-10-0-UI | Solute carrier family 6 (neurotransmitter transporter), member 17 | BF543206 |
| 4318 | UI-R-Y0-abg-c-10-0-UI | Cytochrome P450, family 46, subfamily a, polypeptide 1 | BF543341 |
| 4319 | UI-R-Y0-abs-b-10-0-UI | G protein-coupled receptor 56 | AI715576 |
| 4320 | UI-R-Y0-acd-b-07-0-UI | cisplatin resistance associated | AI716026 |
| 4321 | UI-R-BJ0p-afd-a-01-0-UI | Cytochrome c oxidase, subunit XVII assembly protein homolog | CK839236 |
| 4322 | UI-R-BJ0p-afj-h-04-0-UI | WD repeat domain 39 | AW435217 |
| 4323 | UI-R-BJ0p-afp-e-12-0-UI | NTF2-related export protein 1 | CK841568 |
| 4324 | UI-R-BJ0p-afz-h-03-0-UI | Transportin 2 (importin 3, karyopherin beta 2b) | BF566985 |
| 4325 | UI-R-C2p-rl-h-06-0-UI | Nerve growth factor receptor (TNFR superfamily, member 16) | AI556540 |
| 4326 | UI-R-C2p-rt-g-03-0-UI | Mannoside acetylglucosaminyltransferase 5 | BF547459 |
| 4327 | UI-R-C2p-sc-f-07-0-UI | Down syndrome critical region gene 1-like 1 | AI501566 |
| 4328 | UI-R-C3-sl-b-08-0-UI | KIAA1730 protein | AI547457 |
| 4329 | UI-R-G0-ub-f-11-0-UI | Natriuretic peptide receptor 3 | BF522483 |
| 4330 | UI-R-G0-uo-e-12-0-UI | Heat shock 105kDa/110kDa protein 1 | BF525282 |
| 4331 | UI-R-Y0-uy-b-07-0-UI | Transcribed locus | AI575804 |
| 4332 | UI-R-Y0-vi-d-11-0-UI | Chaperone, ABC1 activity of bc1 complex like | BF523989 |
| 4333 | UI-R-AF0-xy-e-05-0-UI | Cysteine-rich hydrophobic domain 2 | BF524854 |
| 4334 | UI-R-AC0-yl-a-07-0-UI | Williams-Beuren syndrome chromosome region 1 homolog | CK838855 |
| 4335 | UI-R-E1-gb-b-06-0-UI | Carbonic anhydrase 3 | AA957597 |
| 4336 | UI-R-E1-gj-b-05-0-UI | Insulin-like growth factor 2, binding protein 1 | AA963215 |
| 4337 | UI-R-C0-jk-a-07-0-UI | Glucosamine | BF550557 |
| 4338 | UI-R-C1-kg-b-01-0-UI | CGI-09 protein | AI045105 |
| 4339 | UI-R-C1-ky-b-05-0-UI | Oxidized low density lipoprotein (lectin-like) receptor 1 | AI071531 |
| 4340 | UI-R-C1-ln-f-10-0-UI | Ring finger protein 39 | AI070049 |
| 4341 | UI-R-C2p-oj-b-09-0-UI | Inositol 1,4,5-trisphosphate 3-kinase C | AI137200 |
| 4342 | UI-R-BT0-po-e-08-0-UI | hypothetical protein MGC5297 | AI144981 |
| 4343 | UI-R-BT0-pu-b-04-0-UI | ST8 alpha-N-acetyl-neuraminide alpha-2,8-sialyltransferase 3 | BF544363 |
| 4344 | UI-R-BT0-qc-e-02-0-UI | Protein kinase, cGMP-dependent, type 1 | BF545798 |
| 4345 | UI-R-A0-ao-b-04-0-UI | GLI pathogenesis-related 1 (glioma) | AA819081 |
| 4346 | UI-R-A0-bb-e-03-0-UI | XP_001085984.1 dynein, axonemal, heavy polypeptide 1 | AA858749 |
| 4347 | UI-R-A0-bn-h-09-0-UI | Transmembrane protein 55A | AA858821 |
| 4348 | UI-R-E0-bt-e-04-0-UI | Ring finger protein 7 | AA858518 |
| 4349 | UI-R-E0-cy-d-07-0-UI | RAS, dexamethasone-induced 1 | AA899689 |
| 4350 | UI-R-E0-dg-a-05-0-UI | Rho guanine nucleotide exchange factor (GEF) 1 | BF558536 |
| 4351 | UI-R-E0-dn-e-12-0-UI | Transcription factor 19 | BF556672 |
| 4352 | UI-R-A1-ds-a-02-0-UI | Jun D proto-oncogene | AA924375 |
| 4353 | UI-R-A1-es-e-11-0-UI | Surfactant associated protein B | AI112934 |
| 4354 | UI-R-E1-fg-b-01-0-UI | exosome component 10 | AA956147 |
| 4355 | UI-R-CU0-bvb-c-12-0-UI | MORN repeat containing 1 | BG377132 |
| 4356 | UI-R-DE0-caf-a-07-0-UI | hypothetical protein MGC15677 | BI279648 |
| 4357 | UI-R-E1-gf-h-06-0-UI | Protein tyrosine phosphatase-like (proline instead of catalytic arginine), member b | AA957576 |
| 4358 | UI-R-C0-hm-d-12-0-UI | Aldehyde dehydrogenase 1 family, member B1 | AA997683 |
| 4359 | UI-R-C0-in-c-02-0-UI | Malate dehydrogenase 1B, NAD (soluble) | AA999081 |
| 4360 | UI-R-C1-kk-c-11-0-UI | RIKEN cDNA 5430432M24 | BF523570 |
| 4361 | UI-R-C2p-rc-f-03-0-UI | Sterile alpha motif domain containing 4 | AI555969 |
| 4362 | UI-R-C2p-rs-c-04-0-UI | Solute carrier family 9 (sodium/hydrogen exchanger), member 6 | AI501017 |
| 4363 | UI-R-C0-ir-c-05-0-UI | Glutaryl-Coenzyme A dehydrogenase | AI029435 |
| 4364 | UI-R-C0-ix-d-07-0-UI | Cell division cycle 37 homolog -like 1 | AI029565 |
| 4365 | UI-R-C1-km-b-02-0-UI | ORM1-like 2 | AI045922 |
| 4366 | UI-R-C1-ku-c-09-0-UI | Heterogeneous nuclear ribonucleoprotein C | AI058534 |
| 4367 | UI-R-C1-lj-d-04-0-UI | TAF11 RNA polymerase II, TATA box binding protein (TBP)-associated factor | AI059572 |
| 4368 | UI-R-C1-ln-a-10-0-UI | CAMP responsive element binding protein 3-like 4 | AI070020 |
| 4369 | UI-R-C0-gy-a-06-0-UI | Transcribed locus |  |
| 4370 | UI-R-C0-ir-a-10-0-UI | Ufm1-conjugating enzyme 1 | AI029348 |
| 4371 | UI-R-C1-kn-d-09-0-UI | High mobility group nucleosomal binding domain 2 | AI058430 |
| 4372 | UI-R-Y0-lt-b-11-0-UI | Chaperonin containing TCP1, subunit 2 (beta) | AI070372 |
| 4373 | UI-R-C0-hl-c-08-0-UI | Protease, serine, 15 | AA997362 |
| 4374 | UI-R-C0-ix-g-01-0-UI | Checkpoint kinase 1 homolog | AI029334 |
| 4375 | UI-R-C1-ka-d-12-0-UI | Mki67 (FHA domain) interacting nucleolar phosphoprotein | AI045625 |
| 4376 | UI-R-C1-ln-g-09-0-UI | Sertolin | AI070054 |
| 4377 | UI-R-A1-ev-e-02-0-UI | Laminin, alpha 2 | BF556962 |
| 4378 | UI-R-E1-fr-e-10-0-UI | Rhomboid family 1 | AA956764 |
| 4379 | UI-R-E1-fw-e-12-0-UI | Methylmalonyl CoA epimerase | BF561940 |
| 4380 | UI-R-E1-gj-d-02-0-UI | Tubulin, epsilon 1 | AA963234 |
| 4381 | UI-R-C0-ih-b-04-0-UI | Transcribed locus | AI137704 |
| 4382 | UI-R-C0-im-h-12-0-UI | GLIS family zinc finger 1 | BF561793 |
| 4383 | UI-R-A1-es-c-03-0-UI | Tyrosine 3-monooxygenase/tryptophan 5-monooxygenase activation protein, eta polypeptide | BF556364 |
| 4384 | UI-R-A1-ex-h-05-0-UI | TYRO3 protein tyrosine kinase 3 | AA955504 |
| 4385 | UI-R-C0-io-g-05-0-UI | Alanine-glyoxylate aminotransferase | BF551383 |
| 4386 | UI-R-Y0-ls-h-03-0-UI | Tyrosine 3-monooxygenase/tryptophan 5-monooxygenase activation protein, beta polypeptide | AI070102 |
| 4387 | UI-R-BT0-qi-f-04-0-UI | Amiloride-sensitive cation channel 1, neuronal (degenerin) | BF544644 |
| 4388 | UI-R-A0-bi-c-04-0-UI | RAN, member RAS oncogene family | AA858998 |
| 4389 | UI-R-C1-ll-g-07-0-UI | Pancreatic lipase-related protein 2 | BF559904 |
| 4390 | UI-R-A0-ag-g-04-0-UI | Cysteine-rich protein 2 | BF547534 |
| 4391 | UI-R-E0-by-b-02-0-UI | Matrix metallopeptidase 23 | AA900609 |
| 4392 | UI-R-A1-eu-e-11-0-UI | Deoxyribonuclease I | BF556940 |
| 4393 | UI-R-C2-ng-a-08-0-UI | DNA cross-link repair 1C, PSO2 homolog | BF549652 |
| 4394 | UI-R-C2-ni-c-10-0-UI | Dipeptidylpeptidase 7 | BF553141 |
| 4395 | UI-R-C2-nk-b-05-0-UI | Pumilio 2 | AI072431 |
| 4396 | UI-R-C2-nm-f-01-0-UI | RIKEN cDNA 1110008J03 | BF552835 |
| 4397 | UI-R-A0-aj-d-08-0-UI | Peroxiredoxin 2 | CK845616 |
| 4398 | UI-R-A0-av-b-12-0-UI | ribosomal protein L27 | AA818880 |
| 4399 | UI-R-A0-bc-e-06-0-UI | Cysteine dioxygenase 1, cytosolic | AA818579 |
| 4400 | UI-R-A0-bk-f-06-0-UI | Dual specificity phosphatase 6 | AA819366 |
| 4401 | UI-R-E0-cr-f-02-0-UI | Tuberous sclerosis 2 | AA899998 |
| 4402 | UI-R-E0-dd-c-01-0-UI | Apurinic/apyrimidinic endonuclease 1 | AA900301 |
| 4403 | UI-R-C0-jg-h-11-0-UI | Protein phosphatase 1, regulatory (inhibitor) subunit 11 | AI029677 |
| 4404 | UI-R-C0-jj-h-01-0-UI | BolA domain-containing protein like (11.4 kD) (1P25) | AI043827 |
| 4405 | UI-R-C1-ke-a-07-0-UI | Guanine nucleotide binding protein, alpha q polypeptide | AI044971 |
| 4406 | UI-R-C1-kg-d-03-0-UI | Dihydrolipoamide dehydrogenase | AI045119 |
| 4407 | UI-R-C1-kj-d-11-0-UI | Carbonic reductase 4 | AI045497 |
| 4408 | UI-R-C1-kk-h-06-0-UI | Membralin | AI044927 |
| 4409 | UI-R-C1-lc-b-08-0-UI | 2-4-dienoyl-Coenzyme A reductase 2, peroxisomal | BF546509 |
| 4410 | UI-R-C1-lf-c-09-0-UI | Muscle glycogen phosphorylase | AI059321 |
| 4411 | UI-R-C1-lo-h-09-0-UI | RNA binding motif protein 21 | AI069971 |
| 4412 | UI-R-Y0-lu-g-09-0-UI | Endothelial differentiation-related factor 1 | AI070143 |
| 4413 | UI-R-A1-ez-b-04-0-UI | Ribosomal protein S6 kinase, polypeptide 4 | AA955129 |
| 4414 | UI-R-E1-ff-f-04-0-UI | TAR (HIV) RNA binding protein 2 | BF560147 |
| 4415 | UI-R-E1-fk-a-10-0-UI | transmembrane protein induced by tumor necrosis factor alpha | BF556480 |
| 4416 | UI-R-E1-fl-h-07-0-UI | XP_577579.1 novel SCAN domain containing C2H2 type zinc finger protein | AA956967 |
| 4417 | UI-R-C0-hd-a-12-0-UI | Catenin (cadherin associated protein), alpha-like 1 | BF560031 |
| 4418 | UI-R-C0-he-g-11-0-UI | Mannosidase 2, alpha B1 | BF557874 |
| 4419 | UI-R-C0-hj-a-12-0-UI | Papillary renal cell carcinoma (translocation-associated) | AA996628 |
| 4420 | UI-R-C0-hv-e-04-0-UI | Chemokine (C-X-C motif) ligand 9 | BF554350 |
| 4421 | UI-R-C0-iu-e-05-0-UI | Ash2 (absent, small, or homeotic)-like | AI030342 |
| 4422 | UI-R-C0-ja-a-03-0-UI | XP_001088711.1 TATA element modulatory factor 1 isoform 2 | AI030004 |
| 4423 | UI-R-C0-hv-h-11-0-UI | CD48 antigen | AA997842 |
| 4424 | UI-R-C0-jc-a-04-0-UI | Pyruvate kinase, liver and red blood cell | BF560699 |
| 4425 | UI-R-C1-kk-a-09-0-UI | Hydroxysteroid (17-beta) dehydrogenase 7 | AI045145 |
| 4426 | UI-R-C1-kr-h-01-0-UI | Transcribed locus | AI059942 |
| 4427 | UI-R-C1-kz-b-05-0-UI | Mitogen-activated protein kinase 9 | BF547102 |
| 4428 | UI-R-C1-lh-b-06-0-UI | Annexin A4 | BF549511 |
| 4429 | UI-R-C2p-ny-a-09-0-UI | Fatty acid amide hydrolase | AI136093 |
| 4430 | UI-R-C2p-ok-f-06-0-UI | Peroxisomal membrane protein 2 | BF552142 |
| 4431 | UI-R-A0-ac-b-08-0-UI | RIKEN cDNA 0610008C08 | AA859102 |
| 4432 | UI-R-A0-ah-a-11-0-UI | Elongation factor Tu GTP binding domain containing 2 | BF549231 |
| 4433 | UI-R-CV2-cie-b-06-0-UI | splicing factor, arginine/serine-rich 2, interacting protein | BI300366 |
| 4434 | UI-R-DN0-cit-b-10-0-UI | Ornithine decarboxylase antizyme 1 | BI291781 |
| 4435 | UI-R-DN0-civ-k-09-0-UI | Mucin 3 | BI292202 |
| 4436 | UI-R-DO0-cix-n-02-0-UI | Eukaryotic translation elongation factor 1 alpha 1 | BI302705 |
| 4437 | UI-R-A1-ea-f-03-0-UI | Cytochrome P450, family 4, subfamily a, polypeptide 14 | AA924591 |
| 4438 | UI-R-A1-ee-b-03-0-UI | Cytochrome P450, subfamily 51 | BF550230 |
| 4439 | UI-R-A1-eh-h-06-0-UI | Cathepsin K | AA925246 |
| 4440 | UI-R-A1-en-e-07-0-UI | Platelet-activating factor acetylhydrolase, isoform Ib, alpha subunit 45kDa | AA925047 |
| 4441 | UI-R-C0-hl-c-06-0-UI | COP9 (constitutive photomorphogenic) homolog, subunit 8 | AA997360 |
| 4442 | UI-R-C0-hq-f-10-0-UI | Potassium voltage gated channel, Shaw-related subfamily, member 1 | AA997380 |
| 4443 | UI-R-CU0s-cbw-c-09-0-UI | CCAAT/enhancer binding protein zeta | BI282078 |
| 4444 | UI-R-CW0s-ccb-a-04-0-UI | Tetratricopeptide repeat domain 1 | BI285512 |
| 4445 | UI-R-DK0-cdp-d-02-0-UI | expressed sequence AV312086 | BI293086 |
| 4446 | UI-R-DK0-cdy-g-12-0-UI | Retinol dehydrogenase 13 (all-trans and 9-cis) | BI294052 |
| 4447 | UI-R-DK0-ced-e-01-0-UI | Transcribed locus | BI294902 |
| 4448 | UI-R-DK0-ceg-a-10-0-UI | Thioredoxin domain containing 12 (endoplasmic reticulum) | BI295123 |
| 4449 | UI-R-BJ2-bqv-g-02-0-UI | Nitric oxide synthase 3, endothelial cell | BF407713 |
| 4450 | UI-R-BJ2-brc-b-10-0-UI | Upregulated during skeletal muscle growth 5 | CK843426 |
| 4451 | UI-R-DK0-cfh-b-01-0-UI | Phosphodiesterase 4B, cAMP specific | BI289444 |
| 4452 | UI-R-DK0-cfq-g-01-0-UI | Uncoupling protein 3 (mitochondrial, proton carrier) | BI290005 |
| 4453 | UI-R-BJ2-bov-g-08-0-UI | Translocase of inner mitochondrial membrane 10 homolog | BF415172 |
| 4454 | UI-R-BJ2-bpk-d-11-0-UI | DNA segment on chromosome X and Y (unique) 155 expressed sequence isoform 1 | CK844616 |
| 4455 | UI-R-BJ2-bpr-e-01-0-UI | Transcription factor E3 | BF407083 |
| 4456 | UI-R-BJ2-bqf-c-09-0-UI | KIAA1749 protein | BF418421 |
| 4457 | UI-R-CX0-bwu-e-11-0-UI | Adrenomedullin 2 | BI275361 |
| 4458 | UI-R-CX0-bxi-a-07-0-UI | CDC-like kinase 1 | BI275917 |
| 4459 | UI-R-CW0-bxn-e-07-0-UI | Aconitase 2, mitochondrial | BI278591 |
| 4460 | UI-R-CY0-bxv-e-03-0-UI | GS2 gene | BI277158 |
| 4461 | UI-R-DE0-cak-f-06-0-UI | Keratin complex 2, basic, gene 5 | BI286411 |
| 4462 | UI-R-CS0s-cbn-a-04-0-UI | cullin 7 | BI287277 |
| 4463 | UI-R-CA1-big-a-04-0-UI | Astrotactin 1 | BF404491 |
| 4464 | UI-R-CA1-biw-a-14-0-UI | Sorting nexing 24 | BF404172 |
| 4465 | UI-R-CV1-bsk-c-05-0-UI | FGF receptor activating protein 1 | BG373817 |
| 4466 | UI-R-CV1-bsx-g-09-0-UI | Hepatoma-derived growth factor, related protein 2 | BG375158 |
| 4467 | UI-R-CS0-bto-e-11-0-UI | CDK5 regulatory subunit associated protein 3 | BG379981 |
| 4468 | UI-R-CT0-btz-b-09-0-UI | Pyruvate dehydrogenase kinase, isoenzyme 2 | BG380597 |
| 4469 | UI-R-CV1-bvt-h-05-0-UI | WW domain binding protein 5 | BG378874 |
| 4470 | UI-R-BT1-bkn-c-11-0-UI | Interleukin 17B | BF409208 |
| 4471 | UI-R-CN0-blc-e-08-0-UI | Microtubule-associated protein 1 light chain 3 alpha | BF417225 |
| 4472 | UI-R-CA1-blm-f-09-0-UI | Sortilin-related VPS10 domain containing receptor 3 | BF409626 |
| 4473 | UI-R-BS1-aza-e-02-0-UI | hypothetical protein FLJ21816 | BE108967 |
| 4474 | UI-R-BS1-azj-f-04-0-UI | Tropomyosin 1, alpha | BE118215 |
| 4475 | UI-R-CA0-azy-f-01-0-UI | Vacuolar ATPase subunit H | BE119108 |
| 4476 | UI-R-CA0-bao-c-05-0-UI | Gamma-aminobutyric acid (GABA-A) receptor, subunit alpha 5 | BE119983 |
| 4477 | UI-R-CA1-bcv-d-07-0-UI | RIKEN cDNA 2610003J06 | BF391268 |
| 4478 | UI-R-BS2-bdh-e-01-0-UI | Transcribed locus | BF389068 |
| 4479 | UI-R-BS2-bdu-d-04-0-UI | Ubiquitin-like, containing PHD and RING finger domains, 1 | BF396024 |
| 4480 | UI-R-BS2-beb-b-06-0-UI | PAX interacting (with transcription-activation domain) protein 1 | BF396958 |
| 4481 | UI-R-CA0-bha-a-06-0-UI | Gastrin | BF394134 |
| 4482 | UI-R-CA0-bhn-c-08-0-UI | Endothelial cell-specific molecule 1 | BF402357 |
| 4483 | UI-R-BT1-aqm-f-06-0-UI | Protein phosphatase 4, regulatory subunit 2 | BE102438 |
| 4484 | UI-R-BX0-arb-a-05-0-UI | Solute carrier family 25 (mitochondrial carrier, phosphate carrier), member 25 | BE103366 |
| 4485 | UI-R-BJ1-ato-a-06-0-UI | CDC-like kinase 2 | BE099313 |
| 4486 | UI-R-BJ1-ats-a-12-0-UI | REV3-like, catalytic subunit of DNA polymerase zeta RAD54 like | BE100131 |
| 4487 | UI-R-BJ1-atz-f-01-0-UI | Arsenic (+3 oxidation state) methyltransferase | CK839422 |
| 4488 | UI-R-BJ1-auh-d-11-0-UI | Signal transducer and activator of transcription interacting protein 1 | BE101129 |
| 4489 | UI-R-BJ1-awa-c-12-0-UI | Rhesus blood group CE and D | CK844295 |
| 4490 | UI-R-BJ1-awf-a-04-0-UI | NNX3 | BE112976 |
| 4491 | UI-R-BJ1-awp-c-07-0-UI | ATPase, aminophospholipid transporter (APLT), class I, type 8A, member 1 | CK844473 |
| 4492 | UI-R-CA0-awx-g-06-0-UI | Transcribed locus | BE107868 |
| 4493 | UI-R-BO1-aja-c-10-0-UI | Protocadherin gamma subfamily C, 3 | AW526288 |
| 4494 | UI-R-BO1-ajg-h-10-0-UI | Arylsulfatase A | BF563218 |
| 4495 | UI-R-BO1-ajl-d-12-0-UI | hypothetical protein FLJ32954 | AW528443 |
| 4496 | UI-R-BO1-ajr-f-10-0-UI | Ubiquitin carboxyl-terminal esterase L3 (ubiquitin thiolesterase) | AW528639 |
| 4497 | UI-R-C4-alb-f-12-0-UI | Sema domain, immunoglobulin domain (Ig), short basic domain, secreted, (semaphorin) 3 F | AW530809 |
| 4498 | UI-R-C4-alj-g-03-0-UI | Adenosine deaminase, RNA-specific | AW532213 |
| 4499 | UI-R-C4-alq-c-04-0-UI | Lymphotoxin B | AW534186 |
| 4500 | UI-R-BT1-ame-d-03-0-UI | Alanine and arginine rich domain containing protein | BE107658 |
| 4501 | UI-R-BU0-apj-h-02-0-UI | RIKEN cDNA 4930444A02 | BE096173 |
| 4502 | UI-R-BO1-aqb-a-12-0-UI | Prominin 1 | BE097735 |
| 4503 | UI-R-AA1-aab-h-08-0-UI | Striatin, calmodulin binding protein 4 | CK843245 |
| 4504 | UI-R-AF1-aar-c-12-0-UI | Polymerase (RNA) III (DNA directed) polypeptide H | CK840618 |
| 4505 | UI-R-Y0-acl-d-01-0-UI | Fibroblast growth factor 12 | BF524044 |
| 4506 | UI-R-Y0-acw-d-10-0-UI | RIKEN cDNA 1190003A07 | AI764230 |
| 4507 | UI-R-BJ0-adn-b-03-0-UI | N-terminal asparagine amidohydrolase | AW251881 |
| 4508 | UI-R-BJ0-aei-g-09-0-UI | Glycolipid transfer protein | AW254426 |
| 4509 | UI-R-BJ0p-agh-g-06-0-UI | G elongation factor | CK845318 |
| 4510 | UI-R-BO0-agq-b-12-0-UI | GTP-binding protein 8 | BF567557 |
| 4511 | UI-R-BO0-ahk-c-10-0-UI | SEC23B | AW522707 |
| 4512 | UI-R-BO0-ahs-a-11-0-UI | Mannoside acetyl glucosaminyltransferase 3 | BF567210 |
| 4513 | UI-R-C3-st-b-04-0-UI | Claudin 1 | AI535225 |
| 4514 | UI-R-C3-th-b-11-0-UI | 4930431B09Rik protein | AI547973 |
| 4515 | UI-R-C3-tm-f-09-0-UI | Fc fragment of IgG, low affinity IIIa, receptor | AI710030 |
| 4516 | UI-R-C3-tu-d-02-0-UI | Transcribed locus | AI549481 |
| 4517 | UI-R-AB0-vy-h-02-0-UI | Yip1 domain family, member 3 | CK838531 |
| 4518 | UI-R-AD0-wi-g-10-0-UI | Parn protein | BF524593 |
| 4519 | UI-R-AG0-ww-g-05-0-UI | Acylpeptide hydrolase | AI579268 |
| 4520 | UI-R-AE0-xh-c-12-0-UI | Proteasome (prosome, macropain) subunit, beta type 5 | CK844763 |
| 4521 | UI-R-AD1-zm-d-03-0-UI | Ceroid lipofuscinosis, neuronal 3, juvenile | CK839677 |
| 4522 | UI-R-AD1-zr-e-05-0-UI | Mammary tumor virus receptor 2 | CK842435 |
| 4523 | UI-R-C0-ht-b-12-0-UI | AL023001 protein | BF550694 |
| 4524 | UI-R-C0-il-b-06-0-UI | Zinc finger and BTB domain containing 11 | AI028900 |
| 4525 | UI-R-Y0-mg-b-08-0-UI | RAN binding protein 3 isoform RANBP3-b | AI112494 |
| 4526 | UI-R-C2-mt-f-03-0-UI | G protein-coupled receptor 107 | AI070919 |
| 4527 | UI-R-C2p-np-f-04-0-UI | G protein-coupled receptor 137B | BF542593 |
| 4528 | UI-R-C2p-oc-d-10-0-UI | RIKEN cDNA 1500003O22 | AI137525 |
| 4529 | UI-R-BT0-qk-a-02-0-UI | Kelch-like 11 | AI454666 |
| 4530 | UI-R-C2p-qp-d-06-0-UI | Neutrophil cytosolic factor 1 | BF545680 |
| 4531 | UI-R-C2p-qu-f-05-0-UI | Hypothetical protein LOC680259 | AI555002 |
| 4532 | UI-R-C2p-re-e-09-0-UI | vang, van gogh-like 1 | BF546055 |
| 4533 | UI-R-E0-bx-a-12-0-UI | Splicing factor, arginine/serine rich 9 | AA859734 |
| 4534 | UI-R-E0-cb-g-11-0-UI | PFTAIRE protein kinase 1 | BF559030 |
| 4535 | UI-R-E0-ck-d-05-0-UI | CDNA clone IMAGE:7460165 | BF554952 |
| 4536 | UI-R-E0-cr-g-03-0-UI | Solute carrier family 25 (mitochondrial carrier, brain), member 14 | BF555150 |
| 4537 | UI-R-A1-dw-e-09-0-UI | Oxytocin | AA901242 |
| 4538 | UI-R-A1-eb-a-01-0-UI | chromosome 1 open reading frame 50 | AA924844 |
| 4539 | UI-R-A1-eg-d-06-0-UI | Phosphatidic acid phosphatase type 2B | AA924925 |
| 4540 | UI-R-A1-el-a-12-0-UI | DKFZP566K1924 protein | AA926035 |
| 4541 | UI-R-C0-gx-g-07-0-UI | mKIAA0738 protein | AA964744 |
| 4542 | UI-R-C0-hn-g-02-0-UI | Actin-binding LIM protein 1 | BF557048 |
| 4543 | UI-R-C2-no-c-03-0-UI | Selenophosphate synthetase 1 | AI070739 |
| 4544 | UI-R-C3-sm-d-06-0-UI | 5,10-methenyltetrahydrofolate synthetase (5-formyltetrahydrofolate cyclo-ligase) | BF542664 |
| 4545 | UI-R-BO0-agn-d-05-0-UI | RIO kinase 2 | BF566561 |
| 4546 | UI-R-BJ1-auk-e-10-0-UI | EGF-like module containing, mucin-like, hormone receptor-like sequence 1 | CK839638 |
| 4547 | UI-R-A0-ao-f-08-0-UI | Glycine-N-acyltransferase | AA818998 |
| 4548 | UI-R-E0-ci-e-12-0-UI | Zinc finger protein 307 | AA874880 |
| 4549 | UI-R-A1-do-e-09-0-UI | Matrilin 1, cartilage matrix protein | BF558617 |
| 4550 | UI-R-A1-ej-e-10-0-UI | SMC6 structural maintenance of chromosomes 6-like 1 | AA955217 |
| 4551 | UI-R-C1-lg-h-08-0-UI | Dopey family member 2 | AI059699 |
| 4552 | UI-R-C2p-nr-e-08-0-UI | Vestigial like 1 homolog | AI113317 |
| 4553 | UI-R-C0-ha-e-10-0-UI | DNA segment, Chr 7, ERATO Doi 462, expressed | AA965032 |
| 4554 | UI-R-C0-hf-e-05-0-UI | Germ cell-less homolog 1 | AA996718 |
| 4555 | UI-R-C0-jc-g-09-0-UI | BRF2, subunit of RNA polymerase III transcription initiation factor, BRF1-like | AI030418 |
| 4556 | UI-R-C0-jm-d-11-0-UI | START domain containing 10 | AI043768 |
| 4557 | UI-R-C0-jr-a-05-0-UI | Transcribed locus | AI030051 |
| 4558 | UI-R-C1-ju-d-06-0-UI | Mitochondrial ribosomal protein S14 | AI045287 |
| 4559 | UI-R-A0-ao-h-11-0-UI | Defensin beta 4 | AA819022 |
| 4560 | UI-R-A1-ew-a-05-0-UI | Gonadotropin releasing hormone receptor | AI146077 |
| 4561 | UI-R-Y0-ml-a-10-0-UI | Phosphoglycerate mutase 2 | AI112130 |
| 4562 | UI-R-A0-av-c-07-0-UI | Cyclin D2 | BF548337 |
| 4563 | UI-R-E1-fg-c-07-0-UI | Scavenger receptor class B, member 1 | AA956371 |
| 4564 | UI-R-C0-it-c-06-0-UI | Inter-alpha trypsin inhibitor, heavy chain 3 | AI030246 |
| 4565 | UI-R-A0-ba-c-12-0-UI | RAD23b homolog | BF558954 |
| 4566 | UI-R-E1-fw-a-02-0-UI | All-trans-13,14-dihydroretinol saturase | BF560365 |
| 4567 | UI-R-Y0-lt-g-06-0-UI | Crystallin, beta A2 | AI070317 |
| 4568 | UI-R-E1-ft-a-05-0-UI | Transcribed locus | AA956802 |
| 4569 | UI-R-A0-aw-c-06-0-UI | Retinoic acid receptor responder (tazarotene induced) 1 | AA819288 |
| 4570 | UI-R-A0-az-e-05-0-UI | Cornichon homolog | BF549783 |
| 4571 | UI-R-E1-gk-h-01-0-UI | Cytotoxic granule-associated RNA binding protein 1 | AA963798 |
| 4572 | UI-R-C0-gv-c-04-0-UI | XP_001092038.1 fatso | BF557299 |
| 4573 | UI-R-E0-cb-a-05-0-UI | Aldo-keto reductase family 1, member A1 | AA875038 |
| 4574 | UI-R-E0-cg-g-07-0-UI | Histidine decarboxylase | BF555053 |
| 4575 | UI-R-E1-fa-h-02-0-UI | Delta-like 3 | AA955549 |
| 4576 | UI-R-E1-fl-c-08-0-UI | Spleen tyrosine kinase | AA956939 |
| 4577 | UI-R-E1-fv-e-03-0-UI | Transcribed locus | AA957761 |
| 4578 | UI-R-C0-hy-g-02-0-UI | S100 calcium-binding protein A4 | BF557654 |
| 4579 | UI-R-A0-ba-e-08-0-UI | Testis enhanced gene transcript | AA819031 |
| 4580 | UI-R-E1-fw-b-12-0-UI | Glutamate receptor, ionotropic, N-methyl D-asparate-associated protein 1 (glutamate binding) | AA957470 |
| 4581 | UI-R-C0-hb-d-05-0-UI | Nudix (nucleoside diphosphate linked moiety X)-type motif 4 | BF557711 |
| 4582 | UI-R-C0-jd-c-06-0-UI | CASK interacting protein 1 | AI030734 |
| 4583 | UI-R-Y0-lv-f-11-0-UI | XP_001110972.1 synaptojanin 2 binding protein isoform 1 | AI070523 |
| 4584 | UI-R-C2-mw-c-05-0-UI | Mitochondrial ribosomal protein S31 | AI385284 |
| 4585 | UI-R-C2-na-f-03-0-UI | XP_914429.1 retinoblastoma-associated protein 140 isoform 7 | AI070982 |
| 4586 | UI-R-C2-nc-g-10-0-UI | A disintegrin and metalloproteinase domain 9 (meltrin gamma) | AI071724 |
| 4587 | UI-R-C2-nn-e-04-0-UI | RGD1563866 | AI072615 |
| 4588 | UI-R-C2p-nq-f-11-0-UI | Kelch-like 26 | AI136694 |
| 4589 | UI-R-C2p-nw-b-04-0-UI | Zinc finger X-linked protein ZXDB | AI136549 |
| 4590 | UI-R-C2p-ny-d-03-0-UI | RIKEN cDNA 1300017J02 | AI136120 |
| 4591 | UI-R-A0-bm-g-06-0-UI | 5-oxoprolinase (ATP-hydrolysing) | AA866338 |
| 4592 | UI-R-E0-bu-g-11-0-UI | Lectin, galactose binding, soluble 3 | AA859797 |
| 4593 | UI-R-C0-ie-g-06-0-UI | Pancreatic lipase related protein 1 | BF561621 |
| 4594 | UI-R-C0-ij-d-11-0-UI | Presenilin 1 | BF551086 |
| 4595 | UI-R-C0-jo-f-08-0-UI | Hypothetical LOC298077 | BF554685 |
| 4596 | UI-R-C1-jw-e-04-0-UI | Myosin X | AI044576 |
| 4597 | UI-R-C1-jx-h-06-0-UI | Transmembrane BAX inhibitor motif containing 1 | AI044338 |
| 4598 | UI-R-C1-jz-c-12-0-UI | Endothelial differentiation, lysophosphatidic acid G-protein-coupled receptor, 2 | BF545611 |
| 4599 | UI-R-C1-kp-g-09-0-UI | Paralemmin | AI058672 |
| 4600 | UI-R-C1-kr-c-03-0-UI | COP9 (constitutive photomorphogenic) homolog, subunit 2 | BF554418 |
| 4601 | UI-R-C1-kt-f-01-0-UI | Protein tyrosine phosphatase, non-receptor type 6 | BF552604 |
| 4602 | UI-R-C1-ky-a-09-0-UI | Hus1 homolog | AI071523 |
| 4603 | UI-R-A0-aj-b-06-0-UI | Regulator of G-protein signaling 2 | AA858950 |
| 4604 | UI-R-A0-am-a-08-0-UI | Transcribed locus |  |
| 4605 | UI-R-A0-aq-e-10-0-UI | RNA binding motif protein 9 | BF549163 |
| 4606 | UI-R-A0-au-c-07-0-UI | Heterochromatin protein 1, binding protein 3 | AA818517 |
| 4607 | UI-R-E1-fn-c-06-0-UI | Fat tumor suppressor homolog | BF556831 |
| 4608 | UI-R-E1-fq-c-06-0-UI | CAMP responsive element binding protein 3 | AA956991 |
| 4609 | UI-R-E1-fu-e-08-0-UI | Gamma-aminobutyric acid receptor associated protein | AA957304 |
| 4610 | UI-R-E1-gq-g-10-0-UI | Presenilin associated, rhomboid-like | AA964791 |
| 4611 | UI-R-C0-hy-g-08-0-UI | SDA1 domain containing 1 | AA997613 |
| 4612 | UI-R-C0-ib-h-08-0-UI | NADP+-specific isocitrate dehydrogenase | AA998470 |
| 4613 | UI-R-C0-gv-f-12-0-UI | Solute carrier family 5 (sodium-dependent vitamin transporter), member 6 | AI113021 |
| 4614 | UI-R-C0-gz-f-08-0-UI | Glutamate cysteine ligase, modifier subunit | AA965220 |
| 4615 | UI-R-C0-jf-g-09-0-UI | Myogenin | AI030924 |
| 4616 | UI-R-C0-jn-g-01-0-UI | Ribosomal protein S13 | AI029832 |
| 4617 | UI-R-C1-jt-d-06-0-UI | CD47 antigen (Rh-related antigen, integrin-associated signal transducer) | AI044792 |
| 4618 | UI-R-C1-jz-h-03-0-UI | Translocator of inner mitochondrial membrane 44 | AI045558 |
| 4619 | UI-R-C1-lk-a-04-0-UI | ATPase, Ca++-sequestering | CK840733 |
| 4620 | UI-R-Y0-ls-a-02-0-UI | Aquaporin 5 | AI070064 |
| 4621 | UI-R-C2-my-e-04-0-UI | Cyclin G associated kinase | AI071179 |
| 4622 | UI-R-C2-ng-d-04-0-UI | Lactate dehydrogenase A | AI072330 |
| 4623 | UI-R-DK0-cfu-b-02-0-UI | Aquaporin 8 | BI290255 |
| 4624 | UI-R-DK0-cga-b-01-0-UI | Prolactin receptor | BI290761 |
| 4625 | UI-R-DK0-cgi-g-03-0-UI | opposite strand transcription unit to Stag3; Gats protein | BI290890 |
| 4626 | UI-R-CV2-cgs-d-04-0-UI | RIKEN cDNA 4732418C07 | BI296537 |
| 4627 | UI-R-DQ0-cja-a-22-0-UI | Transcribed locus |  |
| 4628 | UI-R-E0-dk-a-07-0-UI | 3-phosphoglycerate dehydrogenase | AA900851 |
| 4629 | UI-R-A1-dq-c-12-0-UI | Betaine-homocysteine methyltransferase | BF558671 |
| 4630 | UI-R-A1-du-c-02-0-UI | NP_066390.1 histone family, member A | AA955185 |
| 4631 | UI-R-E1-gf-b-02-0-UI | Polymerase (DNA directed), beta | AA957640 |
| 4632 | UI-R-E1-go-a-03-0-UI | Cytochrome c oxidase, subunit VIa, polypeptide 2 | BF556883 |
| 4633 | UI-R-DB0-byw-h-12-0-UI | Kallikrein, submaxillary gland S3 | BI285171 |
| 4634 | UI-R-DC0-bzq-a-11-0-UI | Seminal vesicle secretion 5 | BI281001 |
| 4635 | UI-R-CW0s-ccc-f-01-0-UI | GI:13385412-like protein splice form I | BI282891 |
| 4636 | UI-R-CX0s-ccn-e-06-0-UI | Death-associated protein | BI284210 |
| 4637 | UI-R-CX0s-ccs-a-10-0-UI | RIKEN cDNA 1110007L15 | BI284774 |
| 4638 | UI-R-DK0-cda-d-08-0-UI | Survival motor neuron domain containing 1 | BI288396 |
| 4639 | UI-R-DK0-cej-g-11-0-UI | SWI/SNF related, matrix associated, actin dependent regulator of chromatin, subfamily a, member 3 | BI294135 |
| 4640 | UI-R-DK0-cex-h-10-0-UI | Calumenin | BI296234 |
| 4641 | UI-R-DK0-cfe-a-01-0-UI | hedgehog-interacting protein | BI289052 |
| 4642 | UI-R-CM0-bjh-a-05-0-UI | FXYD domain-containing ion transport regulator 1 | BF395685 |
| 4643 | UI-R-CN0-blx-c-02-0-UI | Thyroid hormone receptor associated protein 6 | BF417791 |
| 4644 | UI-R-BT1-bms-f-12-0-UI | Peroxin 2 | BF412037 |
| 4645 | UI-R-BT1-bnp-b-11-0-UI | XP_001106147.1 thrombospondin repeat containing 1 isoform 1 | BF412563 |
| 4646 | UI-R-CA0-boh-g-07-0-UI | novel protein multidomain presynaptic cytomatrix protein piccolo (presynaptic cytomatrix protein) | BF413723 |
| 4647 | UI-R-BJ2-bqh-g-10-0-UI | Component of oligomeric golgi complex 4 | BF418600 |
| 4648 | UI-R-BJ2-bqo-e-04-0-UI | Acyl-Coenzyme A binding domain containing 3 | BF419134 |
| 4649 | UI-R-BJ2-bqt-d-07-0-UI | XP_001112351.1 BAH domain and coiled-coil containing 1 | CK843842 |
| 4650 | UI-R-CW0-bwe-h-12-0-UI | AI325464 protein | BI274954 |
| 4651 | UI-R-CZ0-byf-d-01-0-UI | Keratinocyte differentiation associated protein | BI277693 |
| 4652 | UI-R-DA0-byl-a-11-0-UI | Common salivary protein 1 | BI279407 |
| 4653 | UI-R-CA0-bft-f-12-0-UI | Cystatin TE-1 | BF393340 |
| 4654 | UI-R-CA0-bgh-a-06-0-UI | Neurocalcin delta | BF401048 |
| 4655 | UI-R-CA1-bix-p-15-0-UI | ELAV (embryonic lethal, abnormal vision, Drosophila)-like 2 (Hu antigen B) | BF403882 |
| 4656 | UI-R-CA1-bjd-a-08-0-UI | Rap guanine nucleotide exchange factor (GEF) 4 | BF403975 |
| 4657 | UI-R-CA1-bjf-h-12-0-UI | RAB33A, member of RAS oncogene family | BF406394 |
| 4658 | UI-R-CV1-brv-c-11-0-UI | Polymerase (DNA directed) kappa | BG372536 |
| 4659 | UI-R-CT0-bub-a-08-0-UI | Tubulin, gamma complex associated protein 6 | BG380753 |
| 4660 | UI-R-CT0-bun-a-10-0-UI | RAB4B, member RAS oncogene family | BG376147 |
| 4661 | UI-R-CU0-buu-f-02-0-UI | RIKEN cDNA 1810020E01 | BG376599 |
| 4662 | UI-R-CU0-bvf-g-09-0-UI | Solute carrier family 22 (organic cation transporter), member 2 | BG377840 |
| 4663 | UI-R-CA0-axc-e-10-0-UI | Carboxypeptidase M | BE108464 |
| 4664 | UI-R-CA0-axh-g-10-0-UI | Synapsin II | BE108731 |
| 4665 | UI-R-BS1-axv-g-10-0-UI | S-adenosylhomocysteine hydrolase-like 1 | BE115907 |
| 4666 | UI-R-BS1-ayn-a-02-0-UI | Solute carrier family 9 (sodium/hydrogen exchanger), member 2 | BE117286 |
| 4667 | UI-R-CA0-bap-f-03-0-UI | Cadherin 23 (otocadherin) | BE120191 |
| 4668 | UI-R-CA0-bay-e-06-0-UI | XP_001089512.1 Coiled-coil-helix-coiled-coil-helix domain-containing protein 2 (HCV NS2 trans-regulated protein) (NS2TP) isoform 2 | BE120698 |
| 4669 | UI-R-CA1-bbm-g-02-0-UI | Leucine rich repeat containing 7 | BF387464 |
| 4670 | UI-R-CA1-bcb-b-12-0-UI | MHC class I-like sequence | BF396090 |
| 4671 | UI-R-BS2-bef-e-10-0-UI | Polo-like kinase 1 | BF397952 |
| 4672 | UI-R-BS2-bev-f-11-0-UI | Transcribed locus | BF396834 |
| 4673 | UI-R-BU0-ane-h-08-0-UI | Mitogen activated protein kinase 10 | BF562409 |
| 4674 | UI-R-BU0-aoy-d-08-0-UI | Retinol binding protein 3, interstitial | BE095712 |
| 4675 | UI-R-BX0-ark-b-10-0-UI | Epidermal growth factor receptor | BE104053 |
| 4676 | UI-R-BX0-asd-f-02-0-UI | Leucine zipper, putative tumor suppressor 1 | BE105345 |
| 4677 | UI-R-BO1-asp-f-08-0-UI | Synaptotagmin II | BE106583 |
| 4678 | UI-R-BJ1-asy-e-05-0-UI | KIAA0240 | BE098338 |
| 4679 | UI-R-BJ1-aul-c-04-0-UI | Hypothetical LOC290577 | CK839509 |
| 4680 | UI-R-BJ1-aux-h-08-0-UI | RIKEN cDNA 9230117N10 | CK839877 |
| 4681 | UI-R-BJ1-avl-a-06-0-UI | CGI-35 protein | CK842521 |
| 4682 | UI-R-BJ1-avq-f-06-0-UI | Eukaryotic translation initiation factor 1B | BE109655 |
| 4683 | UI-R-BO0-ahw-d-03-0-UI | Potassium voltage-gated channel, shaker-related subfamily, member 1 | BF567965 |
| 4684 | UI-R-BO0-aic-e-11-0-UI | TRNA splicing endonuclease 2 homolog | AW523652 |
| 4685 | UI-R-BJ0p-aij-b-12-0-UI | Retinoic acid induced 14 | BF562748 |
| 4686 | UI-R-BJ0p-air-e-05-0-UI | RIKEN cDNA 6530418L21 | AW525760 |
| 4687 | UI-R-BO1-ajt-h-05-0-UI | Solute carrier family 27 (fatty acid transporter), member 2 | BF565216 |
| 4688 | UI-R-BT1-akb-c-12-0-UI | Transmembrane protein 66 | BF566241 |
| 4689 | UI-R-BT1-aki-g-09-0-UI | NudC domain containing 2 | BF563466 |
| 4690 | UI-R-BT1-ako-d-07-0-UI | X-linked myotubular myopathy gene 1 | BF565647 |
| 4691 | UI-R-BS0-amo-b-03-0-UI | Choroidermia | BF564958 |
| 4692 | UI-R-BU0-amz-c-09-0-UI | TNFAIP3 interacting protein 1 | BF564878 |
| 4693 | UI-R-AC0-yp-b-10-0-UI | Eukaryotic translation initiation factor 4E member 2 | AI704533 |
| 4694 | UI-R-AB1-yy-b-08-0-UI | PDZ domain containing 3 | AI703855 |
| 4695 | UI-R-AF1-aat-h-01-0-UI | RIKEN cDNA 0610037D15 | CK838441 |
| 4696 | UI-R-Y0-aba-f-04-0-UI | Epithelial membrane protein 1 | AI713132 |
| 4697 | UI-R-Y0-abn-b-07-0-UI | Neurotrophic tyrosine kinase, receptor, type 1 | AI715169 |
| 4698 | UI-R-Y0-aca-d-12-0-UI | Ryanodine receptor 1, skeletal muscle | BF521961 |
| 4699 | UI-R-BJ0p-aex-c-01-0-UI | Hydroxyacyl-Coenzyme A dehydrogenase/3-ketoacyl-Coenzyme A thiolase/enoyl-Coenzyme A hydratase (trifunctional protein), alpha subunit | AW520311 |
| 4700 | UI-R-BJ0p-aff-h-06-0-UI | Glutamate receptor, ionotropic, AMPA1 (alpha 1) | CK839307 |
| 4701 | UI-R-BJ0p-afm-f-07-0-UI | Homolog of zebrafish ES1 | CK841523 |
| 4702 | UI-R-BJ0p-afv-f-05-0-UI | G protein pathway suppressor 2 | CK841675 |
| 4703 | UI-R-C2p-ri-d-06-0-UI | Apolipoprotein A-IV | AI556613 |
| 4704 | UI-R-C2p-rq-c-02-0-UI | Transcribed locus | AI500789 |
| 4705 | UI-R-C2p-sa-f-03-0-UI | ADP-ribosylation factor interacting protein 1 | BF544801 |
| 4706 | UI-R-C3-sg-d-01-0-UI | hypothetical protein | AI511210 |
| 4707 | UI-R-C3-tz-c-01-0-UI | RE1-silencing transcription factor | BF522402 |
| 4708 | UI-R-G0-uf-h-08-0-UI | Zinc finger protein 324 | AI574933 |
| 4709 | UI-R-G0-us-g-05-0-UI | Carboxypeptidase A2 (pancreatic) | BF522689 |
| 4710 | UI-R-Y0-vb-a-11-0-UI | hypothetical protein FLJ20436 | AI576261 |
| 4711 | UI-R-AC1-xq-b-02-0-UI | Protein CGI-117 (Protein HSPC111) | CK845113 |
| 4712 | UI-R-AF0-ye-g-01-0-UI | Troponin I, skeletal, slow 1 | BF525158 |
| 4713 | UI-R-E1-fp-b-11-0-UI | A disintegrin and metalloprotease domain 5 | AA957169 |
| 4714 | UI-R-E1-ge-d-05-0-UI | Histone deacetylase 2 | AA957075 |
| 4715 | UI-R-C0-ja-b-08-0-UI | Oxysterol binding protein-like 11 | AI029924 |
| 4716 | UI-R-C1-jt-f-07-0-UI | BCL2/adenovirus E1B 19kDa-interacting protein 1, NIP2 | BF554121 |
| 4717 | UI-R-C1-km-c-09-0-UI | Mitogen-activated protein kinase kinase kinase kinase 3 | AI502900 |
| 4718 | UI-R-C1-le-d-08-0-UI | sarcoma antigen NY-SAR-27 | AI059633 |
| 4719 | UI-R-C2p-og-c-08-0-UI | Msx-interacting-zinc finger | AI137350 |
| 4720 | UI-R-BT0-pl-c-04-0-UI | Transmembrane protein 39b | AI144629 |
| 4721 | UI-R-BT0-pr-e-12-0-UI | Spermatogenesis associated 18 | AI145023 |
| 4722 | UI-R-BT0-px-h-01-0-UI | Protocadherin alpha 4 | AI145136 |
| 4723 | UI-R-A0-ad-f-03-0-UI | Amylo-1, 6-glucosidase, 4-alpha-glucanotransferase (glycogen debranching enzyme, glycogen storage disease type III) | BF524963 |
| 4724 | UI-R-A0-av-h-05-0-UI | RIKEN cDNA 0610038D11 | CK845698 |
| 4725 | UI-R-A0-bk-f-10-0-UI | Cirrhosis, autosomal recessive 1A | BF555244 |
| 4726 | UI-R-E0-bq-h-07-0-UI | Actin related protein 2/3 complex, subunit 3 | BF550089 |
| 4727 | UI-R-E0-cv-f-06-0-UI | NudE nuclear distribution gene E homolog like 1 (A. nidulans) | BF550160 |
| 4728 | UI-R-E0-dc-f-11-0-UI | Transcribed locus | AA901173 |
| 4729 | UI-R-E0-di-a-09-0-UI | Serine/arginine-rich protein specific kinase 1 | AA900731 |
| 4730 | UI-R-A1-dp-b-12-0-UI | Poly (ADP-ribose) glycohydrolase | AA901301 |
| 4731 | UI-R-A1-en-d-05-0-UI | Eukaryotic translation initiation factor 4A2 | AA925163 |
| 4732 | UI-R-A1-ex-f-01-0-UI | Mitogen-activated protein kinase-activated protein kinase 3 | AA955477 |
| 4733 | UI-R-CA0-bfl-d-04-0-UI | Tetratricopeptide repeat domain 12 | BF392624 |
| 4734 | UI-R-BJ2-bpv-d-06-0-UI | G0/G1 switch gene 2 | BF406636 |
| 4735 | UI-R-BJ2-bqx-f-06-0-UI | Dual specificity phosphatase 13 | BF407867 |
| 4736 | UI-R-E1-fo-h-06-0-UI | chromosome 20 open reading frame 6 | BF561002 |
| 4737 | UI-R-E1-gl-e-04-0-UI | RIKEN cDNA 6530403A03 | BF557545 |
| 4738 | UI-R-C0-hx-b-05-0-UI | hypothetical protein MGC10993 | BF557643 |
| 4739 | UI-R-C0-jq-b-08-0-UI | thyroid hormone receptor interactor 3 | BF550983 |
| 4740 | UI-R-C2p-qu-b-07-0-UI | Transcribed locus | AI555204 |
| 4741 | UI-R-C2p-rk-e-03-0-UI | minichromosome maintenance protein 8 isoform 1 | AI556302 |
| 4742 | UI-R-C0-ip-a-08-0-UI | rab6 GTPase activating protein (GAP and centrosome-associated) | BF551389 |
| 4743 | UI-R-C0-iv-h-11-0-UI | Enhancer of polycomb homolog 1 | CK840222 |
| 4744 | UI-R-C1-ka-f-05-0-UI | Vestigial like 2 homolog | BF542219 |
| 4745 | UI-R-C1-kn-b-06-0-UI | Angel homolog 2 | AI058332 |
| 4746 | UI-R-C1-kw-a-11-0-UI | Protein prenyltransferase alpha subunit repeat containing 1 | AI059413 |
| 4747 | UI-R-C1-ll-f-12-0-UI | TM2 domain containing 1 | AI060271 |
| 4748 | UI-R-E1-fq-e-01-0-UI | Gap junction membrane channel protein beta 2 | AA957263 |
| 4749 | UI-R-C0-hz-e-03-0-UI | Sialyltransferase 7F | AA998371 |
| 4750 | UI-R-C0-jp-g-06-0-UI | Integrin alpha FG-GAP repeat containing 3 | BF554714 |
| 4751 | UI-R-C1-lh-d-10-0-UI | Ischemia related factor NYW-1 | AI059785 |
| 4752 | UI-R-C0-ha-c-10-0-UI | P55 | BF556740 |
| 4753 | UI-R-C0-in-d-08-0-UI | EGF-like domain 7 | AA999046 |
| 4754 | UI-R-C0-jn-d-10-0-UI | Transcribed locus | AI030140 |
| 4755 | UI-R-C1-kp-a-06-0-UI | Regulator of chromosome condensation (RCC1) and BTB (POZ) domain containing protein 2 | AI058722 |
| 4756 | UI-R-A1-eu-b-12-0-UI | Zinc finger, MYND domain-containing 10 | AA926090 |
| 4757 | UI-R-E1-fb-g-09-0-UI | Testis expressed gene 261 | CK845260 |
| 4758 | UI-R-E1-ft-b-05-0-UI | Recombination activating gene 1 activating protein 1 | BF556145 |
| 4759 | UI-R-E1-fy-c-04-0-UI | Adenylosuccinate lyase | AA963616 |
| 4760 | UI-R-C0-hk-f-01-0-UI | Xeroderma pigmentosum, complementation group A | AA996961 |
| 4761 | UI-R-C0-il-a-09-0-UI | XP_394944.3 CG3173-PA [Apis mellifera] | AI029065 |
| 4762 | UI-R-E0-dh-f-01-0-UI | Ribosomal protein S6 kinase, polypeptide 1 | BF558047 |
| 4763 | UI-R-A1-ev-b-01-0-UI | Neuritin | AA926256 |
| 4764 | UI-R-C0-il-f-12-0-UI | Ribosomal protein L31 | AI029094 |
| 4765 | UI-R-C0-iu-c-06-0-UI | Activin A receptor type II-like 1 | AI030334 |
| 4766 | UI-R-E1-fw-f-07-0-UI | Eukaryotic translation initiation factor 2B, subunit 2 beta | AA957690 |
| 4767 | UI-R-BT0-py-g-03-0-UI | Early growth response 4 | AI145542 |
| 4768 | UI-R-C1-km-e-07-0-UI | GDP dissociation inhibitor 2 | BF552479 |
| 4769 | UI-R-C2-mq-h-05-0-UI | Ubiquitin carboxy-terminal hydrolase L1 | BF542243 |
| 4770 | UI-R-A0-bk-b-08-0-UI | Cytochrome P450, family 2, subfamily A, polypeptide 3a | AA819453 |
| 4771 | UI-R-E0-co-c-02-0-UI | Proteasome (prosome, macropain) 26S subunit, non-ATPase, 1 | BF555103 |
| 4772 | UI-R-C2-ne-f-03-0-UI | CLIP associating protein 2 | BF549639 |
| 4773 | UI-R-C2-ng-f-11-0-UI | XP_001106559.1 FYVE, RhoGEF and PH domain containing 6 isoform 2 | AI072357 |
| 4774 | UI-R-C2-nj-e-02-0-UI | Cytochrome b5 reductase 4 | BF553181 |
| 4775 | UI-R-C2-nl-d-05-0-UI | ELL associated factor 2 | AI071961 |
| 4776 | UI-R-A0-ad-e-05-0-UI | Glutathione-S-transferase, alpha type2 | AA818339 |
| 4777 | UI-R-A0-ao-f-05-0-UI | Palmitoyl-protein thioesterase 1 | AA818995 |
| 4778 | UI-R-A0-az-b-09-0-UI | Myogenic factor 6 | BF547668 |
| 4779 | UI-R-A0-bg-g-06-0-UI | Ribosomal protein S29 | AA866241 |
| 4780 | UI-R-E0-co-b-12-0-UI | Ribosomal protein S15a | BF555102 |
| 4781 | UI-R-E0-cw-e-11-0-UI | Chloride channel, nucleotide-sensitive, 1A | AA923932 |
| 4782 | UI-R-C0-ja-h-07-0-UI | RIKEN cDNA 2610029K21 | AI029955 |
| 4783 | UI-R-C0-ji-e-09-0-UI | Abhydrolase domain containing 14A | AI030452 |
| 4784 | UI-R-C1-kc-h-05-0-UI | CD3 antigen, gamma polypeptide | AI044631 |
| 4785 | UI-R-C1-kf-c-11-0-UI | ABI gene family, member 3 (NESH) binding protein | AI045072 |
| 4786 | UI-R-C1-ki-c-02-0-UI | Acyl-CoA thioesterase 4 (Peroxisomal acyl coenzyme A thioester hydrolase Ib) | AI044811 |
| 4787 | UI-R-C1-kk-b-12-0-UI | protein tyrosine phosphatase, receptor type, C polypeptide-associated protein | AI045153 |
| 4788 | UI-R-C1-la-c-12-0-UI | Prolactin family 2, subfamily b, member 1 | BF558472 |
| 4789 | UI-R-C1-le-e-12-0-UI | Transmembrane emp24 domain containing 3 | AI059643 |
| 4790 | UI-R-C1-lg-d-06-0-UI | Cholinergic receptor, nicotinic, epsilon polypeptide | BF549506 |
| 4791 | UI-R-C1-lp-f-06-0-UI | Transcribed locus | AI070008 |
| 4792 | UI-R-A0-bf-c-03-0-UI | Transcribed locus | BF548109 |
| 4793 | UI-R-E1-fa-c-11-0-UI | Sorting nexin 19 | AA955590 |
| 4794 | UI-R-E1-fg-b-09-0-UI | Sperm associated antigen 9 | BF560163 |
| 4795 | UI-R-E1-fk-h-01-0-UI | Integrin binding sialoprotein | BF556491 |
| 4796 | UI-R-C0-gw-g-12-0-UI | Solute carrier family 26, member 4 | BF557818 |
| 4797 | UI-R-C0-hd-f-03-0-UI | Resistin like alpha | AA996591 |
| 4798 | UI-R-C0-hi-b-11-0-UI | Guanylate kinase 1 | CK840143 |
| 4799 | UI-R-C0-hu-b-05-0-UI | XP_001104479.1 tripartite motif-containing 6 and tripartite motif-containing 34 | AA997970 |
| 4800 | UI-R-C0-it-b-04-0-UI | THAP domain containing, apoptosis associated protein 3 | AI030238 |
| 4801 | UI-R-C0-iy-c-11-0-UI | IBR domain containing 3 | AI029608 |
| 4802 | UI-R-C0-ht-g-10-0-UI | Deformed epidermal autoregulatory factor 1 | AA997917 |
| 4803 | UI-R-C0-iy-b-01-0-UI | Gap junction membrane channel protein beta 1 | BF552730 |
| 4804 | UI-R-C1-kf-c-01-0-UI | Eukaryotic translation elongation factor 1 alpha 2 | CK840704 |
| 4805 | UI-R-C1-km-h-05-0-UI | Cysteine sulfinic acid decarboxylase | AI045953 |
| 4806 | UI-R-C1-kw-a-04-0-UI | ATP-binding cassette, sub-family C (CFTR/MRP), member 1 | AI059506 |
| 4807 | UI-R-C1-ld-g-02-0-UI | Zinc finger protein 36, C3H type-like 1 | AI059256 |
| 4808 | UI-R-BT0-ps-c-01-0-UI | ATP synthase, H+ transporting, mitochondrial F0 complex, subunit F6 | AI145606 |
| 4809 | UI-R-C2p-oe-c-06-0-UI | Transcription factor 12 | AI137102 |
| 4810 | UI-R-BT0-pn-a-06-0-UI | Regulator of G-protein signaling 12 | AI144577 |
| 4811 | UI-R-A0-ad-h-08-0-UI | Heat shock factor binding protein 1 | AA818369 |
| 4812 | UI-R-CV2-chi-a-11-0-UI | Small cell adhesion glycoprotein | BI298418 |
| 4813 | UI-R-CV2-cil-c-08-0-UI | Sprouty homolog 2 | BI300901 |
| 4814 | UI-R-DN0-ciu-b-21-0-UI | KIAA2010 protein | BI291600 |
| 4815 | UI-R-DO0-ciw-f-07-0-UI | Coagulation factor II (thrombin) receptor | BI292631 |
| 4816 | UI-R-A1-dy-f-05-0-UI | Proteasome (prosome, macropain) subunit, beta type 4 | AA924299 |
| 4817 | UI-R-A1-eb-h-10-0-UI | 3-hydroxy-3-methylglutaryl-Coenzyme A synthase 1 | AA924800 |
| 4818 | UI-R-A1-ef-h-02-0-UI | Ribosomal protein L35 | CK840093 |
| 4819 | UI-R-A1-ek-d-09-0-UI | Fibrinogen, alpha polypeptide | AA925421 |
| 4820 | UI-R-C0-hg-f-04-0-UI | ADP-ribosylation factor 4 | AA996844 |
| 4821 | UI-R-C0-hn-e-07-0-UI | Linker of T-cell receptor pathways | AI138146 |
| 4822 | UI-R-CU0s-cbr-d-02-0-UI | FK506 binding protein 4 | BI279823 |
| 4823 | UI-R-CU0s-cbx-h-11-0-UI | XP_001100792.1 60S ribosomal protein L7a | BI282213 |
| 4824 | UI-R-DK0-cdg-c-04-0-UI | Calnexin | BI288553 |
| 4825 | UI-R-DK0-cds-c-11-0-UI | Chitobiase, di-N-acetyl- | BI293589 |
| 4826 | UI-R-DK0-cec-a-04-0-UI | chromosome 6 open reading frame 106 isoform a | BI294775 |
| 4827 | UI-R-DK0-cee-g-06-0-UI | Serine (or cysteine) peptidase inhibitor, clade A (alpha-1 antiproteinase, antitrypsin), member 12 | BI295014 |
| 4828 | UI-R-CA1-bjw-g-11-0-UI | Male germ cell-associated kinase | BF410379 |
| 4829 | UI-R-BJ2-bqz-e-01-0-UI | Coactosin-like 1 (Dictyostelium) | BF408079 |
| 4830 | UI-R-BJ2-bre-b-12-0-UI | Nidogen 1 | BF408476 |
| 4831 | UI-R-DK0-cfn-c-10-0-UI | Transcribed locus | BI289712 |
| 4832 | UI-R-BJ2-bor-a-11-0-UI | hypothetical protein BC018453 | BF414637 |
| 4833 | UI-R-CA0-bpb-f-06-0-UI | Fibroblast growth factor 14 | BF414602 |
| 4834 | UI-R-BJ2-bpo-b-12-0-UI | Desmin | BF406708 |
| 4835 | UI-R-BJ2-bpv-h-09-0-UI | Nuclear factor related to kappa B binding protein | CK843570 |
| 4836 | UI-R-CW0-bwn-b-03-0-UI | hypothetical protein E130310N06 | BI276015 |
| 4837 | UI-R-CX0-bxb-f-04-0-UI | Adaptor-related protein complex AP-4, beta 1 | BI275100 |
| 4838 | UI-R-CX0-bxj-g-10-0-UI | Thyrotropin releasing hormone | BI276733 |
| 4839 | UI-R-CY0-bxq-d-09-0-UI | Diacylglycerol O-acyltransferase homolog 2 | BI277463 |
| 4840 | UI-R-DE0-cae-d-09-0-UI | deleted in malignant brain tumors 1 isoform a precursor | BI279596 |
| 4841 | UI-R-CT0s-cat-f-01-0-UI | Myelin-associated oligodendrocytic basic protein | BI281206 |
| 4842 | UI-R-CA0-bhs-h-08-0-UI | Cytochrome b-561 domain containing 2 | BF403205 |
| 4843 | UI-R-CA1-bio-a-08-0-UI | Protamine 2 | BF404984 |
| 4844 | UI-R-CV1-bsc-c-08-0-UI | Guanine nucleotide binding protein, alpha o | BG372398 |
| 4845 | UI-R-CV1-bsm-g-02-0-UI | Neurexophilin 3 | BG374215 |
| 4846 | UI-R-CS0-btg-c-06-0-UI | Vesicle amine transport protein 1 homolog (T californica) | BG379391 |
| 4847 | UI-R-CT0-btw-c-08-0-UI | Polymerase (DNA directed), lambda | BG380365 |
| 4848 | UI-R-CV1-bvo-e-05-0-UI | Alcohol dehydrogenase 7 (class IV), mu or sigma polypeptide | BG378145 |
| 4849 | UI-R-CA0-bke-f-01-0-UI | ATPase, Ca++ transporting, ubiquitous | BF415770 |
| 4850 | UI-R-CA1-bkt-h-05-0-UI | Ttc15 protein | BF416624 |
| 4851 | UI-R-CA1-blf-d-01-0-UI | Carbonic anyhydrase 12 | BF409414 |
| 4852 | UI-R-BS1-ayv-a-09-0-UI | Nuclear autoantigenic sperm protein (histone-binding) | BE108332 |
| 4853 | UI-R-BS1-azc-f-07-0-UI | DEAD (Asp-Glu-Ala-Asp) box polypeptide 46 | BE109072 |
| 4854 | UI-R-BJ1-azq-b-02-0-UI | Acylphosphatase 1, erythrocyte (common) type | CK842246 |
| 4855 | UI-R-CA0-bae-c-08-0-UI | Hypothetical protein LOC691849 | BE119520 |
| 4856 | UI-R-CA1-bcm-d-09-0-UI | NPC1 (Niemann-Pick disease, type C1, gene)-like 1 | BF390205 |
| 4857 | UI-R-BS2-bdc-f-05-0-UI | Lipolysis stimulated lipoprotein receptor | BF388447 |
| 4858 | UI-R-BS2-bdm-b-09-0-UI | Zinc finger, MYND domain containing 11 | BF389301 |
| 4859 | UI-R-BS2-bdy-f-06-0-UI | CDNA clone IMAGE:7365240, containing frame-shift errors | BF396479 |
| 4860 | UI-R-CA0-bgv-h-08-0-UI | RIKEN cDNA C130022K22 gene | BF393865 |
| 4861 | UI-R-CA0-bhf-h-01-0-UI | XP_001087239.1 neurolysin isoform 3 | BF400841 |
| 4862 | UI-R-BT1-aqg-b-09-0-UI | Sorting nexin family member 27 | BE101622 |
| 4863 | UI-R-BT1-aqs-c-06-0-UI | Mitochondrial translational initiation factor 2 | BE102634 |
| 4864 | UI-R-BJ1-atj-c-10-0-UI | Lymphocyte antigen 6 complex, locus G6F | BE099520 |
| 4865 | UI-R-BJ1-atp-g-04-0-UI | Solute carrier family 27 (fatty acid transporter), member 3 | BE099649 |
| 4866 | UI-R-BJ1-atu-f-10-0-UI | Yippee-like 5 | CK841890 |
| 4867 | UI-R-BJ1-aue-d-10-0-UI | PEST-containing nuclear protein | BE100522 |
| 4868 | UI-R-BJ1-avx-g-04-0-UI | G protein-coupled receptor kinase-interactor 2 | BE111782 |
| 4869 | UI-R-BJ1-awb-h-08-0-UI | Protein phosphatase 1, regulatory (inhibitor) subunit 13B | CK844340 |
| 4870 | UI-R-BJ1-awh-g-12-0-UI | SEL1 domain containing protein RGD735029 | BE113823 |
| 4871 | UI-R-BJ1-awt-g-02-0-UI | MutY homolog | CK840052 |
| 4872 | UI-R-BO1-aix-e-08-0-UI | MAM domain containing glycosylphosphatidylinositol anchor 2 | AW526513 |
| 4873 | UI-R-BO1-ajf-a-09-0-UI | KIAA1409 protein | BF563122 |
| 4874 | UI-R-BO1-ajh-g-04-0-UI | RAS guanyl releasing protein 1 | BF563256 |
| 4875 | UI-R-BO1-ajo-b-08-0-UI | Storkhead box 2 | BF565110 |
| 4876 | UI-R-C4-akx-e-06-0-UI | Flotillin 2 | BF564029 |
| 4877 | UI-R-C4-ale-h-03-0-UI | GDP-mannose pyrophosphorylase B | BF563510 |
| 4878 | UI-R-C4-aln-d-07-0-UI | XP_001087527.1 Dual specificity protein phosphatase 13 (Testis- and skeletal-muscle-specific DSP) (Dual specificity phosphatase SKRP4) | AW534423 |
| 4879 | UI-R-C4-alv-h-02-0-UI | ADP-ribosylation factor GTPase-activating protein 3 (ARF GAP 3) | BF564523 |
| 4880 | UI-R-BU0-apg-c-11-0-UI | Ubiquitin-activating enzyme E1-like | BE096453 |
| 4881 | UI-R-BO1-apu-f-03-0-UI | Sparc/osteonectin, cwcv and kazal-like domains proteoglycan 1 | BE097213 |
| 4882 | UI-R-AA1-zw-b-07-0-UI | Voltage-dependent anion channel 3 | CK839168 |
| 4883 | UI-R-AG1-aak-d-01-0-UI | Coiled-coil domain containing 51 | CK838400 |
| 4884 | UI-R-Y0-acg-c-07-0-UI | Ankyrin repeat and SOCS box-containing protein 8 | BF523836 |
| 4885 | UI-R-Y0-acs-d-04-0-UI | Retbindin | AI763817 |
| 4886 | UI-R-Y0-acy-h-11-0-UI | Aldo-keto reductase family 7, member A2 (aflatoxin aldehyde reductase) | AI764110 |
| 4887 | UI-R-BJ0-aea-h-01-0-UI | Reticulon 4 | CK841110 |
| 4888 | UI-R-BJ0p-agd-d-06-0-UI | Vesicle-associated membrane protein 5 | CK841328 |
| 4889 | UI-R-BO0-agk-h-08-0-UI | Testis expressed gene 10 | AW521245 |
| 4890 | UI-R-BO0-agu-e-01-0-UI | Cdc42 guanine nucleotide exchange factor (GEF) 9 | BF567668 |
| 4891 | UI-R-BO0-ahn-b-09-0-UI | Tropomodulin 2 | BF567833 |
| 4892 | UI-R-C3-sq-a-05-0-UI | B230212L03Rik protein | AI535154 |
| 4893 | UI-R-C3-sy-d-05-0-UI | Protein C | AI535462 |
| 4894 | UI-R-C3-tj-e-06-0-UI | novel protein | AI709780 |
| 4895 | UI-R-C3-tp-g-04-0-UI | Signal transducer and activator of transcription 6 | AI548682 |
| 4896 | UI-R-AB0-vq-c-11-0-UI | Rho GDP dissociation inhibitor (GDI) alpha | CK840343 |
| 4897 | UI-R-AD0-we-d-12-0-UI | RIKEN cDNA 1700037H04 | CK842303 |
| 4898 | UI-R-AA0-ws-h-09-0-UI | Suppressor of initiator codon mutations, related sequence 1 | CK840446 |
| 4899 | UI-R-AG0-xa-f-04-0-UI | LIM and cysteine-rich domains 1 | AI602501 |
| 4900 | UI-R-AE1-zg-a-01-0-UI | JTV1 | AI706337 |
| 4901 | UI-R-AD1-zo-e-05-0-UI | XP_001118138.1 latent transforming growth factor beta binding protein 3 | CK842366 |
| 4902 | UI-R-C0-hr-a-03-0-UI | ATP synthase, H+ transporting, mitochondrial F0 complex, subunit e | AA997071 |
| 4903 | UI-R-C0-ib-b-12-0-UI | Exportin, tRNA (nuclear export receptor for tRNAs) | BF561667 |
| 4904 | UI-R-Y0-lw-h-10-0-UI | KIAA1052 protein | AI072860 |
| 4905 | UI-R-Y0-mn-f-12-0-UI | Signal recognition particle 14 | AI112565 |
| 4906 | UI-R-C2-mw-h-08-0-UI | Pyrimidinergic receptor P2Y, G-protein coupled, 6 | BF521779 |
| 4907 | UI-R-C2p-nt-h-11-0-UI | Dihydrolipoamide S-acetyltransferase (E2 component of pyruvate dehydrogenase complex) | BF553293 |
| 4908 | UI-R-BT0-qh-a-07-0-UI | XP_001091685.1 transient receptor potential cation channel, subfamily V, member 6 | AI146221 |
| 4909 | UI-R-C2p-qm-e-08-0-UI | ATP-binding cassette, sub-family C (CFTR/MRP), member 6 | BF522303 |
| 4910 | UI-R-C2p-qr-h-08-0-UI | Nuclear transcription factor-Y gamma | AI555735 |
| 4911 | UI-R-C2p-rb-a-08-0-UI | Transcribed locus | BF547298 |
| 4912 | UI-R-E0-bu-h-02-0-UI | Ubc6p homolog | AA875146 |
| 4913 | UI-R-E0-by-h-04-0-UI | Protease, serine, 23 | AA900718 |
| 4914 | UI-R-E0-cg-c-07-0-UI | 3-hydroxyisobutyryl-Coenzyme A hydrolase | BF550372 |
| 4915 | UI-R-E0-cm-b-06-0-UI | Reticulocalbin 1 | AA875563 |
| 4916 | UI-R-A1-dt-h-05-0-UI | Dmx-like 1 | BF558799 |
| 4917 | UI-R-A1-dy-e-03-0-UI | Src-like adaptor | AA924285 |
| 4918 | UI-R-A1-ed-g-08-0-UI | Solute carrier family 12 (potassium/chloride transporters), member 8 | BF556220 |
| 4919 | UI-R-A1-ei-f-07-0-UI | Transcribed locus | AI111943 |
| 4920 | UI-R-E1-go-c-03-0-UI | SH3-domain GRB2-like B1 (endophilin) | AA964563 |
| 4921 | UI-R-C0-hf-h-11-0-UI | Fibroblast growth factor 8 | AA996699 |
| 4922 | UI-R-E1-gq-a-08-0-UI | chromosome 16 open reading frame 5 | BF561161 |
| 4923 | UI-R-AD1-zs-c-09-0-UI | C21orf70 protein | CK841027 |
| 4924 | UI-R-BO1-ajt-h-12-0-UI | LOC362793 | AW527494 |
| 4925 | UI-R-CA0-bab-f-04-0-UI | hypothetical protein FLJ20519 | BE118778 |
| 4926 | UI-R-E0-by-d-02-0-UI | hypothetical protein FLJ10204 | BF547836 |
| 4927 | UI-R-E0-dc-c-11-0-UI | XP_001085806.1 elongation protein 4 homolog | AA900917 |
| 4928 | UI-R-A1-du-h-08-0-UI | Intraflagellar transport 74 homolog | BF555700 |
| 4929 | UI-R-A1-ex-b-08-0-UI | Probable phospholipid-transporting ATPase ID (ATPase class I type 8B member 2) | AA955397 |
| 4930 | UI-R-Y0-mp-b-04-0-UI | Transcribed locus | AI111841 |
| 4931 | UI-R-C2p-od-d-12-0-UI | RIKEN cDNA 4932432N11 gene | AI136386 |
| 4932 | UI-R-C0-hb-h-08-0-UI | pericentrin | BF559996 |
| 4933 | UI-R-C0-hh-b-07-0-UI | Tumor necrosis factor, alpha-induced protein 8 | AA996875 |
| 4934 | UI-R-C0-je-d-03-0-UI | LUC7-like 2 | AI030819 |
| 4935 | UI-R-C0-jn-h-08-0-UI | XP_001070204.1 Kinesin-like protein KIF1A (Axonal transporter of synaptic vesicles) | AI030157 |
| 4936 | UI-R-C1-js-h-04-0-UI | Polymerase (DNA directed) sigma | AI044451 |
| 4937 | UI-R-C1-jw-d-01-0-UI | USP6 N-terminal like | BF550833 |
| 4938 | UI-R-E0-bp-g-08-0-UI | Ribosomal protein S27a | BF547955 |
| 4939 | UI-R-C1-kc-c-07-0-UI | Transcribed locus |  |
| 4940 | UI-R-A0-al-d-10-0-UI | Phosphofructokinase, platelet | AA819266 |
| 4941 | UI-R-A0-bf-g-12-0-UI | Splicing factor 1 | BF548130 |
| 4942 | UI-R-E1-fx-c-10-0-UI | SH2-B PH domain containing signaling mediator 1 | AA963098 |
| 4943 | UI-R-BT0-ps-e-08-0-UI | SH3 domain binding protein CR16 | AI145632 |
| 4944 | UI-R-A1-eu-g-02-0-UI | Chitinase 3-like 1 | BF556944 |
| 4945 | UI-R-E1-gj-g-07-0-UI | COMM domain containing 3 | AA963468 |
| 4946 | UI-R-A0-aj-a-11-0-UI | Ras homolog enriched in brain | AA858945 |
| 4947 | UI-R-A0-bh-c-06-0-UI | DEAH (Asp-Glu-Ala-Asp/His) box polypeptide 57 | BF547711 |
| 4948 | UI-R-A0-ax-g-06-0-UI | Bernardinelli-Seip congenital lipodystrophy 2 homolog | BF549716 |
| 4949 | UI-R-A1-es-b-06-0-UI | mKIAA1757 protein | BF556361 |
[truncated: 335,716 more chars]
